# Synthesis and electrochemical properties of molybdenum nitrido complexes supported by redox-active NHC and MIC ligands†

**DOI:** 10.1039/d4dt02405b

**Published:** 2024-11-01

**Authors:** Daniel Leitner, Florian R. Neururer, Stephan Hohloch

**Affiliations:** a Department of General, Inorganic and Theoretical Chemistry, University of Innsbruck Innrain 80-82 6020 Innsbruck Austria Stephan.Hohloch@uibk.ac.at

## Abstract

We report the synthesis of a series of molybdenum nitrido complexes supported by bis-phenolate N-heterocyclic and mesoionic carbenes (NHC & MIC). The reaction between MoN(O^*t*^Bu)_3_ and the corresponding azolium salts [H_3_L^1^]Cl and [H_3_L^2^]Cl (with L^1^ = bis-phenolate triazolylidene and L^2^ = bis-phenolate benzimidazolylidene) gives clean access to the corresponding NHC/MIC complexes 1-Cl and 2-Cl. Electrochemical investigations of these complexes showed that they can be reversibly reduced at potentials of −1.13 and −1.01 V *vs.* Fc/[Fc]^+^ and the reduced complexes [1-Cl]^−^ and [2-Cl]^−^ can be cleanly isolated after chemical reduction with one equivalent of decamethylcobaltocene. Exchange of the halide atoms is furthermore reported to give a series of nitrido complexes supported by *tert*-butanolate (1-O^*t*^Bu and 2-O^*t*^Bu), perfluoro-*tert*-butanolate (1-O^*t*^Bu^F9^ and 2-O^*t*^Bu^F9^), tritylate (1-OCPh_3_ and 2-OCPh_3_), mesitolate (1-OMes and 2-OMes), thio-*tert*-butanolate (1-S^*t*^Bu), thiotritylate (1-SCPh_3_ and 2-SCPh_3_) and thiomesitolate complexes (1-SMes). The electrochemical properties of all complexes were evaluated and compared. All isolated complexes were characterized by multinuclear and multidimensional NMR spectroscopy and (if applicable) by EPR spectroscopy. Furthermore, the reactivity of 1-Cl and 2-Cl in the presence of protons and decamethylcobaltocene was investigated, which shows facile extrusion of ammonia, yielding diamagnetic bis-molybdenum(iii) complexes 3 and 4.

## Abstract 2

We report the synthesis of a series of molybdenum nitrido complexes supported by bis-phenolate N-heterocyclic and mesoionic carbenes, to investigate their (electro-)chemical properties in the absence and presence of protons for ammonia production.

## Graphical abstract

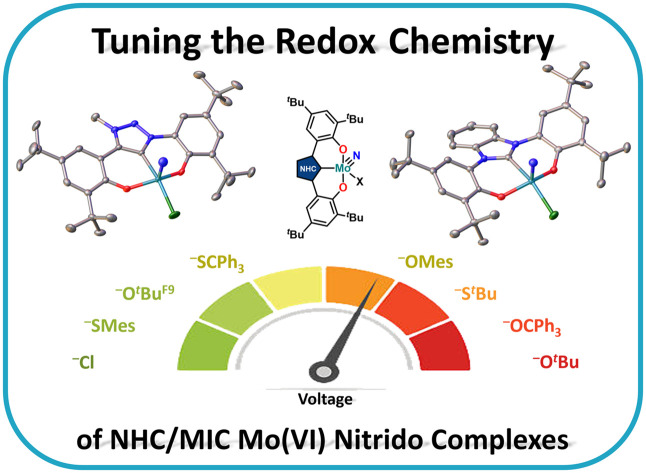

## Introduction

**Fig. 1:**
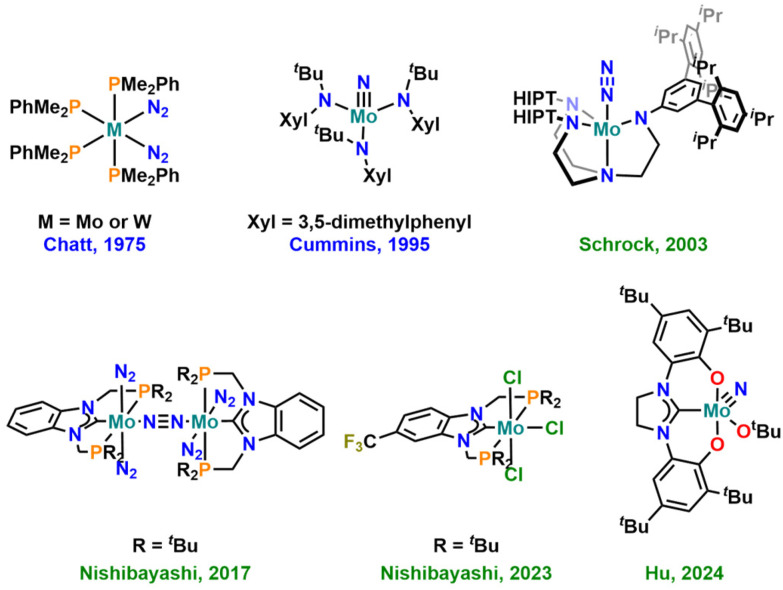
Selected milestones of molybdenum-based complexes for dinitrogen fixation and functionalisation. The green labels indicate that the molecule is catalytic in nitrogen functionalisation, while the blue labels indicate stoichiometric reactivity.

The effective conversion of nitrogen into ammonia under ambient conditions (1 atm N_2_ at room temperature) is one of the key challenges in modern chemistry. Surely, and despite its large energy consumption, this process is efficiently solved on an industrial scale, using the Haber–Bosch process.^1^ However, given the promising results of ammonia also acting as an energy/hydrogen storage system and potential future fuel,^2^ the development of delocalized ammonia generators is an important goal.^3^ This requires catalysts that operate under mild or, at best, ambient conditions, efficiently producing ammonia and related compounds, *e.g.* tris-trimethylsilylamines (N(SiMe_3_)_3_) or nitrogen-functionalized organic molecules.^4^ Within the past few decades, molybdenum-based catalysts, in particular, have been thoroughly studied in this context (Fig. 1),^5^ and also other metals such as titanium,^6^ vanadium,^7^ chromium,^8^ tungsten,^9^ rhenium,^10^ iron,^11^ cobalt,^12^ manganese^13^ or the lanthanides,^14^ the actinides^15^ and boron^16^ have been utilized to facilitate this reaction. For molybdenum, one of the most promising systems so far incorporated the use of tridentate PCP chelating N-heterocyclic carbene (NHC) ligands.^17^ Computational methods have further shown that the substitution pattern (and thus the electronic structure) on the NHC ligand seems to play a crucial role in this reaction.^18^ Given these results and the large variety of NHC ligands reported to date,^19^ it is particularly noteworthy that the number of NHC ligands investigated in this context is still very limited.

Based on the seminal works by Kawaguchi,^20^ Grubbs,^21^ Bercaw^22,23^ and Bellemin-Laponnaz,^24–26^ we and others^27^ have recently started to investigate the chemistry of bis-phenolate-supported normal N-heterocyclic carbene (*n*NHC) and mesoionic carbene (MIC) ligands of the (benz)imidazolylidene- and 1,2,3-triazolylidene type in the chemistry of (early) transition metals.^28–33^ This revealed an unprecedented catalytic potential and metal–carbene stability of benzimidazolylidene-based systems in deoxygenation catalysis.^28,30^ Given the advantageous properties of triazolylidenes,^34^ combined with their modular synthesis,^35–37,38^ these ligands were often found to enhance the catalytic potential of metal complexes,^39^ in some cases outperforming *n*NHC congeners.^37,40–42^ Thus, we believe that both these ligands are valuable and promising candidates for the preparation of efficient and stable ammonia evolution catalysts. This is further supported by the recent appearance of a bis-phenolate *n*NHC molybdenum nitrido complex in the literature that shows unprecedented turnover numbers and selectivity in the catalytic silylation of dinitrogen using TMS–Cl and KC_8_.^43^ Here, we present the synthesis and in-depth characterisation of overall 18 new NHC- and MIC-supported molybdenum nitrido complexes by spectroscopic (NMR, EPR, UV-Vis-NIR, IR), electrochemical (CV) and structural means. Preliminary results show that these complexes can be reductively denitrogenated under protic conditions, leading to dimeric Mo(iii) complexes with a direct Mo–Mo bond, which is indicative of their catalytic potential in ammonia evolution from dinitrogen.

## Results and discussion

**Scheme 1:**
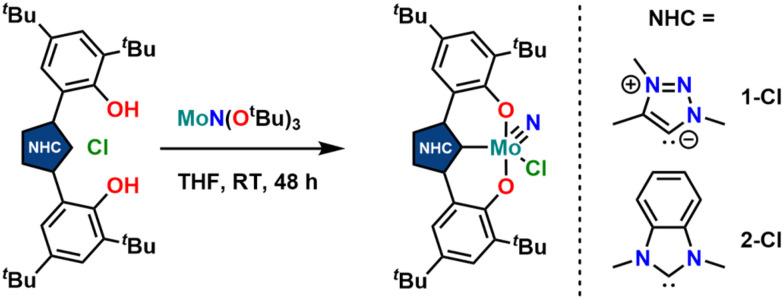
Synthesis of molybdenum nitride complexes featuring triazolylidene (1-Cl) and benzimidazolylidene ligands (2-Cl) *via* protonolysis between MoN(O^*t*^Bu)_3_ and the corresponding azolium salts [H_3_L^1^]Cl and [H_3_L^2^]Cl.

**Fig. 2:**
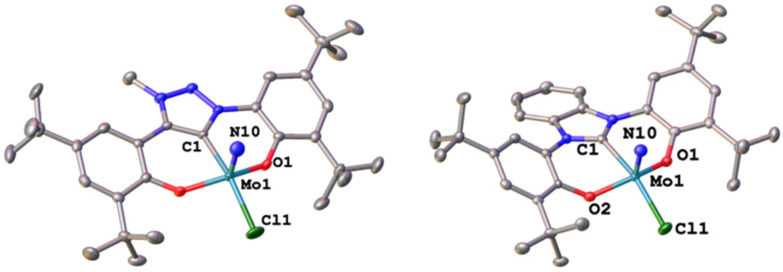
Molecular structures of the triazolylidene and benzimidazolylidene molybdenum nitride complexes 1-Cl and 2-Cl. Hydrogen atoms are omitted for clarity, and ellipsoids are shown at a probability level of 50%.

Synthesis of the triazolylidene and benzimidazolylidene complexes 1-Cl and 2-Cl was achieved following a protonolysis approach between the nitride precursor MoN(O^*t*^Bu)_3_ ^44^ and the corresponding azolium salts [H_3_L^1^]Cl ^29^ and [H_3_L^2^]Cl ^22,25^ in THF at room temperature (Scheme 1).^24,31,33,43^ After stirring the reaction mixture for 24 h, evaporation of the volatiles and washing the residue with hexanes, the target complexes 1-Cl and 2-Cl can be isolated as purple powders in yields of 86% and 83%, respectively. Successful formation of the carbene complexes is evident by the corresponding ^1^H and ^13^C NMR signatures. First, the absence of the benzimidazolium-2*H*/triazolium-5*H* signals in the low-field region in the corresponding ^1^H NMR spectra is an indicator for successful metalation (Fig. S1 and S6†). Furthermore, for complex 1-Cl, the shift of the triazolylidene-CH_3_ group from 3.30 ^29^ to 4.63 ppm is typical (Fig. S1†). Additionally, the ^13^C NMR spectra show resonances at 160.9 ppm for 1-Cl (Fig. S2†) and 191.0 ppm (Fig. S7†) for 2-Cl, which are typical for NHC/MIC complexes.^30^ The presence of the nitride ligand was indicated by IR spectroscopy, showing characteristic Mo–N stretching frequencies at 1035 cm^−1^ for complex 1-Cl and 1039 cm^−1^ for complex 2-Cl.^44^ Assignment of this band to the Mo–N stretching was confirmed by DFT calculations (see the ESI† for further information). The lower frequency obtained in complex 1-Cl compared to complex 2-Cl (1035 *vs.* 1039 cm^−1^) is in line with triazolylidenes being stronger donors compared to classical *n*NHC donors.^30,35,36,40–42^ Unambiguous proof was given by X-ray diffraction studies on single crystals grown from THF/hexane mixtures at −40 °C (Fig. 2). Both complexes crystallize in the orthorhombic system in the space group *Cmce* (1-Cl) and *Pbca* (2-Cl), with half a molecule of complex 1-Cl (with a pseudo-mirror plane going through the C1–Mo1–N10–Cl1 plane) and one molecule of complex 2-Cl in the asymmetric unit. The molybdenum carbene distances (M1–C1) are 2.145(4) Å for 1-Cl and 2.208(2) Å for complex 2-Cl. The shorter distance of the Mo1–C1 bond in the triazolylidene complex is in line with early examples of MIC complexes, showing shorter M–C distances compared to their nNHC analogs and reflects in the stronger donor character of MIC *vs.* the NHC donor.^30,35,36,40–42^ Despite the different donors (well reflected in the Mo–N stretching frequencies, *vide supra* and redox chemistry, *vide infra*) no difference in the Mo1–N10 bond length is visible at 1.642(4) Å and 1.647(2) Å in 1-Cl and 2-Cl. Furthermore, the yaw angle^45^ of the benzimidazolylidene complex 2-Cl at 15.55(1)° is much larger compared to that of the triazolylidene complex 1-Cl at 0.32(1)°. Similar yaw angles have also been seen in other benzimidazolylidene complexes of ligand L^2^ of vanadium, niobium or molybdenum.^30,32,33^

**Fig. 3:**
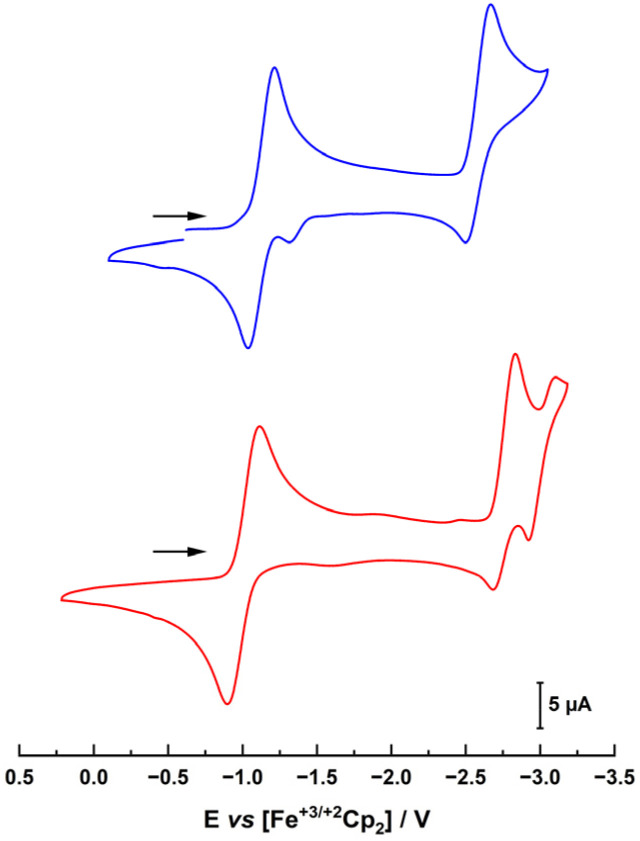
Cyclic voltammograms of complexes 1-Cl (blue trace, top) and 2-Cl (red trace, bottom) in THF with 0.15 M NBu_4_PF_6_ as a supporting electrolyte. Scan rate is shown at 100 mV s^−1^.

Next, the electrochemical properties of the complexes were investigated with special emphasis on their reduction chemistry. The cyclic voltammograms of 1-Cl and 2-Cl showed several (reversible) redox processes (Fig. 3). The first reduction appears at −1.13 and −1.01 V *vs.* Fc/Fc^+^ for 1-Cl and 2-Cl, respectively. Given the stronger donor character of the MIC in 1-Cl*vs*. the *n*NHC in 2-Cl, the reduction potential of 1-Cl is anodically shifted.^31^ This also explains why for the benzimidazoylidene complex 2-Cl, two further redox waves are present at −2.77 and −3.02 V, while for the triazolylidene complex **1-Cl**, the third reduction is shifted beyond the solvent window and only one reduction can be observed at −2.59 V.

**Scheme 2:**
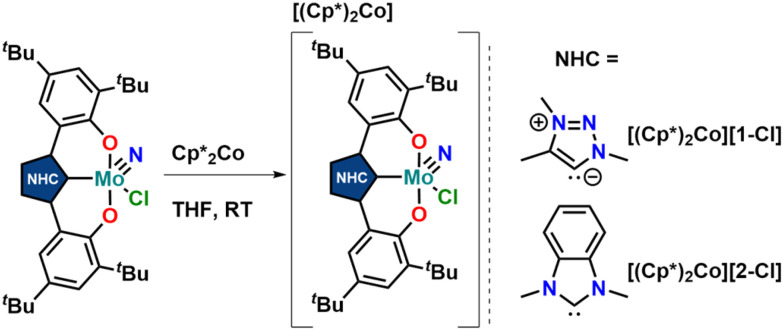
Synthesis of one-electron reduced complexes [1-Cl]^−^ and [2-Cl]^−^ using decamethylcobaltocene as a reductant.

**Fig. 4:**
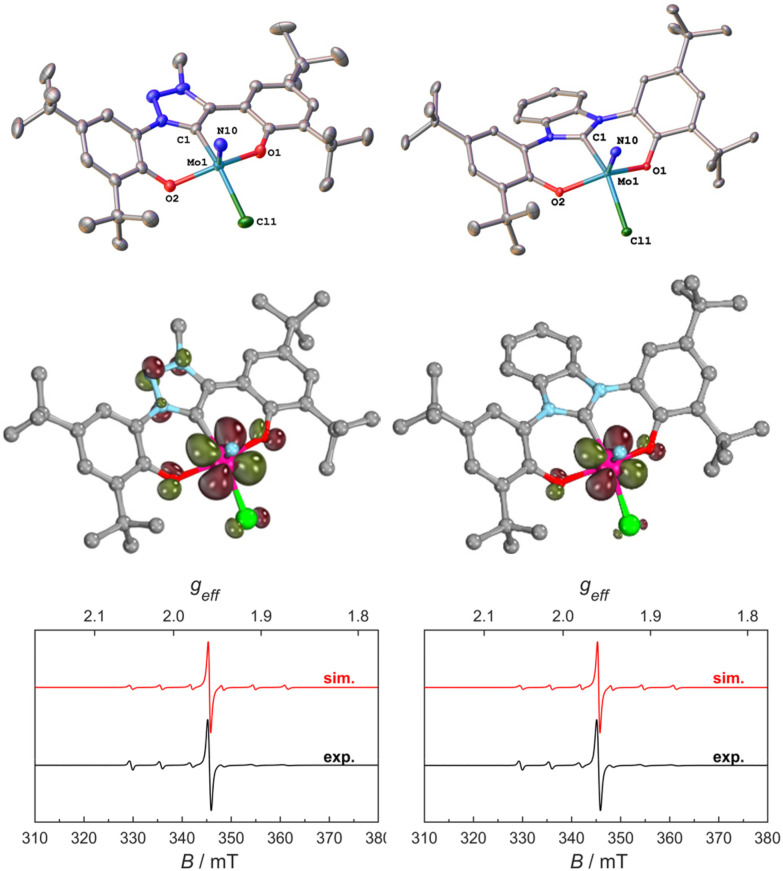
Molecular structures of the anionic Mo(v) complexes [1-Cl]^−^ and [2-Cl]^−^ (top). Hydrogen atoms, decamethyl cobaltocenium counter ions and solvent lattice molecules are omitted for clarity. Ellipsoids are shown at a probability level of 50%. Spin density plots of the two anionic complexes [1-Cl]^−^ and [2-Cl]^−^ showing the unpaired electrons residing in the 3d(*yz*) orbitals (QROs, middle). X-band EPR spectra of [1-Cl]^−^ and [2-Cl]^−^ of 5 mM solution in CH_2_Cl_2_ at 300 K; the black traces show the experimentally observed spectra and the red traces show the corresponding simulations (bottom).

Thus, we aimed for chemical reduction of the Mo(vi) chloride complexes 1-Cl and 2-Cl. Given the fact that the reduction of vanadium-oxo complexes with similar ligand scaffolds has worked well, using decamethylcobaltocene, we used this strategy to obtain Mo(v) complexes (Scheme 2). Mixing decamethylcobaltocene with 1-Cl or 2-Cl in dichloromethane at low temperature is accompanied by a fast colour change from purple to green, giving access to the Mo(v) NHC/MIC complexes [1-Cl]^−^ and [2-Cl]^−^ with a [(Cp*)_2_Co]^+^ counterion in yields of 62 and 75%, respectively.^31^ Due to the paramagnetic nature of these complexes, the ^1^H NMR spectra showed only broadened and unassignable features (Fig. S12 and S14†), but Evans’ method revealed a magnetic moment of 1.86 and 1.97*μ*_B_, respectively (Fig. S11 and S13†), which is in agreement with a d^1^-configured metal centre. The EPR spectra show the typical Mo(v) seven-line pattern at room temperature. Unambiguous proof for the successful reduction of the complexes 1-Cl and 2-Cl was given by X-ray diffraction studies performed on single crystals obtained by the slow evaporation of dichloromethane from a hexane solution of the complexes (Fig. 4 and S120, S121;† note: the reduced complexes [1-Cl]^−^ and [2-Cl]^−^ are entirely insoluble in hexane/aliphatic solvents but dissolve well if dichloromethane is added to these suspensions). The complexes crystalize in the orthorhombic space group *Pbca* for complex [1-Cl]^−^ and the monoclinic space group *Cc* for complex [2-Cl]^−^ with one molecule of dichloromethane in the asymmetric unit. The Mo–C1 distances shorten from 2.145(4) Å in 1-Cl to 2.115(3) Å in [1-Cl]^−^ and from 2.208(2) in 2-Cl to 2.173(3) Å in complex [2-Cl]^−^. Although this is counterintuitive to a metal-centred reduction process, the shortening of the Mo1–C1 bond could indicate some minor back-bonding effects.^31,33^ However, the shortening of the Mo–C bond could also be a result of ligand distortion caused by the elongated Mo–O bonds (*vide infra*), enforcing a shorter Mo–C distance of the central NHC/MIC core unit. The Mo1–O1/O2 distances increase from 1.9268(19)/1.9269(19) Å in 1-Cl to 2.0655(19)/2.0422(19) Å in [1-Cl]^−^ and from 1.9203(18)/1.9217(18) Å in 2-Cl to 2.0585(18)/2.0477(19) in [2-Cl]^−^. Similarly, the Mo1–Cl1 distances increases by about 0.1 Å upon reduction. The Mo1–N10 distance is mostly unaffected, showing a distance of 1.654(3) and 1.657(2) Å in [1-Cl]^−^ and [2-Cl]^−^, respectively.

**Scheme 3:**
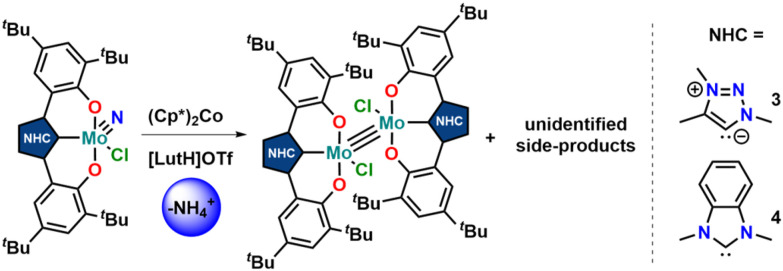
(Attempt) synthesis of molybdenum(iii) dimers 3 and 4 by reduction of the nitrido complexes 1-Cl and 2-Cl under protic conditions.

**Fig. 5:**
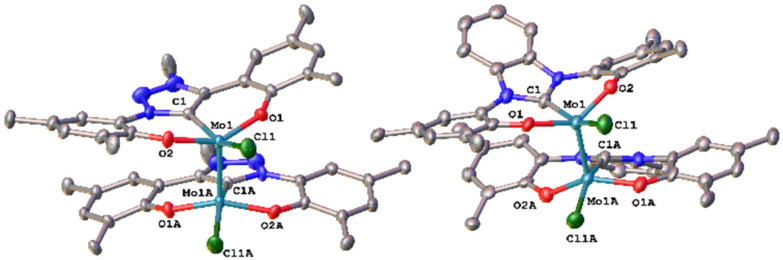
Molecular structures of the dimolybdenum(iii) complexes 3 and 4 with a Mo–Mo triple bond connecting the two molybdenum centers. Hydrogen atoms, lattice solvent molecules and ^*t*^Bu groups are omitted for clarity. Ellipsoids are shown at a probability level of 50%.

Unfortunately, using KC_8_ as a potential reductant fails on the chloride complexes and complicated mixtures are observed. This was also observed in vanadium complexes bearing the same ligands.^31,33^ Since molybdenum nitride complexes are potential precursors for homogeneous nitrogen fixation and functionalisation, we attempted the stoichiometric reduction of complexes 1-Cl and 2-Cl under protic conditions to see if ammonia could be evolved. Upon addition of five equivalents of lutidinium triflate and four equivalents of decamethylcobaltocene, we found the formation of ammonium after aqueous workup (Scheme 3). Quantification of the ammonium salts shows that in the case of complex 1-Cl, almost quantitative amounts of ammonium can be detected (96%, Fig. S17†), while in the case of 2-Cl, only 17% of ammonium could be isolated (Fig. S18†). Concerning the fate of the molybdenum complexes, we saw a moderately clean formation of a single species after recrystallisation from DCM/toluene for the triazolylidene complex **1-Cl** (Fig. 5 and Fig. S15,†*vide infra*) in a yield of approx. 20%, while in the benzimidazolylidene case, several (paramagnetic) products are observed (Fig. S16†). After work-up/crystallisation from dichloromethane/diethylether, we were able to isolate complex 4 in single crystalline yields (<2%). In the triazolylidene case, the yield of the Mo dimer species is notably higher in the crude mixture, but the low yield results from the loss of material during crystallisation. This is in line with the observed ammonium formation (*vide supra*). Both complexes 3 and 4 crystallize in the triclinic space group *P*1̄ with a partially occupied solvent molecule (toluene in the case of 3 and diethyl ether in the case of 4) and one complex molecule in the asymmetric unit (Fig. 5). Given the presence of one chlorido ligand, alongside the dianionic carbene ligands and the presence of a direct molybdenum–molybdenum interaction, we determined a +III oxidation state for the molybdenum centres. The Mo1–Mo1A distances of 2.2707(8) and 2.2726(10) Å in 3 and 4 are thereby in the range of previously reported (unsupported, non-bridged) Mo(iii)–Mo(iii) triple bonds.^46^ The Mo–Mo triple bond also explains the diamagnetic nature of the molecules, as observed by NMR spectroscopy (*vide infra*). Similar to the one-electron reduced complexes, the Mo1–C1 distances slightly decrease from 2.145(4) Å in 1-Cl and 2.208(2) in 2-Cl to 2.142(6) Å in 3 and to 2.177(7) Å in 4.^31,33^ The Mo1–O2/O2 distances remain largely unaffected by the reduction from Mo(vi) to Mo(iii), changing from 1.9268(19)/1.9269(19) Å in 1-Cl to 1.929(5)/1.929(5) Å in 3 and from 1.9203(18)/1.9217(18) Å in 2-Cl to 1.921(5)/1.918(5) Å in 4.

**Scheme 4:**
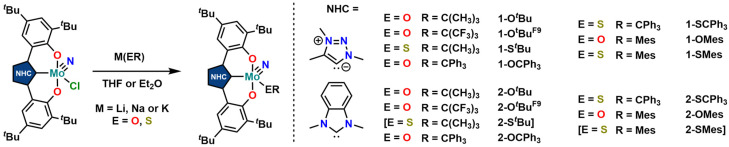
Synthesis of a large variety of functionalized molybdenum nitride complexes supported by triazolylidene and benzimidazolylidene ligands to further study the influence of carbene and the co-ligands on the redox variability of the complexes, entries written in parentheses (namely 2-S^*t*^Bu and 2-SMes) have been attempted, but no clean products have been observed; therefore, only limited (crystals for 2-SMes) or no (crystals for 2-S^*t*^Bu) characterisation data are reported.

Finally, we aimed for further derivatisation of 1-Cl and 2-Cl (Scheme 4) with two main targets: (I) identifying how the reduction potentials change in dependence of other co-ligands (*e.g.* alkoxides, thiolates or (thio-)phenolates) and (II) if dimerization reactions under protic reduction conditions are suppressed by more steric bulk, blocking potential deactivation pathways in catalysis. Both complexes 1-Cl and 2-Cl allow for a range of functionalisations. The reaction between the corresponding lithium, sodium or potassium alkoxides, thiolates or (thio-)phenolates gives clean access to the anticipated functionalized complexes. We were able to synthesize *tert*-butanolate complexes 1-O^*t*^Bu and 2-O^*t*^Bu, the corresponding nona-fluoro-*tert*-butanolate complexes 1-O^*t*^Bu^F9^ and 2-O^*t*^Bu^F9^, *tert*-butanthiolate complex 1-S^*t*^Bu, tritylate complexes 1-OCPh_3_ and 2-OCPh_3_, thiotritylate complexes 1-SCPh_3_ and 2-SCPh_3_, mesitolate complexes 1-OMes and 2-OMes as well as the thiomesitolate complex 1-SMes. While the yields for 1-S^*t*^Bu and 1-SMes are significantly lower than their alkoxide congeners, the *tert*-butanthiolate complex 2-S^*t*^Bu and the thiomesitolate complex 2-SMes could not be isolated in pure form. ^13^C NMR spectroscopy confirmed the presence of a C–Mo carbene interaction by showing the characteristic low-field signals at 165.3 ppm for 1-O^*t*^Bu, 196.7 ppm for 2-O^*t*^Bu, 163.4 ppm for 1-O^*t*^Bu^F9^, 193.2 ppm for 2-O^*t*^Bu^F9^, 160.3 ppm for 1-S^*t*^Bu, 164.7 ppm for 1-OCPh_3_, 197.6 ppm for 2-OCPh_3_, 161.1 ppm for 1-SCPh_3_, 193.6 ppm for 2-SCPh_3_, 165.9 ppm for 1-OMes, 197.1 ppm for 2-OMes and 161.8 ppm for 1-SMes. From these values, certain trends are observable: for all functionalized complexes, the ^13^C resonances seem to be shifted to lower fields, indicating a stronger donation compared to the chloride ligand. Furthermore, oxygen-based donor systems cause a more pronounced low-field shift compared to sulfur-based systems, which agrees with the donor strength further corroborated by X-ray diffraction experiments and cyclic voltammetry (*vide infra*).

**Fig. 6:**
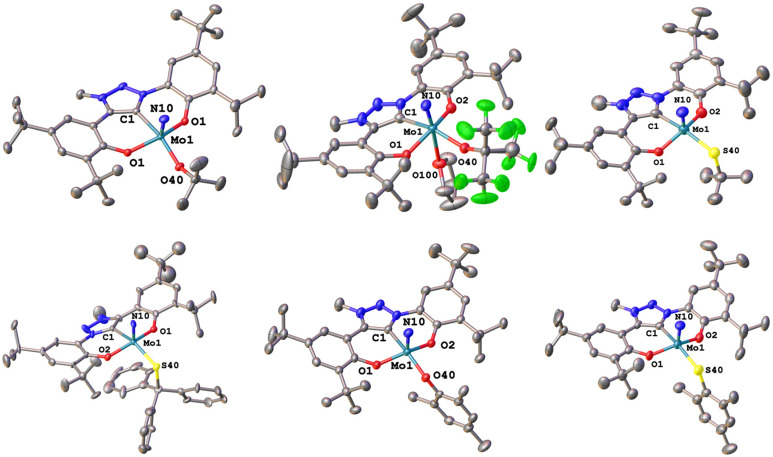
Molecular structures of the functionalized triazolylidene complexes 1-O^*t*^Bu, 1-O^*t*^Bu^F9^, and 1-S^*t*^Bu (top) and 1-OCPh_3_, 1-OMes, and 1-SMes (bottom). Hydrogen atoms and lattice solvent molecules are omitted for clarity. Ellipsoids are shown at a probability level of 50%.

**Fig. 7:**
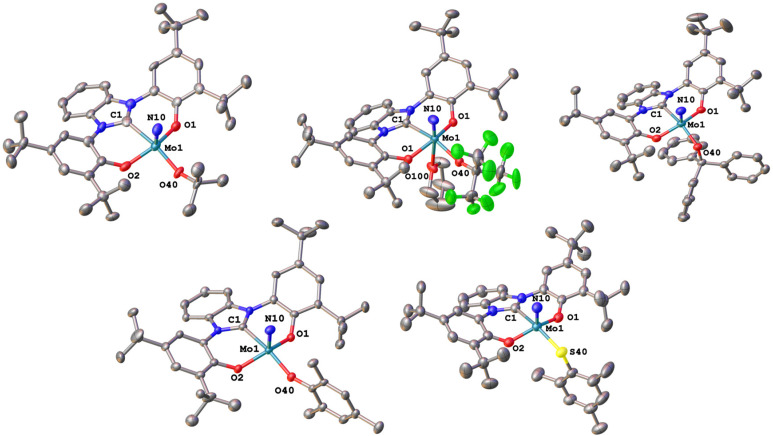
Molecular structures of the functionalized triazolylidene complexes 2-O^*t*^Bu, 2-O^*t*^Bu^F9^, and 2-OCPh_3_ (top) and 2-OMes, and 2-SMes (bottom). Hydrogen atoms and lattice solvent molecules are omitted for clarity. Ellipsoids are shown at a probability level of 50%.

We were able to grow single crystals for all complexes, except for 2-S^*t*^Bu, 1-OCPh_3_ and 2-SCPh_3_ (Fig. 6 and 7). Given the structural similarity of all complexes, only the metal–carbene and metal–ligand bonds will be discussed in the following and further bond metrics can be found in the ESI (Tables S1–S3†). In all complexes, the additional donor ligand is situated in a *trans*-position to the carbene center,^31–33^ which elucidates its large influence on the ^13^C NMR carbene resonances described above (*vide supra*). Focussing on the triazolylidene complexes, the metal ligand distances (Mo1–O40 and Mo1–S40) are 1.8987(17) Å in 1-O^*t*^Bu, 1.997(2) Å in 1-O^*t*^Bu^F9^, 2.360(2) Å in 1-S^*t*^Bu, 2.369(3) Å in 1-SCPh_3_, 1.9378(17) Å in 1-OMes and 2.3767(17) Å in 1-SMes. The longer distances of the Mo–S bonds compared to those of the Mo–O bonds are in line with the size of the donor atoms. The perfluorinated *tert*-butanolate ligand in 1-O^*t*^Bu^F9^ shows an about 0.1 Å longer Mo1–O40 distance (1.997(2) Å), compared to the non-fluorinated system 1-O^*t*^Bu (1.8987(17) Å), while this distance in the arylated complex 1-OMes lies in between these two values (1.9378(17) Å), in line with the donor strength of the three alkoxide/phenolate ligands (O^*t*^Bu > OMes > O^*t*^Bu^F9^). This influence is also reflected in their electrochemical properties (*vide infra*). Interestingly, the Mo–S–C angles also seem to be more bent compared to the Mo–O–C angles. While for the complexes 1-O^*t*^Bu, 1-O^*t*^Bu^F9^ and 1-OMes, Mo1–O40–C40 angles of 141.87°, 152.0(2)° and 138.89(17)° are observed, and the Mo1–S40–C40 angles are 112.2(3)°, 107.5(3)° and 118.1(2)° in 1-S^*t*^Bu, 1-SCPh_3_ and 1-SMes. This effect is also well reflected in the line widths of the ligand groups in ^1^H-NMR, indicating significantly different rotational barriers. While 1-S^*t*^Bu displays a much broader *tert*-butyl signal than 1-O^*t*^Bu (Fig. S19 and S41†), 1-SMes shows notably sharper signals than 1-OMes (Fig. S66 and S76†). The molybdenum–triazolylidene carbon interaction remains largely unaffected by the donor variations, displaying values between 2.154(3) and 2.188(2) Å with 1-O^*t*^Bu showing the longest and 1-OMes showing the shortest metal carbene distance. For the benzimidazolylidene congeners, the above-mentioned trends are reproduced with the only difference that the Mo1–C1 distances are slightly longer, ranging from 2.201(4) Å in 2-OMes to 2.256(11) Å in 2-O^*t*^Bu.^28,30^ This is in line with the stronger donor character of triazolylidenes compared to benzimidazolylidenes, leading to stronger metal–carbene interactions.^40–42^ Further information on the structural parameters of the functionalized complexes can be found in the ESI, Tables S1–S3.†

**Fig. 8:**
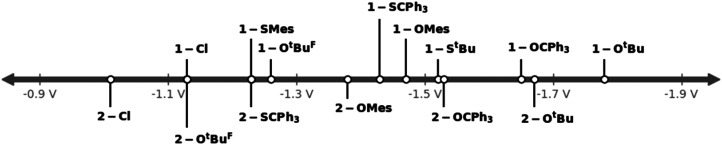
Graphical comparison of the Mo(vi)/Mo(v) reduction potentials of the complex series 1-L (top) and 2-L (bottom). (L = Cl, O^*t*^Bu, O^*t*^Bu^F9^, S^*t*^Bu, OCPh_3_, SCPh_3_, OMes and SMes.)

Next, we turned to the investigation of their electrochemical properties in comparison to the halide complexes 1-Cl and 2-Cl (Table 1, Fig. 8). As expected, the exchange of the halide with chalcogenide ligands shifts the reduction potential anodically. As such, the *tert*-butanolate complexes 1-O^*t*^Bu and 2-O^*t*^Bu show pronounced shifts from −1.13 V and −1.01 V in 1-Cl and 2-Cl to −1.78 V and −1.67 V *vs.* Fc/[Fc]^+^. Similar to the halide complexes, the triazolylidene-supported system has a potential approx. 0.1 V more negative than the benzimidazolylidene-supported system. Perfluorination of the *tert*-butanolate ligand in 1-O^*t*^Bu^F9^ and 2-O^*t*^Bu^F9^ results in the potential shifting cathodically to −1.26 V and −1.13 V compared to the non-fluorinated *tert*-butanolate complexes 1-O^*t*^Bu and 2-O^*t*^Bu. Replacing *tert*-butanolate with tritylate residues gives redox potentials of −1.65 V in 1-OCPh_3_ and −1.53 V in 2-OCPh_3_. Switching from alkoxide to phenolate, *e.g.* mesitolate changes the redox potentials to −1.47 V and 1.38 V in 1-OMes and 2-OMes. Another way to tune the redox potential is the exchange of oxygen for sulfur-based ligands. Compared to their oxygen congeners, the sulfur-based systems shift the redox potentials less anodically, taking 1-Cl and 2-Cl as reference points. For example, we found the Mo(vi)/Mo(v) reduction at −1.78 V *vs.* Fc/[Fc]^+^ for 1-O^*t*^Bu while for the sulfur congener, 1-S^*t*^Bu, the reduction appears at −1.52 V *vs.* Fc/[Fc]^+^. Similarly, the reduction potentials for 1-SCPh_3_ and 1-SMes are found at −1.43 V and −1.23 V *vs.* Fc/[Fc]^+^ (compare 1.65 V for 1-OCPh_3_ and −1.47 V for 1-OMes). These trends are also observed in the benzimidazolylidene complex 2-SCPh_3_*vs.*2-OCPh_3_ (see Table 1 for comparison).

**Table 1:** Electrochemical potentials determined by cyclic voltammetry measured in 0.15 M NBu_4_PF_6_ solution in THF using a three-electrode setup (working electrode: glassy carbon; pseudo reference electrode: silver wire; counter electrode: platinum wire). All values are referenced *vs.* Fc/[Fc]^+^. The complexes are ordered by the type (1-L*vs.*2-L) and increasing reduction potentials of *E*_1,red_

Compound	*E*_1,red_	*E*_2,red_	*E*_3,red_
1-Cl	−1.13	−2.59	n.o.^a^
1-SMes	−1.23	−2.82^b^	n.o.^a^
1-O^*t*^Bu^F9^	−1.26	−2.71^b^	n.o.^a^
1-SCPh_3_	−1.43	−2.76^b^	−3.12^b^
1-OMes	−1.47	−3.02^b^	n.o.^a^
1-S^*t*^Bu	−1.52	−2.77^b^	n.o.^a^
1-OCPh_3_	−1.65	−2.93^b^	n.o.^a^
1-O^*t*^Bu	−1.78	−2.82^b^	n.o.^a^

2-Cl	−1.01	−2.77	−3.02
2-O^*t*^Bu^F9^	−1.13	−2.89	n.o.^a^
2-SCPh_3_	−1.23	n.o.^a^	n.o.^a^
2-OMes	−1.38	−2.96	n.o.^a^
2-OCPh_3_	−1.53	−2.90	n.o.^a^
2-O^*t*^Bu	−1.67	−2.91	n.o.^a^

a n.o. = not observed.

b Irreversible process, value is given as *E*_pc_.

Given the fact that the first reduction is metal centred in all complexes 1-L and 2-L, the different redox potentials observed can be directly correlated to the donor strength of the corresponding co-ligands and allow us to sort their donor properties in the following order: O^*t*^Bu > OCPh_3_ > S^*t*^Bu > OMes ≈ SCPh_3_ > O^*t*^Bu^F9^ ≈ SMes (compare also Table 1 and Fig. 8).^47^ Since efficient nitrogen reduction chemistry is a complex interplay between sterics and reduction potentials (more positive reduction potentials are favoured as they allow the use of “weaker” reductants), this series suggests thiomesitolate, as well as the perfluorinated *tert*-butanolate ligands to be promising candidates for reductive nitrogen functionalization.

Schrock as well as Hu and co-workers have already successfully demonstrated that the use of *tert*-butanolate ligands has advantageous effects on catalysis, leading to higher TONs and efficiencies.^43,48^

## Conclusions

In summary, we have presented the synthesis of sixteen new molybdenum nitrido complexes 1-L and 2-L as well as two new dimeric molybenium(iii) complexes 3 and 4, with a direct molybdenum(iii)–molybdenum(iii) triple bond supported by NHC and MIC bis-phenolate ligands. While the former sixteen complexes represent potential catalysts for the catalytic functionalization of dinitrogen, the latter two show potential catalyst deactivation routes, which need to be suppressed. Addressing this issue, we identified the thiomesitolate complexes 1-Mes and 2-Mes as well as the nonafluoro-*tert*-butanolate complexes 1-O^*t*^Bu^F9^ and 2-O^*t*^Bu^F9^ to be promising candidates, providing low reduction potentials, while offering enough steric bulk to prevent the dimerization reactions.

## Experimental section

### General considerations

Unless stated otherwise, all operations were performed in an argon filled glovebox (H_2_O and O_2_ < 1 ppm) or using high-vacuum standard Schlenk techniques under an argon atmosphere. Solvents were dried using an MBraun SPS and stored over 3 Å molecular sieves at least 3 days prior to use. THF was distilled over sodium/benzophenone and stored over 3 Å molecular sieves. CD_2_Cl_2_ and C_6_D_6_ were degassed with an argon stream and stored over 3 Å molecular sieves for at least three days prior to use. The metal precursor MoN(O^*t*^Bu)_3_ ^44^ and proligands [H_3_L^1^]Cl ^29^ and [H_3_L^2^]Cl ^22,25^ were synthesized according to literature-known procedures. Lithium and potassium salts were obtained by deprotonation of the alcohol or thiol in hexane with *n*-BuLi or KHDMS, respectively. All other reagents were used as received from commercial sources. NMR spectra were recorded on a 400 MHz Bruker Avance 4 Neo spectrometer. ^1^H and ^13^C NMR chemical shifts (*δ*) are reported in ppm and were calibrated to residual solvent peaks. IR spectra were recorded on a Bruker Alpha spectrometer using the ATR method. UV/Vis/NIR spectra were recorded on an Avantes spectrometer using deuterium and halogen light sources and a CMOS detector. Elemental analysis was performed using an Elementar Vario Micro cube instrument. Single-crystal X-ray diffraction was performed on a Bruker D8 Quest diffractometer and data were collected using the ApexIV software package. Structures were solved using SHELXT^49^ and refined using the OLEX 2 software package.^50^ All non-hydrogen atoms were refined anisotropically, and hydrogen atoms were included at the geometrically calculated positions and refined using a riding model using SHELXL.^51^ For heavily disordered solvent molecules, the SQUEEZE algorithm was applied.^52^ EPR measurements were performed on a Magnettech MS5000 X-band spectrometer equipped with a temperature control unit in 3 mm o.d. J-Young style fused silica tubes. Spectra were processed using the EasySpin package for Matlab®.^53^ Cyclic voltammograms were recorded with a Gamry Interface 1010B potentiostat using a three-electrode setup (a glass carbon WE, an Ag wire RE, and a Pt wire CE) in 0.15 M NBu_4_PF_6_ solution in THF. Potentials are referenced to the Fc/Fc^+^ couple.

### General procedure for the synthesis of complexes 1 and 2

The molybdenum precursor MoN(O^*t*^Bu)_3_ and the corresponding proligand were mixed in a 100 mL Schlenk flask and 50 mL THF was added. The reaction mixture was stirred for 24 hours, during which the color changed to a deep purple. The solvent was removed *in vacuo*, hexane (20 mL) was added and the resulting solution was stirred for two hours. The product precipitated as dark purple powder was filtered off, washed with hexane and dried under high vacuum. When thoroughly dried, both complexes are pale purple.

#### L^1^MoNCl (1-Cl)

From MoN(O^*t*^Bu)_3_ (3.00 g, 5.69 mmol, 1 equiv.) and [H_3_L^1^]Cl (1.87 g, 5.68 mmol, 1 equiv.), 3.45 g (4.88 mmol, 86%) of a pale purple powder was obtained after lyophilization from benzene. Single crystals were obtained from slow evaporation of a concentrated solution of dichloromethane at room temperature. ^1^H NMR (400 MHz, CD_2_Cl_2_) *δ* 8.07 (d, *J* = 2.4 Hz, 1H), 7.75 (d, *J* = 2.4 Hz, 1H), 7.65 (d, *J* = 2.4 Hz, 1H), 7.58 (d, *J* = 2.4 Hz, 1H), 4.63 (s, 3H), 1.52 (s, 9H), 1.52 (s, 9H), 1.42 (s, 9H), 1.41 (s, 9H). ^13^C NMR (101 MHz, CD_2_Cl_2_) *δ* 161.0, 160.0, 152.8, 143.8, 143.4, 141.5, 140.9, 139.5, 127.5, 126.3, 122.6, 119.3, 114.3, 111.5, 41.0, 36.2, 35.0, 31.7, 31.6, 30.0, 29.9. UV/VIS/NIR: *λ*_max_ = 327 (*ε* = 20 430 L mol^−1^ cm^−1^), 352 (*ε* = 20 570 L mol^−1^ cm^−1^), 511 (*ε* = 2410 L mol^−1^ cm^−1^). IR (cm^−1^): 2955, 2906, 2869, 1605, 1533, 1484, 1466, 1431, 1413, 1388, 1362, 1303, 1245, 1211, 1153, 1127, 1088, 1047, 1035, 970, 921, 876, 849, 800, 774, 759, 723, 712, 692, 643, 559, 480, 453, 416. Elemental analysis (%) calc'd for C_31_H_43_ClMoN_4_O_2_·0.25CH_2_Cl_2_: C 57.19, H 6.68, N 8.54; found: C 56.6, H 6.74, N 8.18.

#### L^2^MoNCl (2-Cl)

From MoN(O^*t*^Bu)_3_ (1.80 g, 5.47 mmol, 1 equiv.) and [H_3_L^2^]Cl (3.08 g, 5.47 mmol, 1 equiv.), 3.05 g (4.55 mmol, 83%) of a pale purple powder was obtained. Single crystals were obtained from a concentrated solution of dichloromethane at room temperature. ^1^H NMR (400 MHz, CD_2_Cl_2_) *δ* 8.18 (dd, *J* = 6.4, 3.3 Hz, 2H), 7.86 (d, *J* = 2.3 Hz, 2H), 7.64 (dd, *J* = 6.4, 3.3 Hz, 2H), 7.55 (d, *J* = 2.3 Hz, 2H), 1.56 (s, 18H), 1.44 (s, 18H). ^13^C NMR (101 MHz, CD_2_Cl_2_) *δ* 191.0, 153.7, 144.1, 139.8, 134.3, 126.3, 123.9, 123.8, 116.5, 114.7, 36.1, 35.2, 31.7, 30.1. UV/VIS/NIR: *λ*_max_ = 326 (*ε* = 40 710 L mol^−1^ cm^−1^), 484 (*ε* = 4580 L mol^−1^ cm^−1^). IR (cm^−1^): 2961, 2906, 2869, 1580, 1482, 1468, 1431, 1386, 1362, 1333, 1290, 1254, 1239, 1200, 1162, 1121, 1070, 1039, 988, 921, 880, 857, 810, 800, 759, 747, 696, 639, 612, 567, 541, 486, 478, 449, 439. Elemental analysis (%) calc'd for C_35_H_44_ClMoN_3_O_2_: C 62.73, H 6.62, N 6.27; found: C 62.75, H 6.72, N 6.02.

### General procedure for the one-electron reduction of complexes 1-Cl and 2-Cl

The corresponding Mo^VI^ complex 1-Cl or 2-Cl was dissolved in THF (5 mL) in a 20 mL scintillation vial and cooled to −40 °C. Solid (Cp*)_2_Co was added in small portions under stirring during which the color of the reaction changed to a dark brownish-green. The reaction mixture was allowed to warm to room temperature and after two hours, the solvent was removed *in vacuo*. The product was purified by crystallization from dichloromethane.

#### [(Cp*)_2_Co][L^1^MoNCl] ([1-Cl]^−^)

From 1-Cl (75.0 mg, 0.118 mmol, 1 equiv.) and (Cp*)_2_Co (42.8 mg, 0.130 mmol, 1.1 equiv.), a pale green powder was obtained. Single crystals suitable for X-ray diffraction were obtained from a concentrated solution in dichloromethane at room temperature. Yield: 71 mg (0.073 mmol, 62%). Evans method (CD_2_Cl_2_, 293 K): *μ*_eff_ = 1.86*μ*_B_. X-band EPR (CD_2_Cl_2_, 293 K): *g*_iso_ = 1.958 and *a*_iso_ = 172 MHz.

#### [(Cp*)_2_Co][L^2^MoNCl] ([2-Cl]^−^)

From 2-Cl (75.0 mg, 0.112 mmol, 1 equiv.) and (Cp*)_2_Co (40.5 mg, 0.123 mmol, 1.1 equiv.), a pale green to light brown powder was obtained. Single crystals suitable for X-ray diffraction were obtained from a concentrated solution in dichloromethane at room temperature. Yield 84 mg (0.084 mmol, 75%). Evans method (CD_2_Cl_2_, 293 K): *μ*_eff_ = 1.97*μ*_B_. X-band EPR (CD_2_Cl_2_, 293 K): *g*_iso_ = 1.958 and *a*_iso_ = 170 MHz.

### General procedure for the (attempted) synthesis of dimers 3 and 4

The corresponding Mo^VI^ complex 1-Cl or 2-Cl was dissolved in THF (5 mL) in a 20 mL scintillation vial and cooled to −40 °C. In two separate scintillation vials, (Cp*)_2_Co and [LutH][OTf] were dissolved in 2 mL THF each. Keeping the reaction mixture at −40 °C, solutions with the reactants were simultaneously added dropwise to the complex upon which the mixture turned black. After the addition was complete, the reaction mixture was allowed to warm to room temperature and stirred for 16 hours. Yellow [(Cp*)_2_Co][OTf] was filtered off and the solvent was removed *in vacuo*.

#### [L^1^MoCl]_2_ (3)

From 1-Cl (100 mg, 0.158 mmol, 1 equiv.), [LutH][OTf] (130 mg, 0.504 mmol, 3.2 equiv.) and Cp*_2_Co (161 mg, 0.488 mmol, 3.1 equiv.), a dark brown solid with yellow [Cp*_2_Co][OTf] impurities was obtained. Crystals suitable for X-ray diffraction were obtained by evaporation of a mixture of dichloromethane and toluene. ^1^H NMR (400 MHz, CD_2_Cl_2_) *δ* 7.73 (s, 2H), 7.72 (s, 2H), 7.54 (s, 2H), 7.52 (s, 2H), 7.49 (s, 2H), 7.47 (s, 2H), 7.43 (s, 2H), 7.38 (s, 2H), 4.35 (s, 3H), 4.29 (s, 3H), 1.73 (s, [Cp*_2_Co][OTf]), 1.56 (s, 9H), 1.55 (s, 9H), 1.49 (s, 9H), 1.49 (s, 9H), 1.45 (s, 9H), 1.33 (s, 9H), 1.29 (s, 9H), 1.10 (s, 9H).

#### [L^2^MoCl]_2_ (4)

From 2-Cl (75 mg, 0.112 mmol, 1 equiv.), [LutH][OTf] (92 mg, 0.358 mmol, 3.2 equiv.) and Cp*_2_Co (114 mg, 0.347 mmol, 3.1 equiv.), crystals suitable for X-ray diffraction were obtained by evaporation of a mixture of dichloromethane and diethyl ether.

### General procedure for the ammonia quantification experiment

Inside an argon filled glovebox, the respective complex (1 equiv.) and [LutH][OTf] (5 equiv.) were placed in a Schlenk flask and dissolved in 2 mL dry THF. A dropping funnel was attached and charged with a THF solution (approx. 10 mL) of Cp*_2_Co (4 equiv.). The apparatus was removed from the glovebox and the flask was cooled to −78 °C. The Cp*_2_Co solution was added dropwise over a period of 15 minutes and the apparatus was warmed to room temperature over the course of 2 hours. 2 mL of HCl in diethyl ether was added quickly through a dropping funnel and the mixture was stirred for an additional one hour. All subsequent steps were performed under air. The reaction mixture was filtered to remove yellow decamethyl cobaltocenium triflate and the solvent was removed *in vacuo*. The residue was suspended in dichloromethane, filtered and washed thoroughly with DCM. The remaining solid was extracted with water, evaporated to dryness and 17.7 mg (0.020 mmol) NaBArF_24_ was added. NMR spectra were recorded in DMSO-d_6_ and the yield was calculated relative to the NaBArF_24_ standard.

For 1-Cl: From 25.0 mg (0.039 mmol, 1 equiv.) 1-Cl, 50.6 mg (0.197 mmol, 5 equiv.) [LutH][OTf] and 51.9 mg (0.157 mmol, 4 equiv.) Cp*_2_Co. Isolated yield of NH_4_^+^: 96% (0.038 mmol).

For 2-Cl: From 25.0 mg (0.037 mmol, 1 equiv.) 2-Cl, 48.0 mg (0.187 mmol, 5 equiv.) [LutH][OTf] and 49.2 mg (0.149 mmol, 4 equiv.) Cp*_2_Co. Isolated yield of NH_4_^+^: 17% (0.0065 mmol).

### General procedure for the synthesis of salt metathesis products 1-ER and 2-ER

The respective chloride complex 1-Cl or 2-Cl was dissolved in THF (5 mL) in a 20 mL scintillation vial and cooled to −40 °C. A solution of the alkoxide or thiolate in THF (5 mL) was added dropwise at −40 °C, followed by slow warming to room temperature. The reaction mixture was stirred for 16 hours during which the color slowly changed from deep purple to between bright yellow and dark red, depending on the reactant. The solvent was removed *in vacuo* and the residue was dissolved in 5 mL of hexane. The complexes bearing OC(CF_3_)_3_, OCPh_3_ and SCPh_3_ ligands are barely soluble in hexane, therefore 1 mL diethyl ether or dichloromethane was added. The resulting suspension was filtered and the solution was concentrated *in vacuo*. Unless stated otherwise, an analytically pure product was precipitated upon cooling to −40 °C which was filtered off and washed with a minimal amount of cold hexane. The washed product was concentrated and the process was repeated for a total of three times.

#### L^1^MoN(O^*t*^Bu) (1-O^*t*^Bu)

From 1-Cl (150 mg, 0.236 mmol, 1 equiv.) and NaO^*t*^Bu (22.7 mg, 0.236 mmol, 1 equiv.), 124 mg (0.184 mmol, 78%) of a bright yellow powder was obtained. Single crystals suitable for X-ray diffraction were obtained from a concentrated solution in hexane at −40 °C. ^1^H NMR (400 MHz, C_6_D_6_) *δ* 8.16 (d, *J* = 2.5 Hz, 1H), 7.72 (d, *J* = 2.4 Hz, 1H), 7.71 (d, *J* = 2.5 Hz, 1H), 7.08 (d, *J* = 2.4 Hz, 1H), 3.15 (s, 3H), 2.04 (s, 9H), 1.78 (s, 9H), 1.77 (s, 9H), 1.44 (s, 9H), 1.39 (s, 9H). ^13^C NMR (101 MHz, C_6_D_6_) *δ* 165.3, 160.5, 153.7, 142.6, 142.0, 140.9, 140.4, 140.0, 126.0, 125.3, 125.0, 119.3, 114.6, 113.8, 80.6, 38.7, 36.6, 36.4, 34.8, 34.6, 32.8, 31.9, 31.8, 30.5, 30.3. UV/VIS/NIR: *λ*_max_ = 327 (*ε* = 22 840 L mol^−1^ cm^−1^), 362 (*ε* = 25 170 L mol^−1^ cm^−1^), 368 (*ε* = 25 230 L mol^−1^ cm^−1^). IR (cm^−1^): 2957, 2903, 2867, 1533, 1480, 1429, 1417, 1386, 1358, 1294, 1258, 1237, 1202, 1174, 1129, 1090, 1074, 1060, 1019, 955, 874, 849, 802, 780, 757, 717, 692, 643, 572, 547, 492, 476, 445. Elemental analysis (%) calc'd for C_35_H_52_MoN_4_O_3_: C 62.49, H 7.79, N 8.33; found: C 62.35, H 7.66, N 8.15.

#### L^2^MoN(O^*t*^Bu) (2-O*t*Bu)

From 2-Cl (250 mg, 0.373 mmol, 1 equiv.) and NaO^*t*^Bu (36 mg, 0.373 mmol, 1 equiv.), 178 mg (0.251 mmol, 67%) of a bright yellow powder was obtained. Single crystals suitable for X-ray diffraction were obtained from a concentrated solution in hexane at −40 °C. ^1^H NMR (400 MHz, C_6_D_6_) *δ* 7.85 (m, 2H), 7.68 (d, *J* = 2.4 Hz, 2H), 7.64 (d, *J* = 2.4 Hz, 2H), 6.97 (m, 2H), 1.94 (s, 9H), 1.79 (s, 18H), 1.39 (s, 18H). ^13^C NMR (101 MHz, C_6_D_6_) *δ* 196.8, 154.0, 141.0, 140.7, 134.5, 128.6, 125.7, 124.5, 123.0, 116.7, 114.0, 81.8, 36.4, 34.8, 32.0, 31.8, 30.5. UV/VIS/NIR: *λ*_max_ = 325 (*ε* = 76 630 L mol^−1^ cm^−1^). IR (cm^−1^): 2950, 2903, 2867, 1572, 1482, 1466, 1431, 1382, 1358, 1333, 1292, 1270, 1256, 1229, 1202, 1176, 1055, 1041, 1025, 960, 921, 878, 855, 800, 786, 766, 745, 690, 637, 614, 594, 557, 492, 445. Elemental analysis (%) calc'd for C_39_H_53_MoN_3_O_3_: C 66.18, H 7.55, N 5.94; found: C 66.00, H 7.46, N 5.86.

#### L^1^MoN(O^*t*^Bu^F9^) (1-O^*t*^Bu^F9^)

From 1-Cl (150 mg, 0.236 mmol, 1 equiv.) and KO^*t*^Bu^F9^ (65 mg, 0.236 mmol, 1 equiv.), 154 mg (0.184 mmol, 78%) of a pale red powder was obtained. Single crystals suitable for X-ray diffraction were obtained from a concentrated solution in hexane/diethylether at −40 °C. ^1^H NMR (400 MHz, C_6_D_6_) *δ* 8.08 (d, *J* = 2.4 Hz, 1H), 7.75 (d, *J* = 2.4 Hz, 1H), 7.74 (d, *J* = 2.4 Hz, 1H), 7.03 (d, *J* = 2.4 Hz, 1H), 3.18 (s, 3H), 1.75 (s, 9H), 1.74 (s, 9H), 1.39 (s, 12H), 1.35 (s, 9H). ^13^C NMR (101 MHz, C_6_D_6_) *δ* 163.4, 160.1, 153.2, 142.8, 142.3, 141.9, 140.8, 126.9, 126.1, 123.8, 121.0, 119.1, 114.4, 112.6, 39.0, 36.5, 36.3, 34.8, 34.6, 31.7, 31.6, 30.2, 30.1. ^19^F NMR (377 MHz, C_6_D_6_) *δ* −71.67. UV/VIS/NIR: *λ*_max_ = 315 (*ε* = 21 010 L mol^−1^ cm^−1^), 499 (*ε* = 790 L mol^−1^ cm^−1^). IR (cm^−1^): 2959, 2908, 2871, 1776, 1535, 1482, 1433, 1390, 1362, 1294, 1262, 1239, 1178, 1131, 1080, 1055, 1035, 970, 921, 878, 847, 802, 774, 757, 727, 694, 643, 553, 492, 476, 447. Due to the fluorine present in the sample, no reproducible and reliable elemental analysis could be achieved.

#### L^2^MoN(O^*t*^Bu^F9^) (2-O^*t*^Bu^F9^)

From 2-Cl (75 mg, 0.112 mmol, 1 equiv.) and KO^*t*^Bu^F9^ (31 mg, 0.112 mmol, 1 equiv.), 67 mg (0.770 mmol, 69%) of a pale pink powder was obtained. Single crystals suitable for X-ray diffraction were obtained from a concentrated solution in hexane/diethylether at −40 °C. ^1^H NMR (400 MHz, C_6_D_6_) *δ* 7.78 (m, 2H), 7.65 (d, *J* = 2.4 Hz, 2H), 7.59 (d, *J* = 2.4 Hz, 2H), 6.95 (m, 2H), 1.76 (s, 18H), 1.33 (s, 19H). ^13^C NMR (101 MHz, C_6_D_6_) *δ* 193.2, 153.8, 142.9, 141.1, 134.3, 125.2, 124.4, 123.7, 116.2, 114.3, 36.4, 34.8, 31.6, 30.3. ^19^F NMR (377 MHz, C_6_D_6_) *δ* −71.62. *λ*_max_ = 325 (*ε* = 42 210 L mol^−1^ cm^−1^), 464 (*ε* = 3970 L mol^−1^ cm^−1^). IR (cm^−1^): 2955, 2908, 2871, 1480, 1431, 1384, 1360, 1337, 1323, 1264, 1243, 1180, 1117, 1070, 1051, 1039, 1023, 970, 921, 892, 876, 855, 810, 782, 761, 727, 690, 647, 637, 610, 553, 537, 486, 445. Due to the fluorine present in the sample, no reproducible and reliable elemental analysis could be achieved.

#### L^1^MoN(S^*t*^Bu) (1-S^*t*^Bu)

From 1-Cl (150 mg, 0.236 mmol, 1 equiv.) and LiS^*t*^Bu (27 mg, 0.283 mmol, 1.2 equiv.), 35 mg (0.051 mmol, 22%) of a bright orange powder was obtained. Single crystals suitable for X-ray diffraction were obtained from a concentrated solution of in hexane at −40 °C. ^1^H NMR (400 MHz, CD_2_Cl_2_) *δ* 8.00 (d, *J* = 2.5 Hz, 1H), 7.67 (d, *J* = 2.4 Hz, 1H), 7.56 (d, *J* = 2.4 Hz, 1H), 7.50 (d, *J* = 2.5 Hz, 1H), 4.59 (s, 3H), 1.62 (s, 9H), 1.49 (s, 9H), 1.48 (s, 9H), 1.40 (s, 9H), 1.39 (s, 9H). ^13^C NMR (101 MHz, CD_2_Cl_2_) *δ* 160.3, 142.9, 142.0, 141.7, 141.6, 140.4, 126.9, 125.7, 119.4, 114.5, 112.7, 51.4, 40.7, 36.2, 36.1, 35.2, 34.9, 31.7, 31.7, 30.4, 30.3. UV/VIS/NIR: *λ*_max_ = 388 (*ε* = 59 200 L mol^−1^ cm^−1^). IR (cm^−1^): 2955, 2903, 2865, 1517, 1480, 1445, 1429, 1415, 1392, 1360, 1296, 1256, 1200, 1153, 1131, 1078, 1051, 1031, 921, 874, 853, 815, 798, 768, 757, 714, 692, 637, 576, 545, 502, 461, 449, 431, 408. Elemental analysis (%) calc'd for C_35_H_52_MoN_4_O_2_S·C_6_H_6_: C 64.21, H 7.62, N 7.31; found: C 64.60, H 7.45, N 6.90.

#### L^1^MoN(OCPh_3_) (1-OCPh_3_)

From 1-Cl (150 mg, 0.236 mmol, 1 equiv.) and KOCPh_3_ (63 mg, 0212 mmol, 1 equiv.), 135 mg (0.157 mmol, 74%) of a dark yellow to orange powder was obtained. Single crystals suitable for X-ray diffraction were obtained from a concentrated solution in hexane/dichloromethane at −40 °C. ^1^H NMR (400 MHz, CD_2_Cl_2_) *δ* 7.85 (d, *J* = 2.5 Hz, 1H), 7.56 (m, 6H), 7.52 (d, *J* = 2.4 Hz, 1H), 7.47 (d, *J* = 2.4 Hz, 1H), 7.40 (d, *J* = 2.5 Hz, 1H), 7.14 (m, 6H), 7.07 (m, 3H), 4.51 (s, 3H), 1.37 (s, 9H), 1.35 (s, 9H), 1.24 (s, 9H), 1.24 (s, 9H). ^13^C NMR (101 MHz, CD_2_Cl_2_) *δ* 164.7, 160.1, 153.1, 147.7, 143.0, 141.8, 141.5, 141.5, 140.3, 129.2, 128.0, 126.9, 126.8, 125.6, 124.4, 119.3, 114.6, 113.3, 91.8, 40.3, 36.1, 36.0, 34.9, 34.8, 31.7, 31.6, 30.3, 30.2. UV/VIS/NIR: *λ*_max_ = 313 (*ε* = 41 140 L mol^−1^ cm^−1^), 351 (*ε* = 42 730 L mol^−1^ cm^−1^), 431 (*ε* = 4780 L mol^−1^ cm^−1^). IR (cm^−1^): 2959, 2906, 2871, 1527, 1480, 1448, 1431, 1417, 1394, 1362, 1292, 1256, 1213, 1156, 1131, 1080, 1043, 1033, 1009, 931, 902, 876, 847, 786, 770, 743, 727, 704, 696, 676, 643, 627, 549, 506, 480, 445, 412. Elemental analysis (%) calc'd for C_50_H_58_MoN_4_O_3_: C 69.91, H 6.81, N 6.52; found: C 70.25, H 7.11, N 6.28.

#### L^2^MoN(OCPh_3_) (2-OCPh_3_)

From 2-Cl (135 mg, 0.201 mmol, 1 equiv.) and KOCPh_3_ (60 mg, 0.201 mmol, 1 equiv.), 97 mg (0.109 mmol, 54%) of a pale yellow powder was obtained. Single crystals suitable for X-ray diffraction were obtained from a concentrated solution in pentane/dichloromethane at −40 °C. ^1^H NMR (400 MHz, C_6_D_6_) *δ* 7.87 (m, 3H), 7.85 (m, 3H), 7.82 (m, 2H), 7.57 (s, 4H), 7.04 (m, 6H), 6.97 (m, 2H), 6.88 (m, 3H), 1.56 (s, 18H), 1.34 (s, 18H). ^13^C NMR (101 MHz, C_6_D_6_) *δ* 197.6, 154.2, 147.6, 141.1, 134.2, 129.6, 126.9, 125.6, 124.8, 123.2, 116.2, 114.0, 93.4, 36.2, 34.7, 31.7, 30.7. UV/VIS/NIR: *λ*_max_ = 326 (*ε* = 36 810 L mol^−1^ cm^−1^), 342 (*ε* = 35 430 L mol^−1^ cm^−1^), 357 (*ε* = 37 800 L mol^−1^ cm^−1^), 422 (*ε* = 7370 L mol^−1^ cm^−1^). IR (cm^−1^): 2955, 2865, 1482, 1429, 1382, 1360, 1331, 1290, 1270, 1256, 1231, 1184, 1151, 1117, 1043, 996, 923, 904, 876, 855, 800, 780, 763, 749, 731, 704, 678, 643, 625, 610, 557, 512, 486, 443, 425. Elemental analysis (%) calc'd for C_54_H_59_MoN_3_O_3_·0.33CH_2_Cl_2_: C 70.75, H 6.52, N 4.56; found: C 70.70, H 6.81, N 4.54.

#### L^1^MoN(SCPh_3_) (1-SCPh_3_)

From 1-Cl (150 mg, 0.236 mmol, 1 equiv.) and KSCPh_3_ (82 mg, 0.260 mmol, 1.1 equiv.), 89 mg (0.102 mmol, 43%) of a bright orange powder was obtained. Single crystals suitable for X-ray diffraction were obtained from a concentrated solution in hexane/dichloromethane at −40 °C. ^1^H NMR (400 MHz, CD_2_Cl_2_) *δ* 7.93 (d, *J* = 2.4 Hz, 1H), 7.55 (d, *J* = 2.4 Hz, 1H), 7.49 (d, *J* = 2.4 Hz, 1H), 7.39 (m, 7H), 7.02 (m, 6H), 6.96 (d, *J* = 7.1 Hz, 3H), 4.56 (s, 3H), 1.38 (s, 9H), 1.38 (s, 9H), 1.28 (s, 9H), 1.28 (s, 9H). ^13^C NMR (101 MHz, CD_2_Cl_2_) *δ* 161.1, 160.2, 152.9, 148.5, 142.8, 141.7, 141.4, 141.4, 140.3, 123.0, 128.1, 126.9, 126.4, 125.7, 123.3, 119.2, 114.2, 112.2, 70.6, 69.1, 40.4, 36.0, 35.9, 34.9, 34.8, 31.7, 31.6, 30.4, 30.3. UV/VIS/NIR: *λ*_max_ = 378 (*ε* = 77 510 L mol^−1^ cm^−1^). IR (cm^−1^): 2955, 2903, 2867, 1601, 1525, 1480, 1443, 1417, 1394, 1362, 1292, 1254, 1202, 1186, 1153, 1127, 1074, 1033, 921, 874, 847, 800, 757, 737, 696, 672, 639, 618, 547, 490, 471, 445, 420. Elemental analysis (%) calc'd for C_50_H_58_MoN_4_O_2_S·0.33CH_2_Cl_2_: C 66.92, H 6.55, N 6.20; found: C 66.66, H 6.71, N 6.25.

#### L^2^MoN(SCPh_3_) (2-SCPh_3_)

From 2-Cl (150 mg, 0.224 mmol, 1 equiv.) and KSCPh_3_ (77 mg, 246 mmol, 1.1 equiv.), 103 mg (0.113 mmol, 51%) of a bright orange powder was obtained. Single crystals suitable for X-ray diffraction were obtained from a concentrated solution in hexane/dichloromethane at −40 °C. ^1^H NMR (400 MHz, C_6_D_6_) *δ* 7.94 (m, *J* = 3.2 Hz, 2H), 7.73 (d, *J* = 7.8 Hz, 6H), 7.59 (d, *J* = 2.3 Hz, 2H), 7.57 (d, *J* = 2.4 Hz, 2H), 7.04 (m, *J* = 3.2 Hz, 2H), 6.84 (s, 6H), 6.70 (s, 3H), 1.62 (s, 18H), 1.34 (s, 18H). ^13^C NMR (101 MHz, C_6_D_6_) *δ* 193.6, 154.3, 148.6, 141.2, 134.4, 126.6, 125.0, 124.5, 123.3, 116.2, 113.9, 70.6, 36.1, 34.7, 31.8, 30.7. UV/VIS/NIR: *λ*_max_ = 320 (*ε* = 97 500 L mol^−1^ cm^−1^), 398 (*ε* = 127 610 L mol^−1^ cm^−1^). IR (cm^−1^): 2955, 2867, 1597, 1480, 1441, 1429, 1382, 1360, 1331, 1290, 1266, 1256, 1229, 1205, 1182, 1160, 1133, 1082, 1068, 1031, 988, 921, 876, 855, 800, 761, 745, 737, 698, 672, 645, 623, 610, 553, 476, 443, 420. Elemental analysis (%) calc'd for C_54_H_59_MoN_3_O_2_S: C 71.27, H 6.53, N 4.62; found: C 71.26, H 6.80, N 4.59.

#### L^1^MoN(OMes) (1-OMes)

From 1-Cl (150 mg, 0.236 mmol, 1 equiv.) and LiOMes (33.6 mg, 0.236 mmol, 1 equiv.), 141 mg (0.196 mmol, 83%) of a dark red powder was obtained. Single crystals suitable for X-ray diffraction were obtained from a concentrated solution in benzene. ^1^H NMR (400 MHz, C_6_D_6_) *δ* 8.14 (d, *J* = 2.4 Hz, 1H), 7.70 (d, *J* = 2.4 Hz, 1H), 7.67 (d, *J* = 2.4 Hz, 1H), 7.09 (d, *J* = 2.4 Hz, 1H), 6.97 (s, 1H), 6.83 (s, 1H), 3.09 (s, 3H), 3.07 (s, 2H), 2.45 (s, 2H), 2.24 (s, 3H), 1.62 (s, 9H), 1.59 (s, 9H), 1.40 (s, 9H), 1.37 (s, 9H). ^13^C NMR (101 MHz, C_6_D_6_) *δ* 165.9, 161.6, 161.0, 154.2, 142.3, 142.0, 141.7, 140.8, 140.6, 131.3, 129.8, 128.8, 126.3, 125.6, 124.9, 119.2, 114.4, 113.6, 38.7, 36.3, 36.1, 34.8, 34.6, 31.8, 31.8, 30.1, 30.0, 21.0, 18.2. UV/VIS/NIR: *λ*_max_ = 359 (*ε* = 53 590 L mol^−1^ cm^−1^), 444 (*ε* = 67 400 L mol^−1^ cm^−1^). IR (cm^−1^): 2955, 2869, 1523, 1478, 1445, 1417, 1388, 1362, 1290, 1233, 1205, 1156, 1127, 1088, 1074, 1055, 1037, 957, 921, 874, 849, 800, 772, 757, 745, 733, 712, 692, 643, 608, 557, 541, 500, 478, 447, 416. Elemental analysis (%) calc'd for C_40_H_54_MoN_4_O_3_·C_4_H_8_O: C 65.49, H 7.74, N 6.94; found: C 65.80, H 7.83, N 6.91.

#### L^2^MoN(OMes) (2-OMes)

From 2-Cl (150 mg, 0.224 mmol, 1 equiv.) and LiOMes (32 mg, 0.224 mmol, 1 equiv.), 133 mg (0.173 mmol, 77%) of a dark red powder was obtained. Single crystals suitable for X-ray diffraction were obtained from a concentrated solution in hexane at −40 °C. ^1^H NMR (400 MHz, C_6_D_6_) *δ* 7.78 (m, *J* = 3.3 Hz, 2H), 7.72 (d, *J* = 2.3 Hz, 2H), 7.62 (d, *J* = 2.4 Hz, 2H), 6.93 (m, *J* = 3.1 Hz, 2H), 6.84 (s, 2H), 2.95 (s, 3H), 2.51 (s, 3H), 2.21 (s, 3H), 1.62 (s, 18H), 1.38 (s, 18H). ^13^C NMR (101 MHz, C_6_D_6_) *δ* 197.1, 162.0, 154.8, 141.7, 140.8, 134.4, 132.0, 125.8, 124.7, 123.2, 116.4, 114.0, 36.1, 34.8, 31.8, 30.1, 21.0, 18.0. UV/VIS/NIR: *λ*_max_ = 315 (*ε* = 77 740 L mol^−1^ cm^−1^), 348 (*ε* = 73 530 L mol^−1^ cm^−1^), 459 (*ε* = 112 790 L mol^−1^ cm^−1^). IR (cm^−1^): 2955, 2906, 2865, 1472, 1431, 1386, 1360, 1339, 1288, 1266, 1237, 1158, 1133, 1117, 1070, 1037, 988, 957, 919, 874, 853, 800, 763, 743, 731, 694, 676, 639, 610, 582, 559, 549, 488, 447, 437. Elemental analysis (%) calc'd for C_44_H_55_MoN_3_O_3_: C 68.65, H 7.20, N 5.46; found: C 68.07, H 7.22, N 5.40.

#### L^1^MoN(SMes) (1-SMes)

From 1-Cl (150 mg, 0.236 mmol, 1 equiv.) and KSMes (49 mg, 0.260 mmol, 1.1 equiv.), 56 mg (0.075 mmol, 32%) of a dark red powder was obtained. Single crystals suitable for X-ray diffraction were obtained from a concentrated solution in hexane at −40 °C. ^1^H NMR (400 MHz, C_6_D_6_) *δ* 8.15 (d, *J* = 2.5 Hz, 1H), 7.70 (d, *J* = 2.4 Hz, 1H), 7.66 (d, *J* = 2.4 Hz, 1H), 7.05 (d, *J* = 2.3 Hz, 1H), 6.78 (s, 2H), 3.01 (s, 3H), 2.74 (s, 6H), 2.08 (s, 3H), 1.62 (s, 9H), 1.57 (s, 9H), 1.40 (s, 9H), 1.36 (s, 9H). ^13^C NMR (101 MHz, C_6_D_6_) *δ* 161.8, 161.0, 154.1, 142.6, 142.3, 141.6, 141.5, 141.0, 140.8, 137.6, 136.6, 129.2, 128.6, 126.4, 125.7, 123.9, 119.2, 114.2, 112.9, 38.7, 36.2, 36.1, 34.8, 34.6, 31.8, 31.7, 30.2, 30.1, 24.0, 20.8. UV/VIS/NIR: *λ*_max_ = 351 (*ε* = 113 830 L mol^−1^ cm^−1^), 492 (*ε* = 149 130 L mol^−1^ cm^−1^). IR (cm^−1^): 2955, 2906, 2869, 1605, 1525, 1480, 1445, 1417, 1394, 1362, 1292, 1254, 1202, 1153, 1127, 1086, 1074, 1053, 1037, 921, 874, 849, 800, 772, 757, 743, 712, 688, 678, 641, 551, 490, 476, 447, 418. Elemental analysis (%) calc'd for C_40_H_54_MoN_4_O_2_S·0.5C_6_H_6_: C 65.38, H 7.27, N 7.09; found: C 65.39, H 7.41, N 6.89.

## Conflicts of interest

There are no conflicts to declare.

## Supplementary Material

DT-054-D4DT02405B-s001

DT-054-D4DT02405B-s002

## Data Availability

The spectral data (NMR, IR) as well as electrochemical and computational data supporting this article are available in the ESI† of this article. Crystallographic data have been deposited at the CCDC (for CCDC numbers, see ESI, Tables S1 and S2†) and can be obtained from https://www.ccdc.cam.ac.uk/strucutres.
