# Supplementary material for: Synthesis and electrochemical properties of molybdenum nitrido complexes supported by redox-active NHC and MIC ligands
Source: Dalton Trans. 2024 Nov 1;54(2):582–94. doi: 10.1039/d4dt02405b (PMC11572837; doi:10.1039/d4dt02405b)
Supplement: DT-054-D4DT02405B-s001 [file DT-054-D4DT02405B-s001.pdf]

Supplementary Information (SI) for Dalton Transactions.
This journal is © The Royal Society of Chemistry 2024

Supplementary Information for

Synthesis and Electrochemical Properties of Molybdenum Nitrido Complexes Supported by Redox-Active NHC and MIC Ligands

Daniel Leitner,[a] Florian R. Neururer[a] and Stephan Hohloch[a]*

Table of contents:

1. NMR spectroscopy 2
2. IR spectroscopy 82
3. UV-Vis Spectroscopy 87
4. Cyclic Voltammetry 94
5. Computational Details 100
6. Crystallographic Information 104

[a] *Department of General, Inorganic and Theoretical Chemistry, University of Innsbruck, Innrain 80-82, 6020 Innsbruck, Austria, E-Mail:* Stephan.Hohloch@uibk.ac.at
Electronic Supplementary Information (ESI) available: [details of any supplementary information available should be included here]. See DOI: 10.1039/x0xx00000x

1. NMR spectroscopy

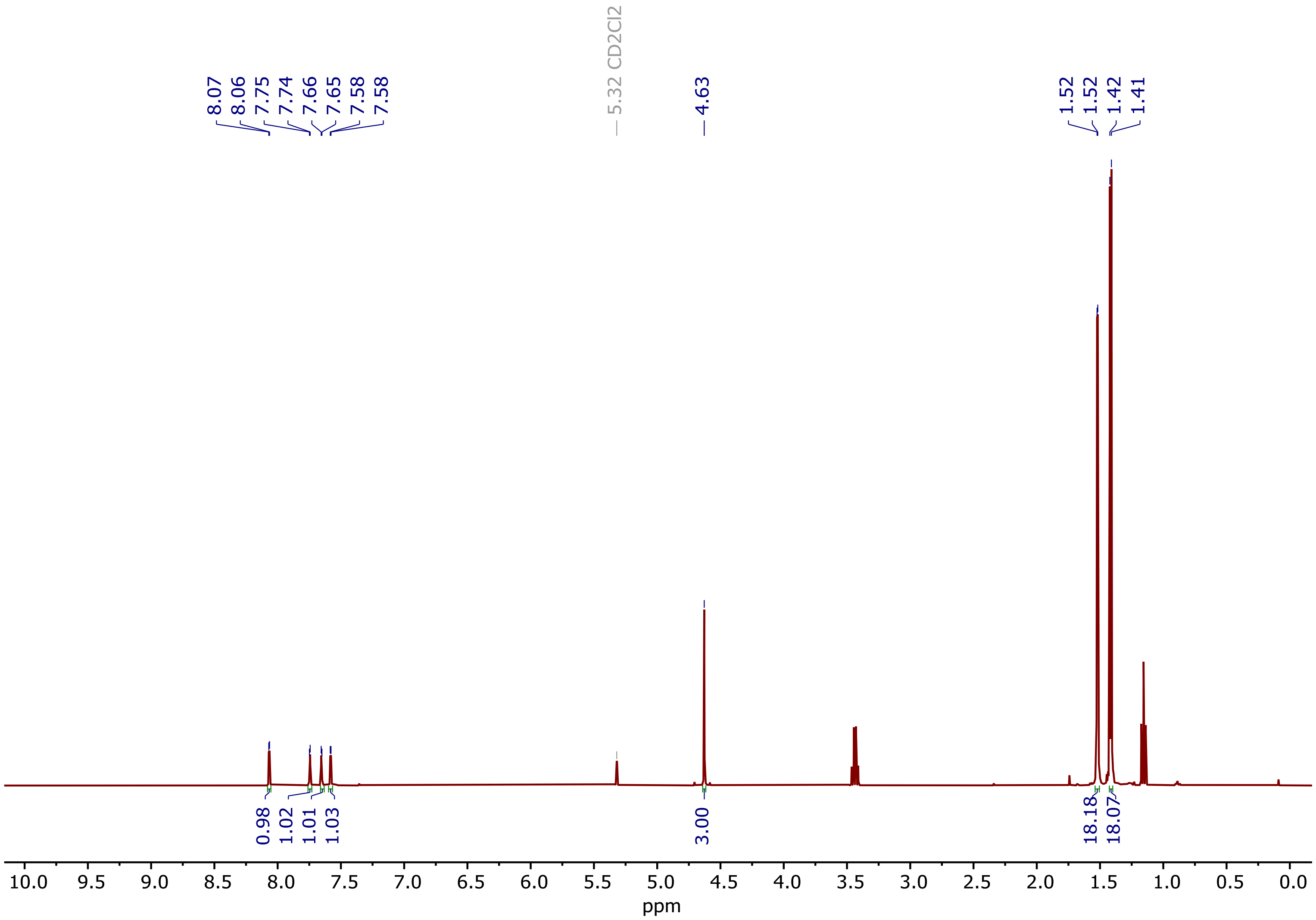

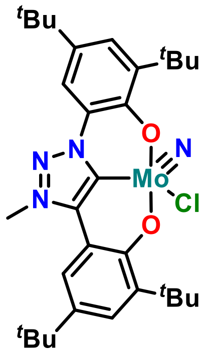

*Figure S 1: ^{1}H NMR of **1-Cl** in CD_2Cl_2 at 298 K.*

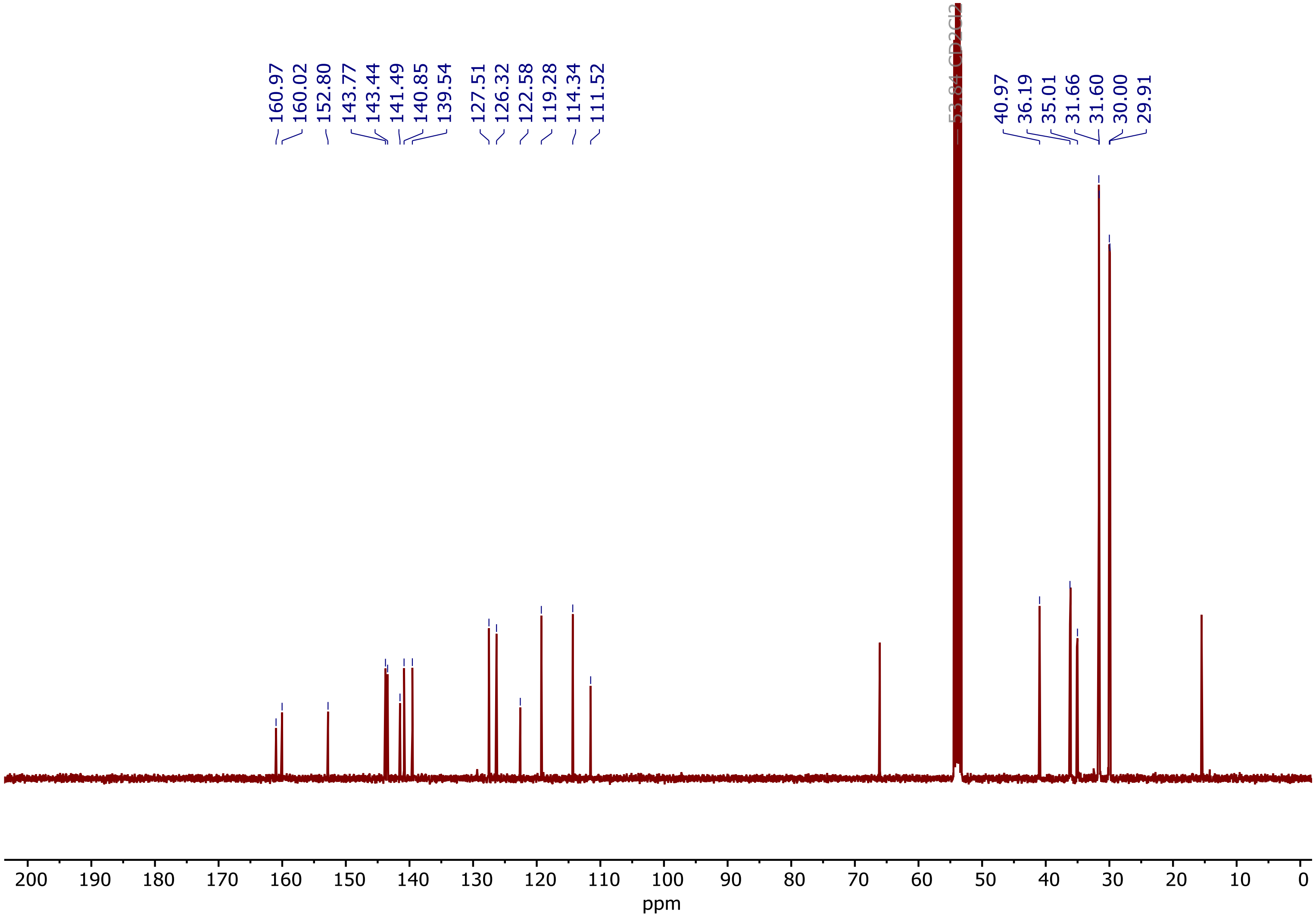

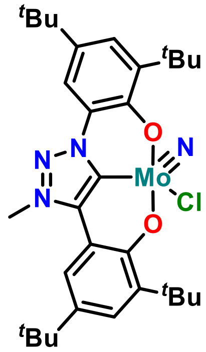

*Figure S 2: ^{13}C NMR of **1-Cl** in CD_2Cl_2 at 298 K.*

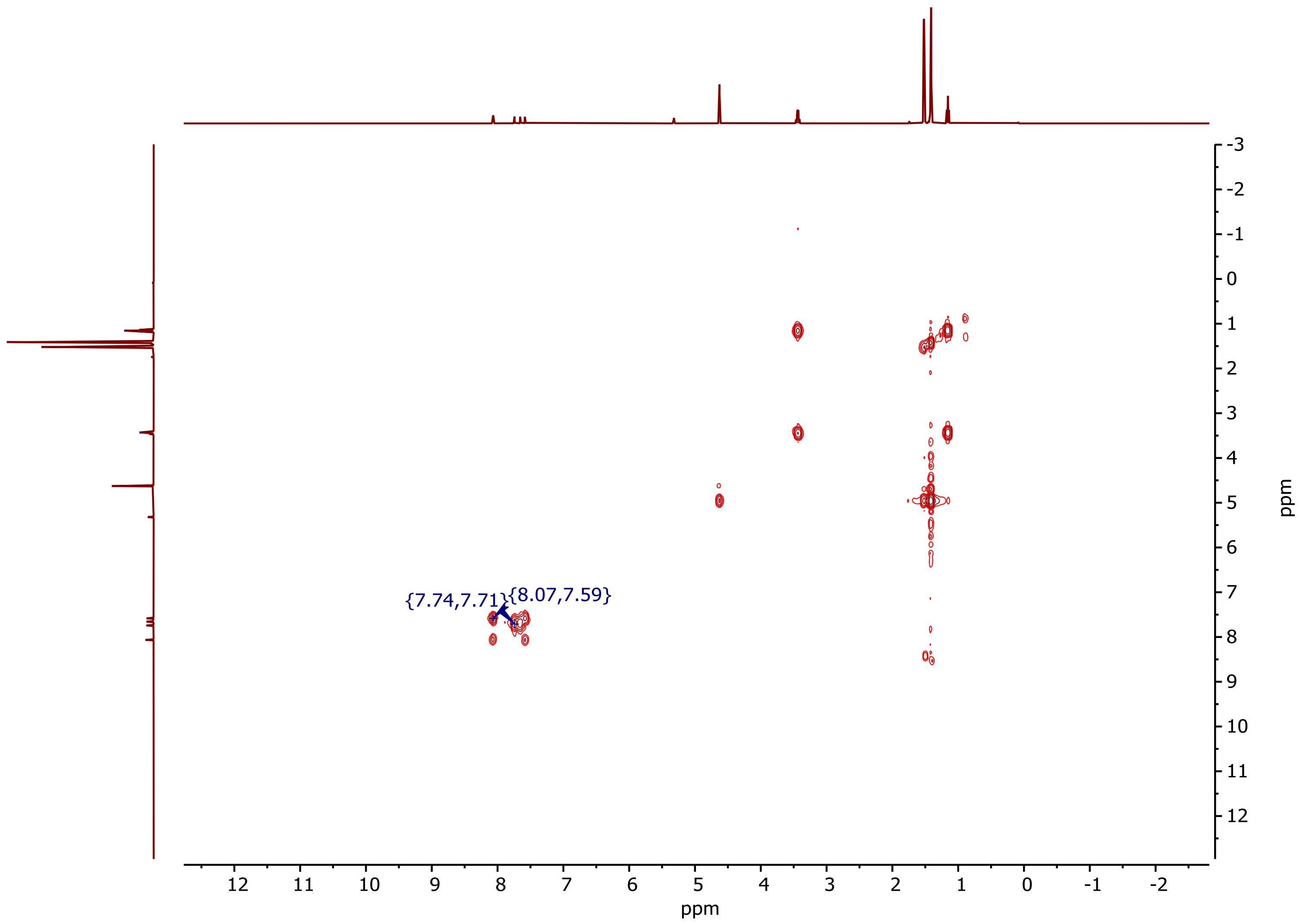

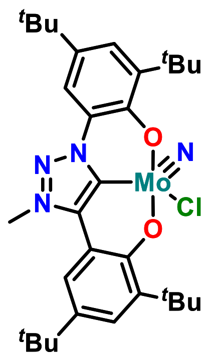

*Figure S 3: 1H – 1H COSY of **1-Cl** in CD_2Cl_2 at 298 K.*

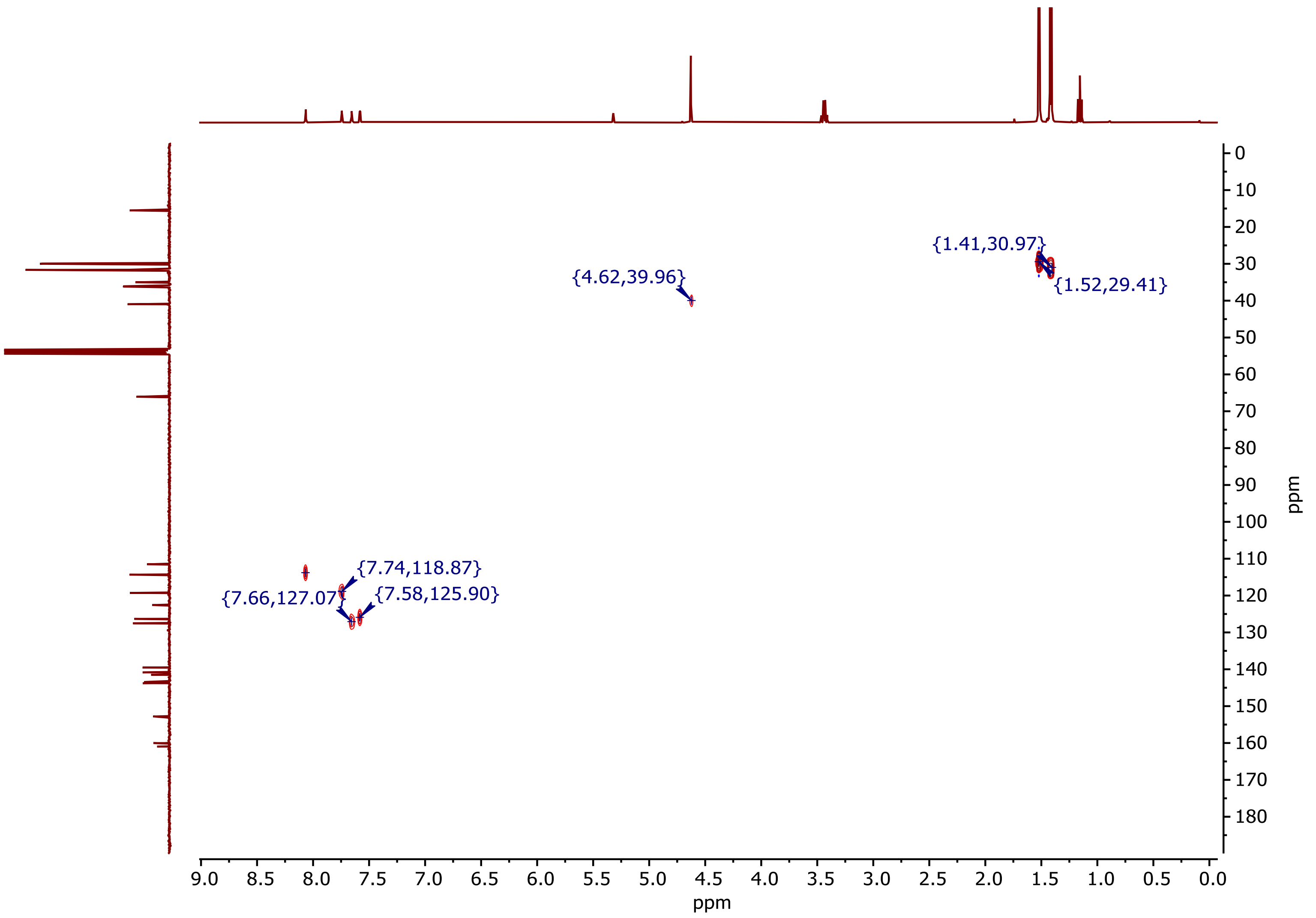

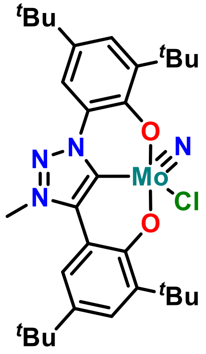

*Figure S 4: ^{1}H – ^{13}C HSQC of **1-Cl** in CD_2Cl_2 at 298 K.*

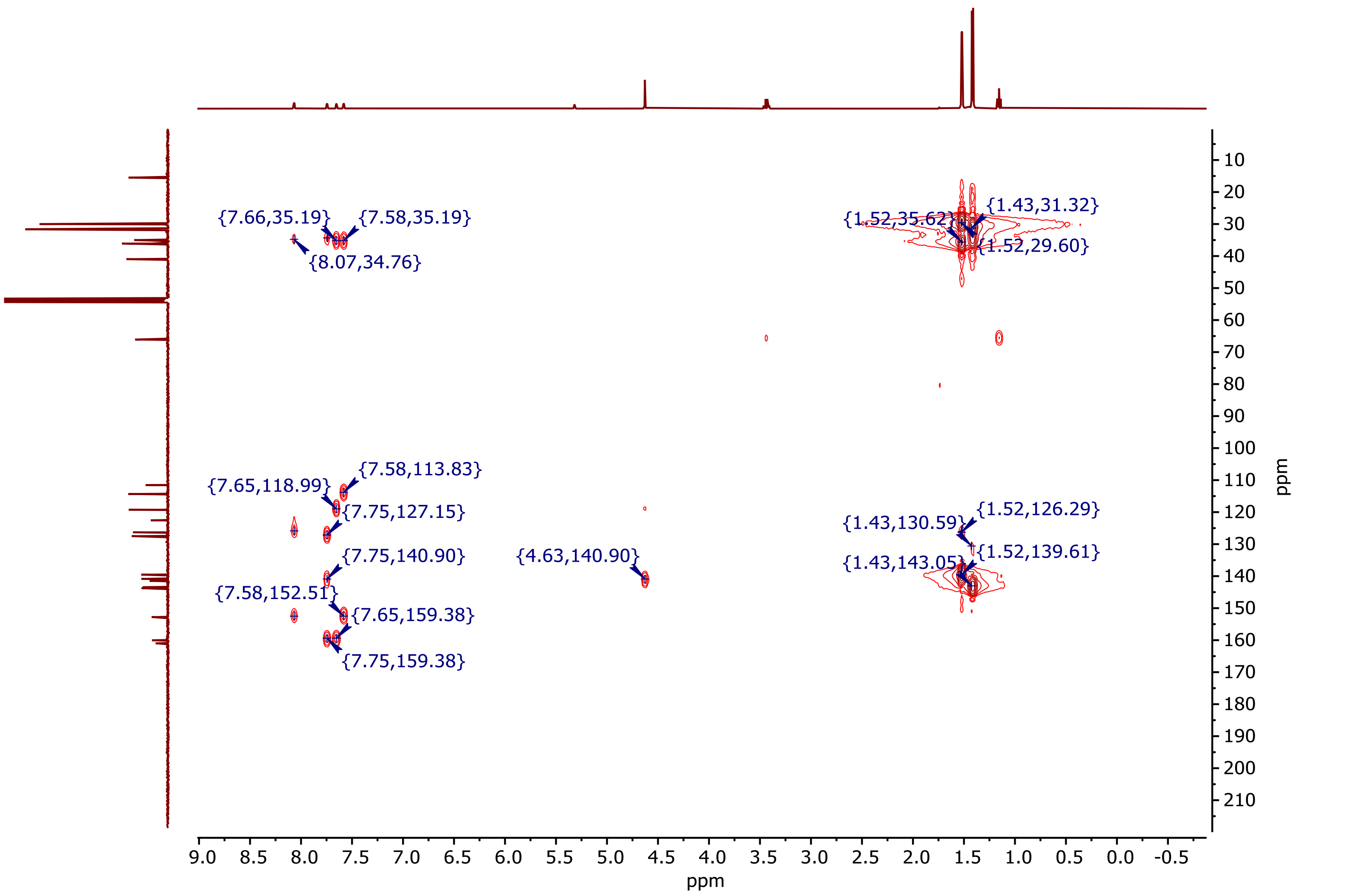

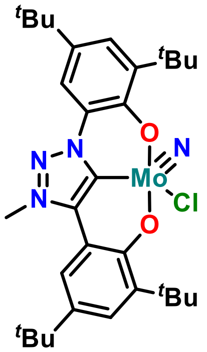

*Figure S 5: $^{1}H - ^{13}C$ HMBC of **1-Cl** in CD_2Cl_2 at 298 K.*

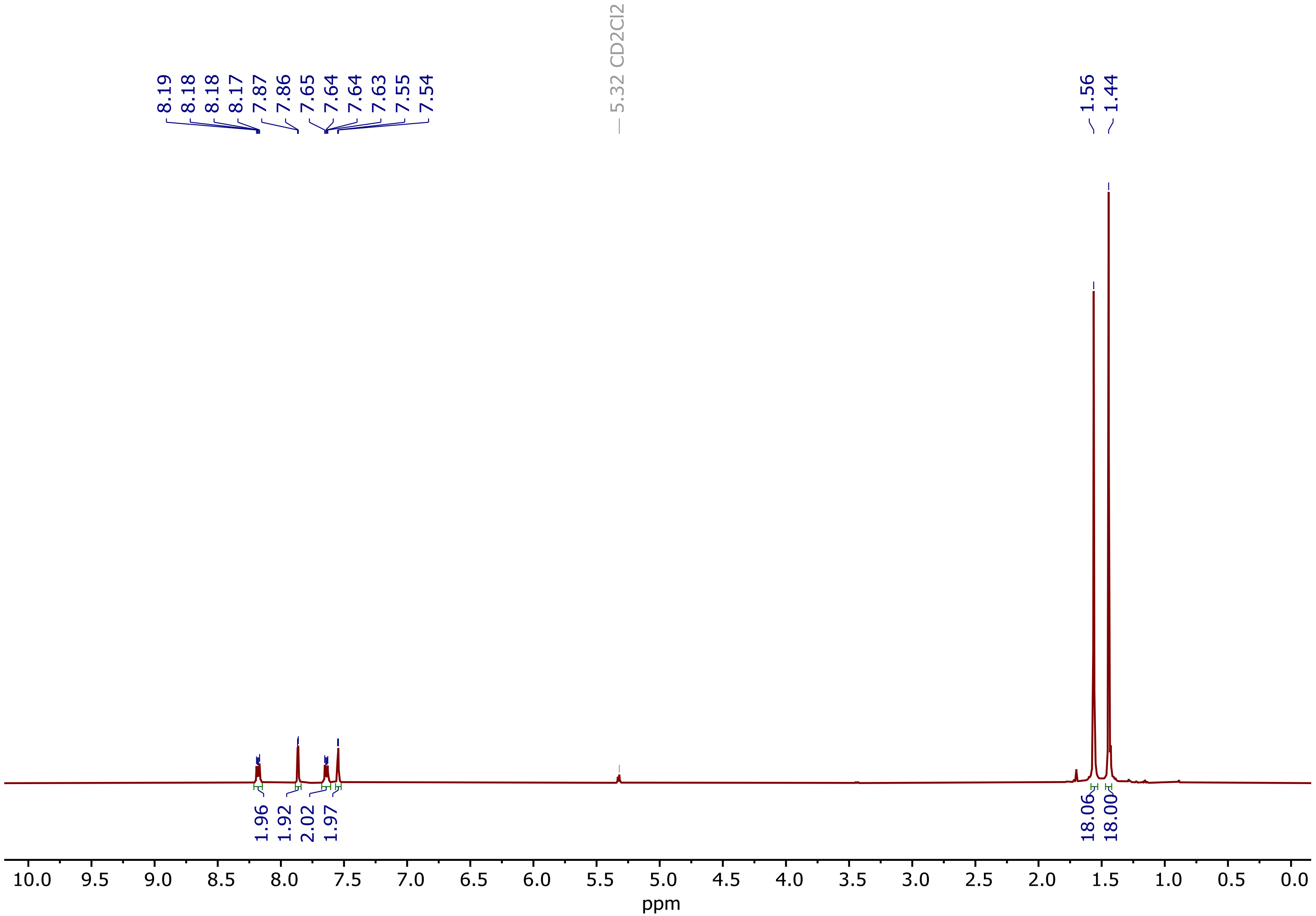

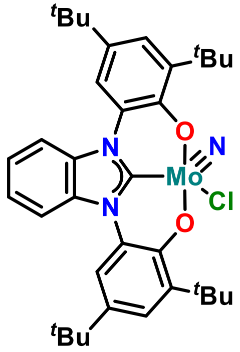

*Figure S 6: ^{1}H NMR of **2-Cl** in CD_2Cl_2 at 298 K.*

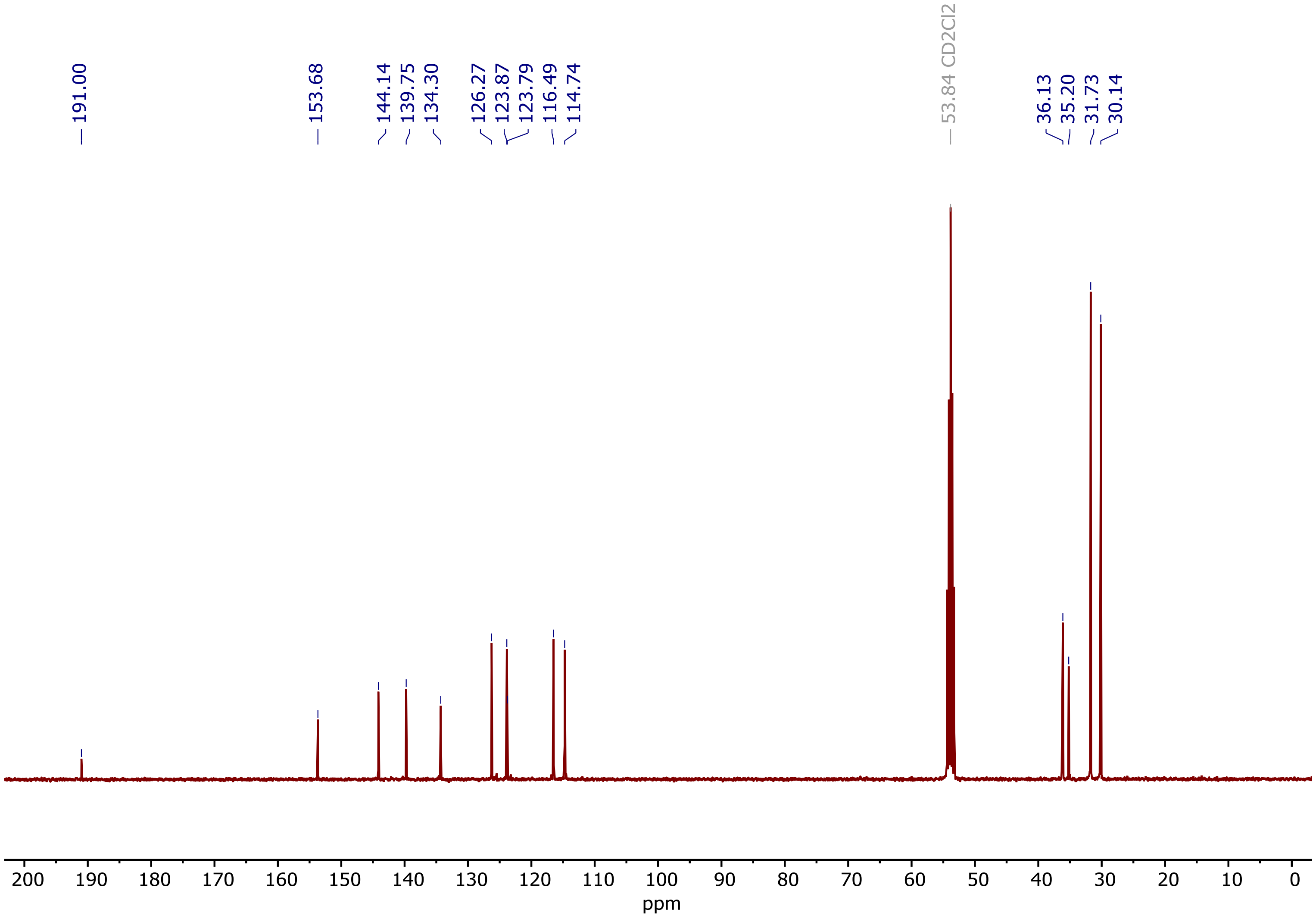

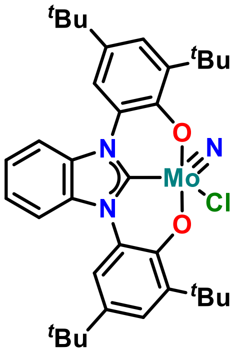

*Figure S 7: ^{13}C NMR of **2-Cl** in CD_2Cl_2 at 298 K.*

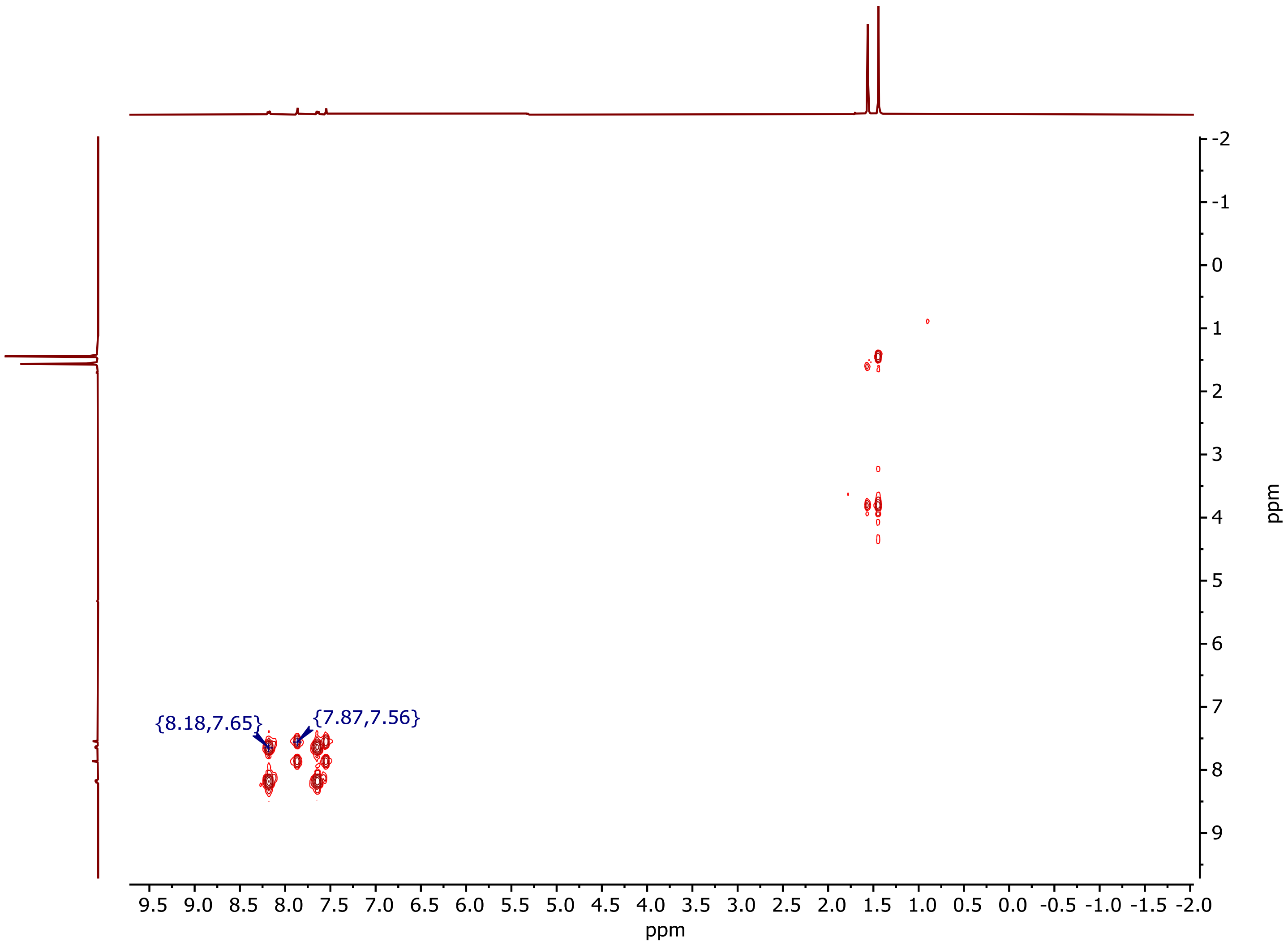

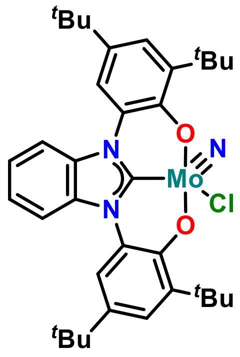

*Figure S 8: $^{1}H - ^{1}H$ COSY of **2-Cl** in CD_2Cl_2 at 298 K.*

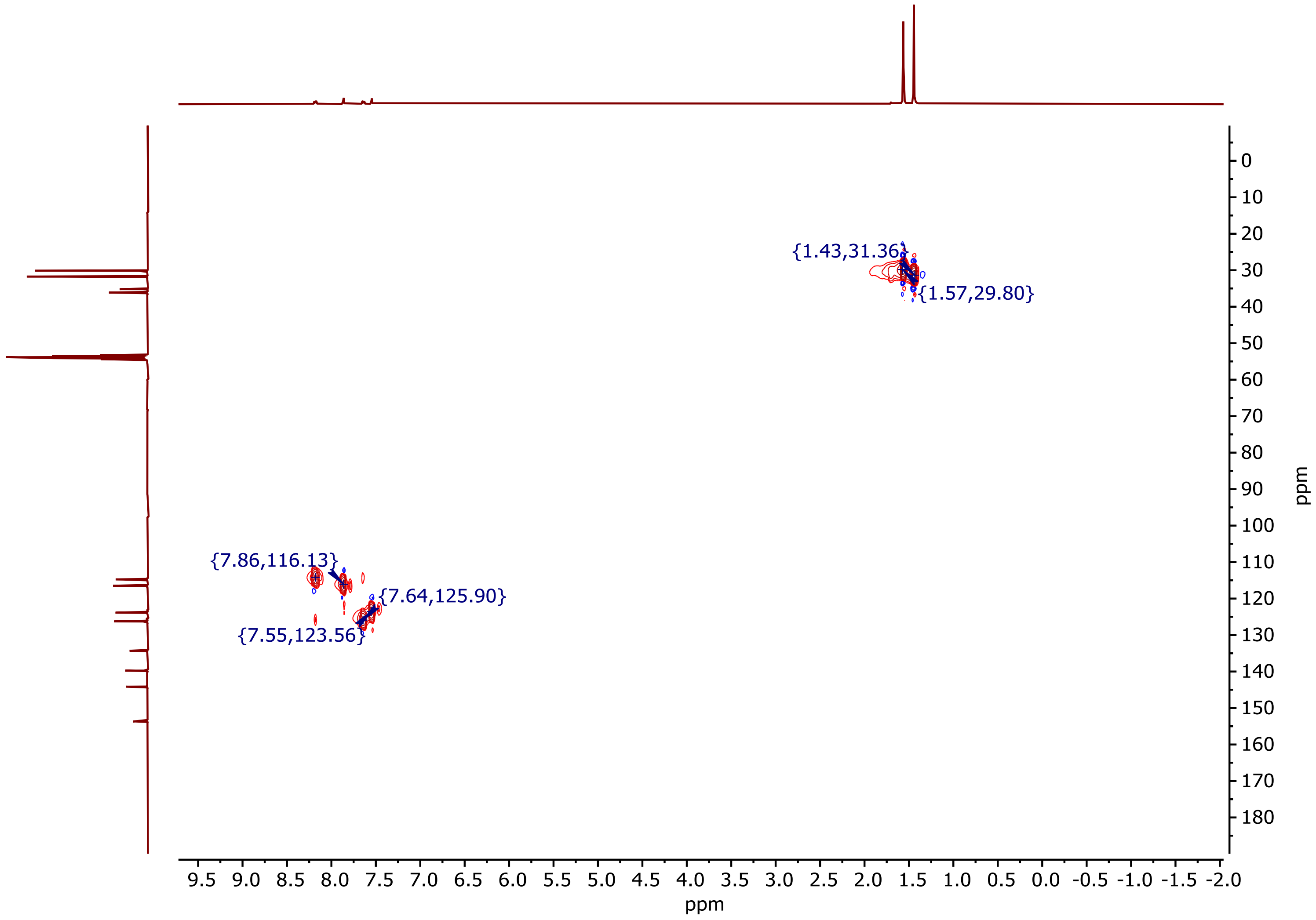

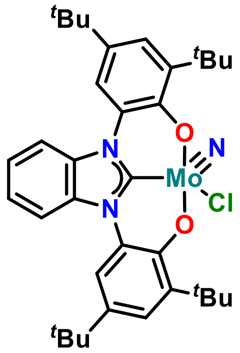

*Figure S 9: ^1H – ^{13}C HSQC of **2-Cl** in CD_2Cl_2 at 298 K.*

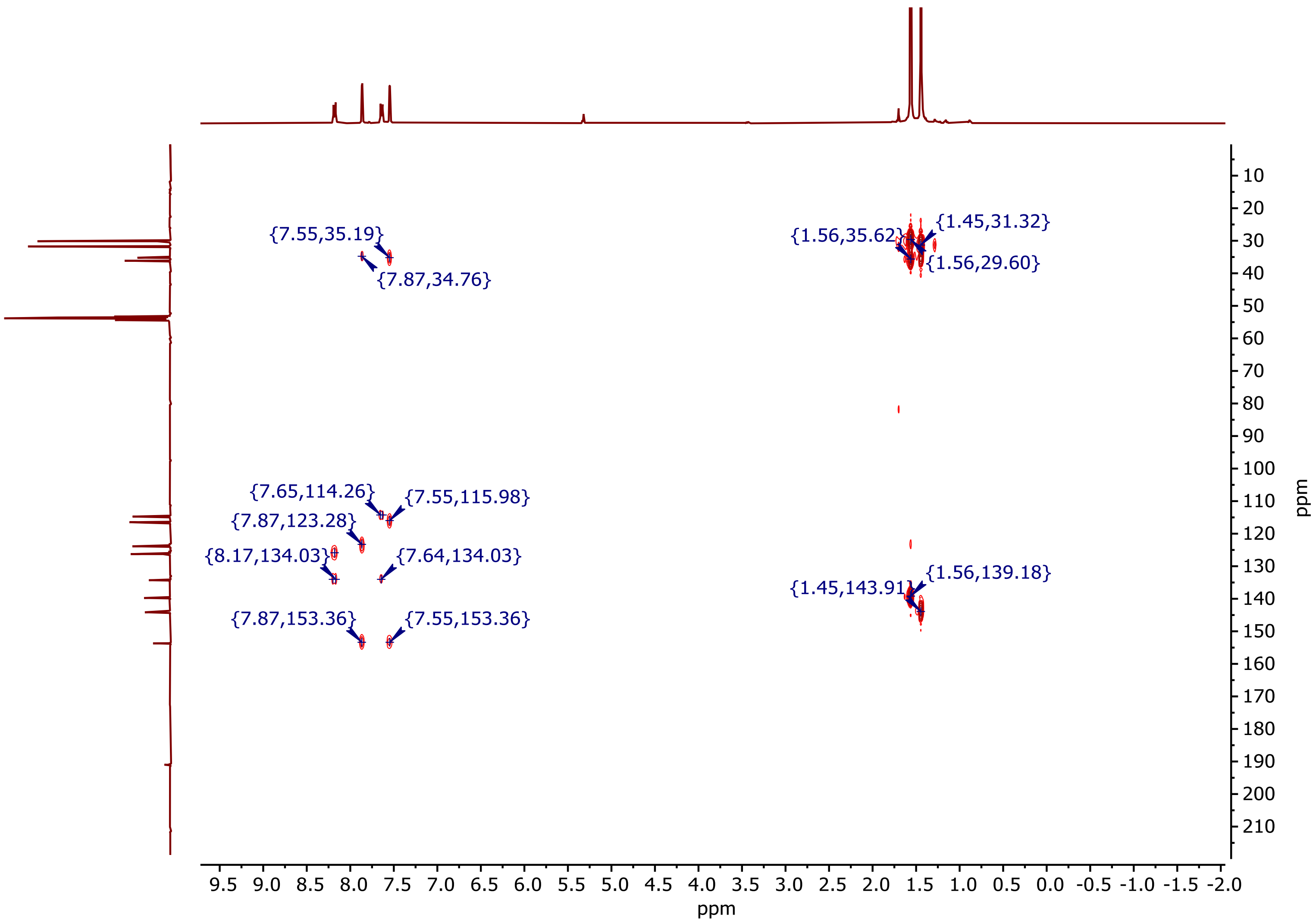

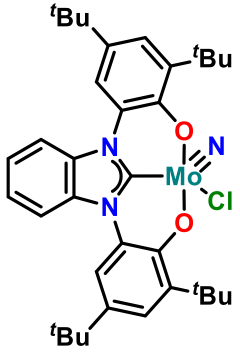

*Figure S 10: ^{1}H – ^{13}C HMBC of **2-Cl** in CD_2Cl_2 at 298 K.*

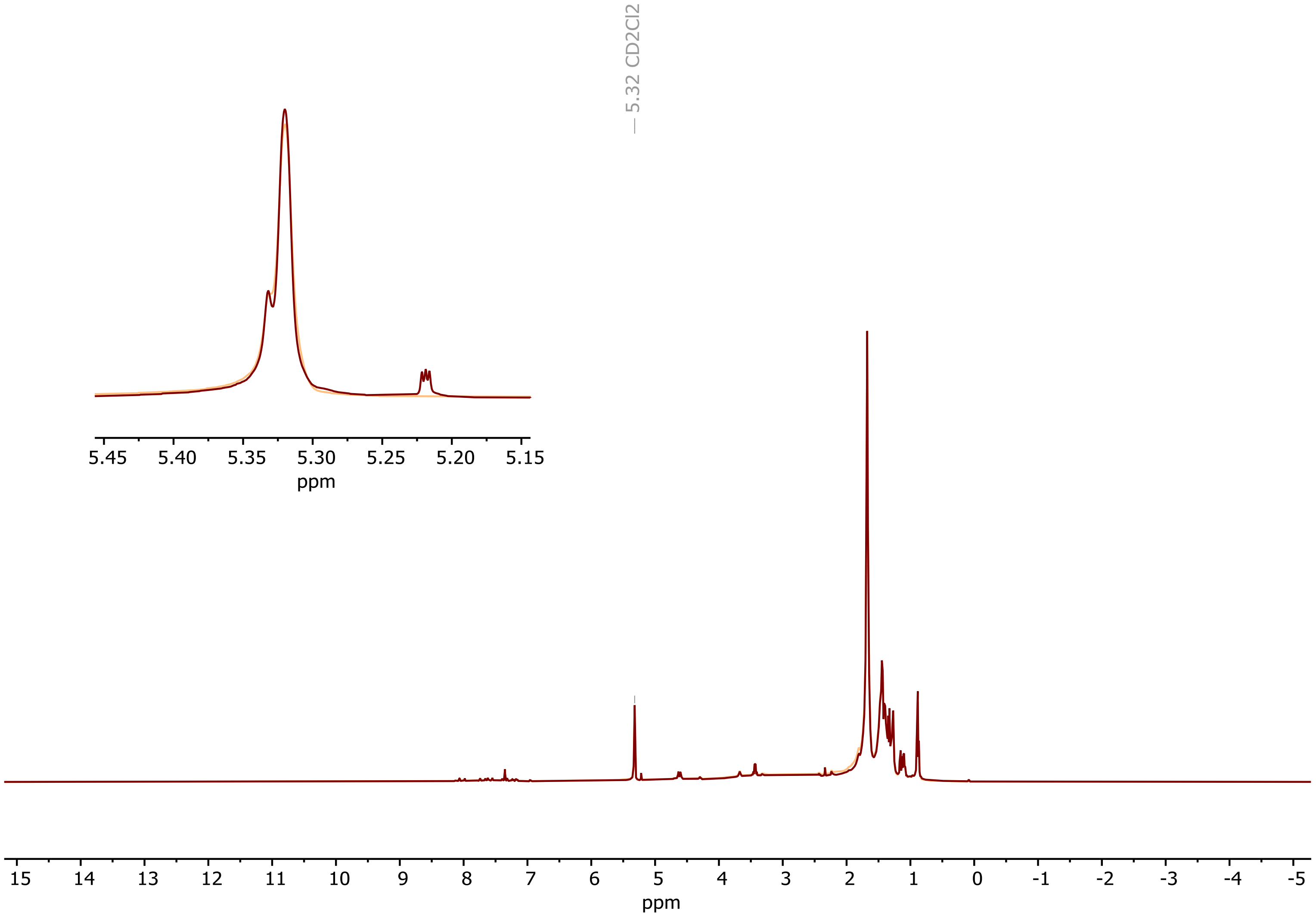

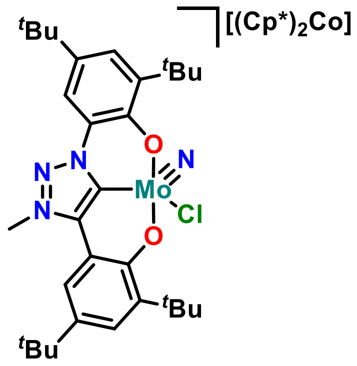

*Figure S 11: ¹H NMR of **[1]**⁻ without (orange) and with (maroon) a CD_2Cl_2 capillary in CD_2Cl_2 at 298 K.*

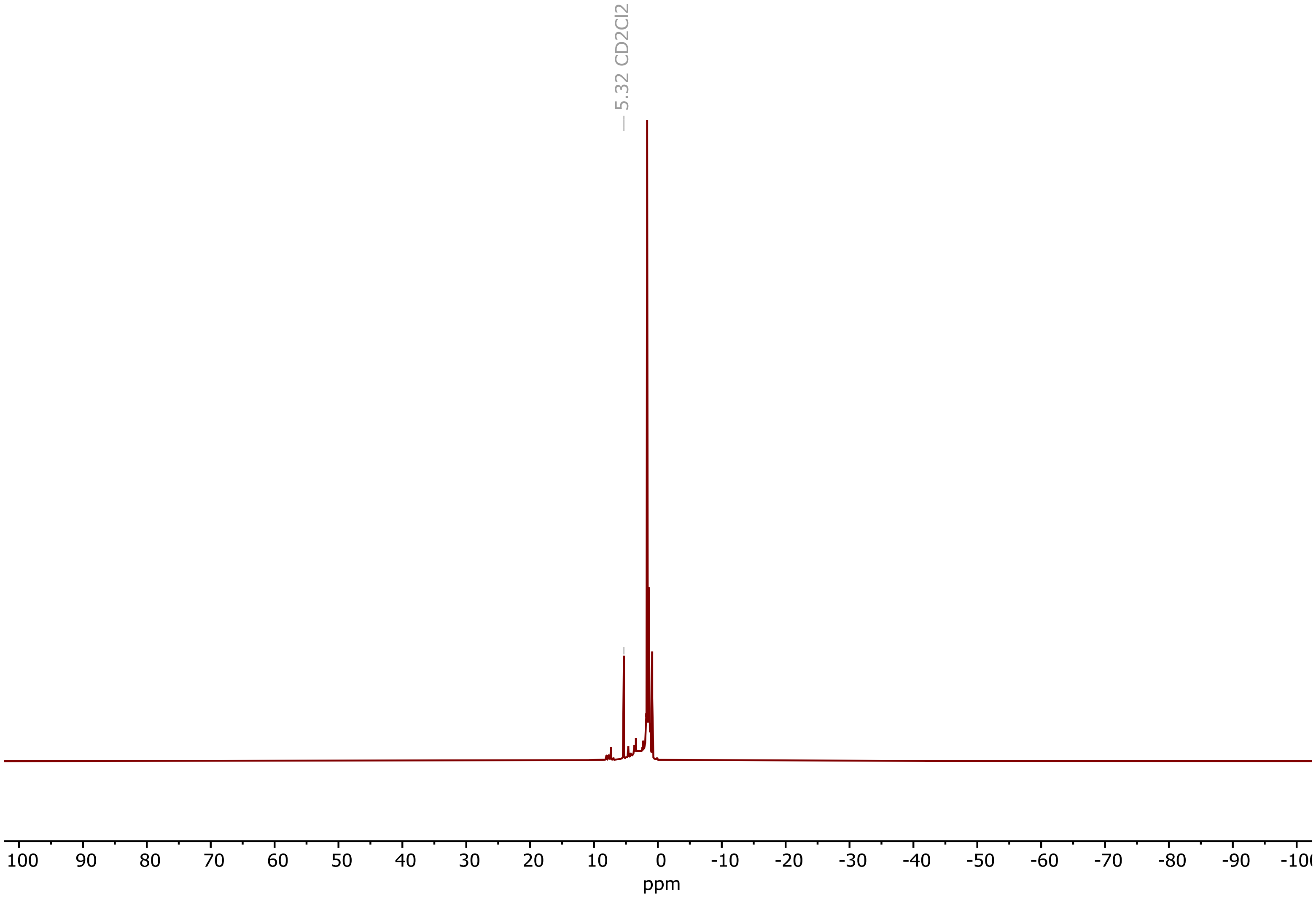

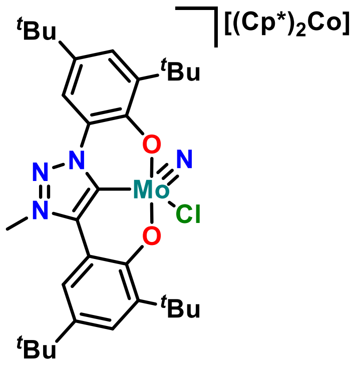

Figure S 12: ¹H NMR of ***[1]****⁻ from -100 to 100 ppm in* CD_2Cl_2 *at 298 K.*

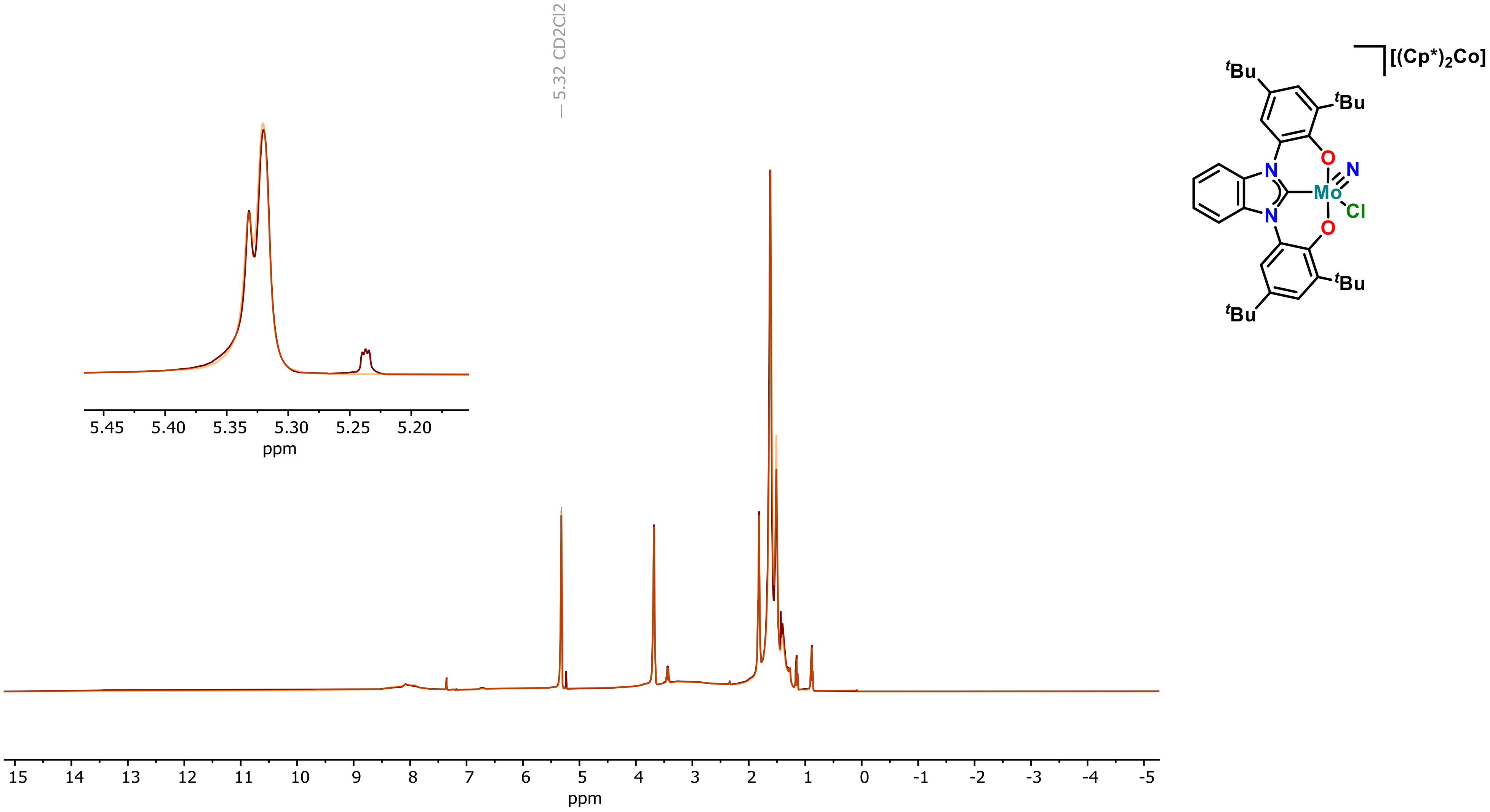

*Figure S 13: ^{1}H NMR of **[2]**$^{-}$ without (orange) and with (maroon) a CD_2Cl_2 capillary in CD_2Cl_2 at 298 K.*

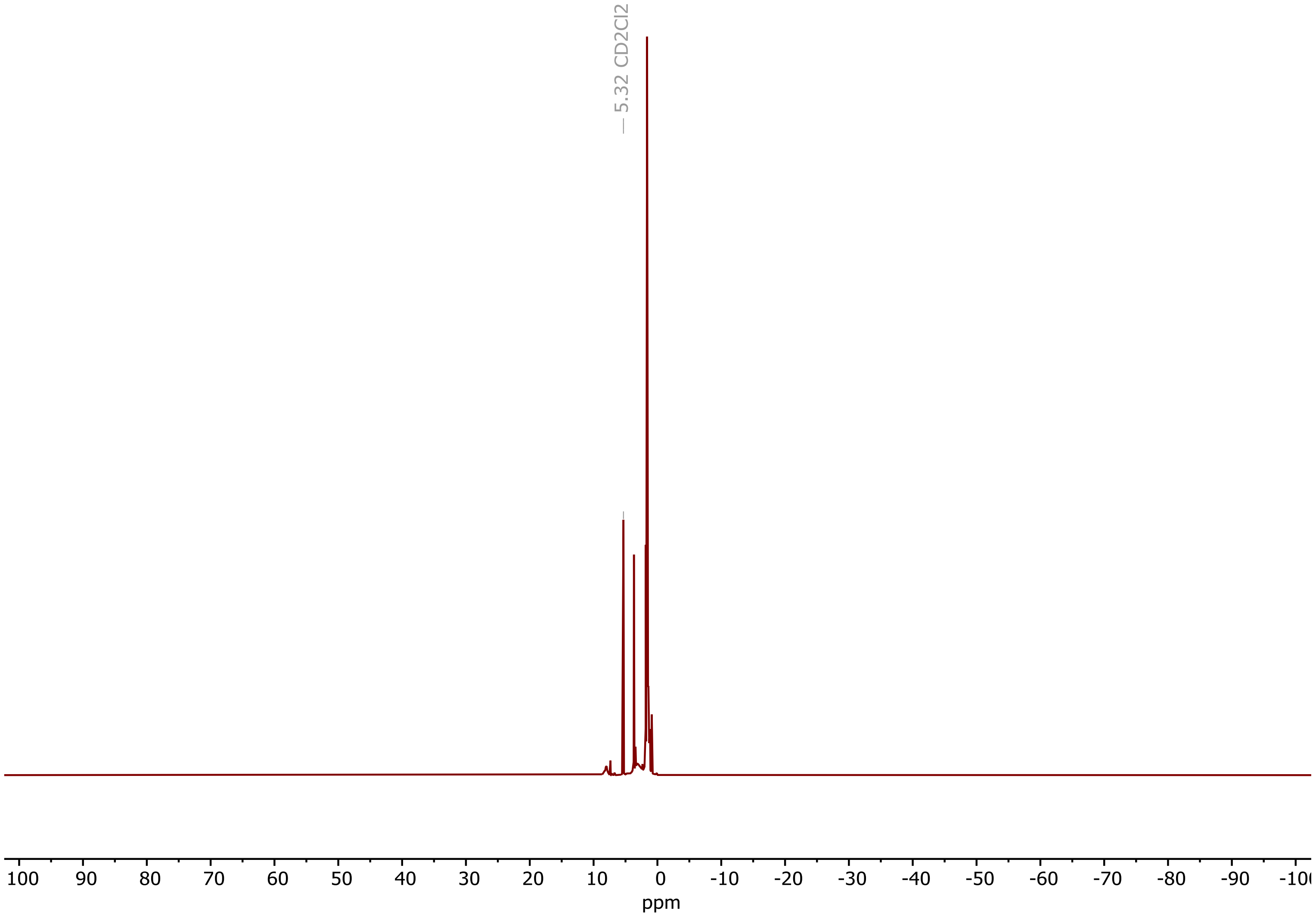

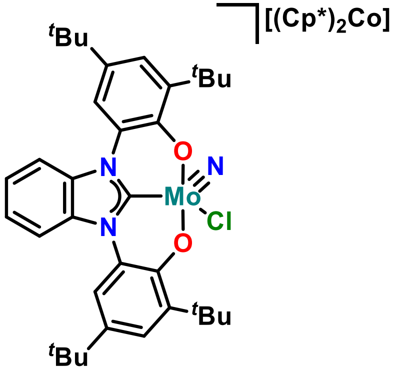

*Figure S 14: ¹H NMR of **[2]**⁻ from -100 to 100 ppm in CD_2Cl_2 at 298 K.*

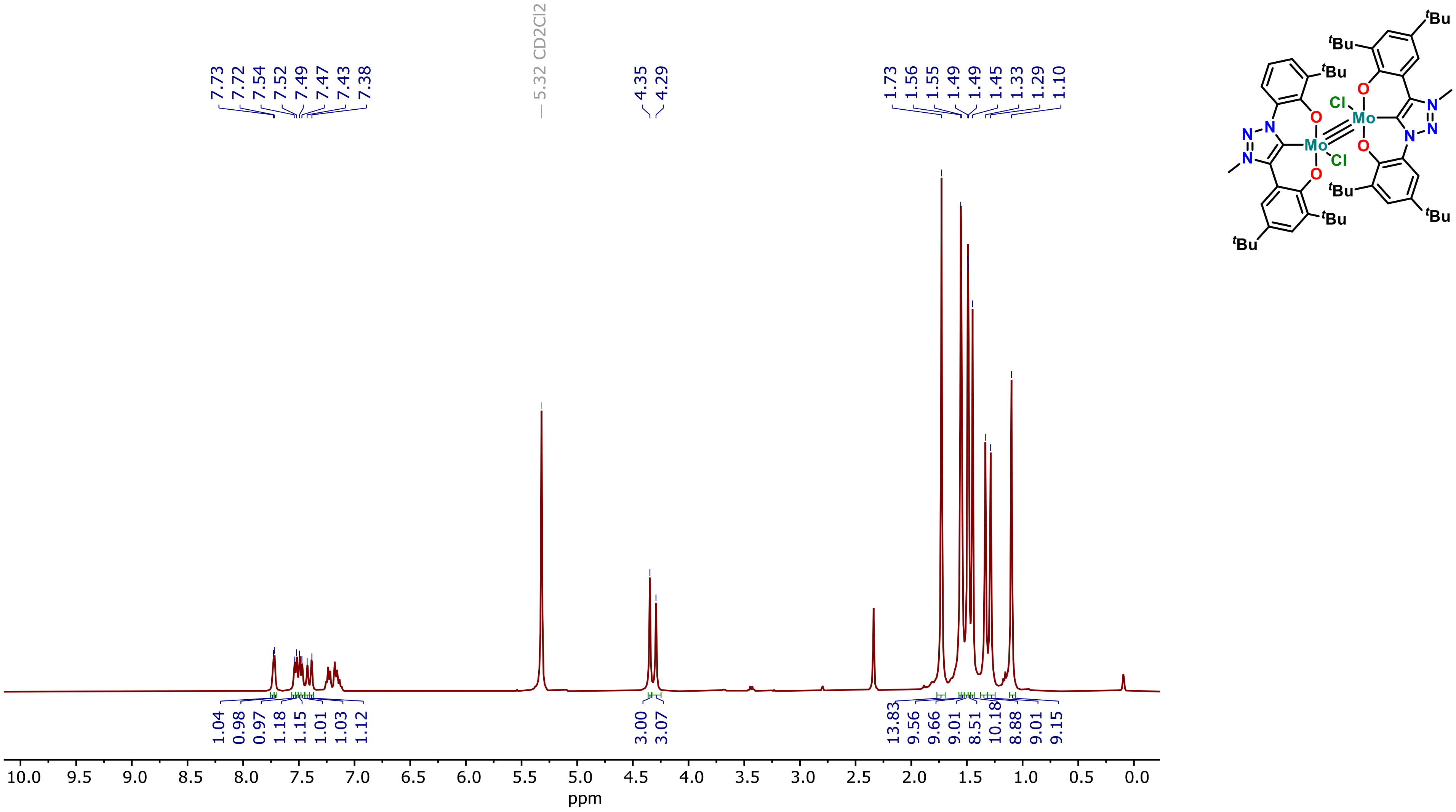

*Figure S 15: ^{1}H NMR of dimer **3** in CD_2Cl_2 at 298 K. The spectrum includes toluene from the crystallization and residual decamethylcobaltocenium triflate (1.73 ppm, 13H).*

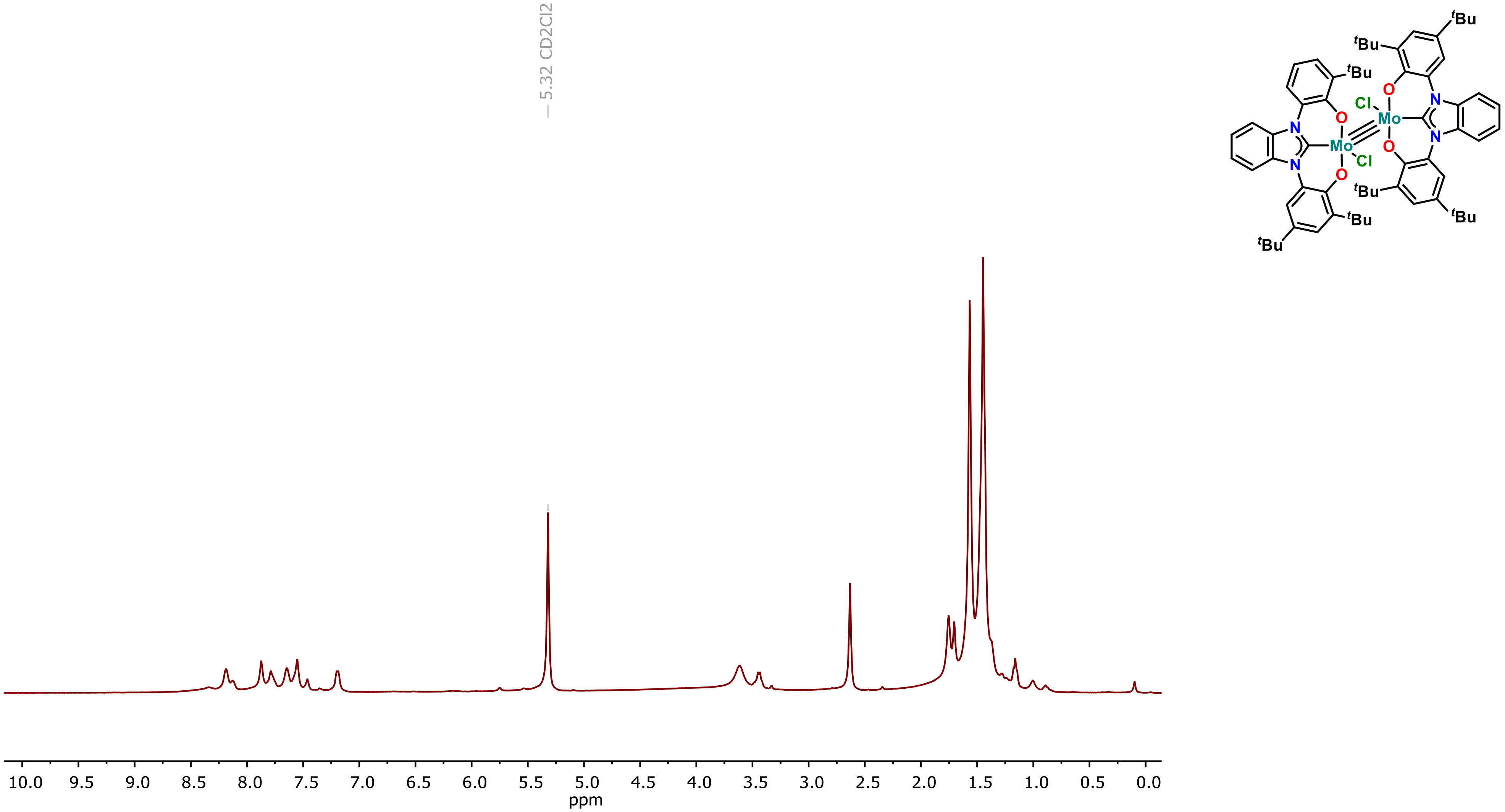

*Figure S 16: Crude ^{1}H NMR after reduction of **2-Cl** under protic conditions. The line broadening is attributed to paramagnetic impurities and no single species can be identified.*

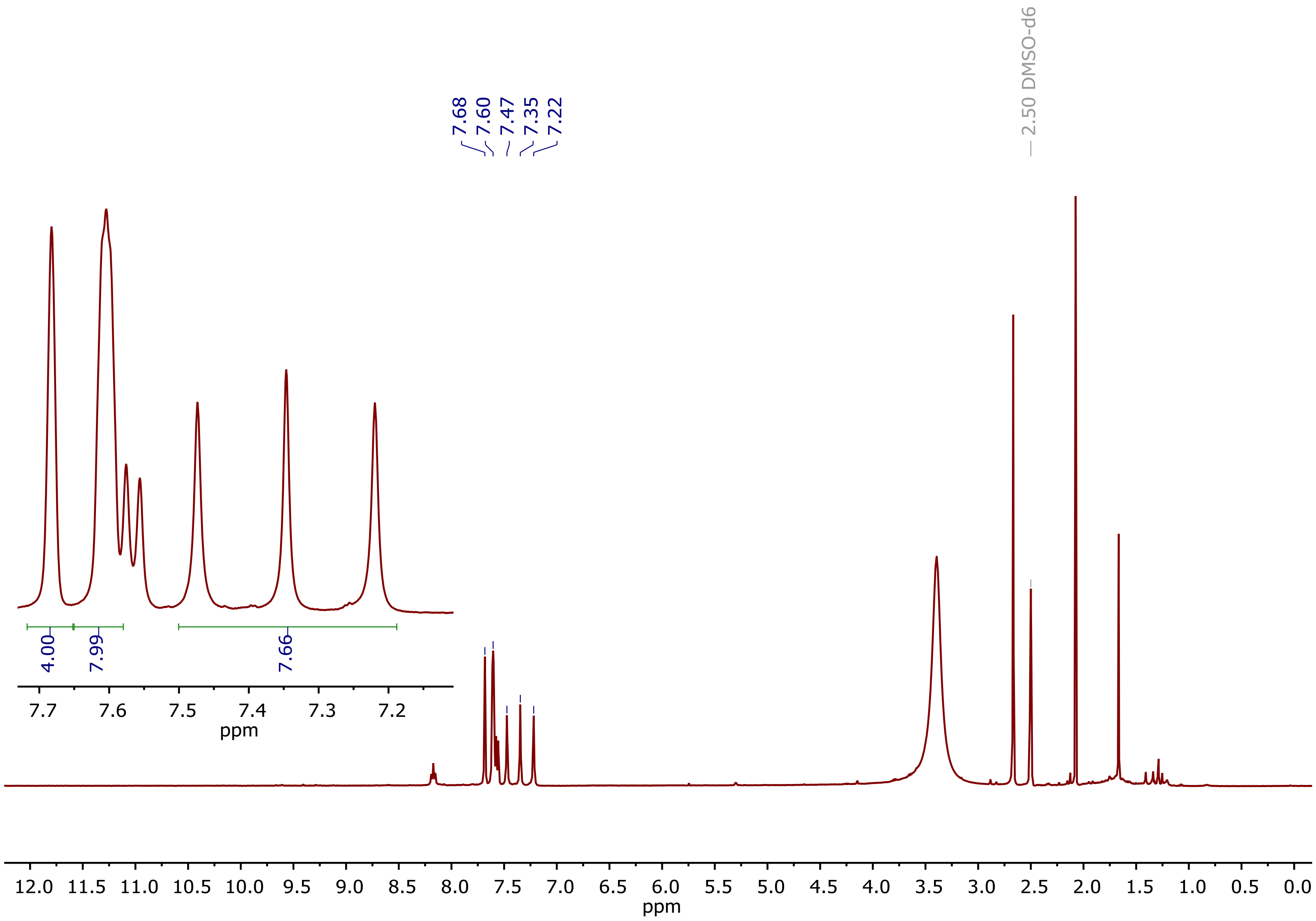

*Figure S 17: ^{1}H NMR in DMSO-d$_6$ for ammonium quantification after aqueous workup of the reduction of **1-Cl** (39 µmol) under protic conditions. The inlay shows the characteristic ammonium triplet and NaBArF$_{24}$ is used as an internal standard (17.7 mg, 20 µmol). The signals at 8.17, 7.57 and 2.67 result from residual lutidine, the signals at 2.07 belongs to residual acetone, while the signal at 1.66 is an unknown impurity.*

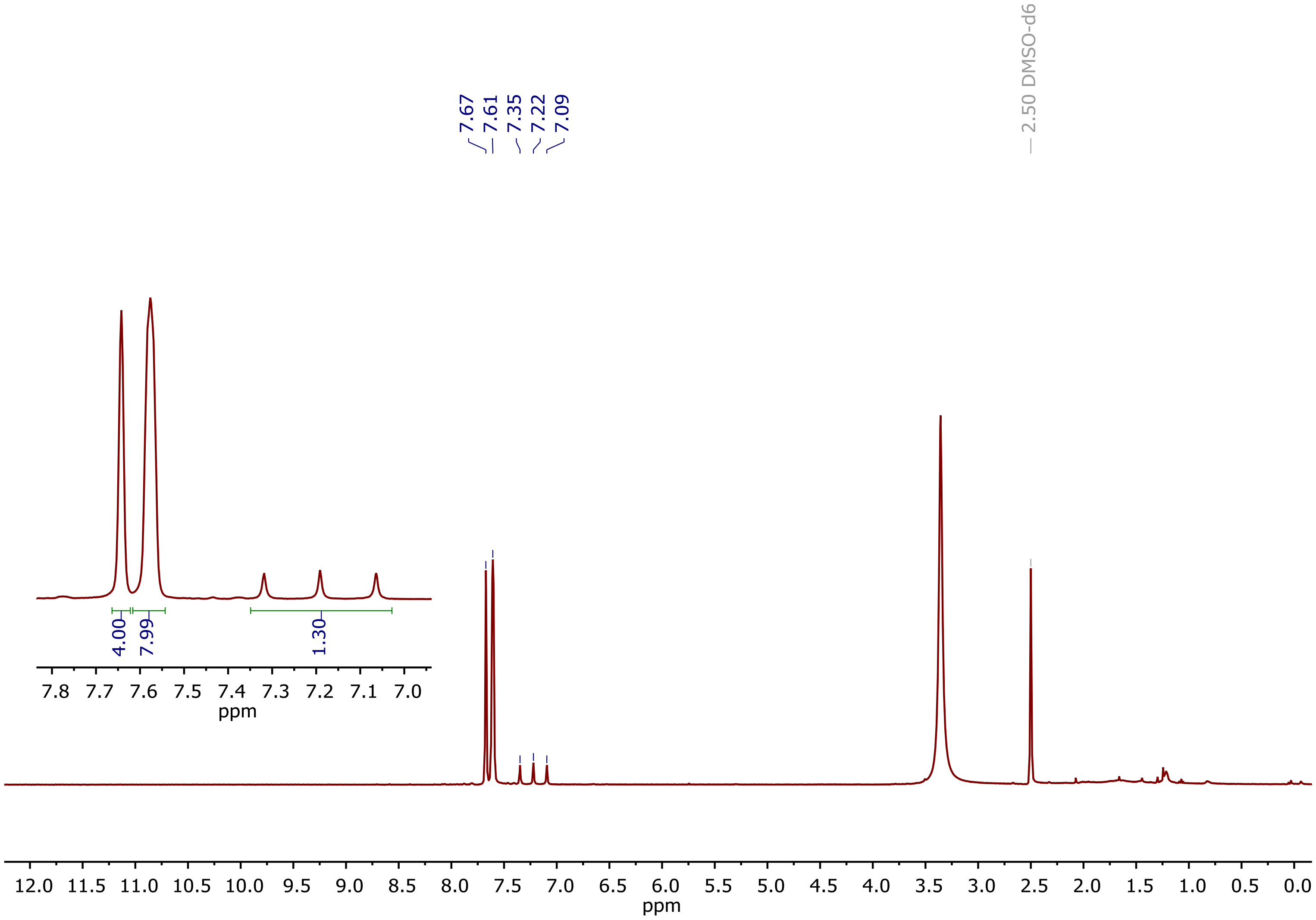

*Figure S 18: ^{1}H NMR in DMSO-d_6 for ammonium quantification after aqueous workup of the reduction of **2-Cl** (37 µmol) under protic conditions. The inlay shows the characteristic ammonium triplet and $NaBArF_{24}$ is used as an internal standard (17.7 mg, 20 µmol).*

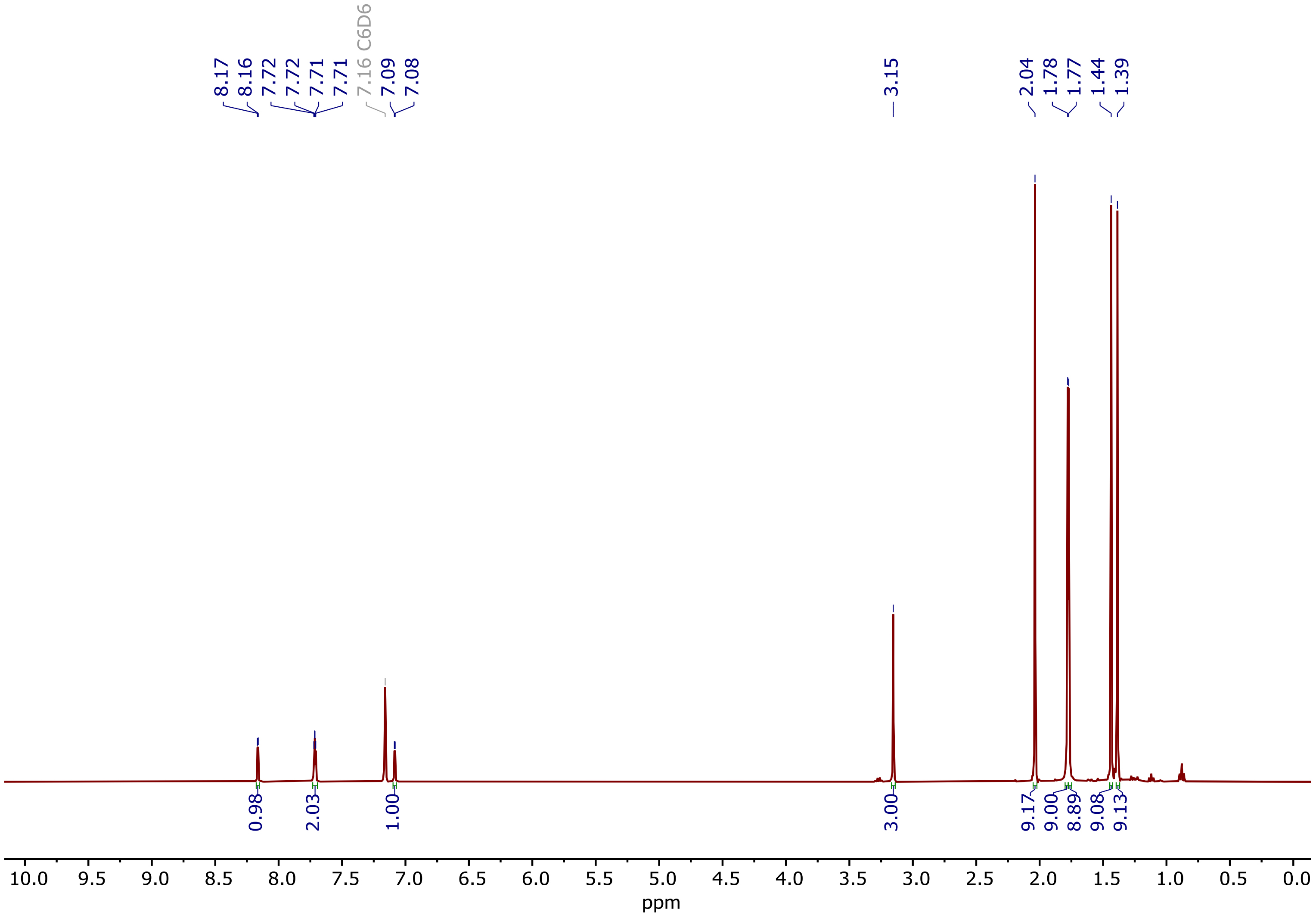

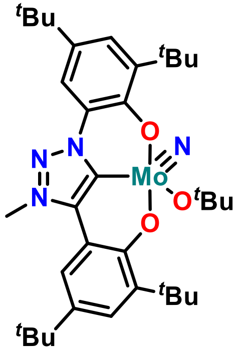

*Figure S 19: ^{1}H NMR of **1-O^{t}Bu** in C_6D_6 at 298 K.*

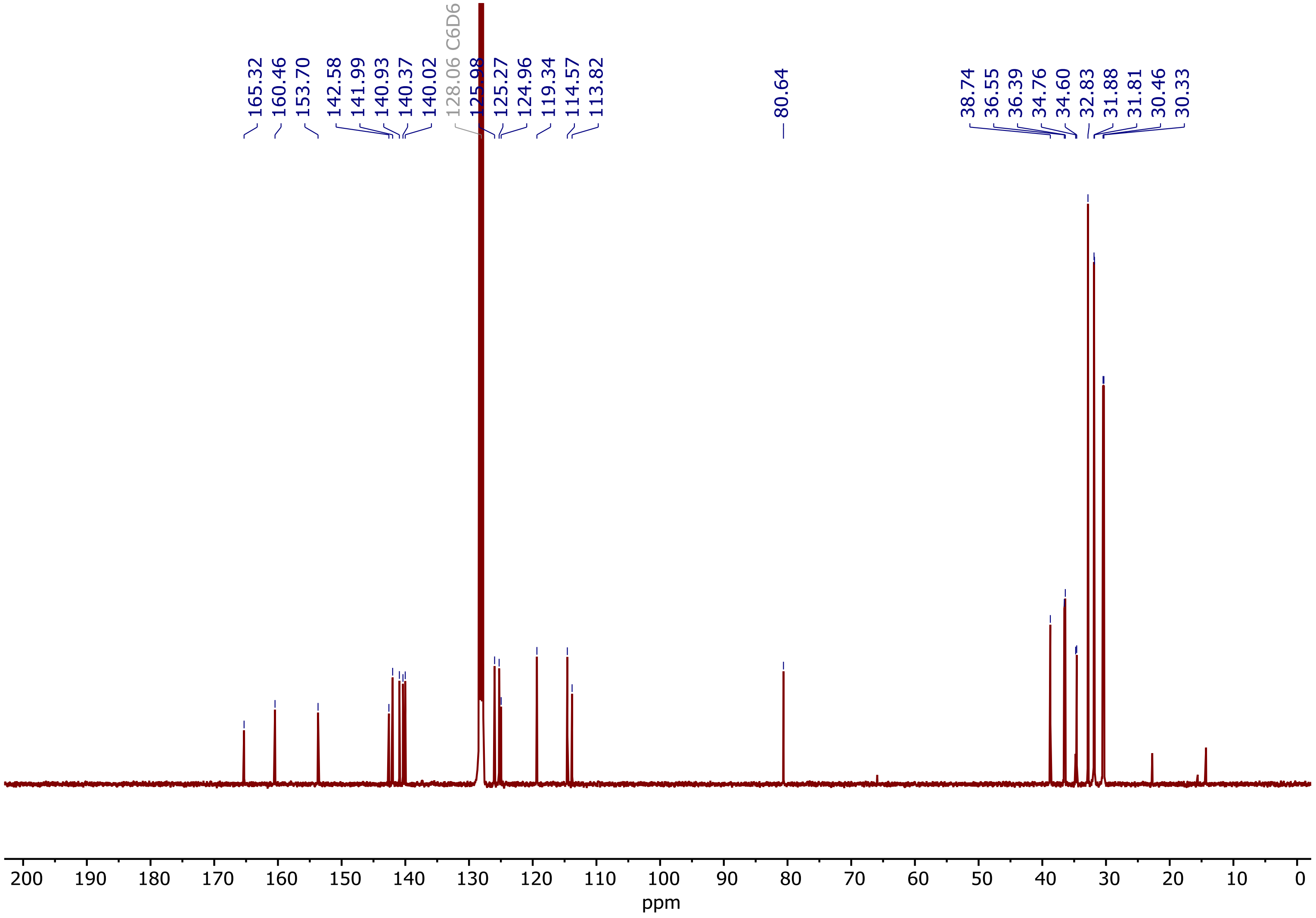

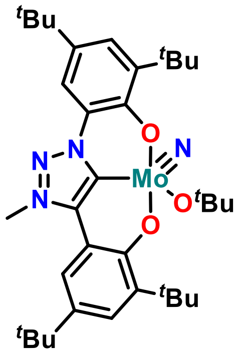

*Figure S 20: ^{13}C NMR of **1-O^{t}Bu** in C_6D_6 at 298 K.*

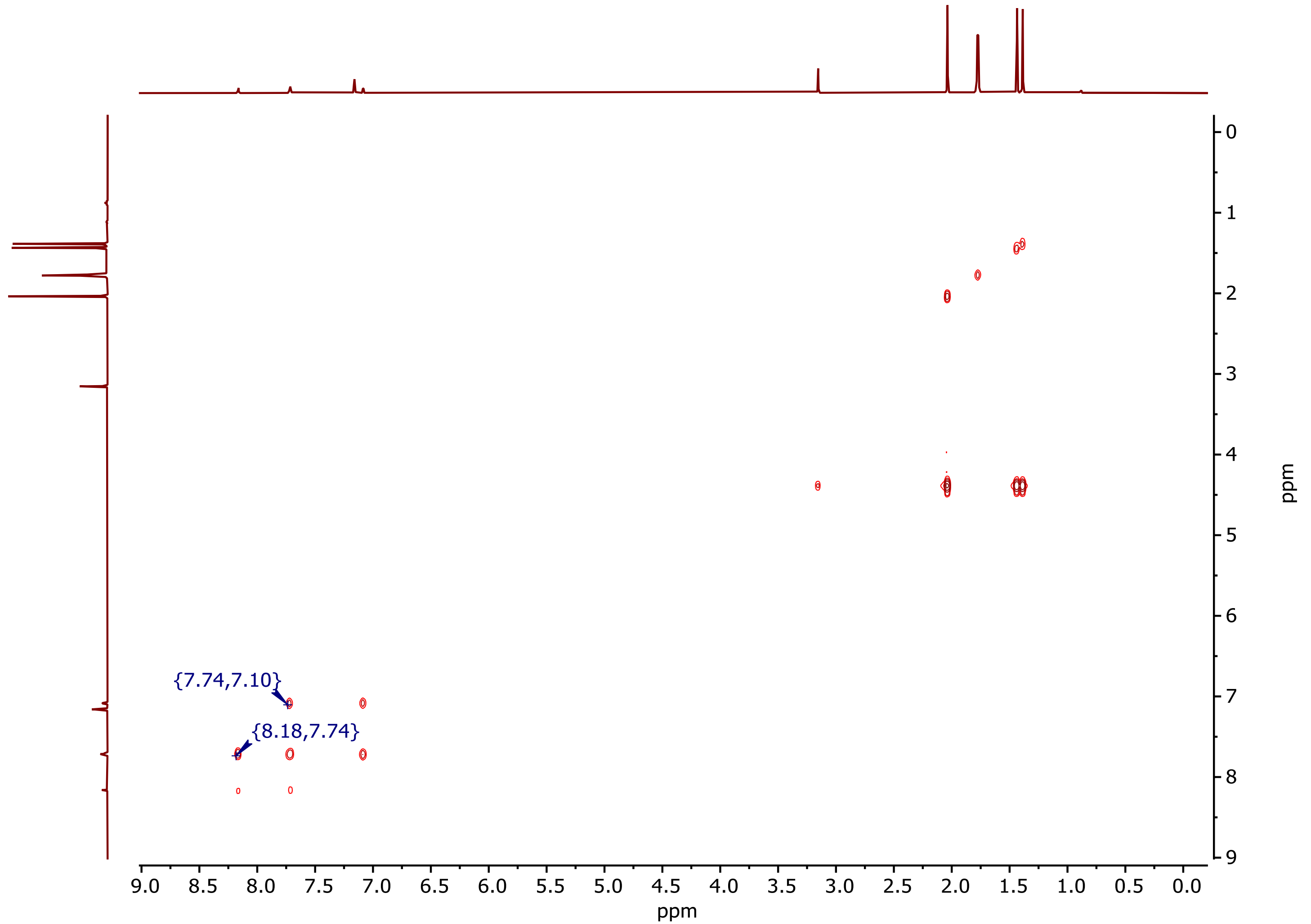

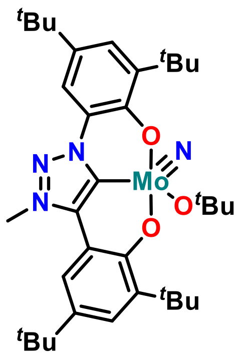

*Figure S 21: ¹H – ¹H COSY of **1-OᵗBu** in C_6D_6 at 298 K.*

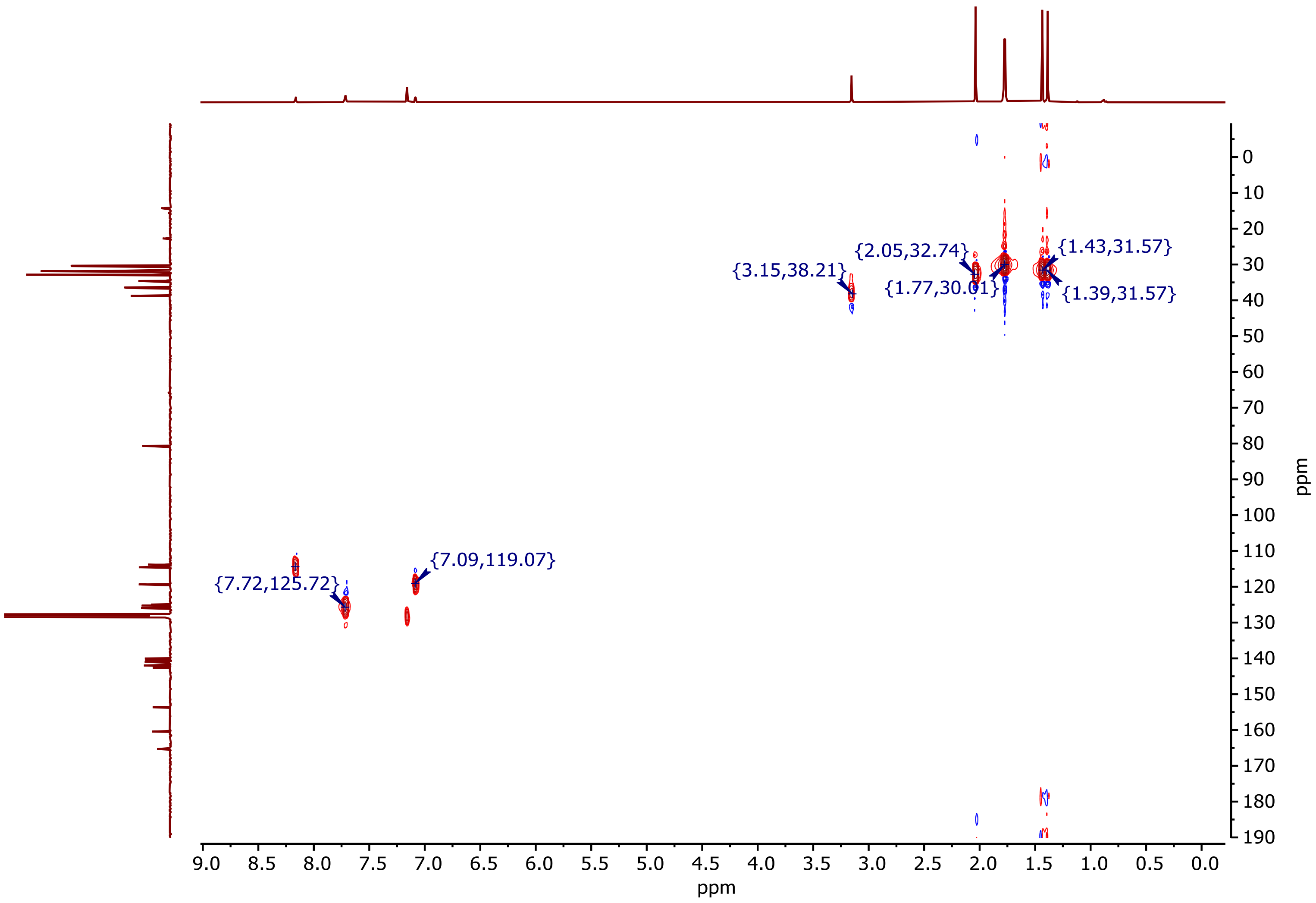

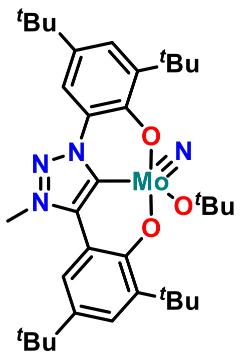

*Figure S 22: ^{1}H – ^{13}C HSQC of **1-O^{t}Bu** in C_6D_6 at 298 K.*

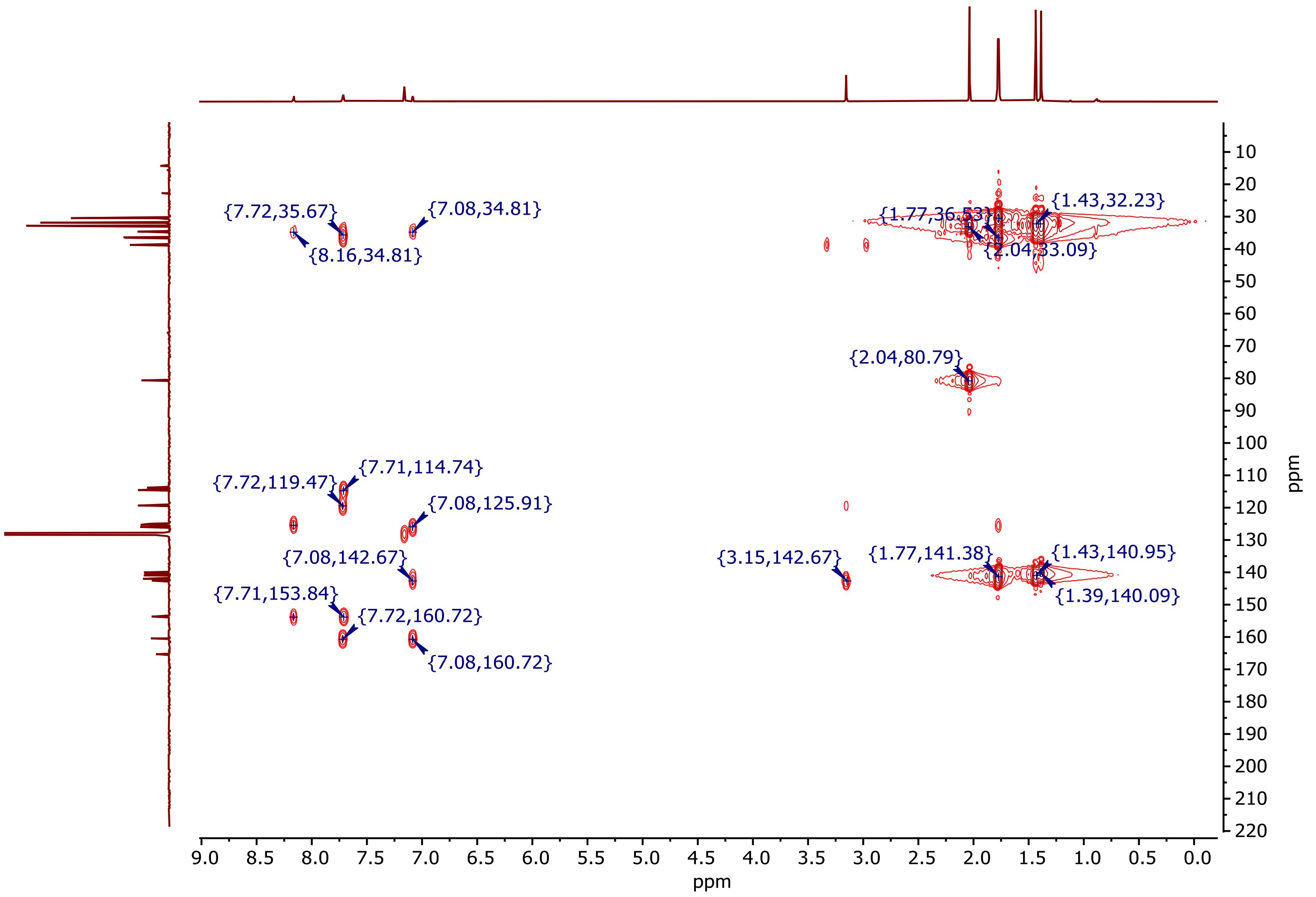

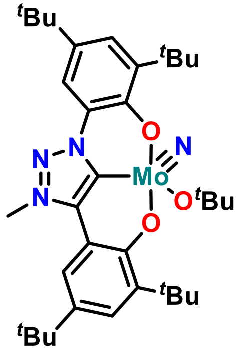

*Figure S 23: ^{1}H – ^{13}C HMBC of **1-O^{t}Bu** in C_6D_6 at 298 K.*

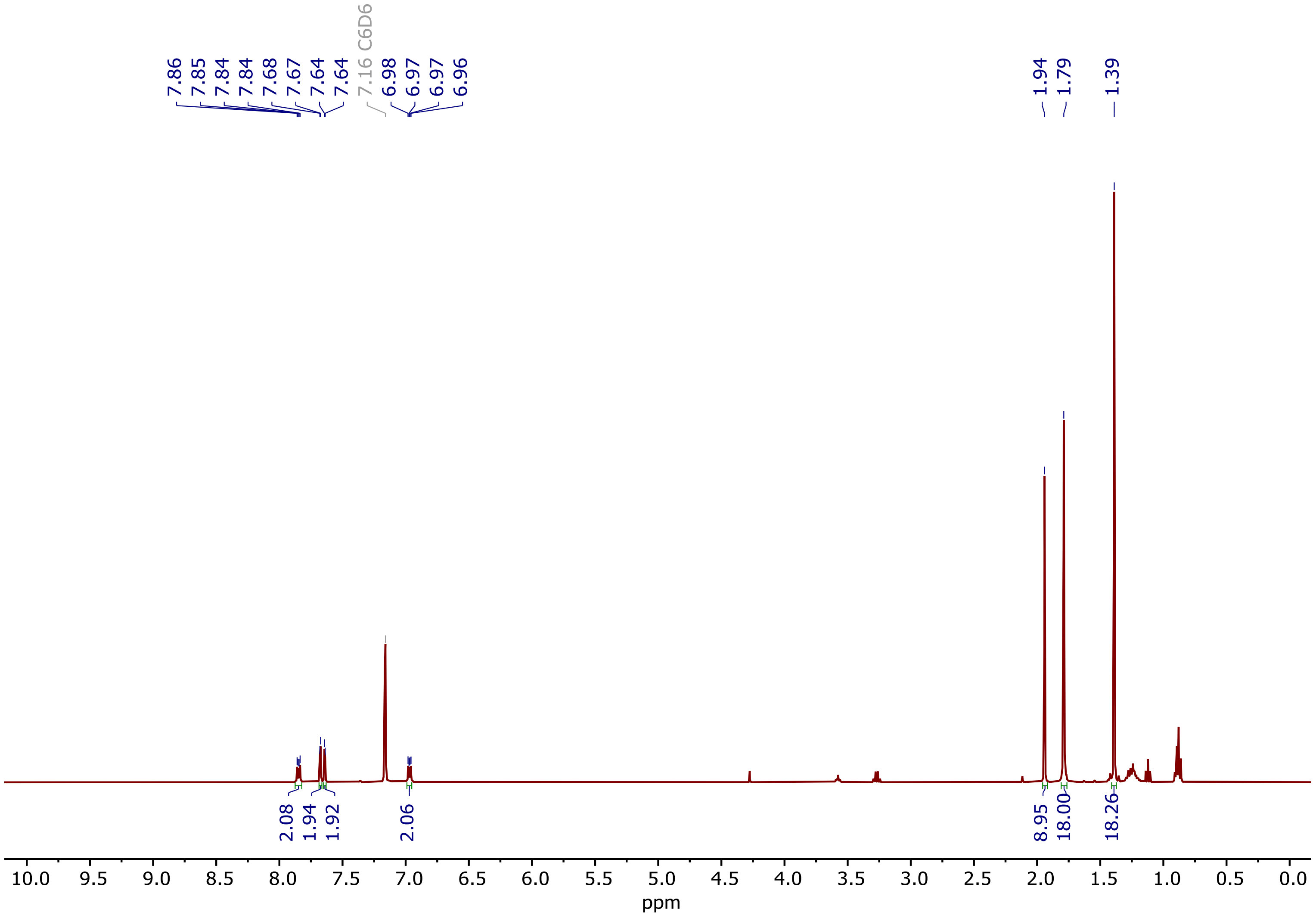

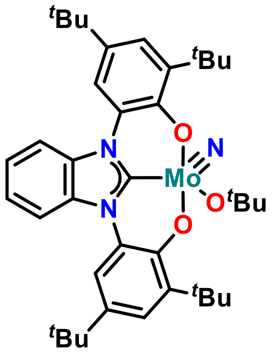

*Figure S 24: ^{1}H NMR of **2-O^{t}Bu** in C_6D_6 at 298 K.*

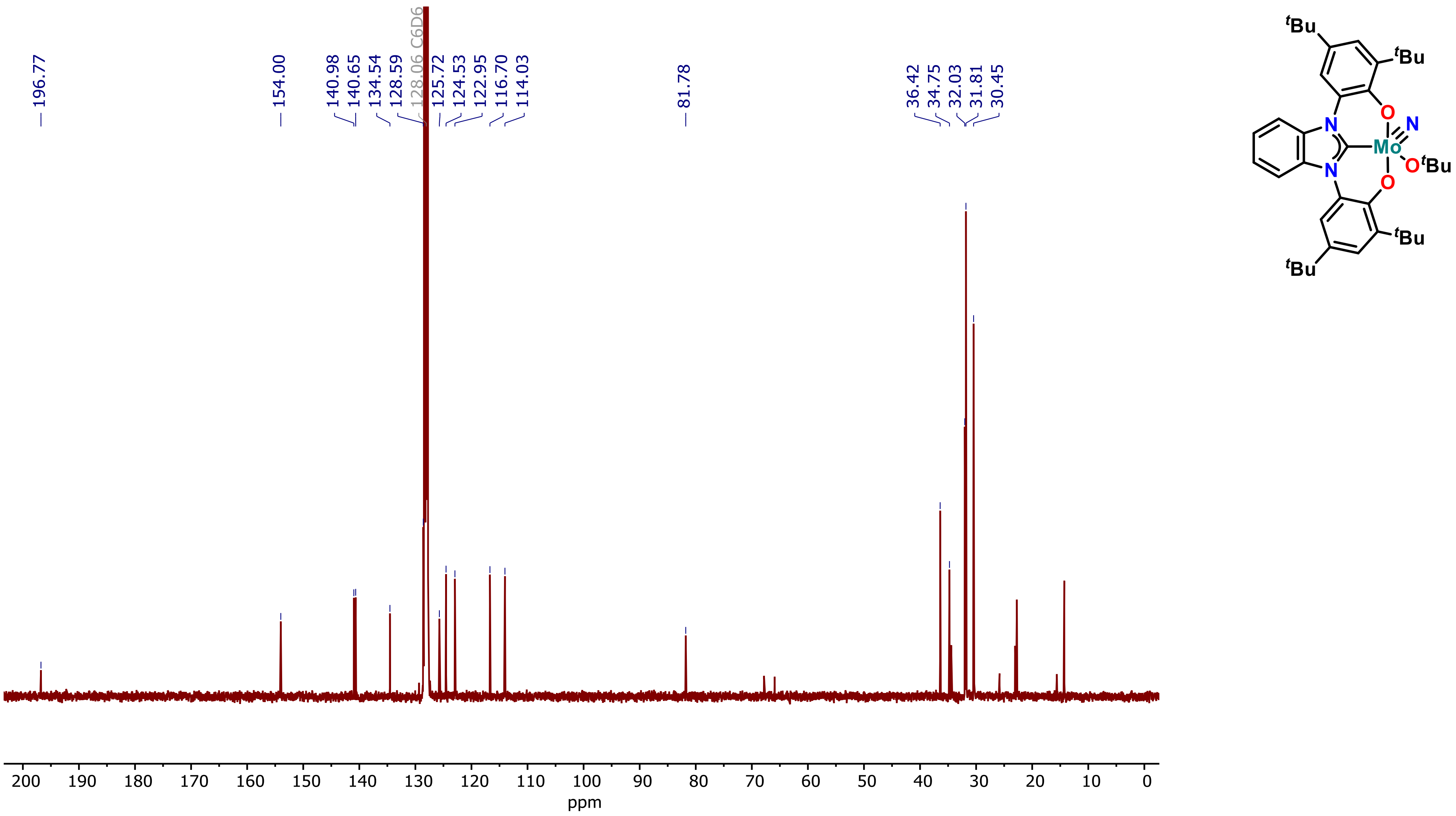

*Figure S 25: ^{13}C NMR of **2-O^{t}Bu** in C_6D_6 at 298 K.*

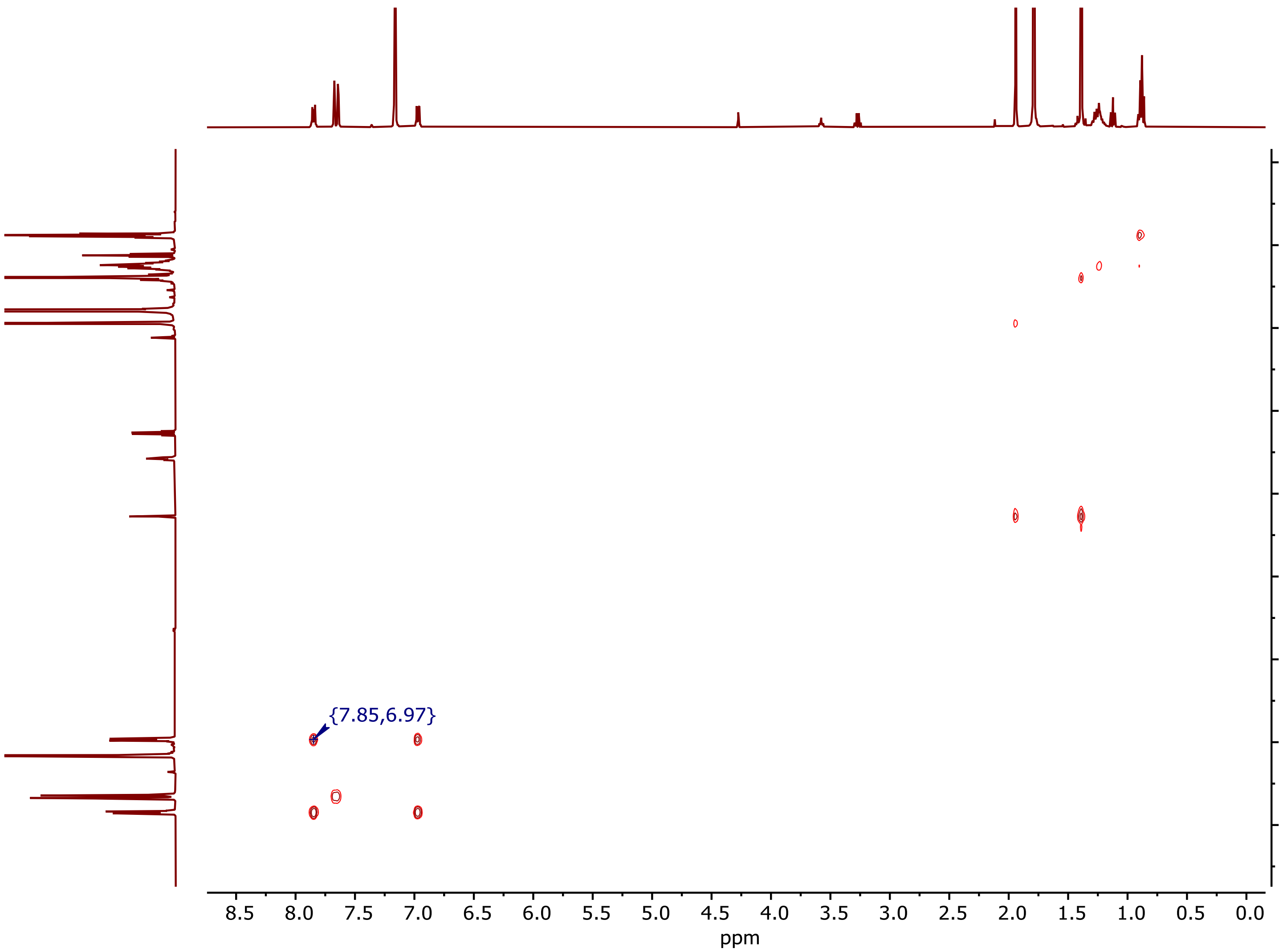

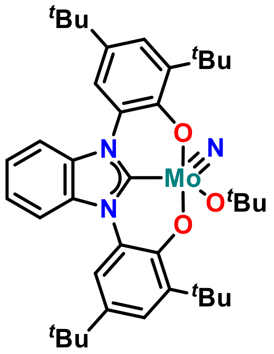

*Figure S 26: ¹H – ¹H COSY of **2-OᵗBu** in C_6D_6 at 298 K.*

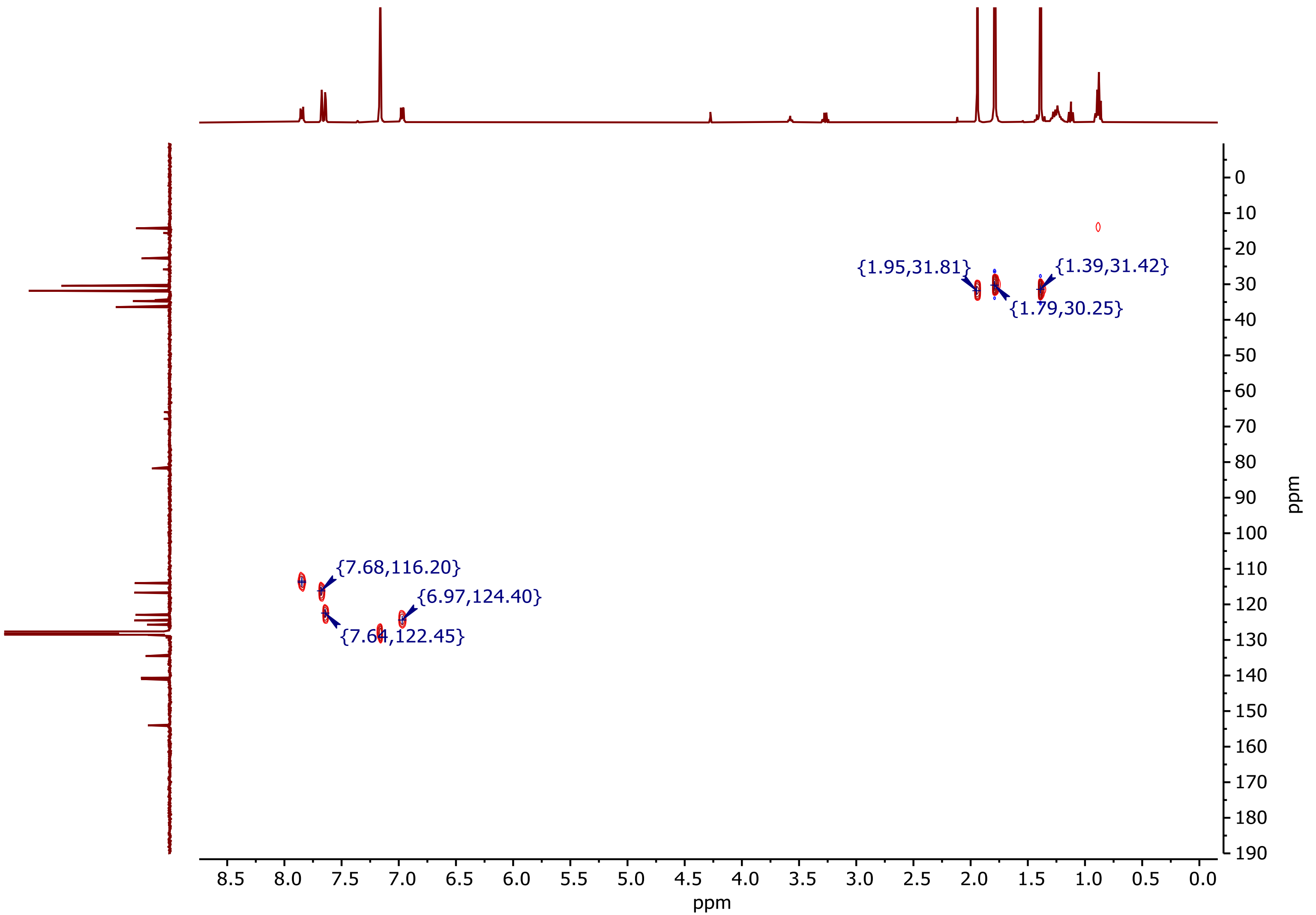

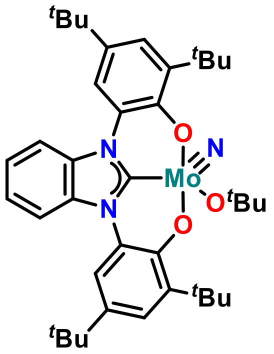

*Figure S 27: ^{1}H – ^{13}C HSQC of **2-O^{t}Bu** in C_6D_6 at 298 K.*

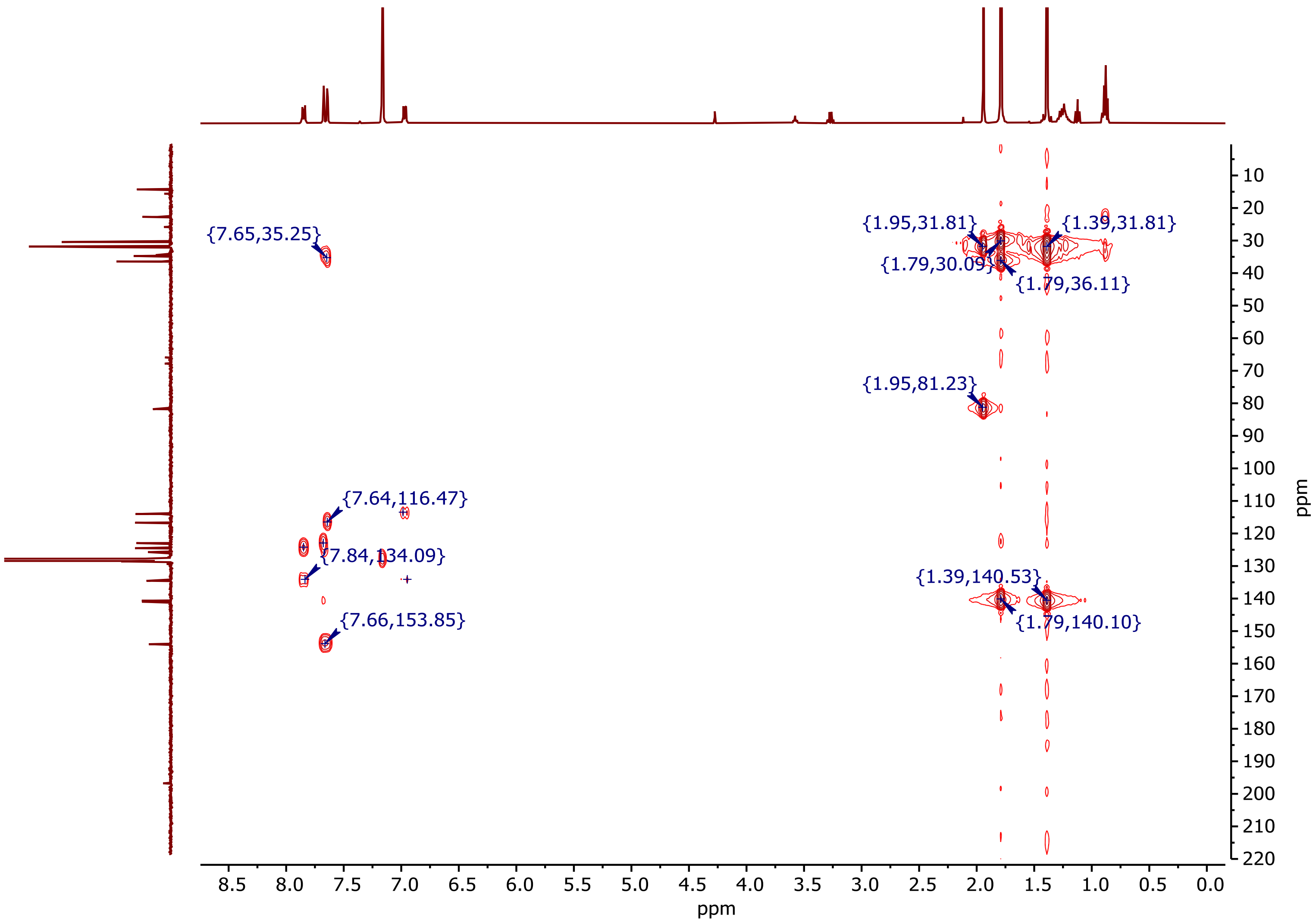

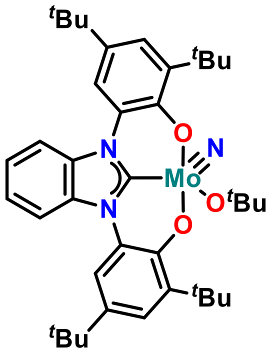

*Figure S 28: ¹H – ¹³C HMBC of **2-OᵗBu** in C_6D_6 at 298 K.*

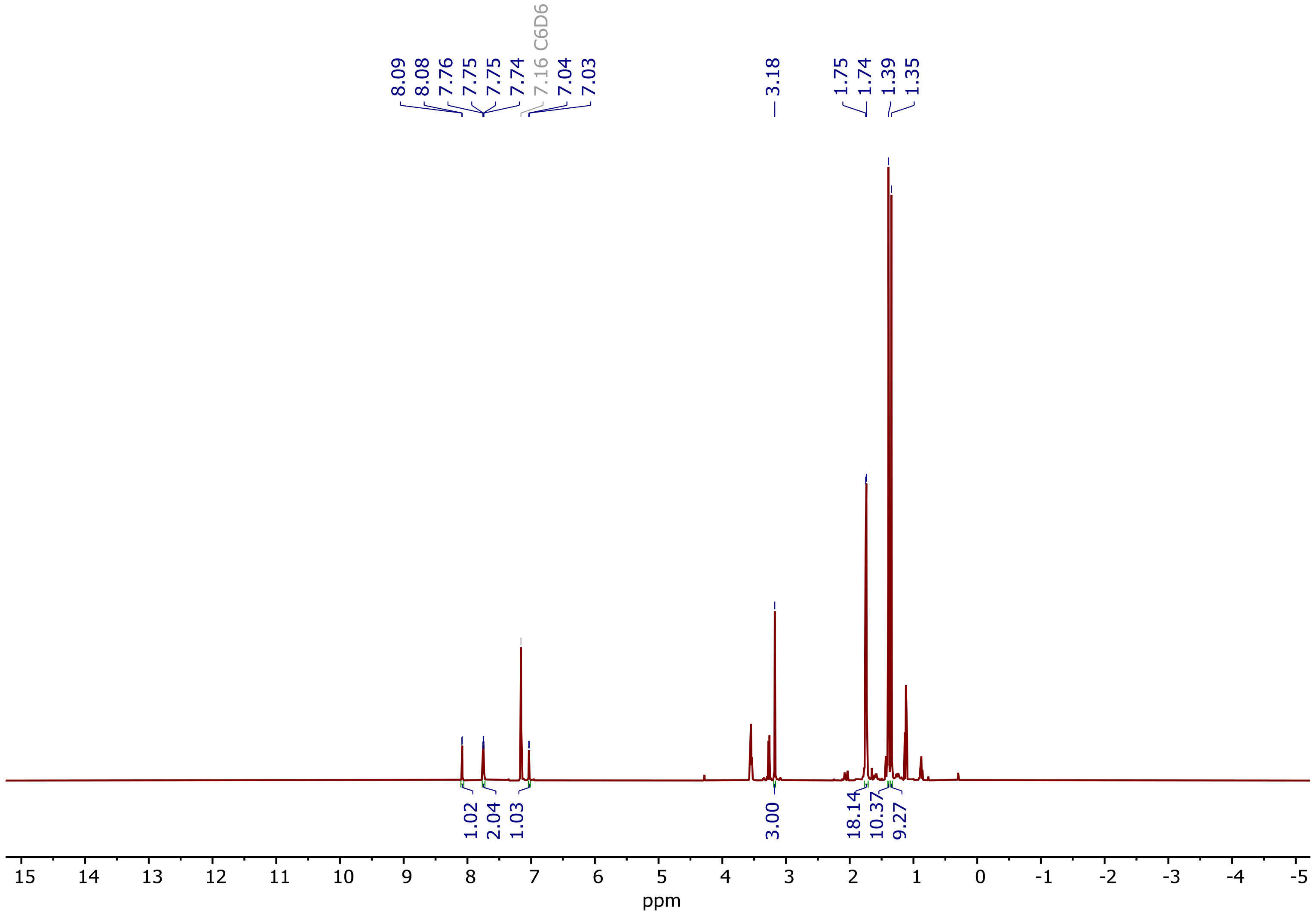

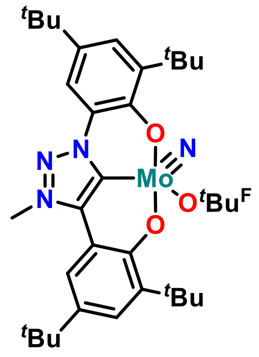

*Figure S 29: ^{1}H NMR of **1-O^{t}Bu^{F9}** in C_6D_6 at 298 K.*

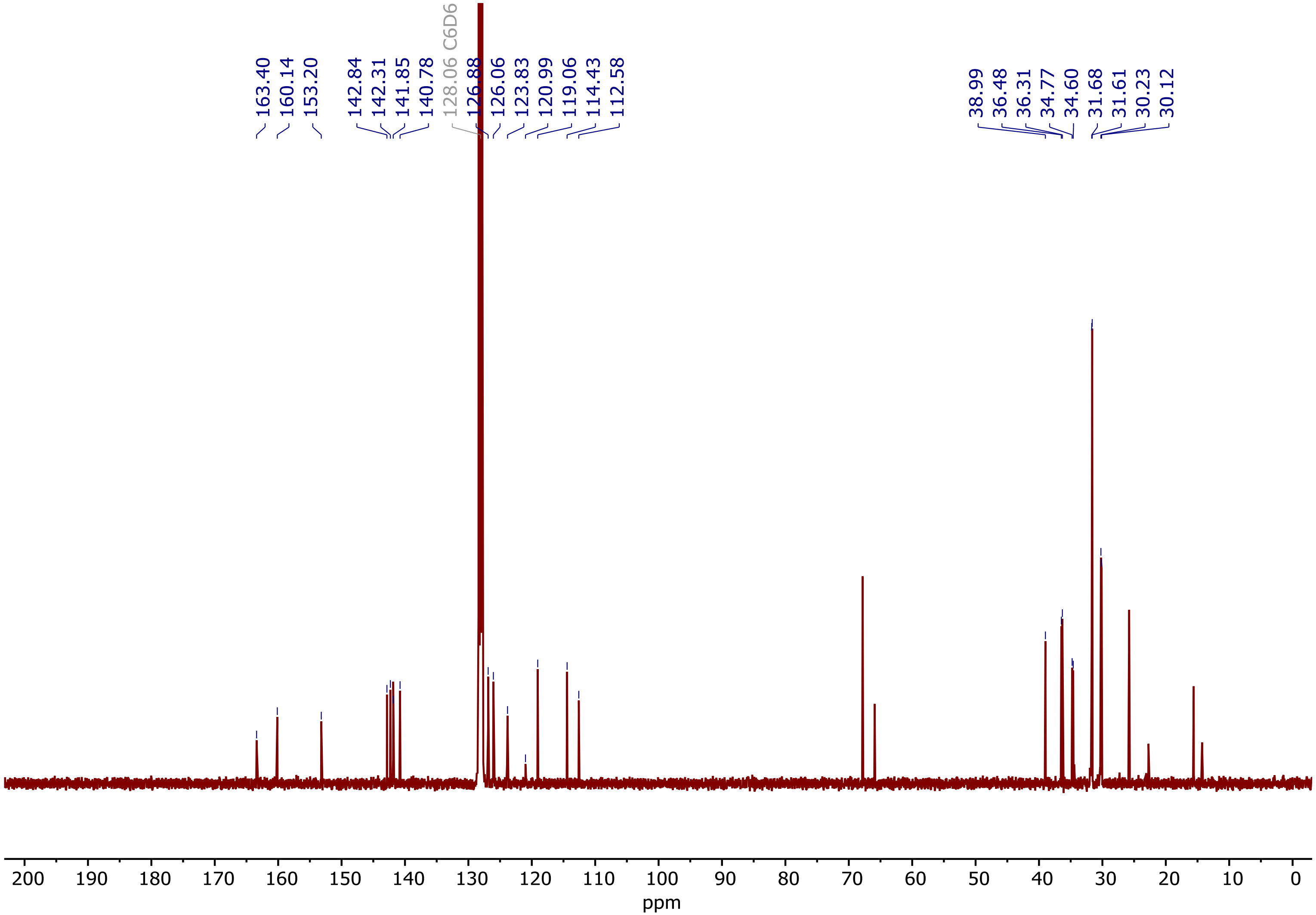

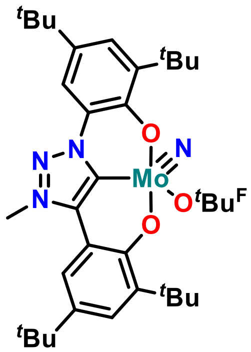

*Figure S 30: ¹³C NMR of **1-O^{t}Bu^{F9}** in C_6D_6 at 298 K.*

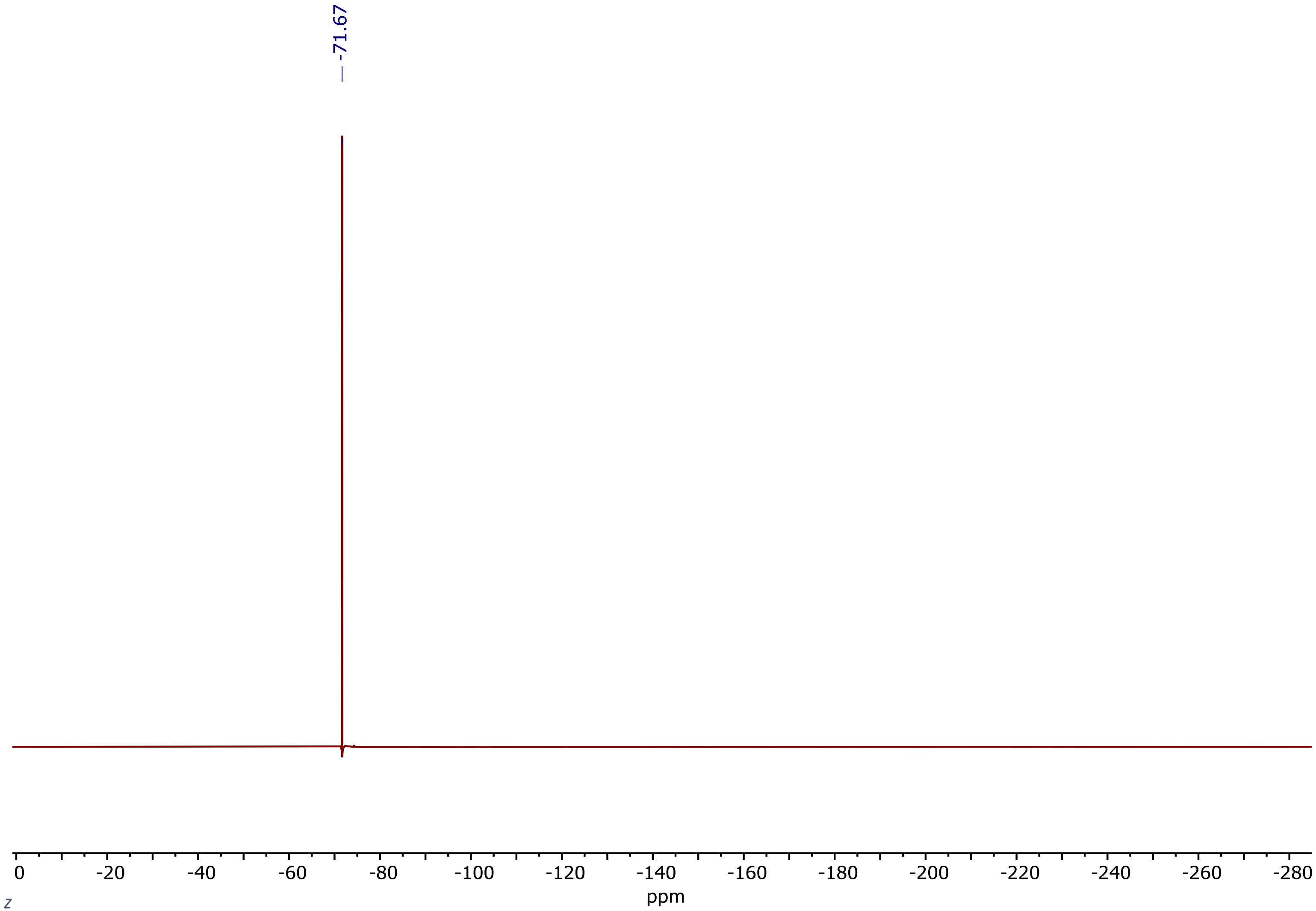

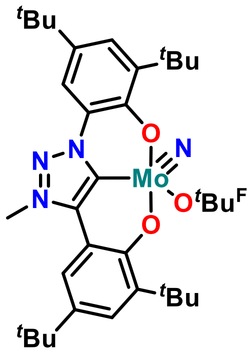

z

*Figure S 31: ^{19}F NMR of **1-O^{t}Bu^{F9}** in C_6D_6 at 298 K.*

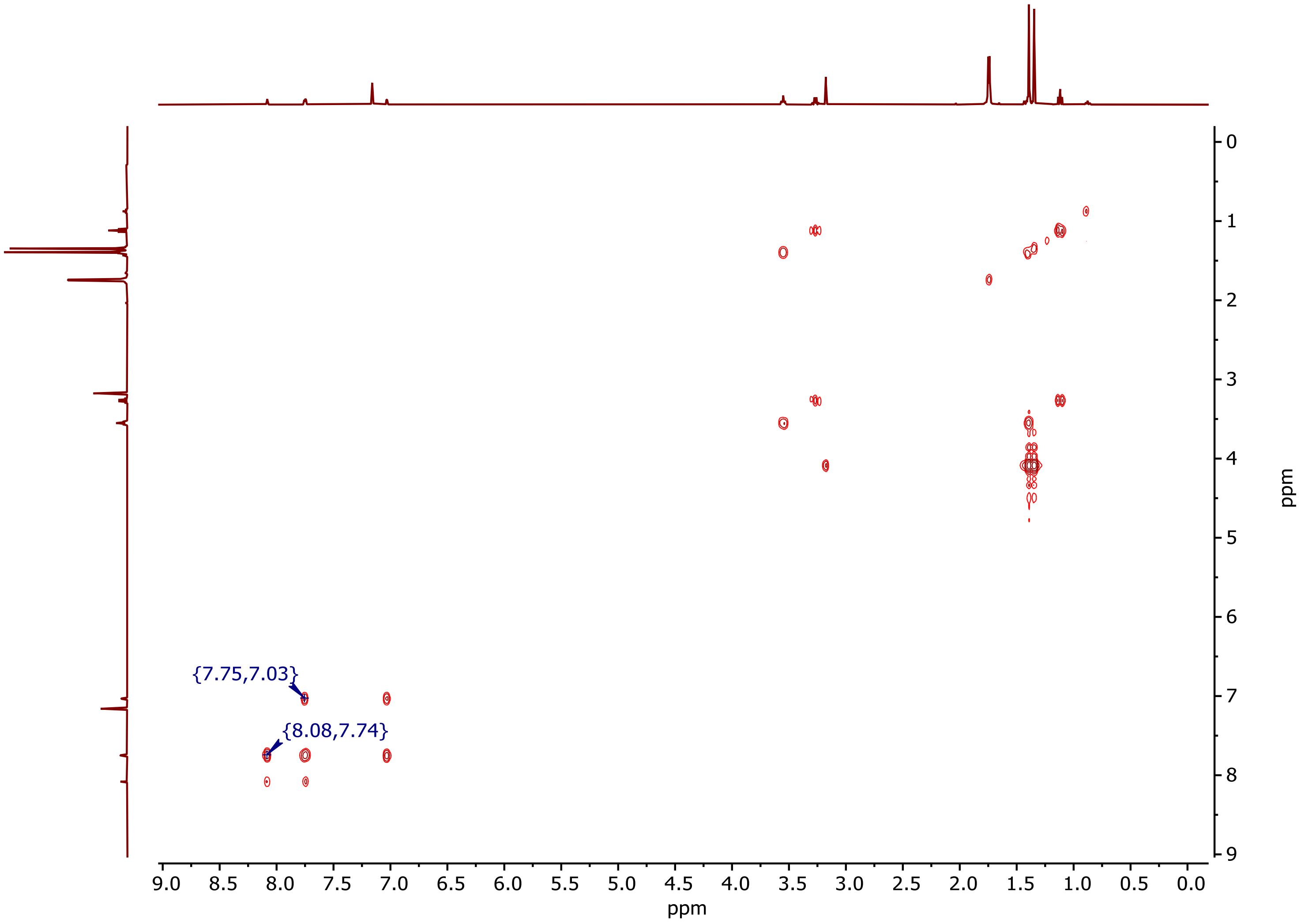

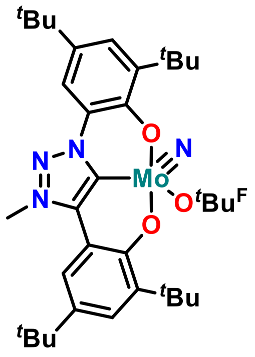

*Figure S 32: ^{1}H – ^{1}H COSY of **1-O^{t}Bu^{F9}** in C_6D_6 at 298 K.*

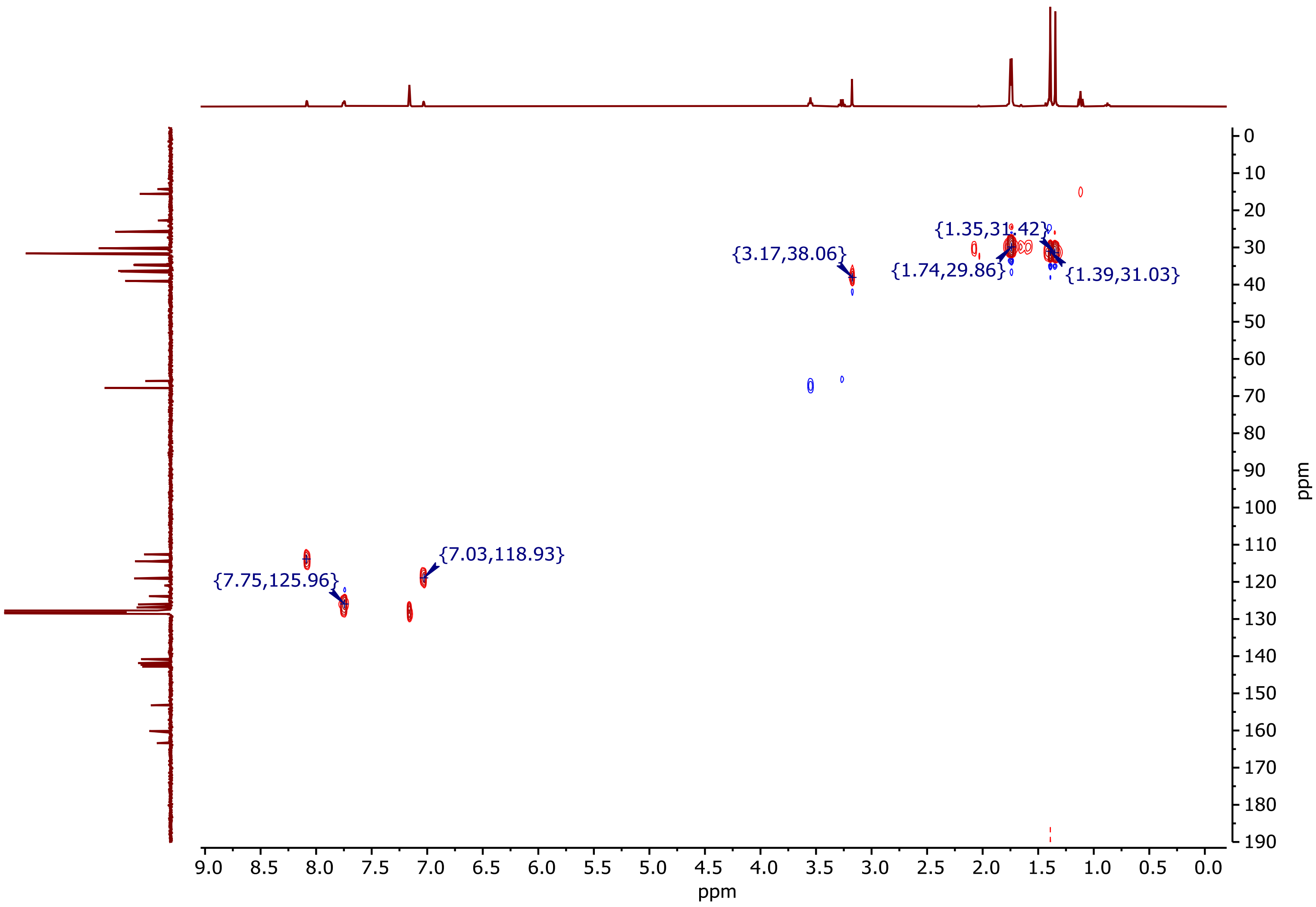

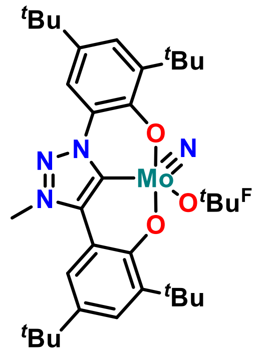

*Figure S 33: $^{1}H – ^{13}C$ HSQC of **1-O^{t}Bu^{F9}** in C_6D_6 at 298 K.*

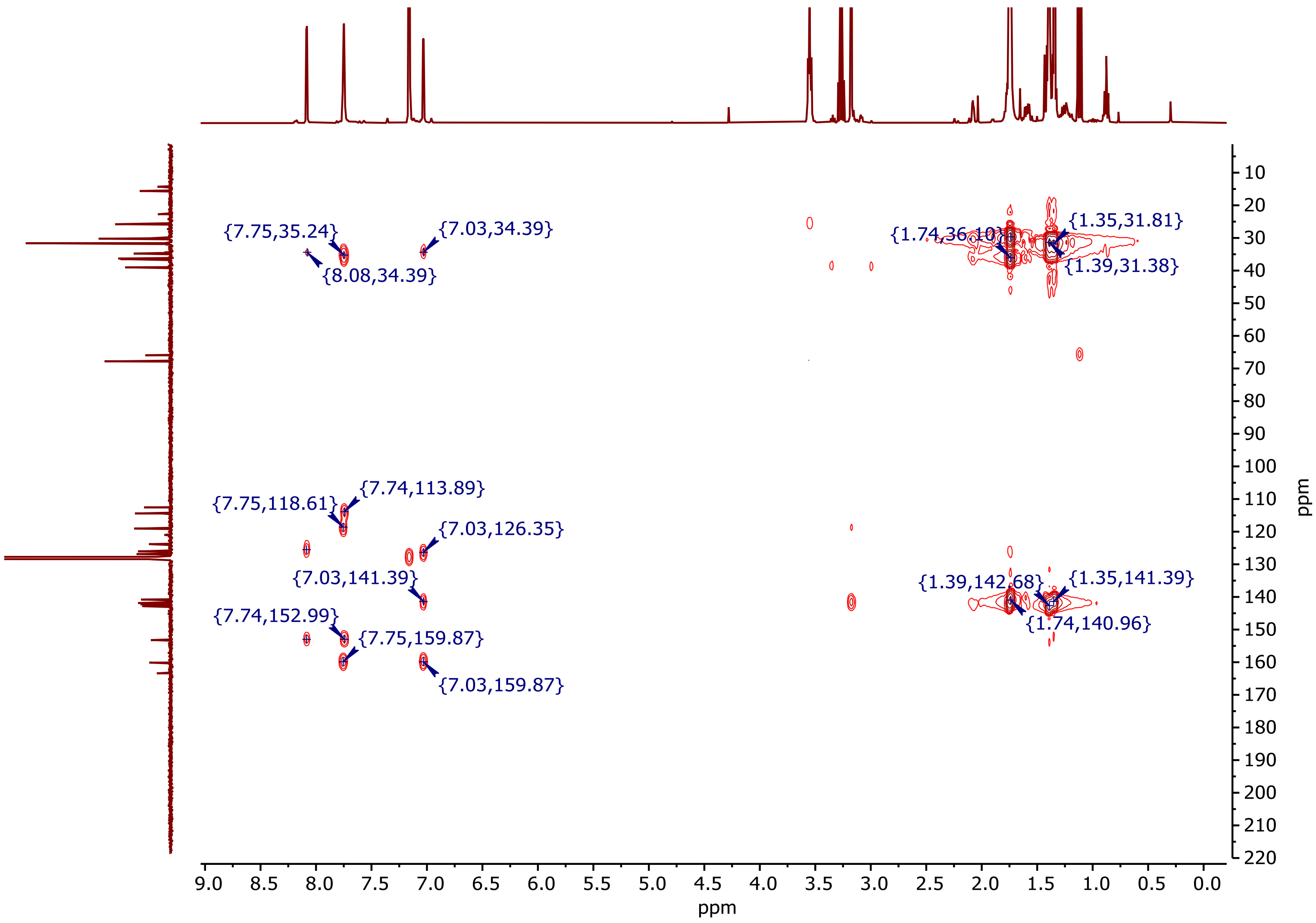

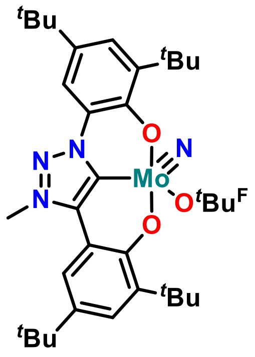

*Figure S 34: $^{1}H - ^{13}C$ HMBC of **1-O^{t}Bu^{F9}** in C_6D_6 at 298 K.*

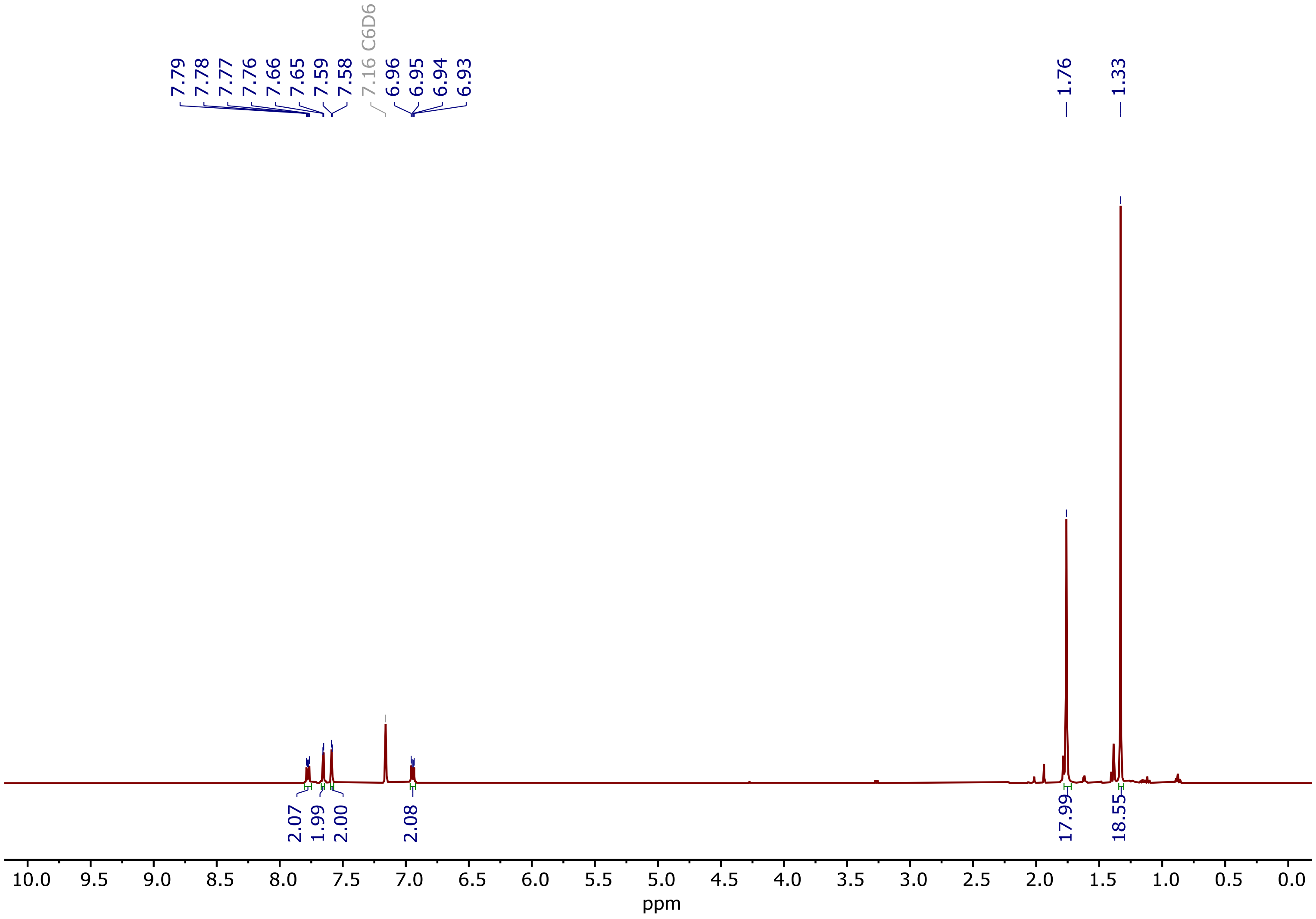

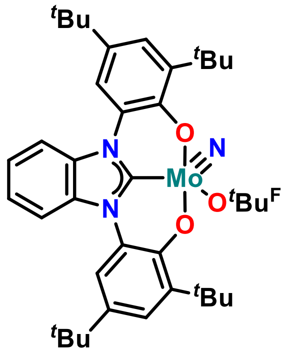

*Figure S 35: ^{1}H NMR of **2-O^{t}Bu^{F9}** in C_6D_6 at 298 K.*

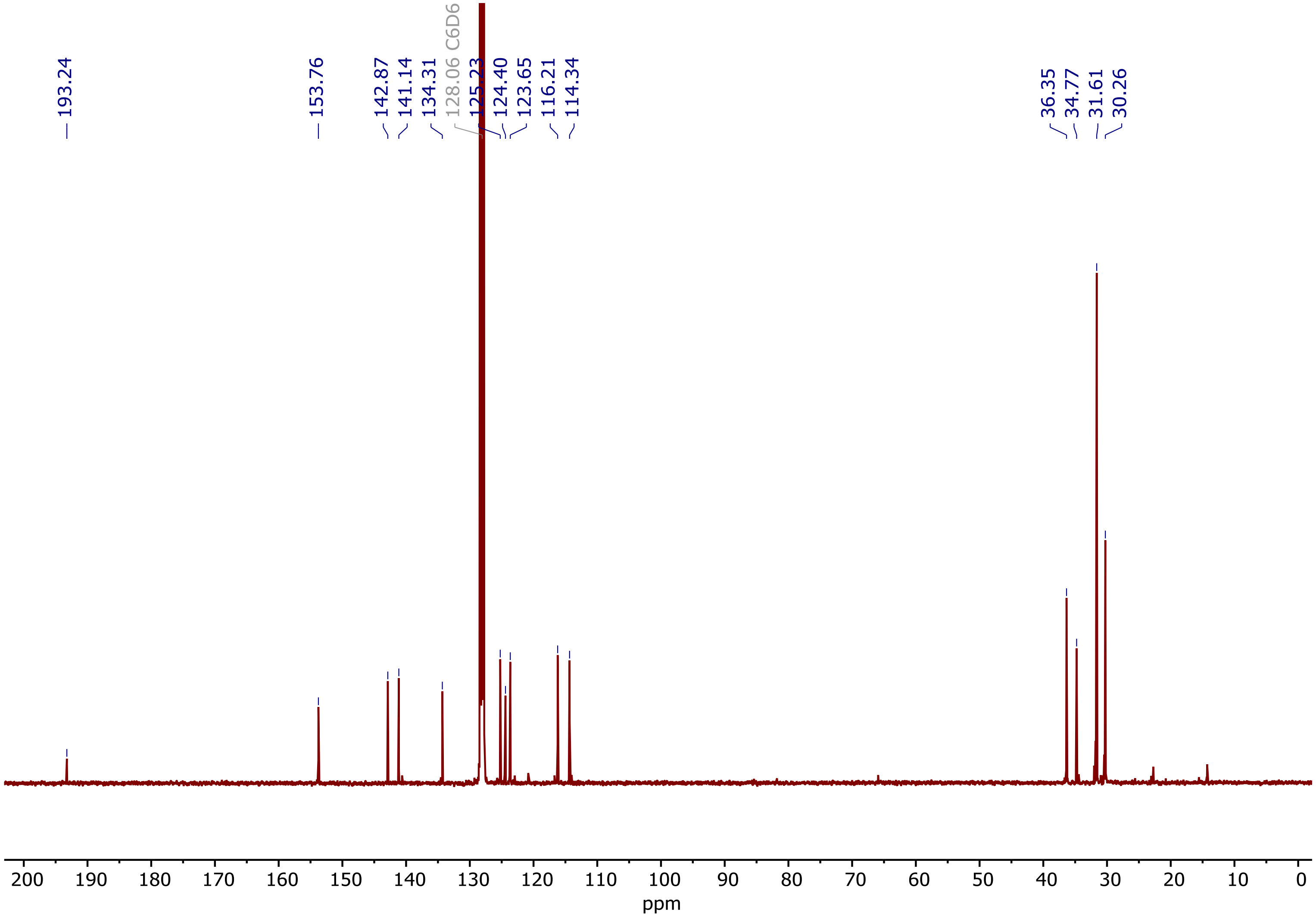

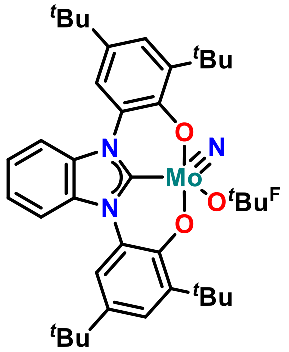

*Figure S 36: ^{13}C NMR of **2-O^{t}Bu^{F9}** in C_6D_6 at 298 K.*

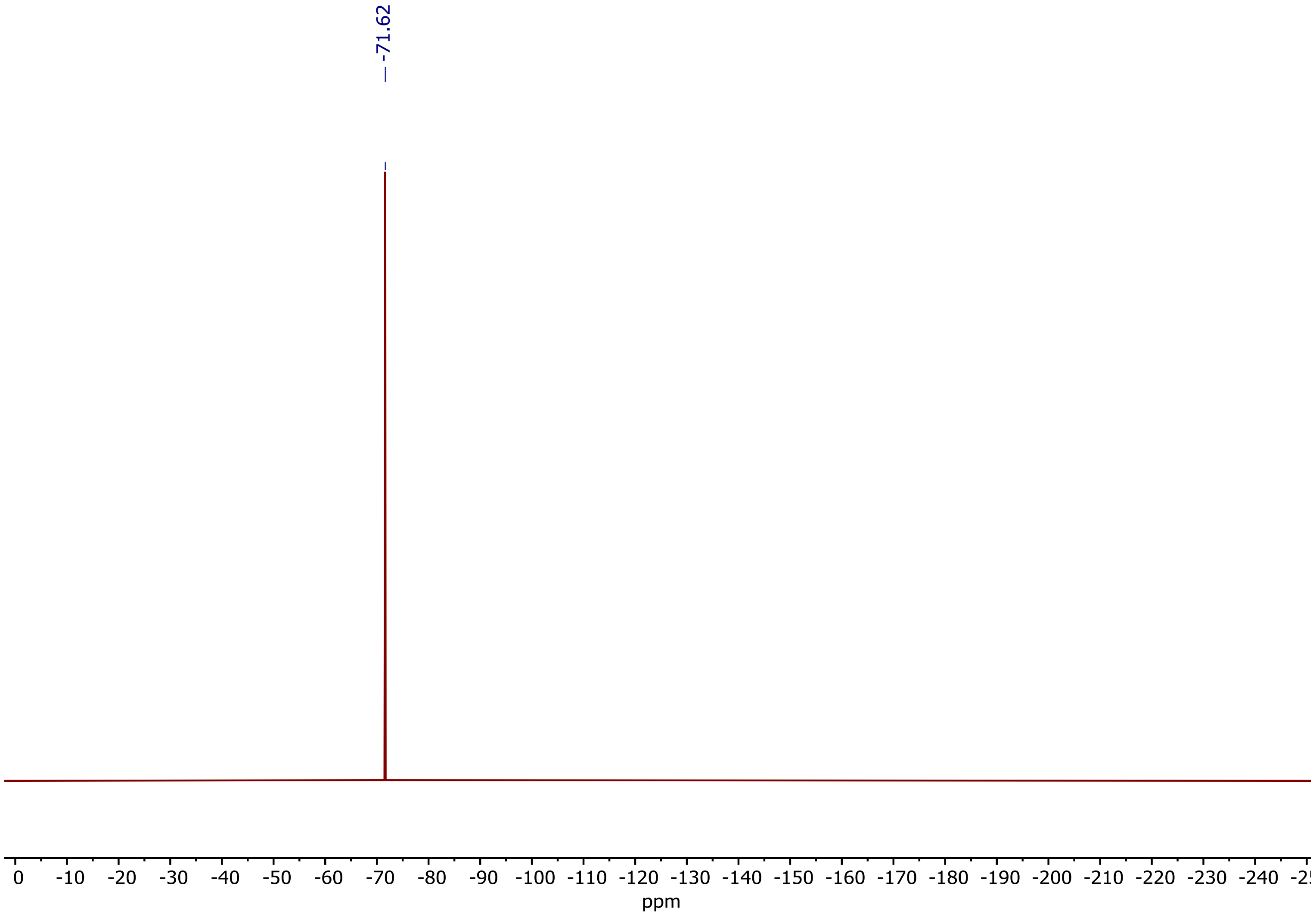

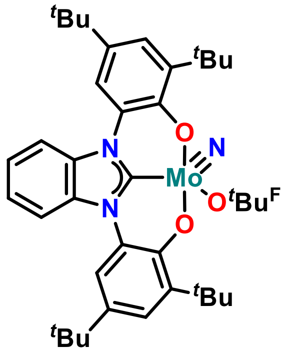

*Figure S 37: ^{19}F NMR of **2-O^{t}Bu^{F9}** in C_6D_6 at 298 K.*

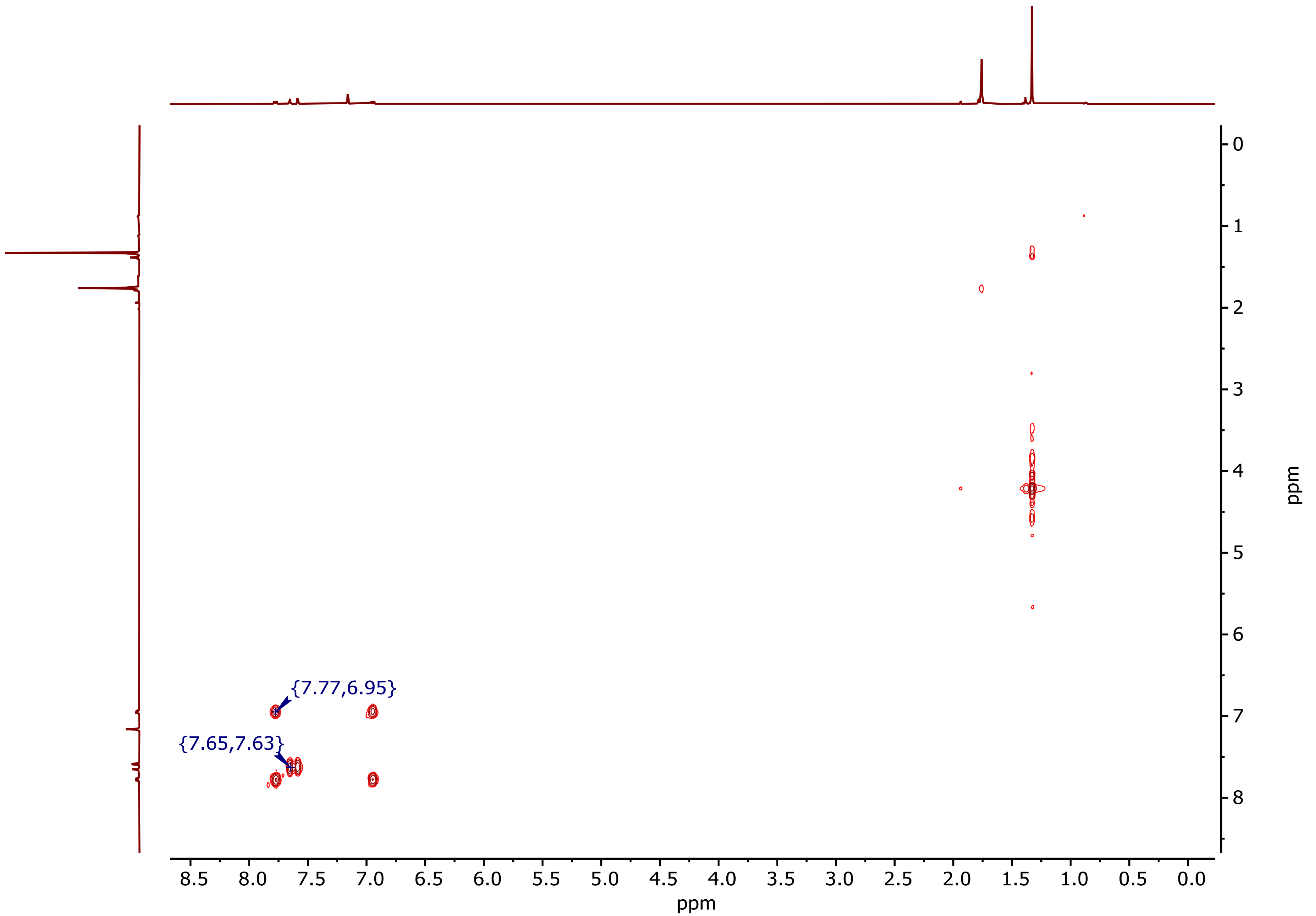

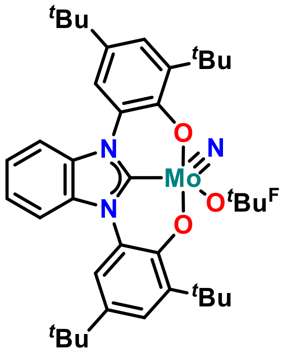

*Figure S 38: $^{1}H - ^{1}H$ COSY of **2-O^{t}Bu^{F9}** in C_6D_6 at 298 K.*

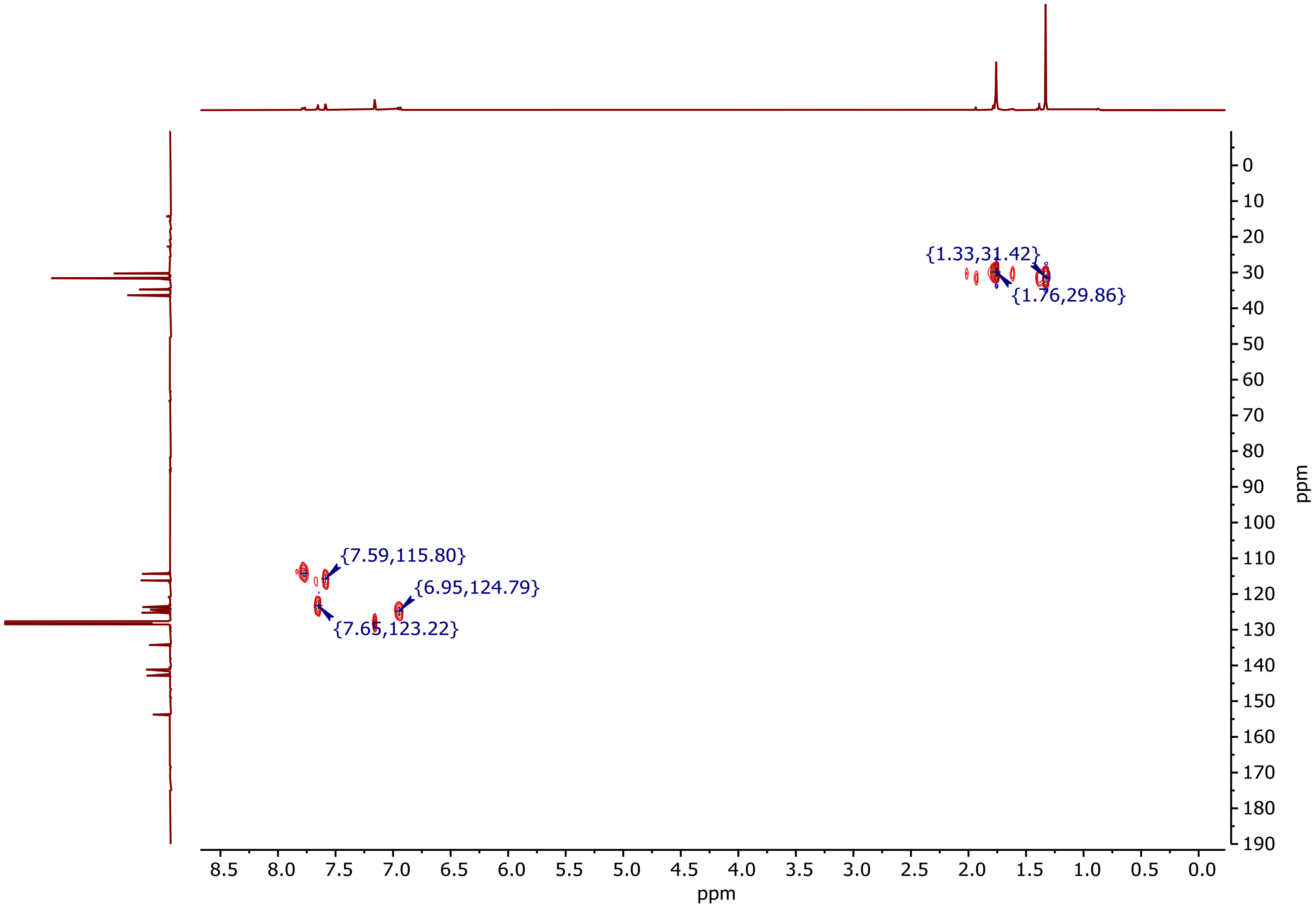

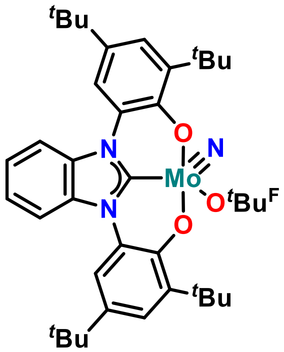

*Figure S 39: ^{1}H – ^{13}C HSQC of **2-O^{t}Bu^{F9}** in C_6D_6 at 298 K.*

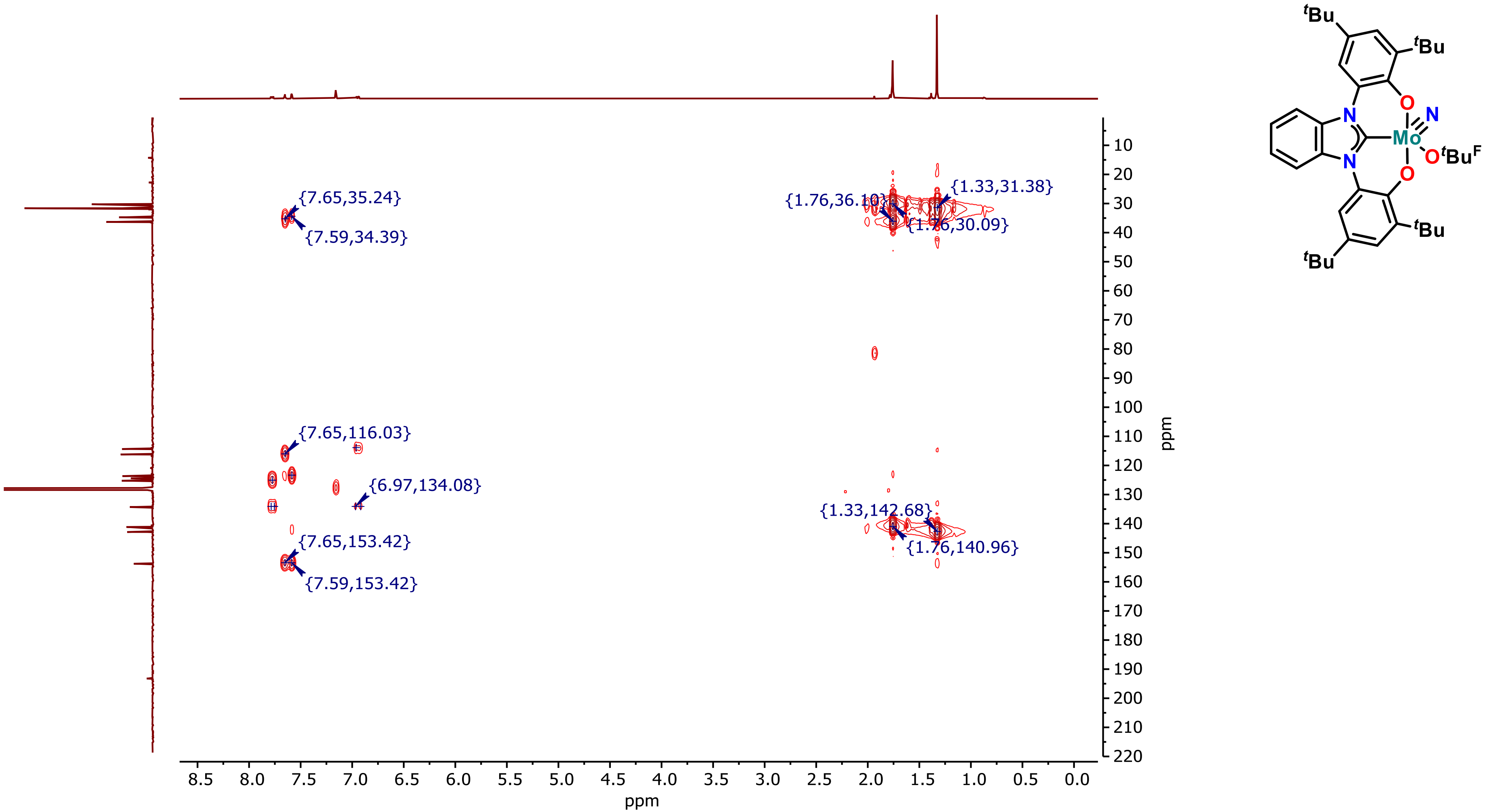

*Figure S 40: $^{1}H - ^{13}C$ HMBC of **2-O^{t}Bu^{F9}** in C_6D_6 at 298 K.*

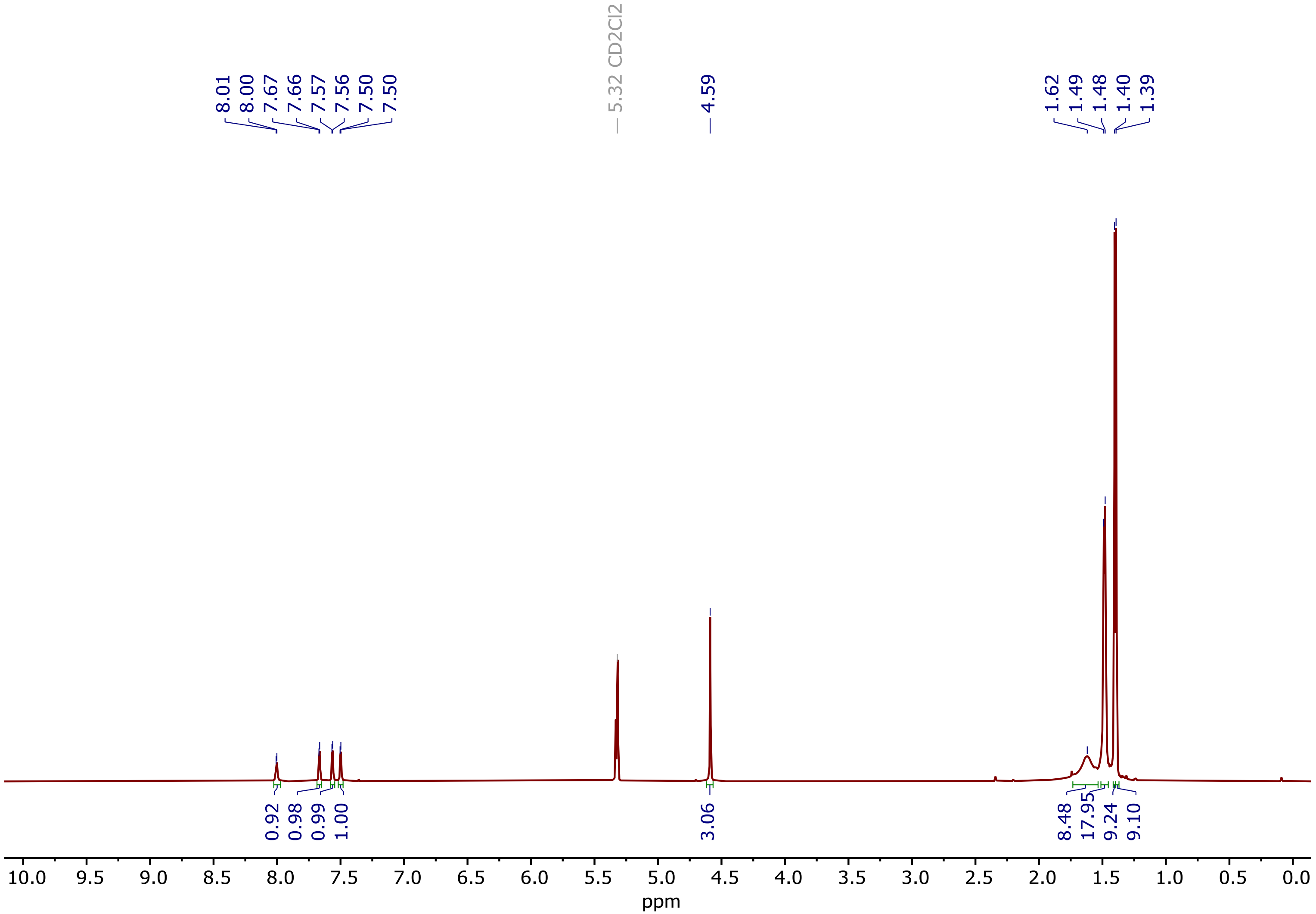

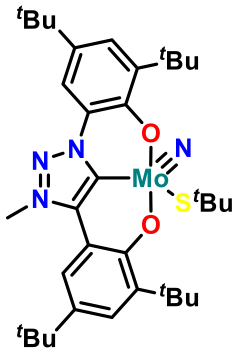

*Figure S 41: ¹H NMR of **1-S^{t}Bu** in CD_2Cl_2 at 298 K.*

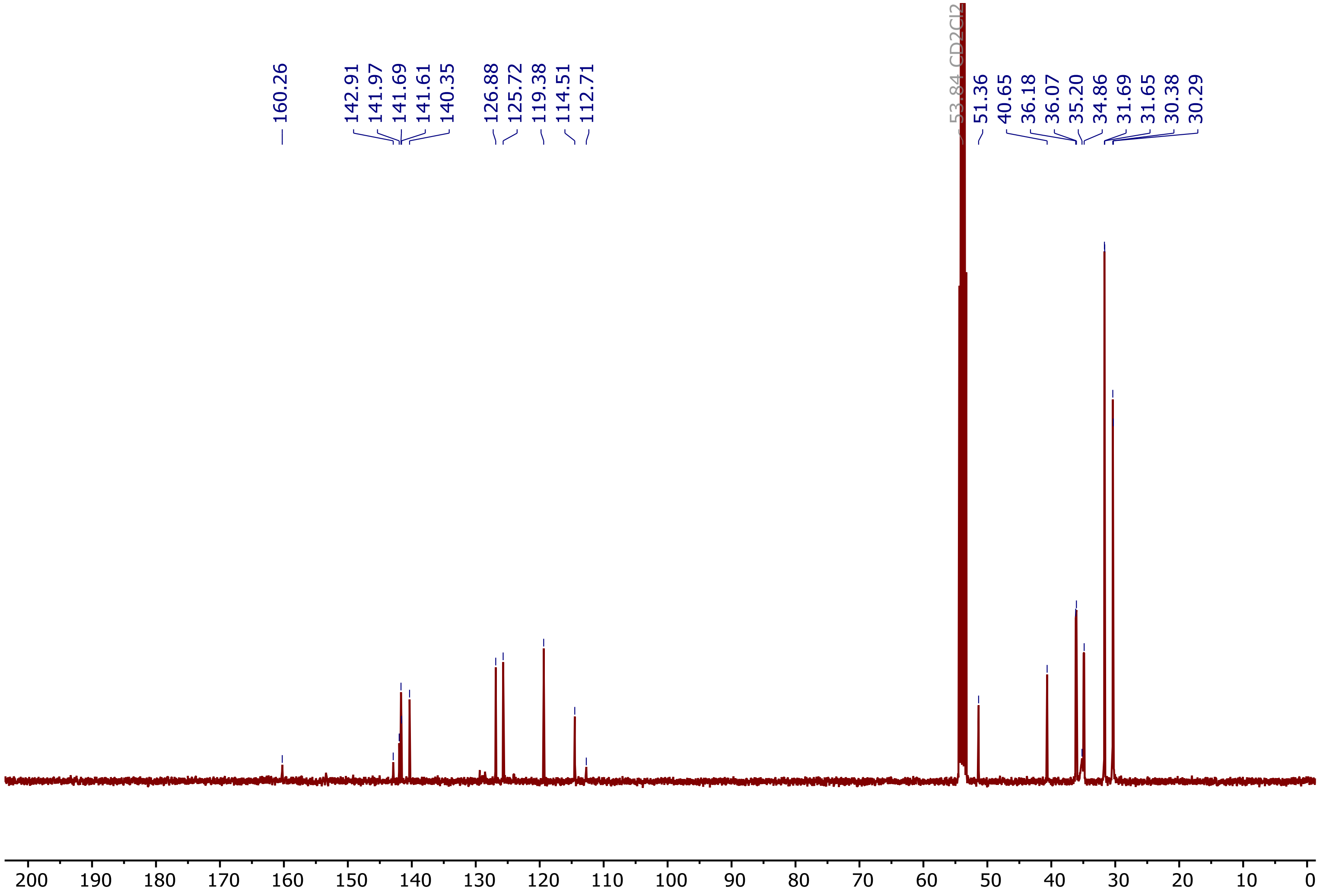

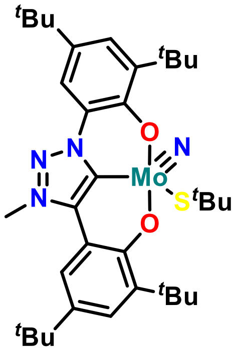

*Figure S 42: ^{13}C NMR of **1-S^{t}Bu** in CD_2Cl_2 at 298 K.*

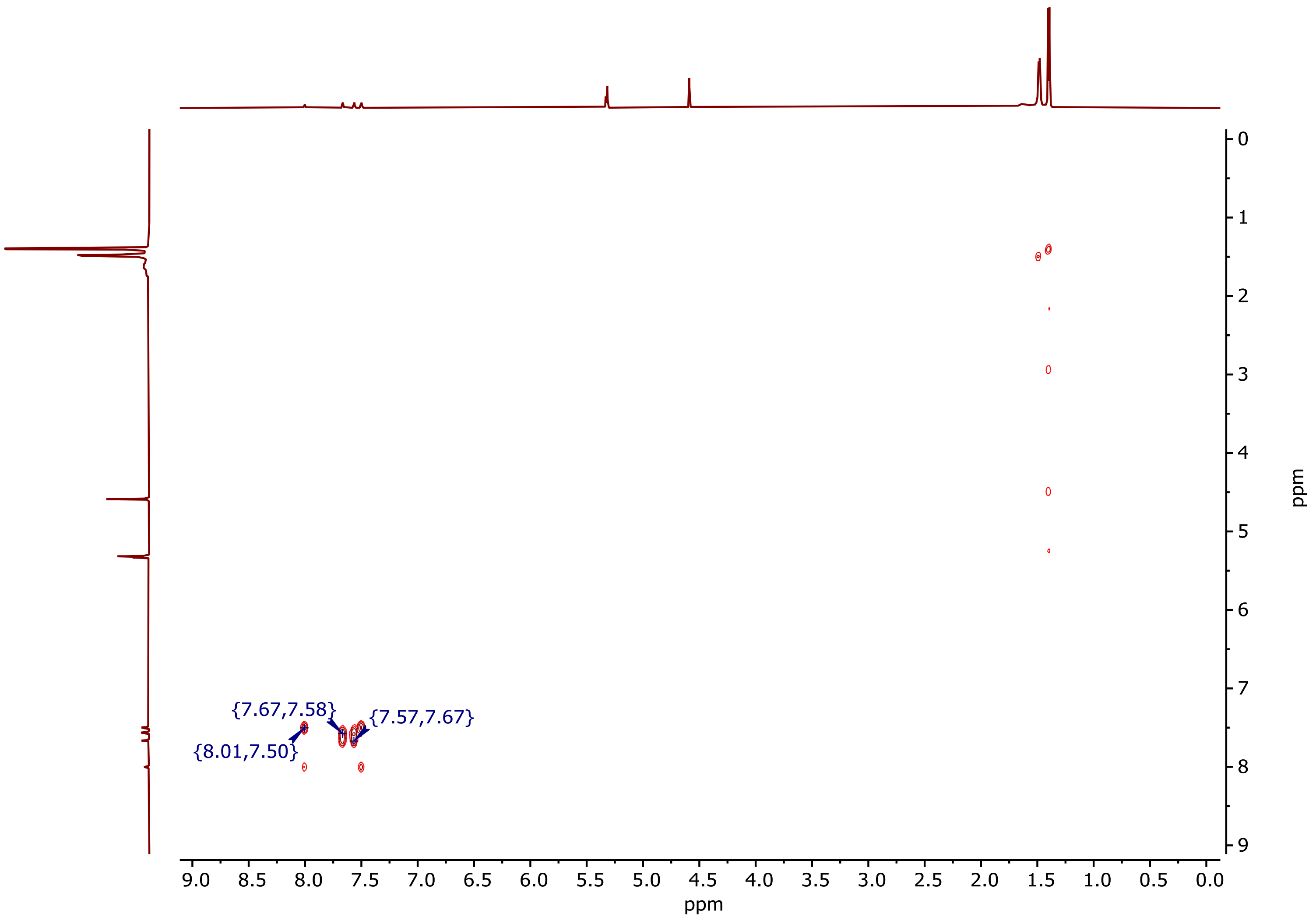

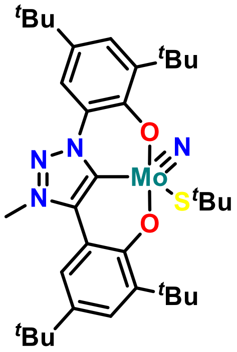

*Figure S 43: ^{1}H – ^{1}H COSY of **1-S^{t}Bu** in CD_2Cl_2 at 298 K.*

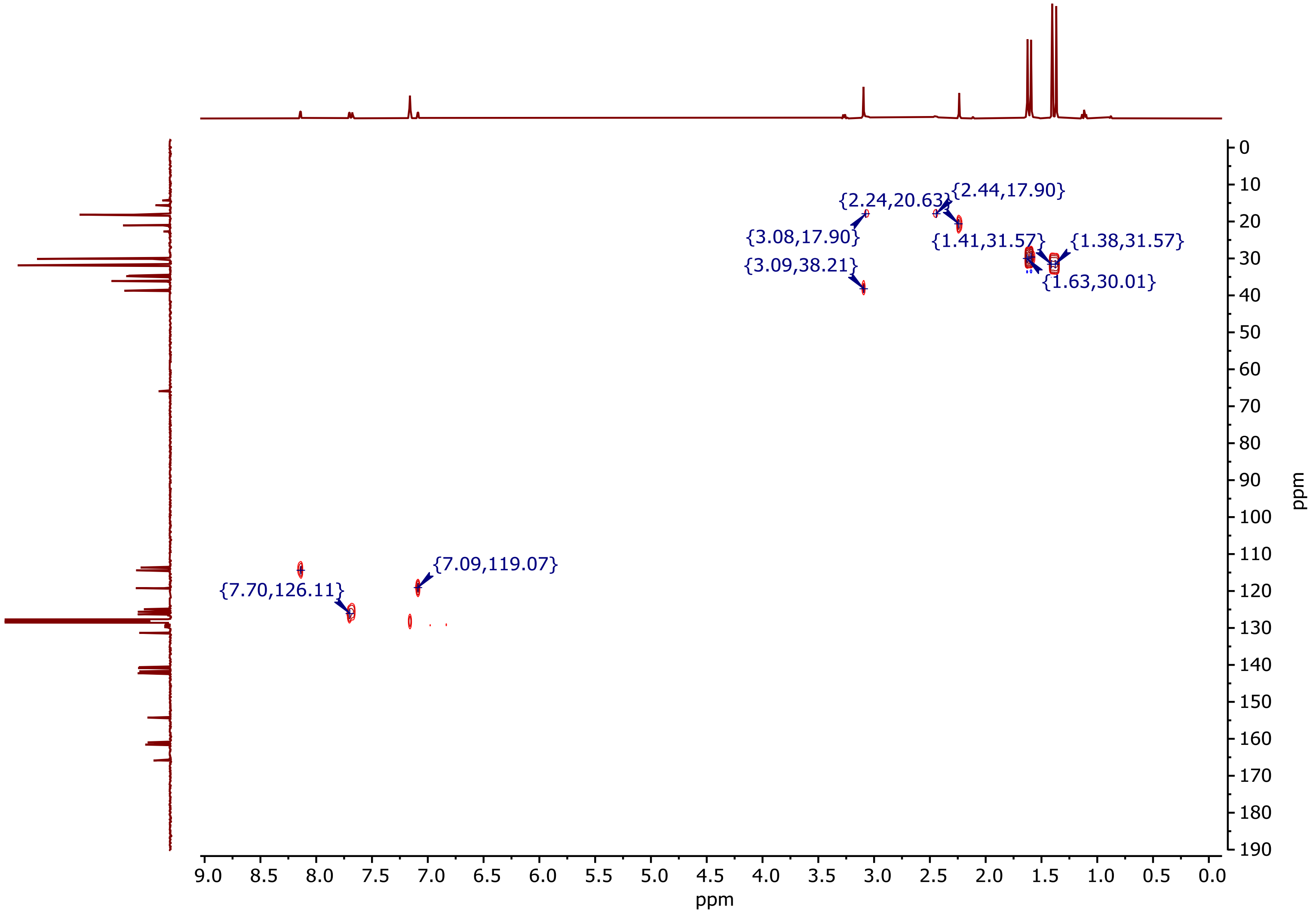

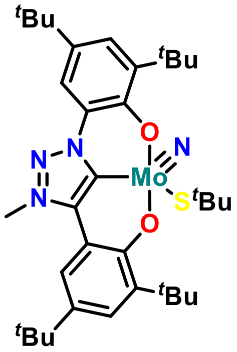

*Figure S 44: ^{1}H – ^{13}C HSQC of **1-S^{t}Bu** in CD_2Cl_2 at 298 K.*

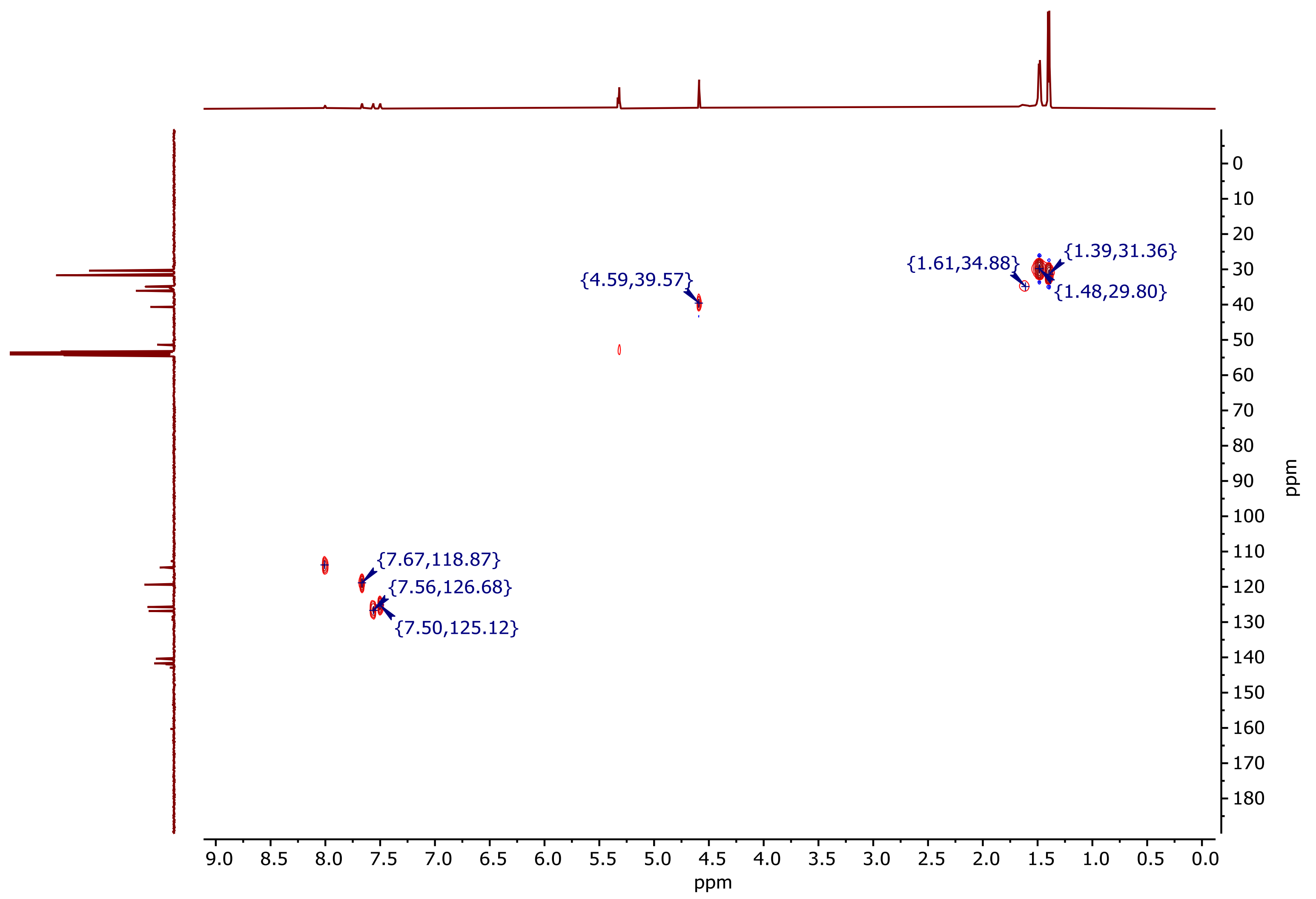

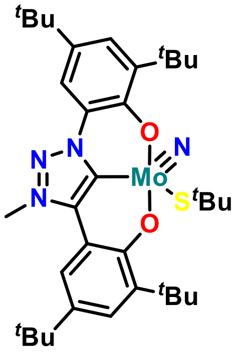

*Figure S 45: $^{1}H - ^{13}C$ HMBC of **1-S^{t}Bu** in CD_2Cl_2 at 298 K.*

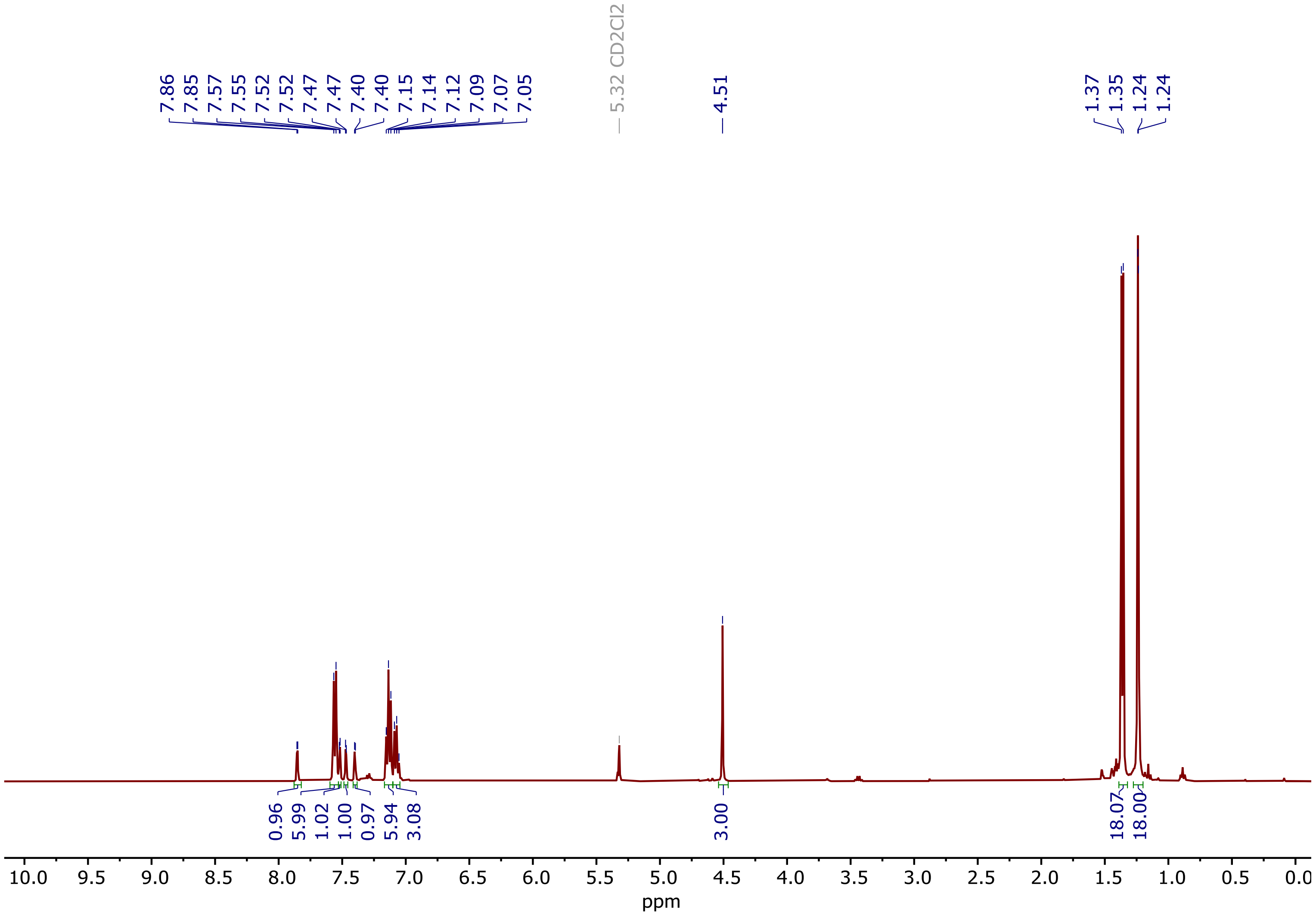

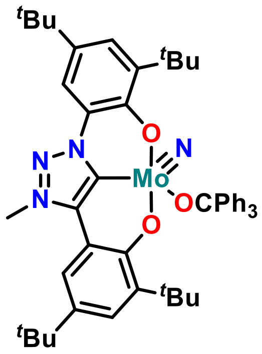

*Figure S 46: ¹H NMR of **1-OCPh₃** in CD_2Cl_2 at 298 K.*

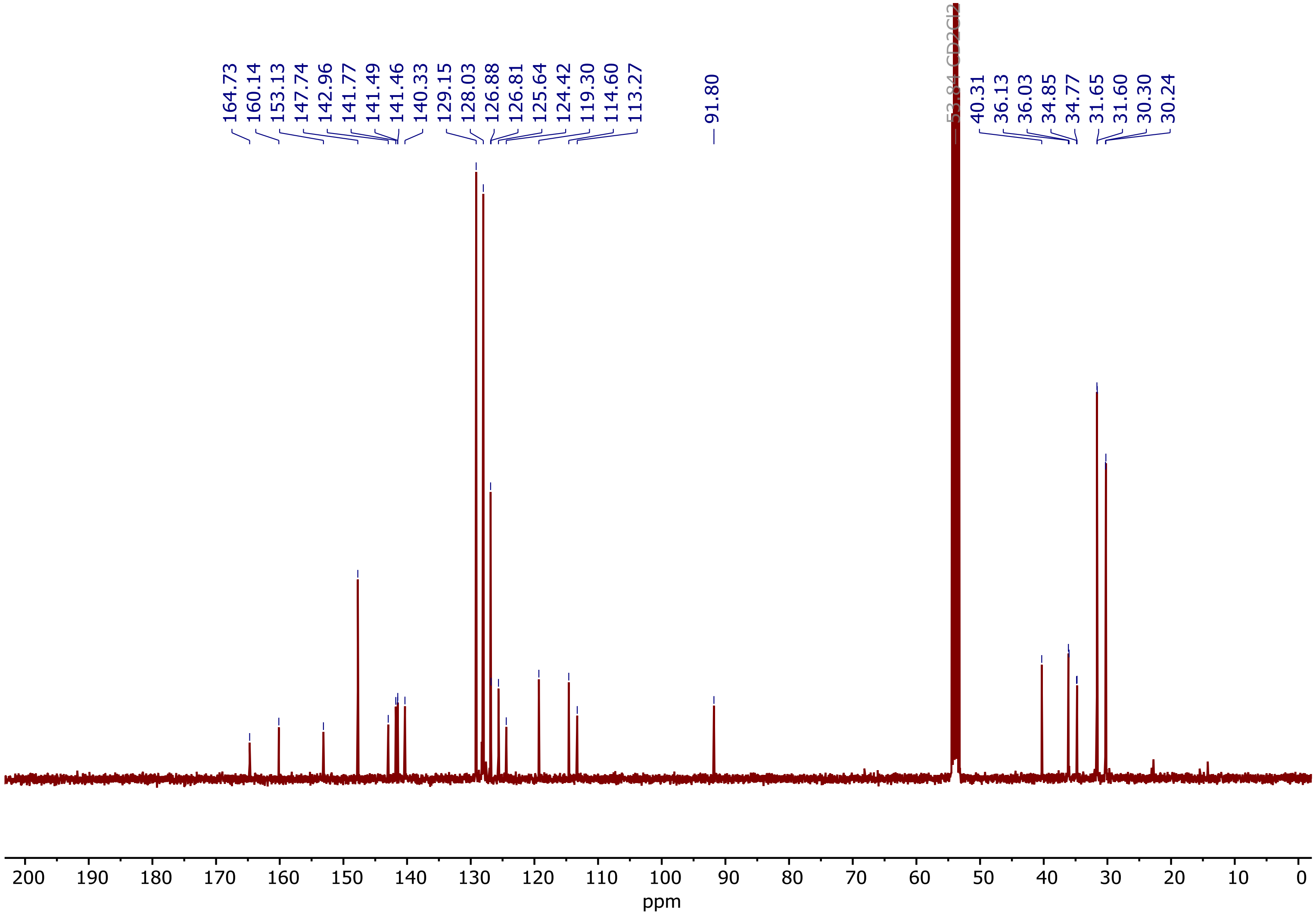

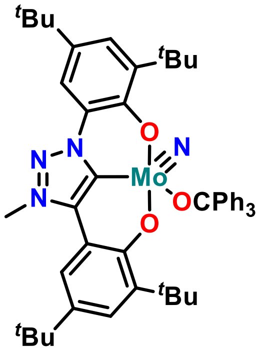

*Figure S 47: ^{13}C NMR of **1-OCPh$_3$** in CD$_2$Cl$_2$ at 298 K.*

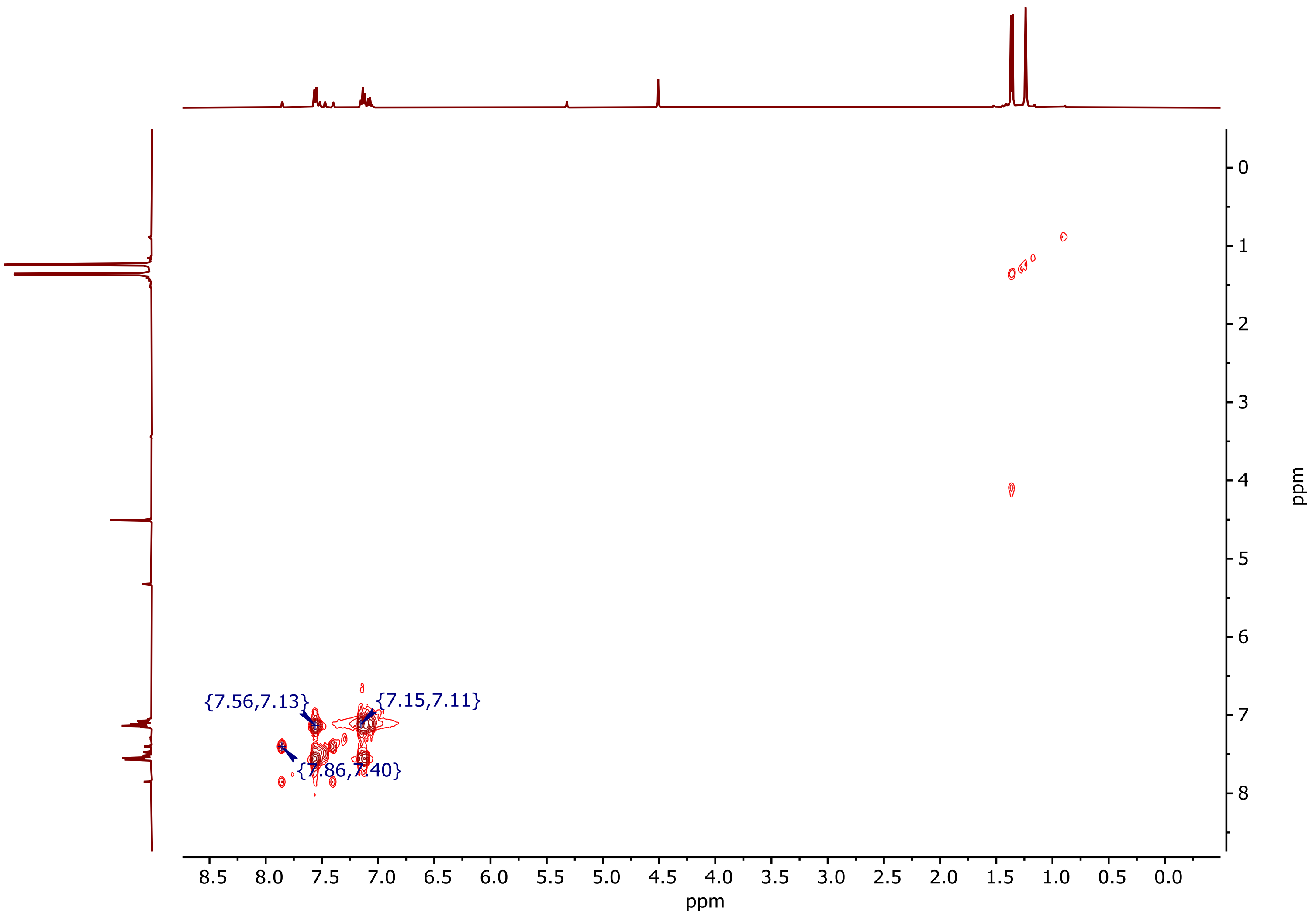

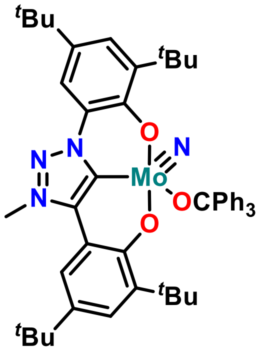

*Figure S 48: ^{1}H – ^{1}H COSY of **1-OCPh$_3$** in CD_2Cl_2 at 298 K.*

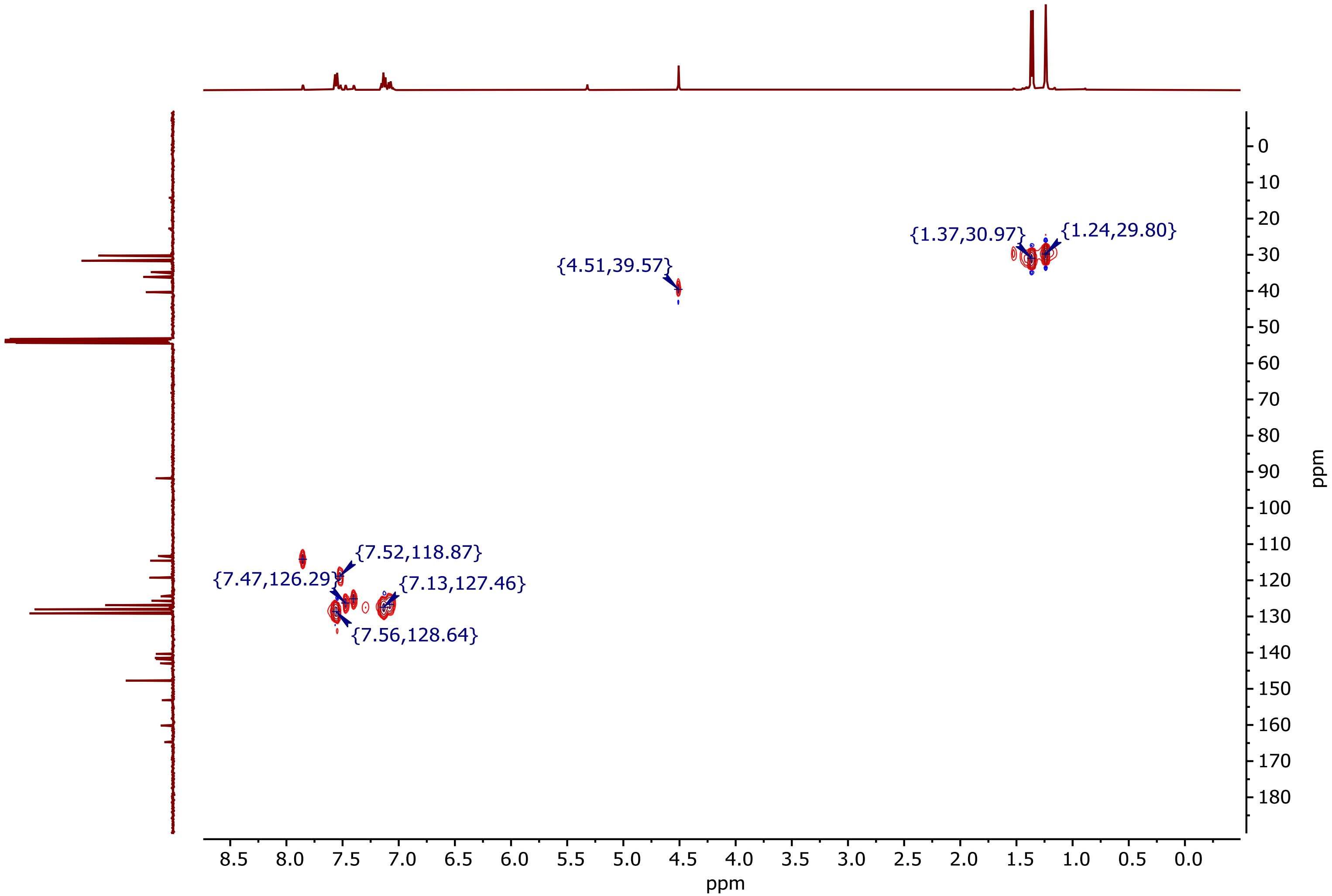

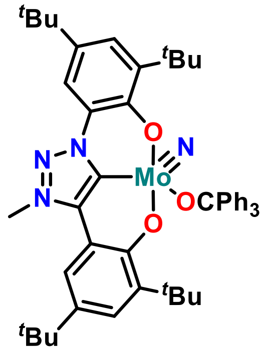

*Figure S 49: ^{1}H – ^{13}C HSQC of **1-OCPh₃** in CD_2Cl_2 at 298 K.*

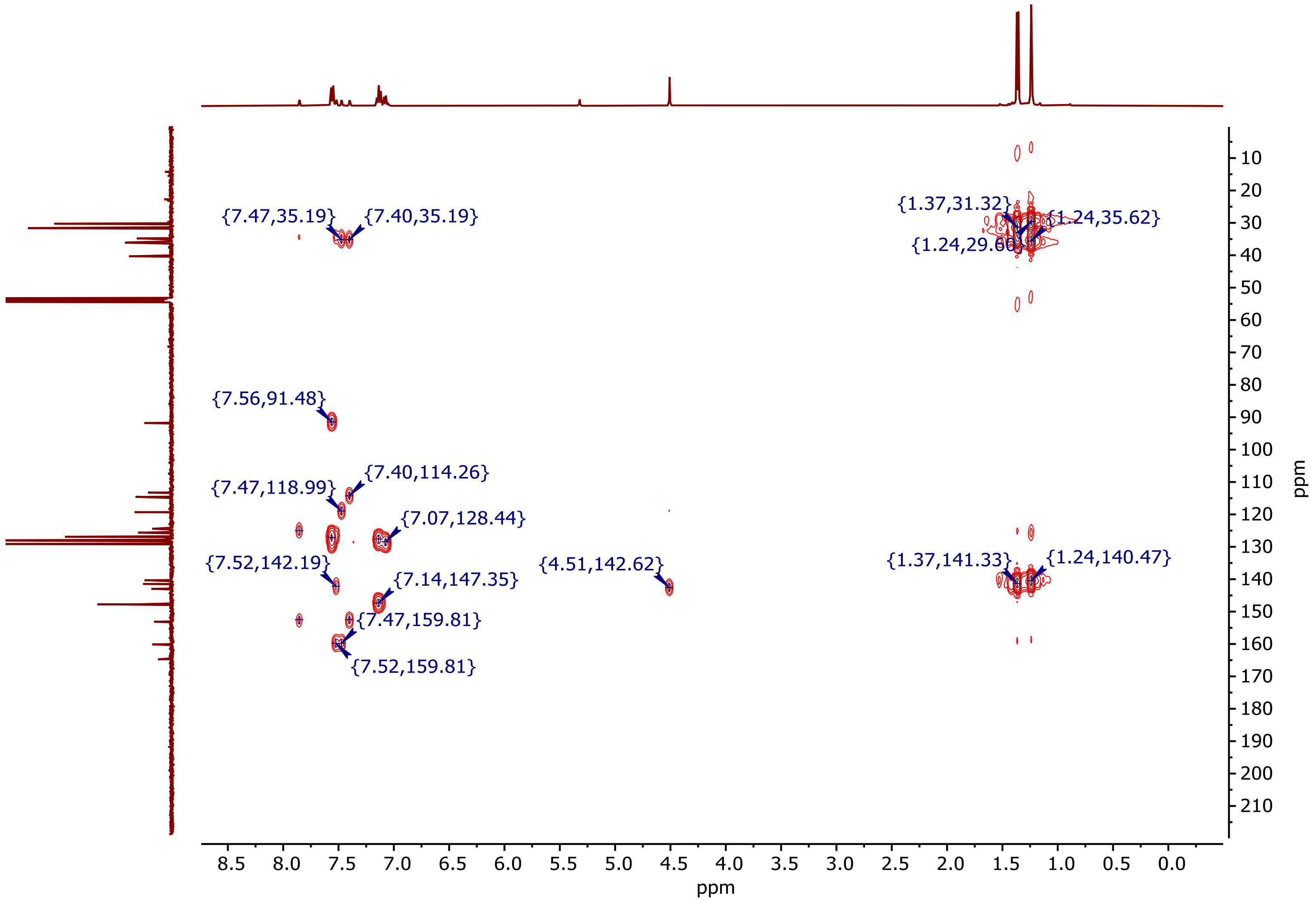

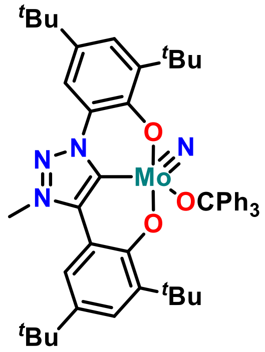

*Figure S 50: ^{1}H – ^{13}C HMBC of **1-OCPh₃** in CD_2Cl_2 at 298 K.*

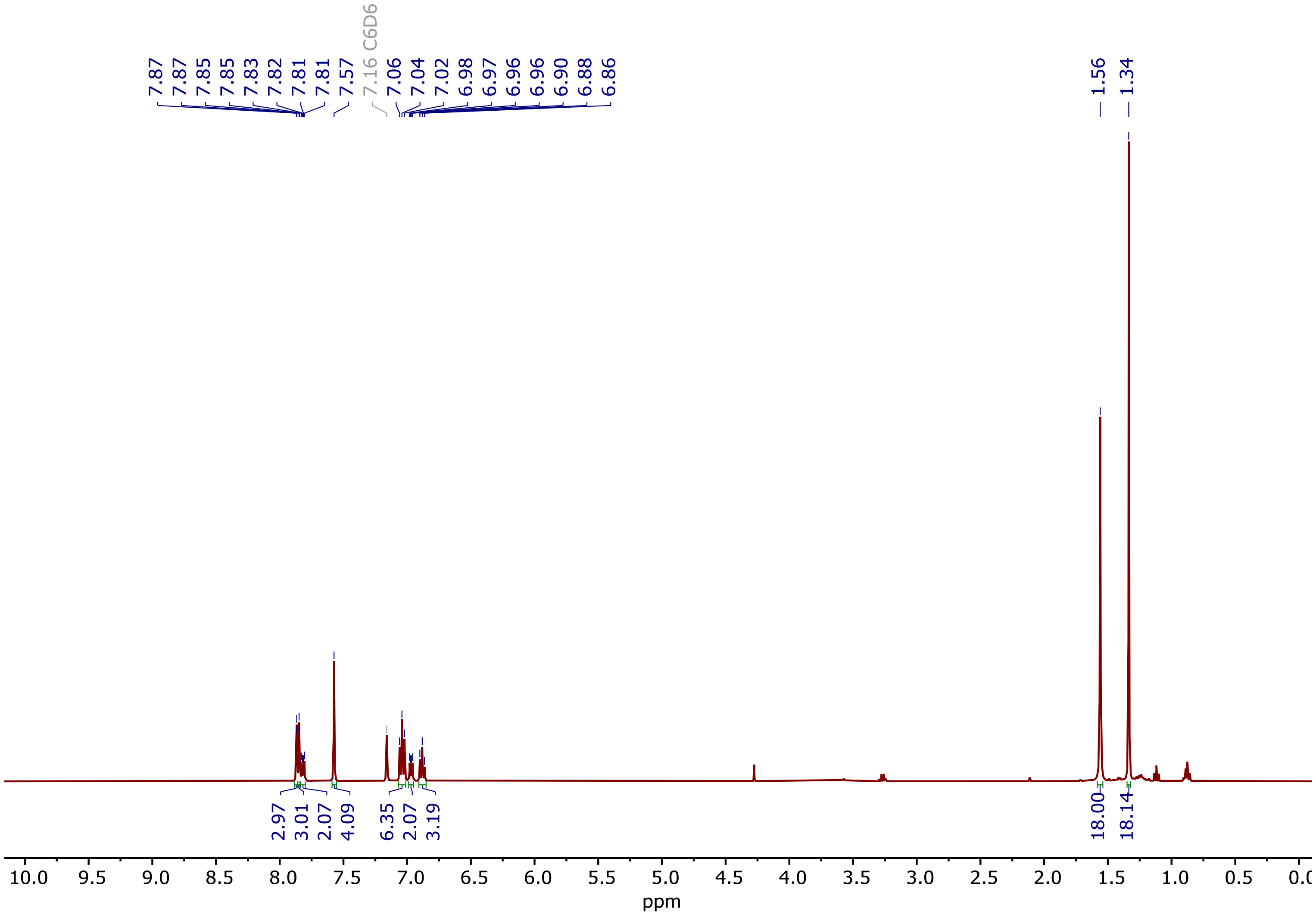

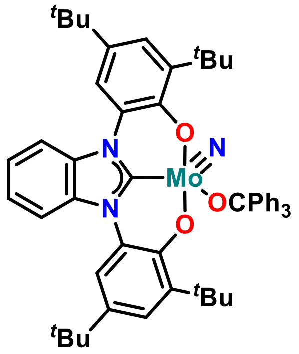

*Figure S 51: ^{1}H NMR of **2-OCPh$_3$** in C_6D_6 at 298 K.*

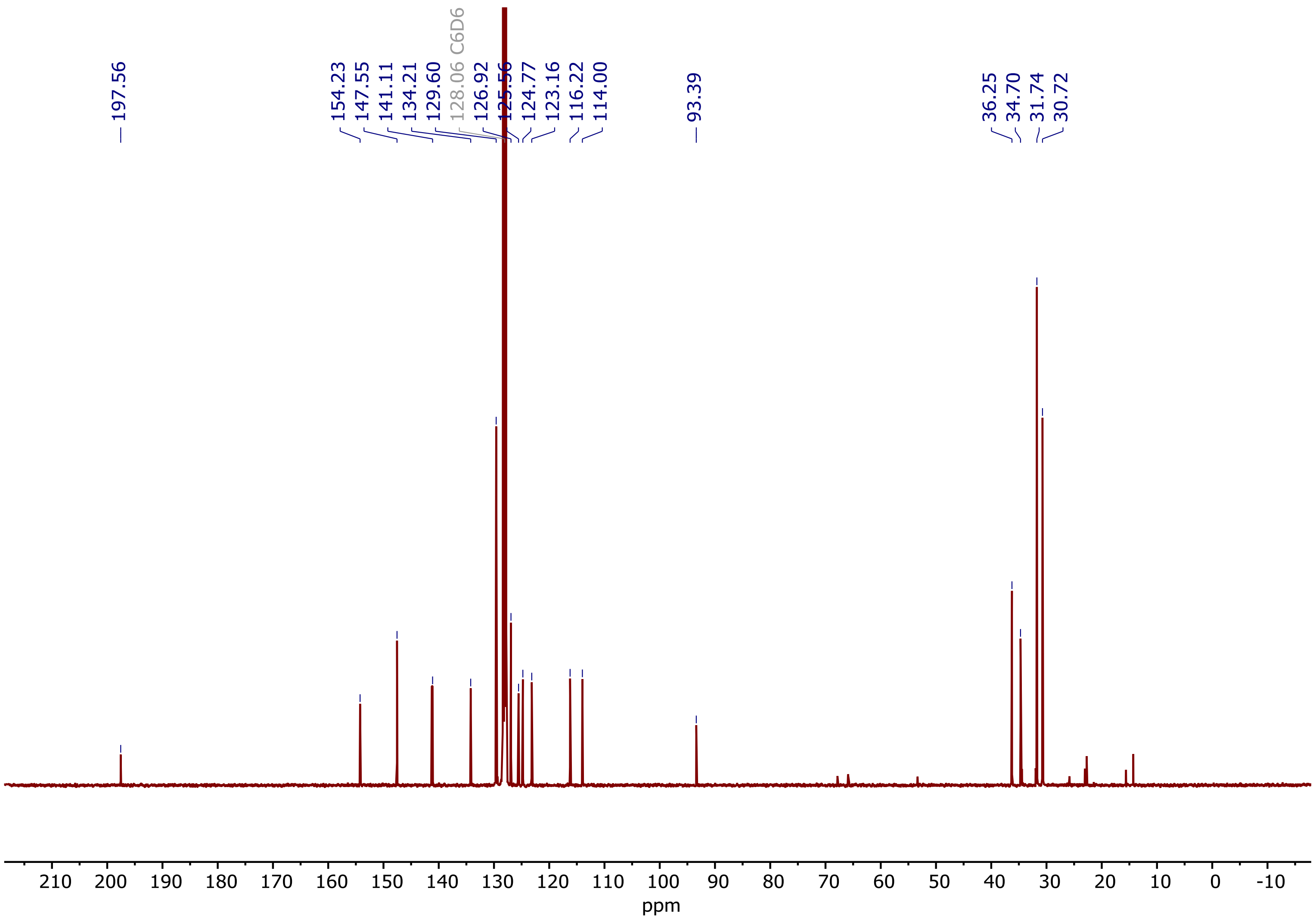

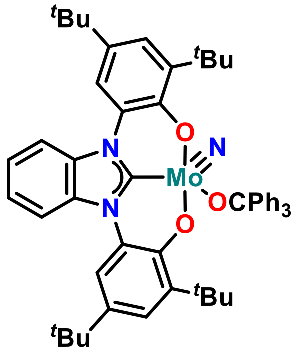

Figure S 52: ^{13}C NMR of ***2-OCPh$_3$*** *in C_6D_6 at 298 K.*

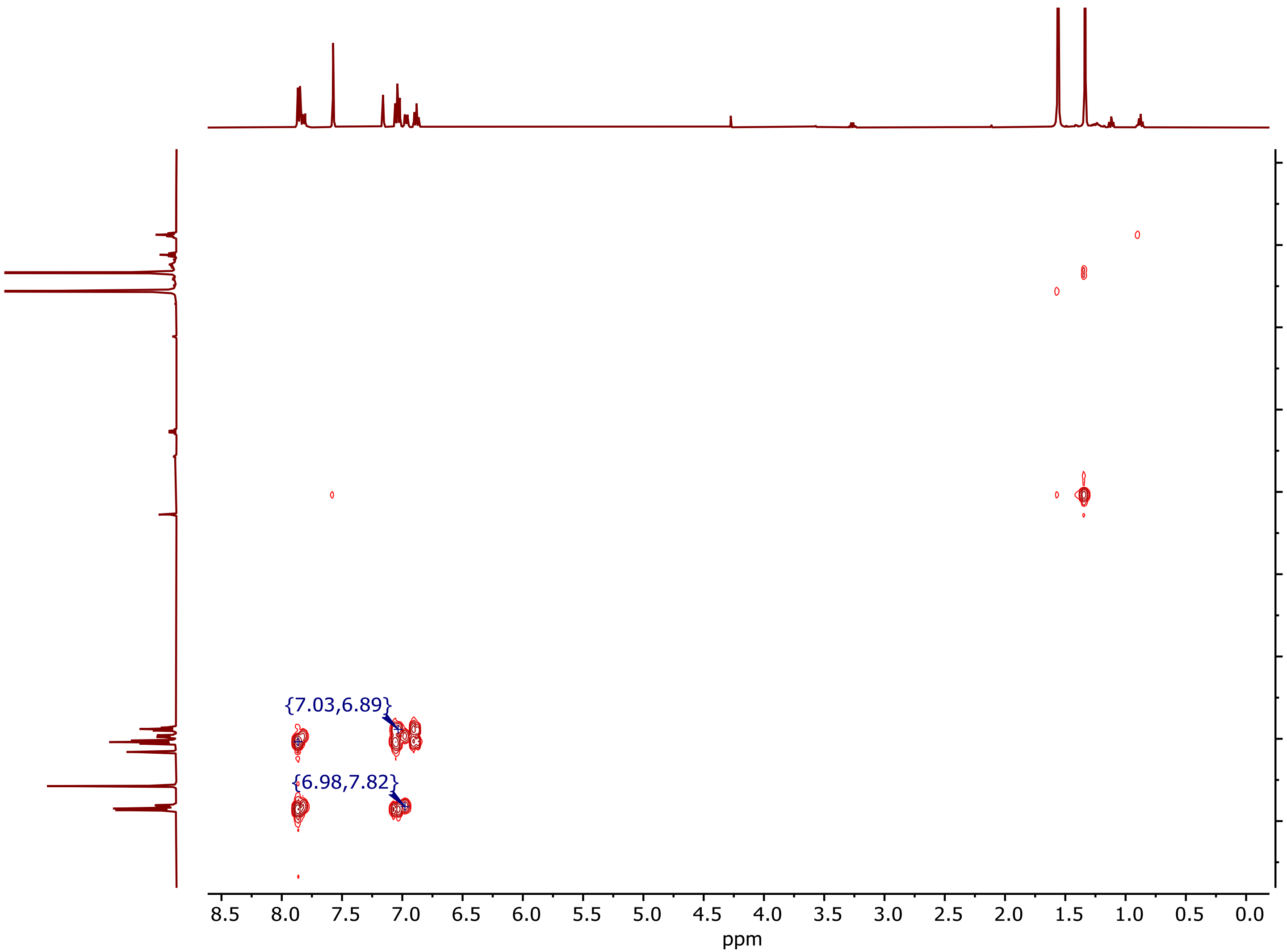

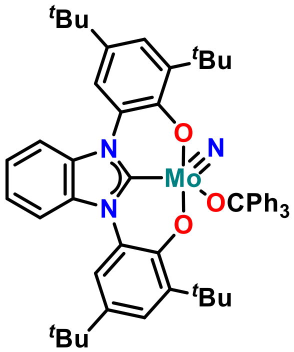

*Figure S 53: ¹H – ¹H COSY of **2-OCPh₃** in C₆D₆ at 298 K.*

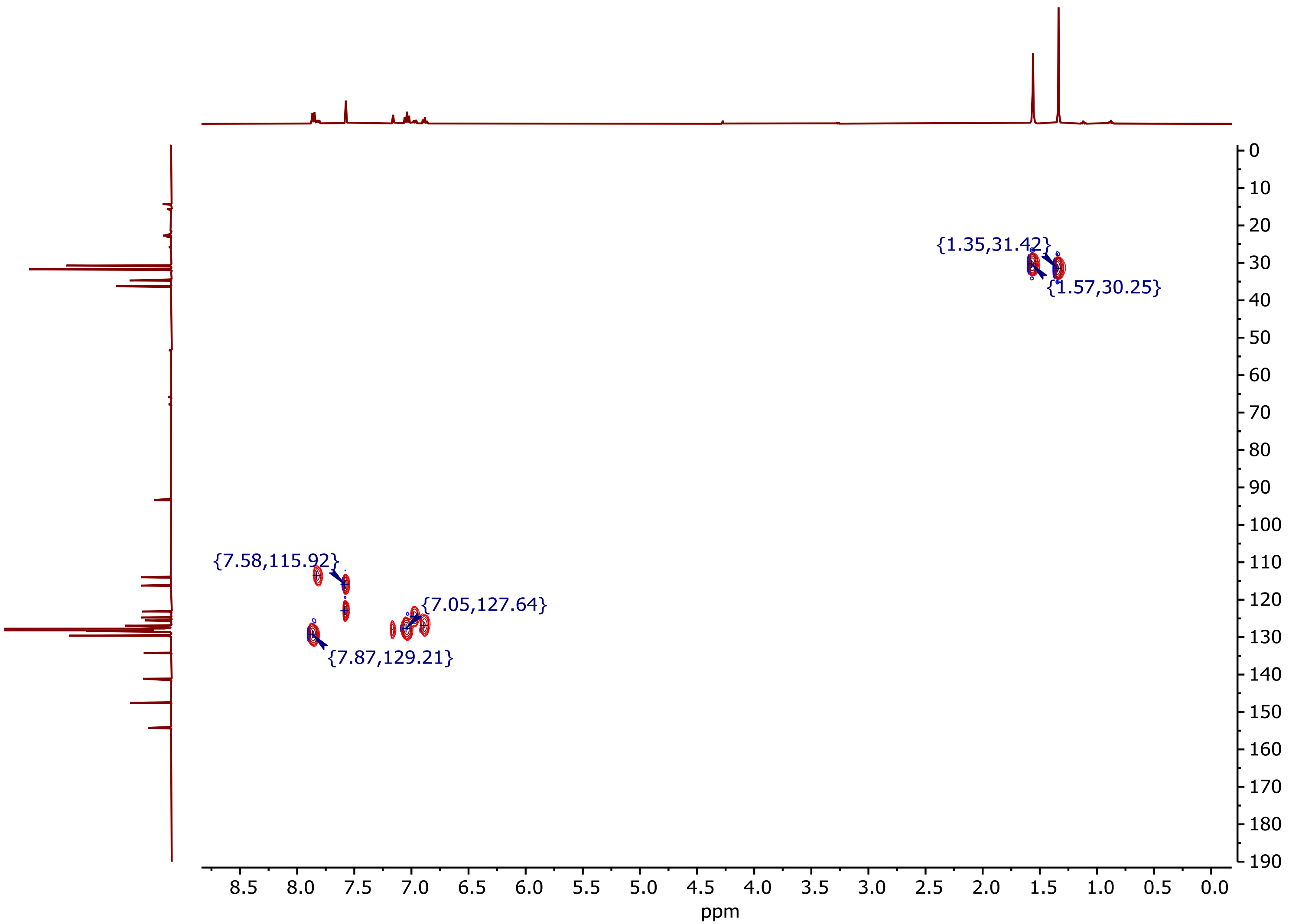

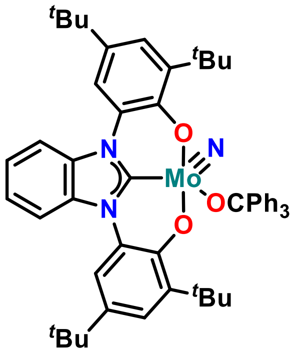

*Figure S 54: ¹H – ¹³C HSQC of **2-OCPh₃** in C₆D₆ at 298 K.*

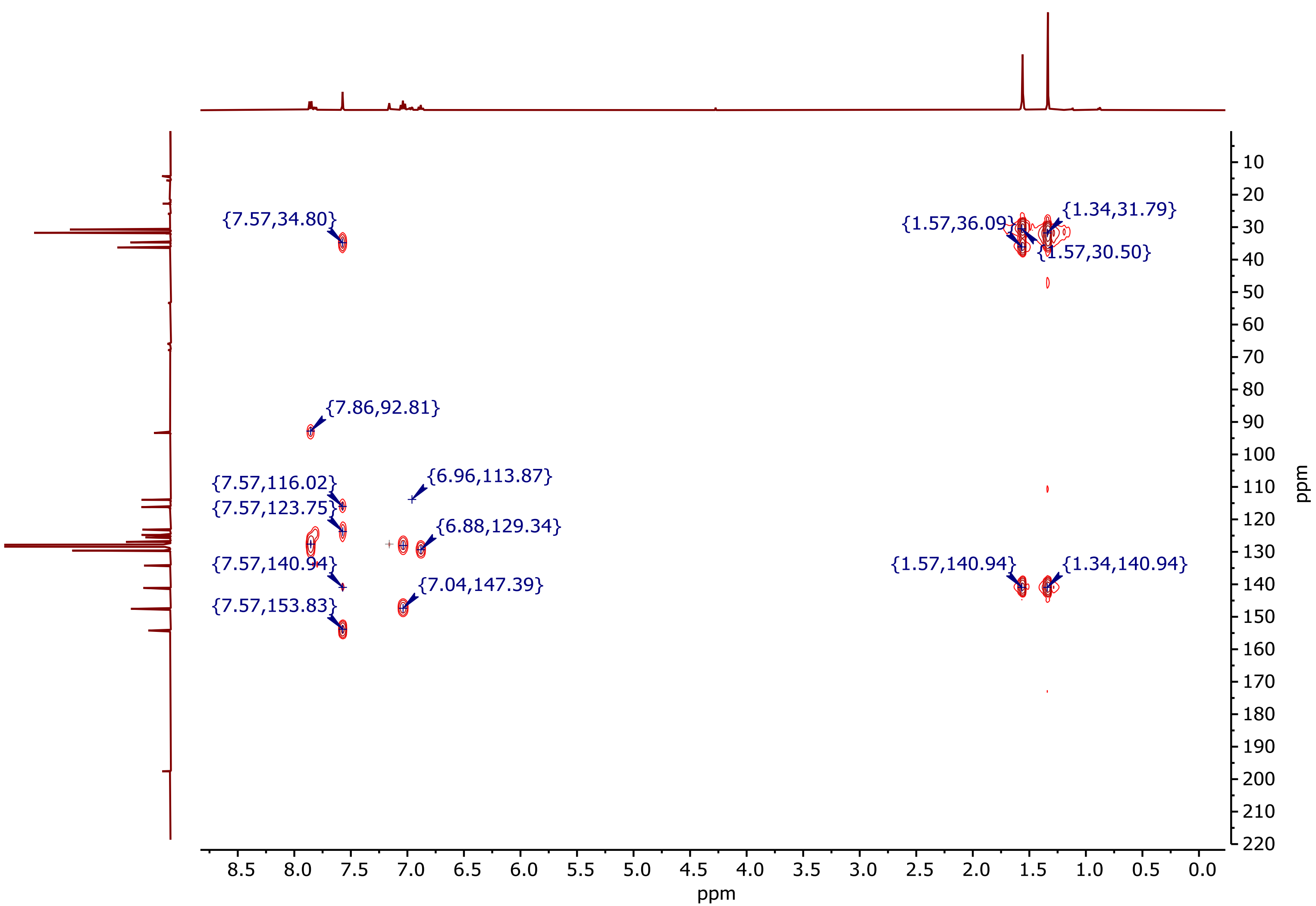

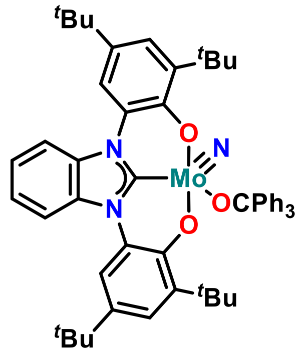

*Figure S 55: $^{1}H - ^{13}C$ HMBC of **2-OCPh₃** in C_6D_6 at 298 K.*

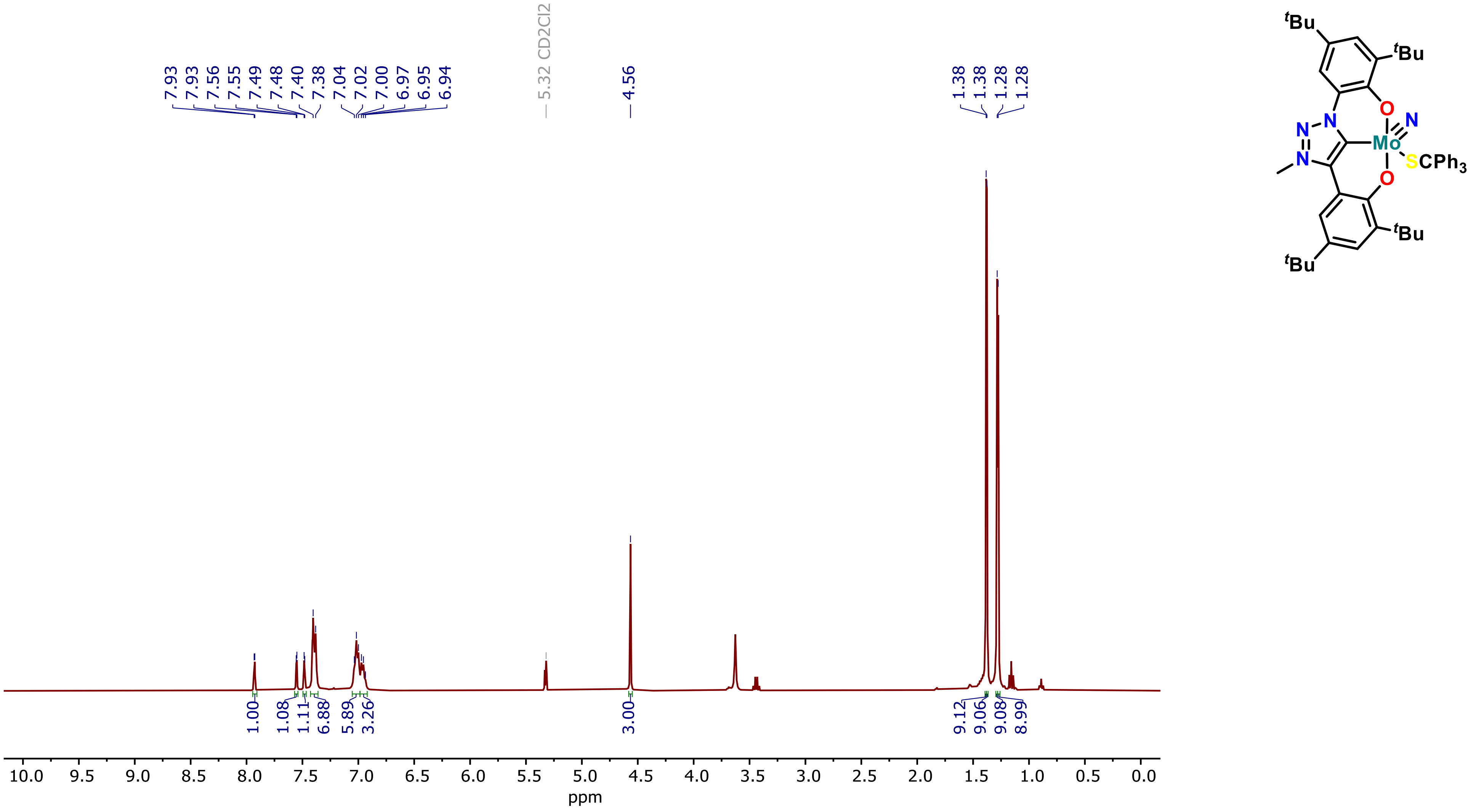

*Figure S 56: ^{1}H NMR of **1-SCPh₃** in CD_2Cl_2 at 298 K.*

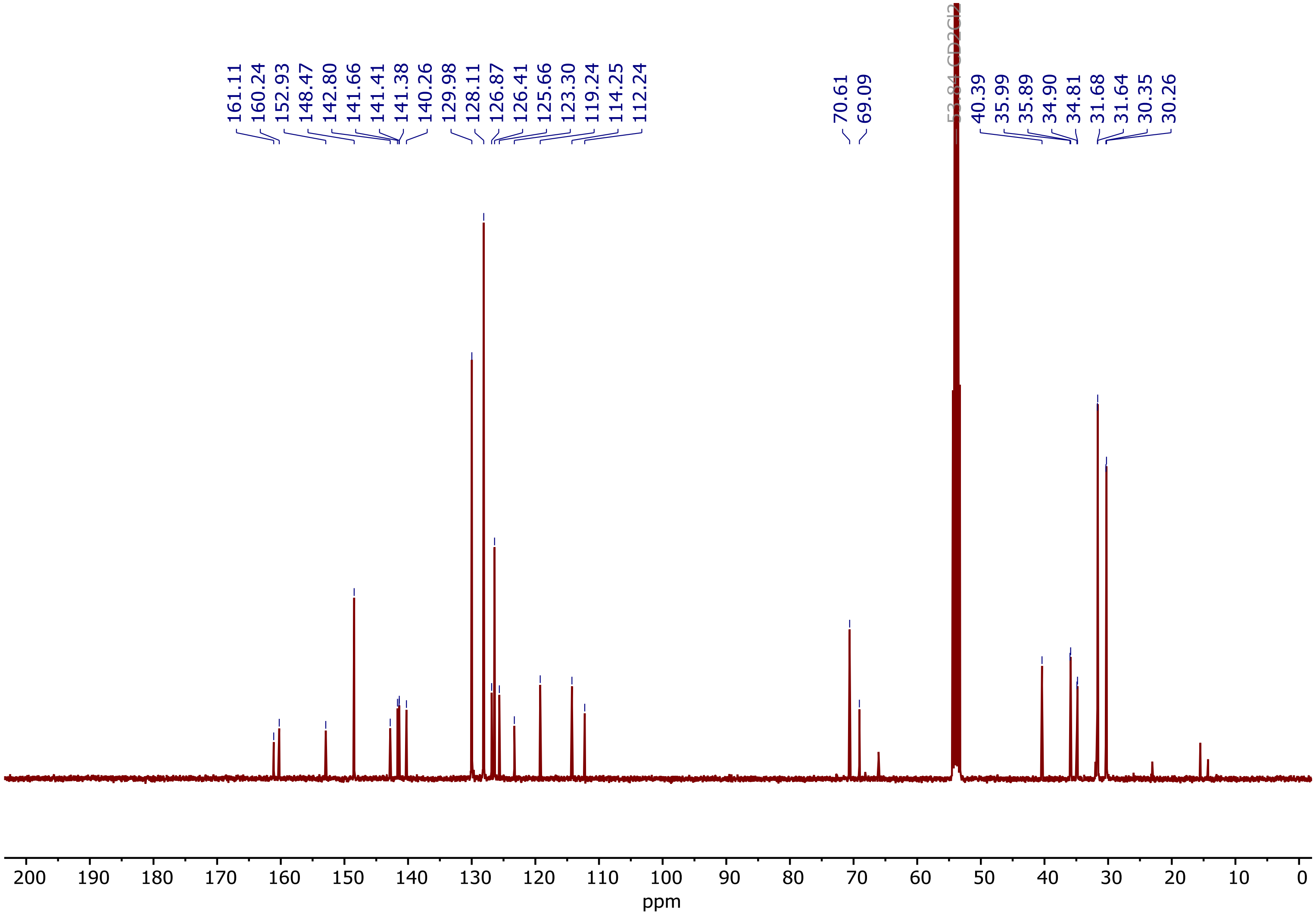

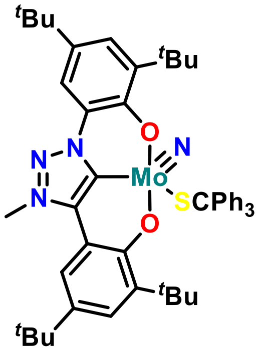

*Figure S 57: ^{13}C NMR of **1-SCPh₃** in CD_2Cl_2 at 298 K.*

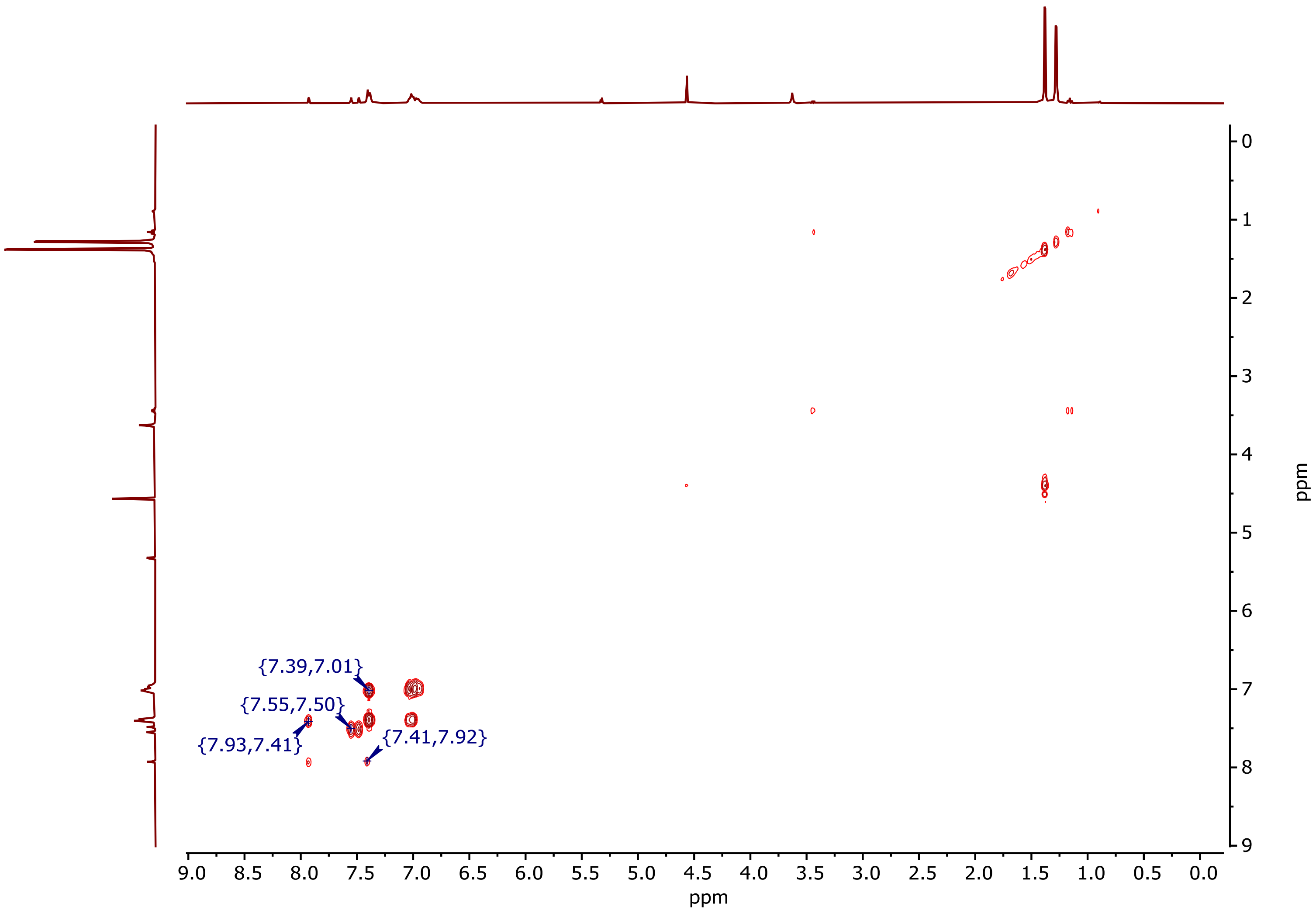

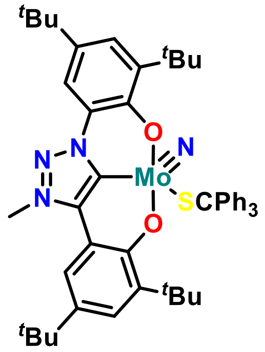

*Figure S 58: $^{1}H - ^{1}H$ COSY of **1-SCPh$_3$** in CD_2Cl_2 at 298 K.*

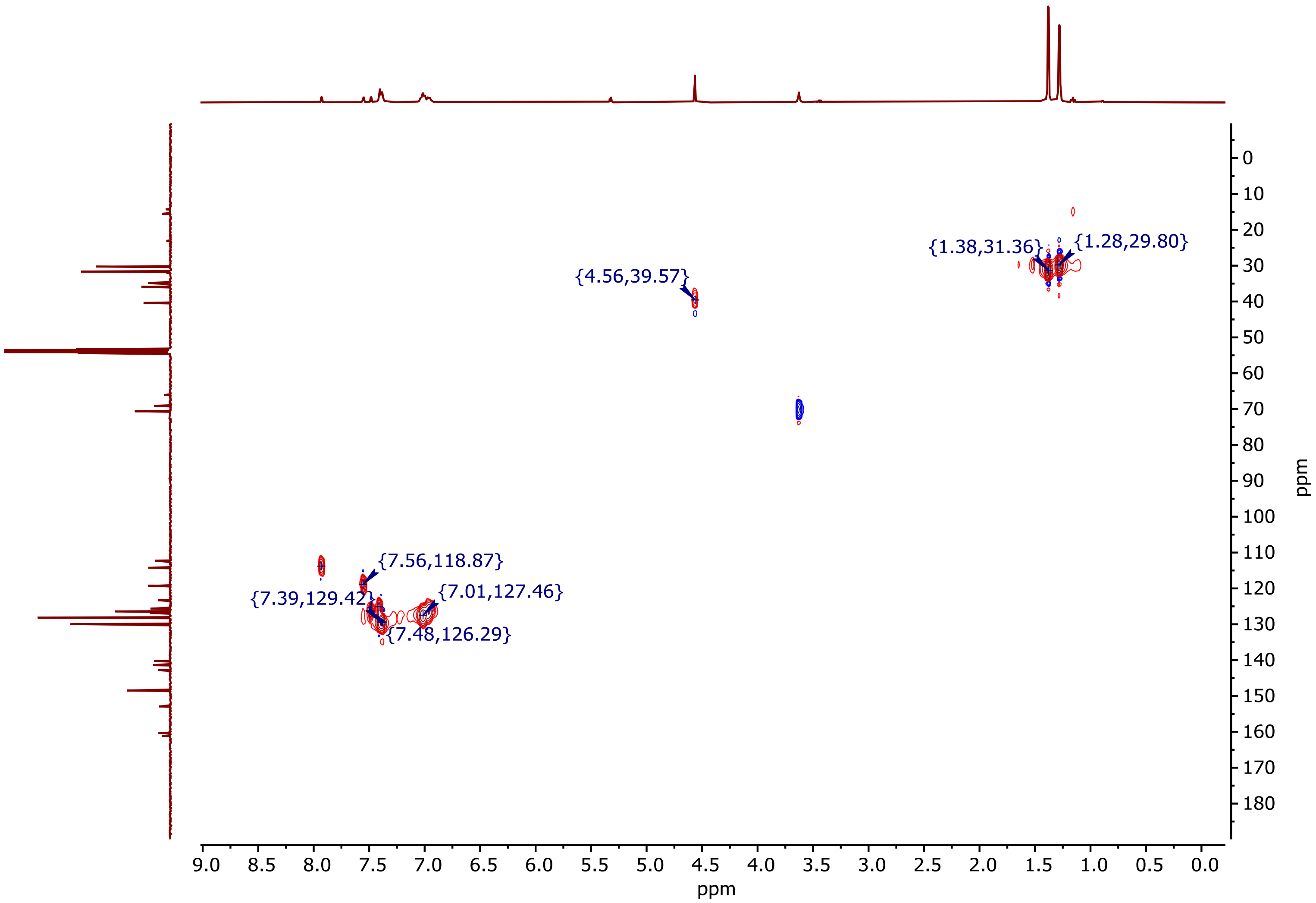

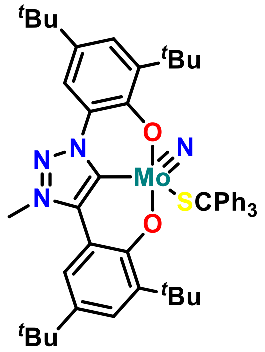

*Figure S 59: $^{1}H - ^{13}C$ HSQC of **1-SCPh₃** in CD_2Cl_2 at 298 K.*

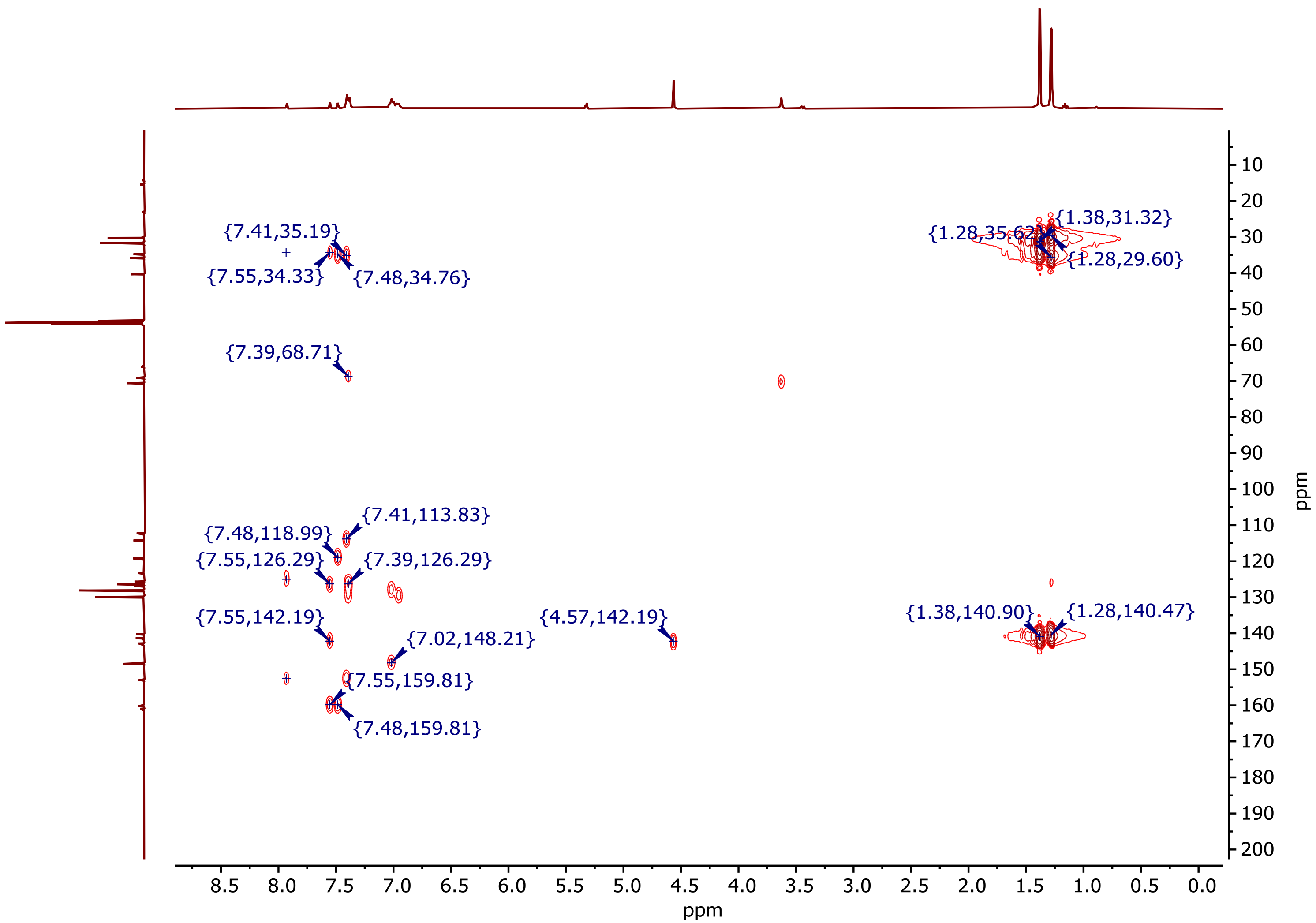

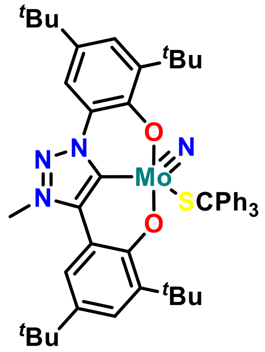

*Figure S 60: ¹H – ¹³C HMBC of **1-SCPh₃** in CD_2Cl_2 at 298 K.*

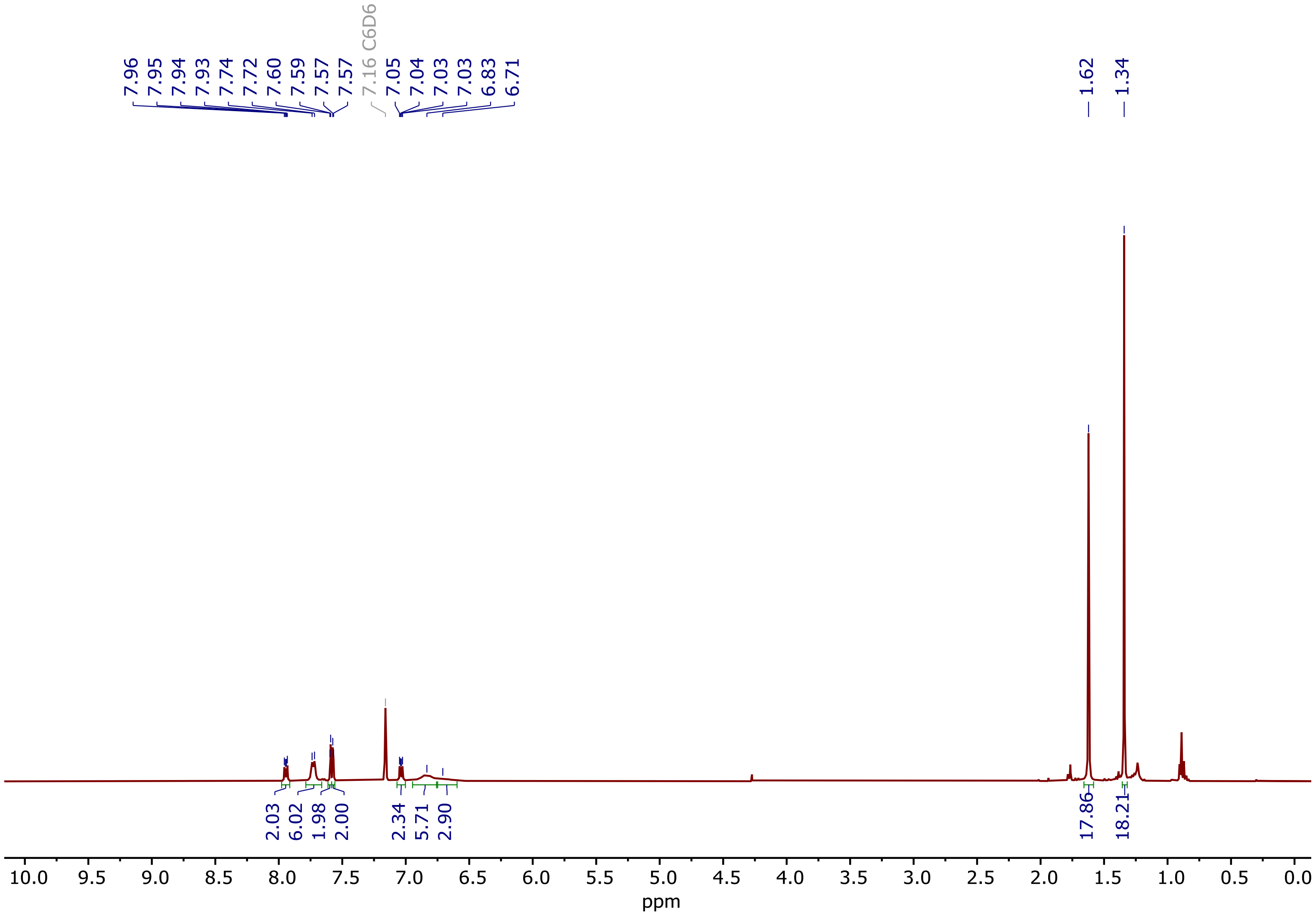

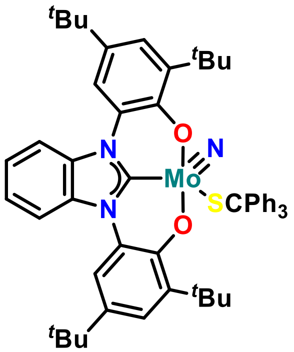

*Figure S 61: ¹H NMR of **2-SCPh₃** in C₆D₆ at 298 K.*

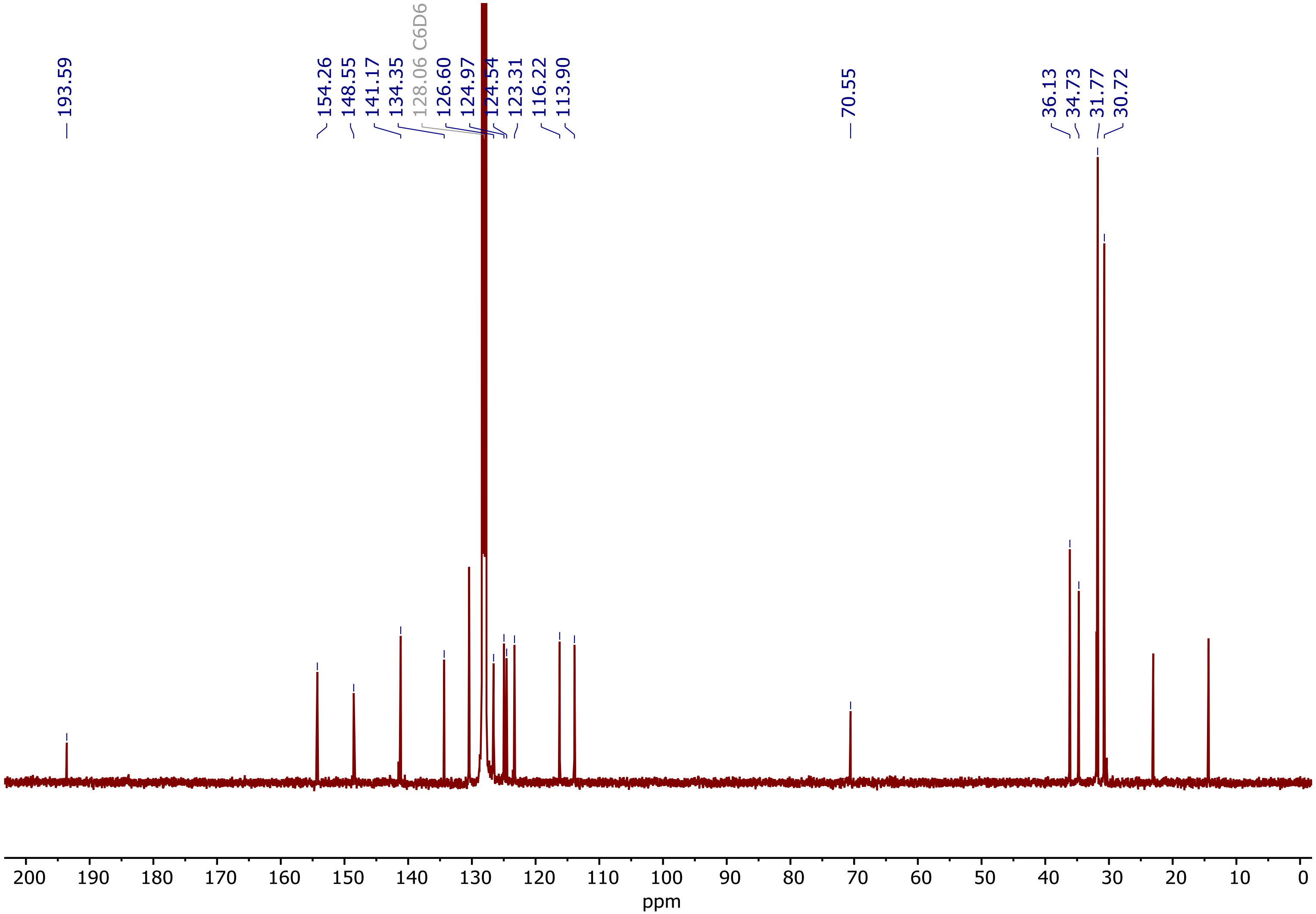

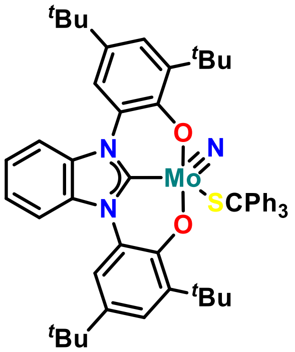

*Figure S 62: ^{13}C NMR of **2-SCPh$_3$** in C_6D_6 at 298 K.*

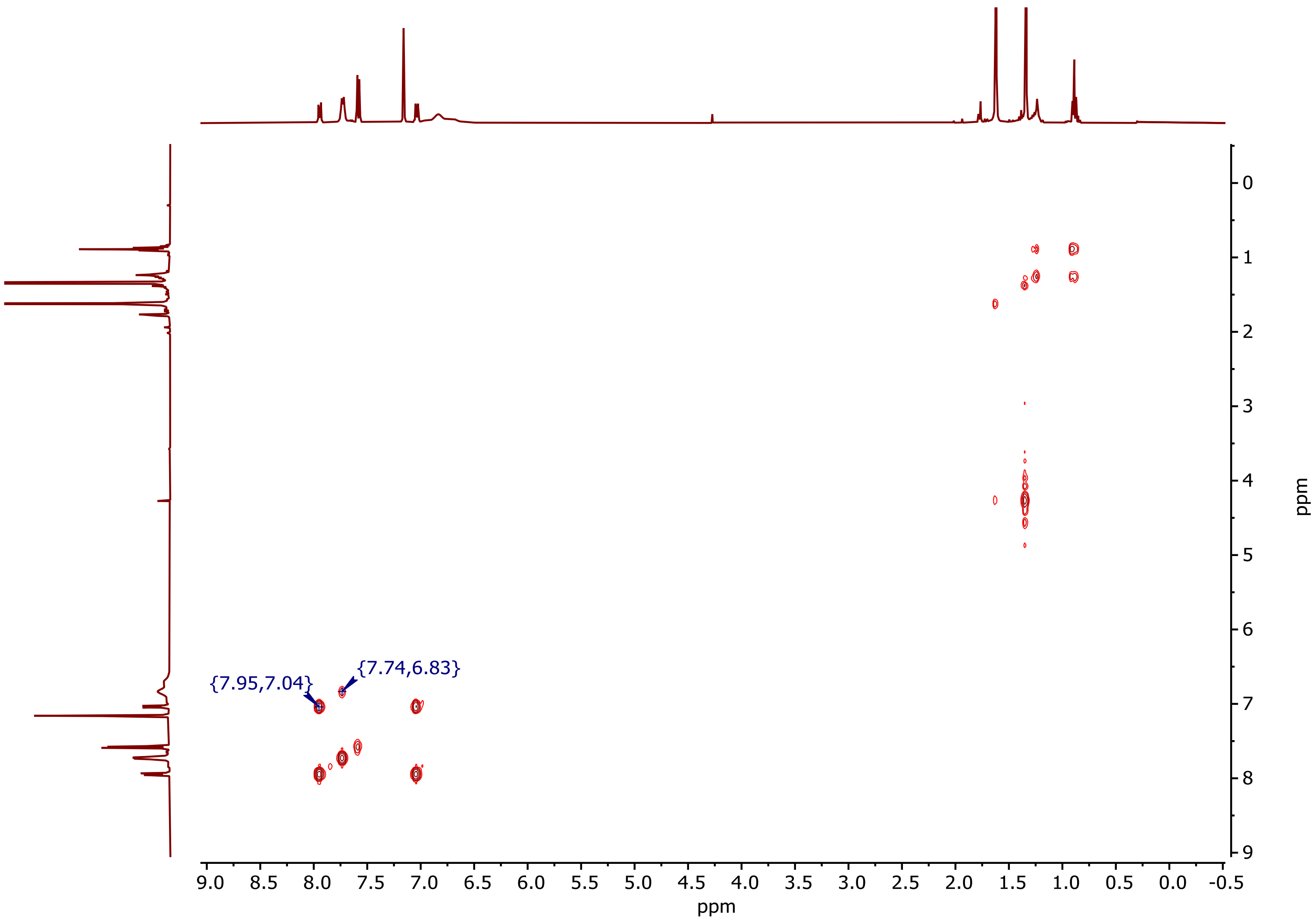

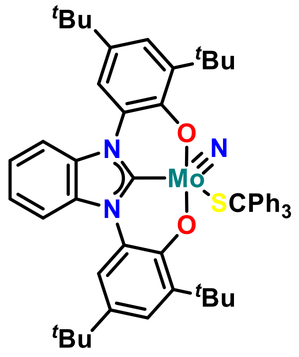

*Figure S 63: ¹H – ¹H COSY of **2-SCPh₃** in C₆D₆ at 298 K.*

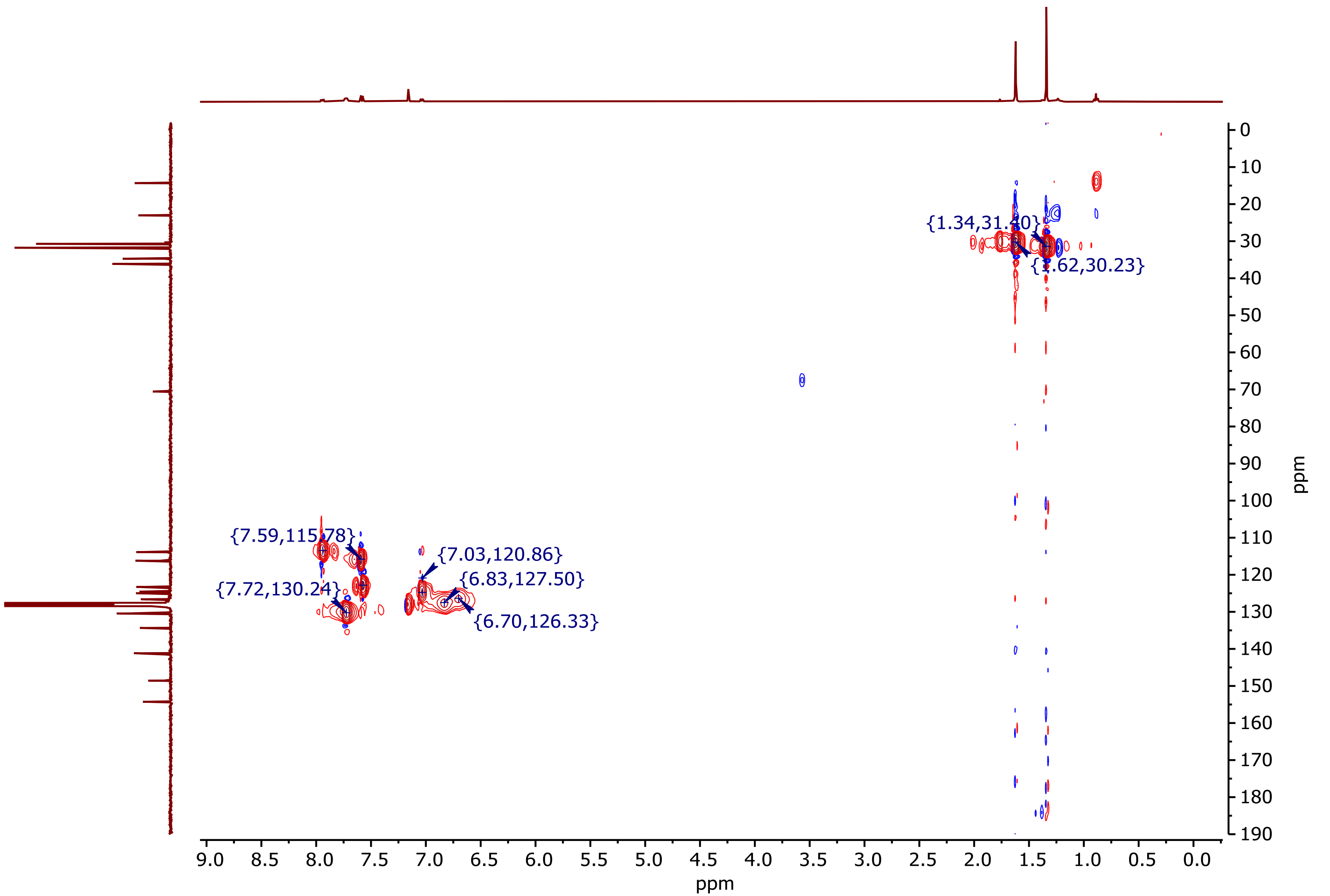

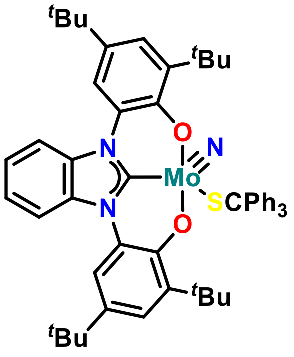

*Figure S 64: $^{1}H - ^{13}C$ HSQC of **2-SCPh$_3$** in C_6D_6 at 298 K.*

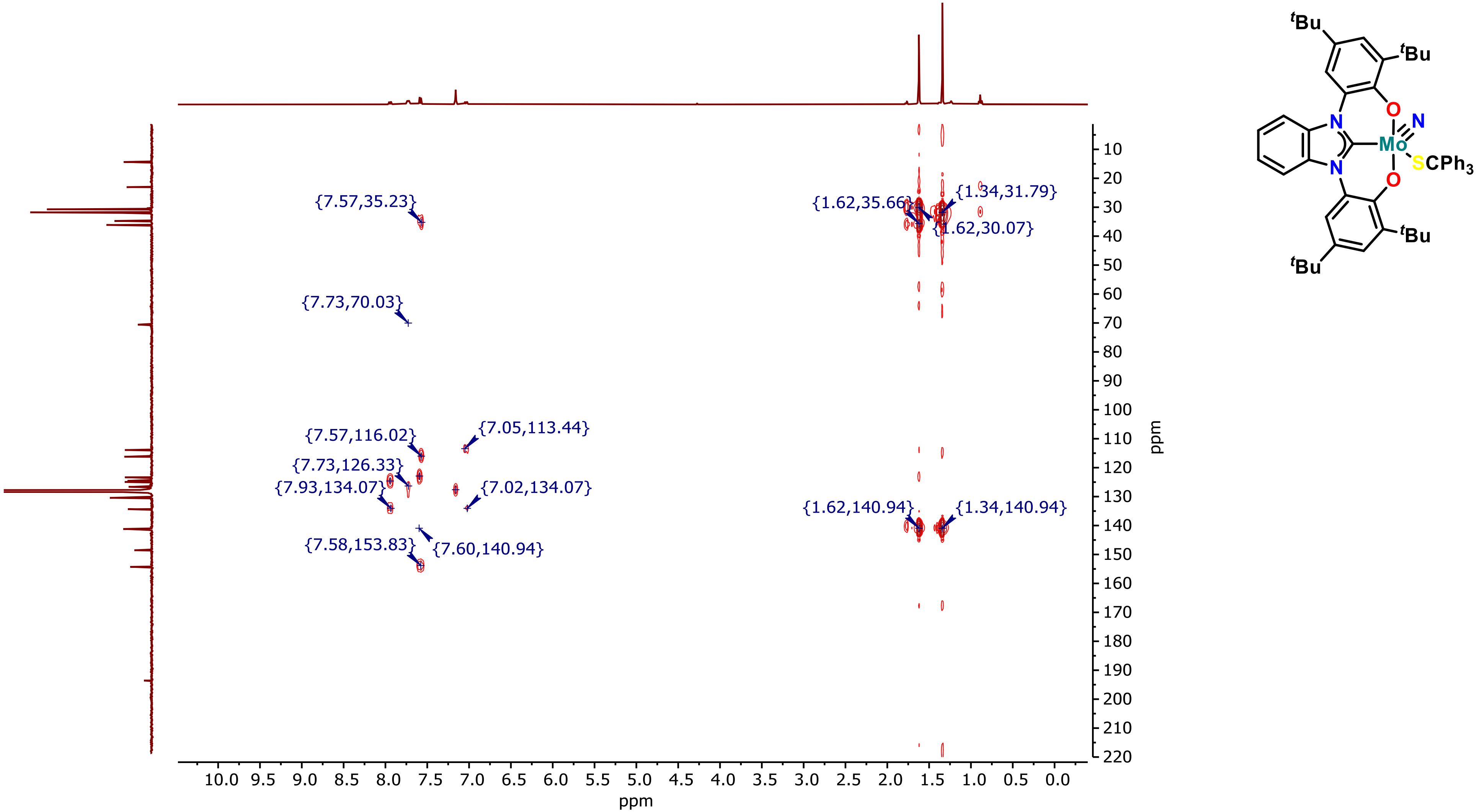

*Figure S 65: $^{1}H - ^{13}C$ HMBC of **2-SCPh$_3$** in C_6D_6 at 298 K.*

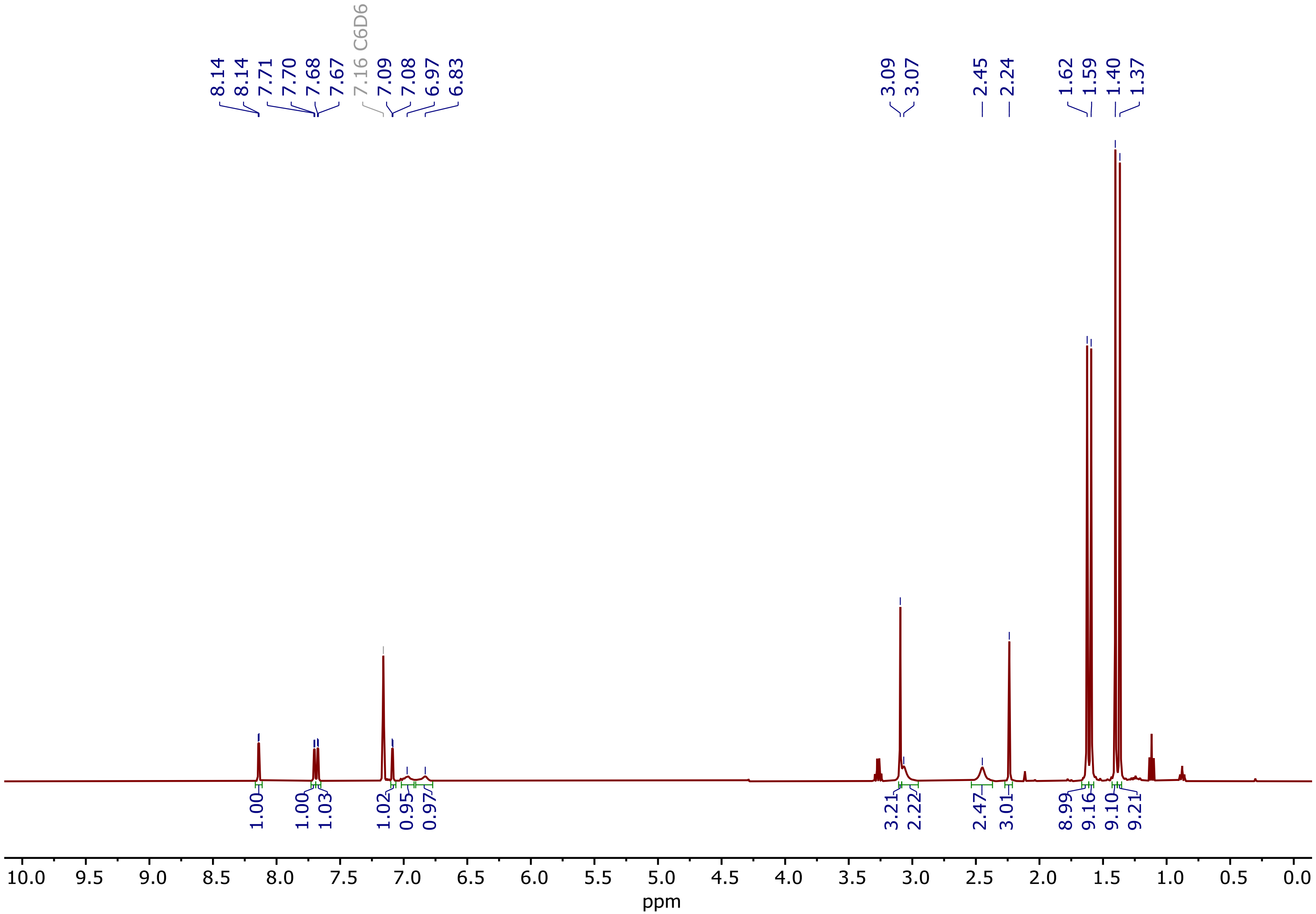

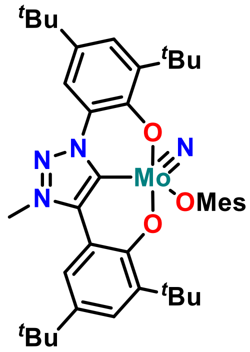

*Figure S 66: ^{1}H NMR of **1-OMes** in C_6D_6 at 298 K.*

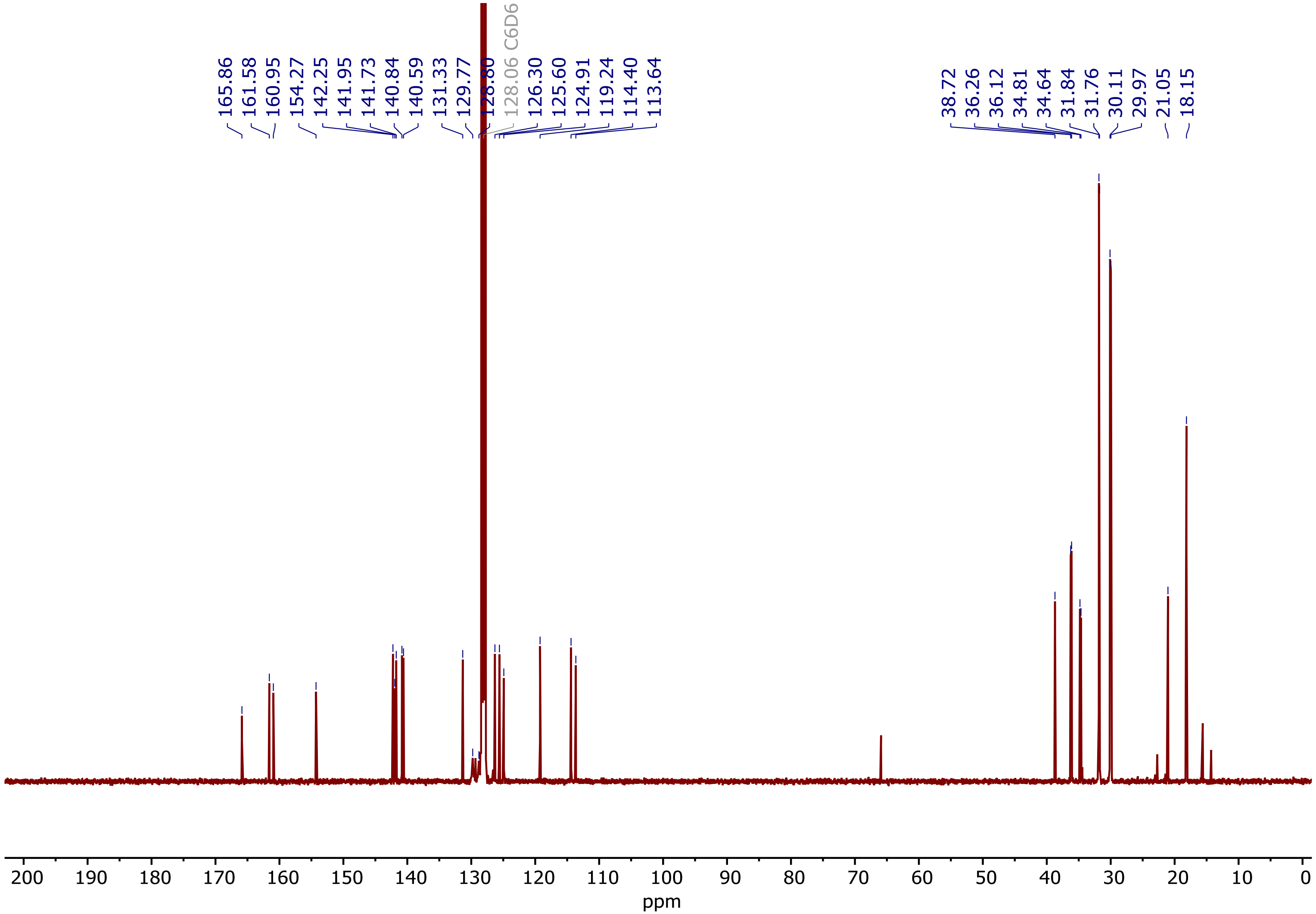

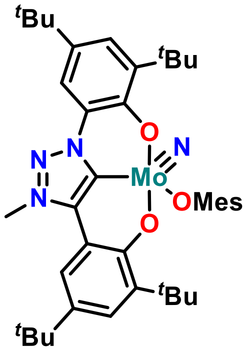

*Figure S 67: ^{13}C NMR of **1-OMes** in C_6D_6 at 298 K.*

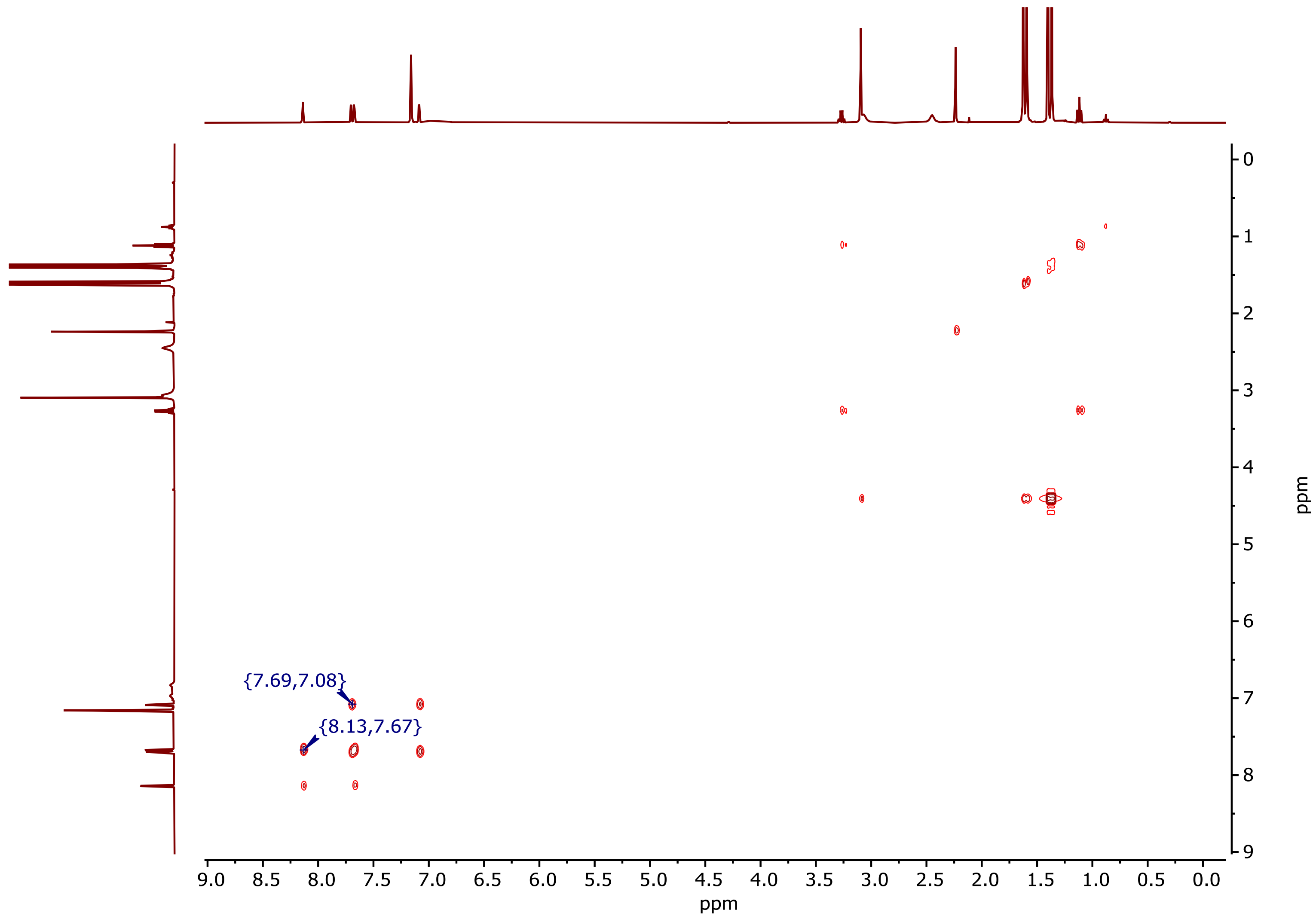

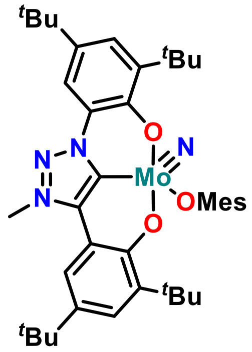

*Figure S 68: ^{1}H – ^{1}H COSY of **1-OMes** in C_6D_6 at 298 K.*

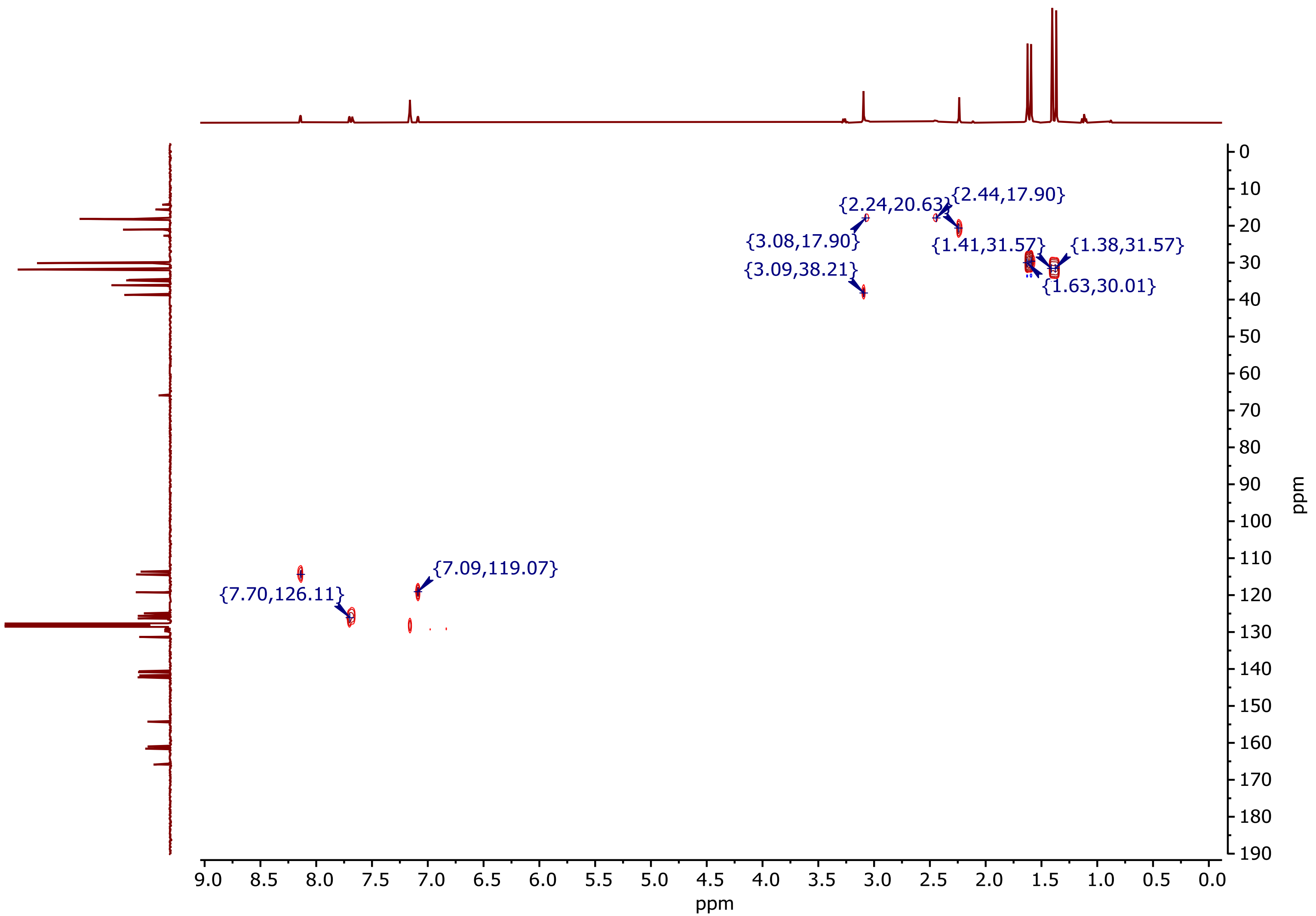

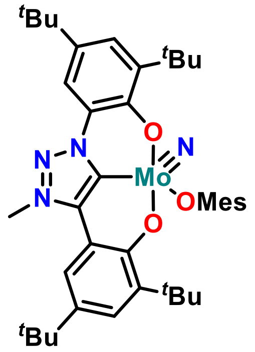

*Figure S 69: ¹H – ¹³C HSQC of **1-OMes** in C_6D_6 at 298 K.*

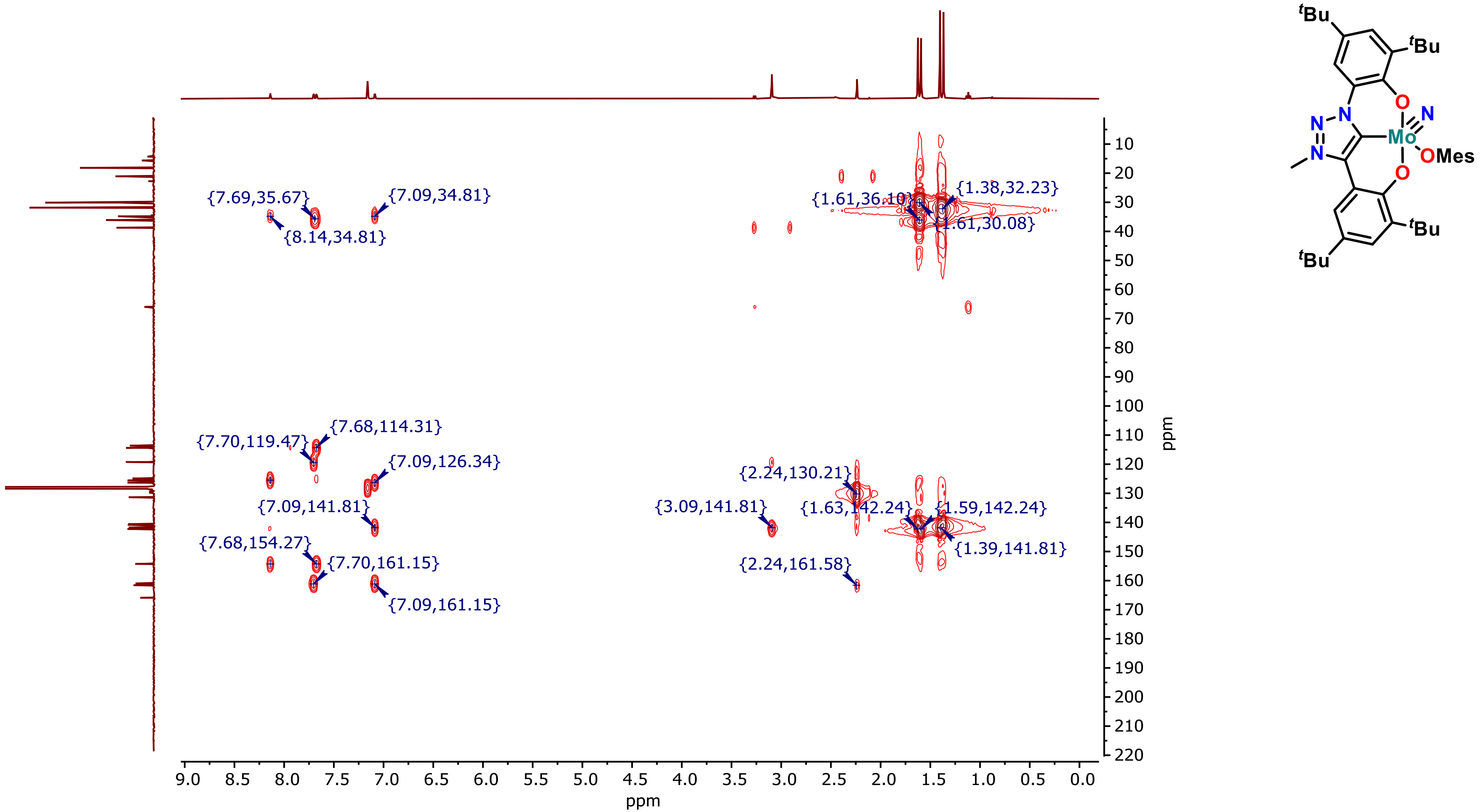

*Figure S 70: ^{1}H – ^{13}C HMBC of **1-OMes** in C_6D_6 at 298 K.*

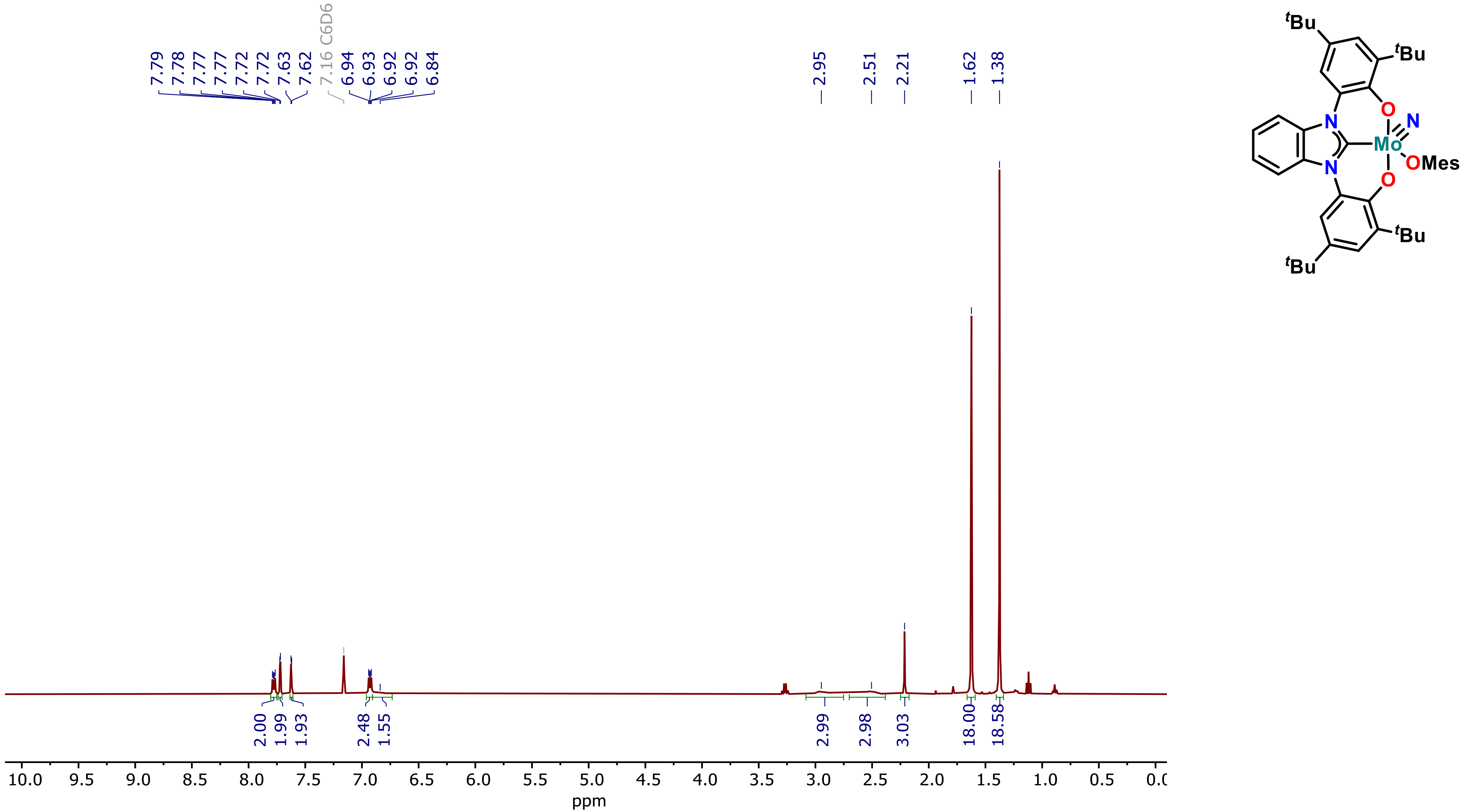

*Figure S 71: ¹H NMR of **2-OMes** in C_6D_6 at 298 K.*

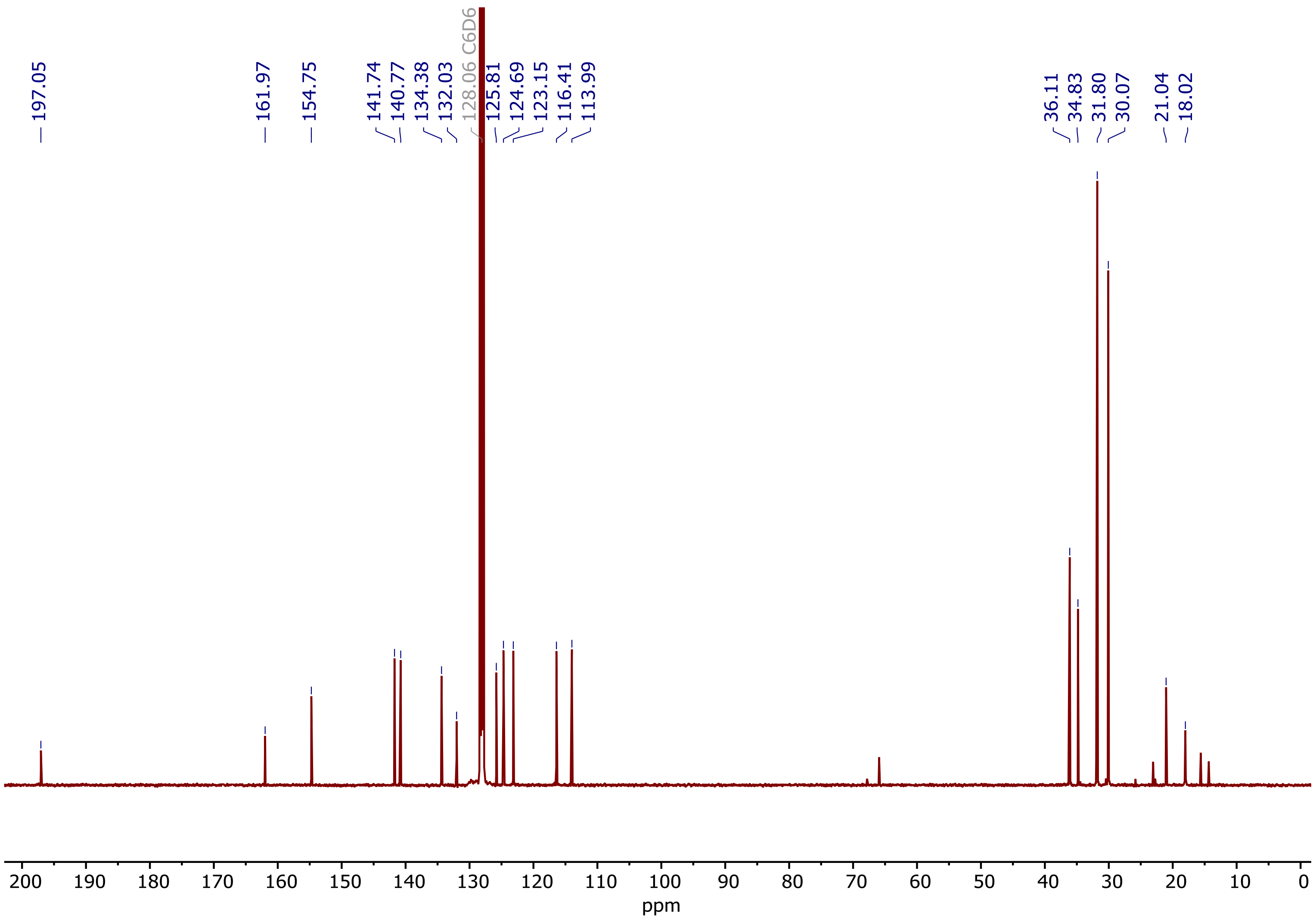

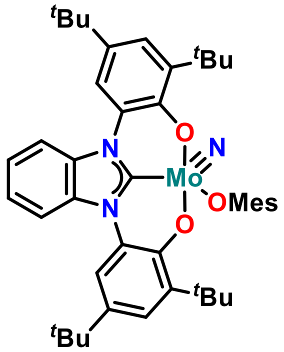

*Figure S 72: ^{13}C NMR of **2-OMes** in C_6D_6 at 298 K.*

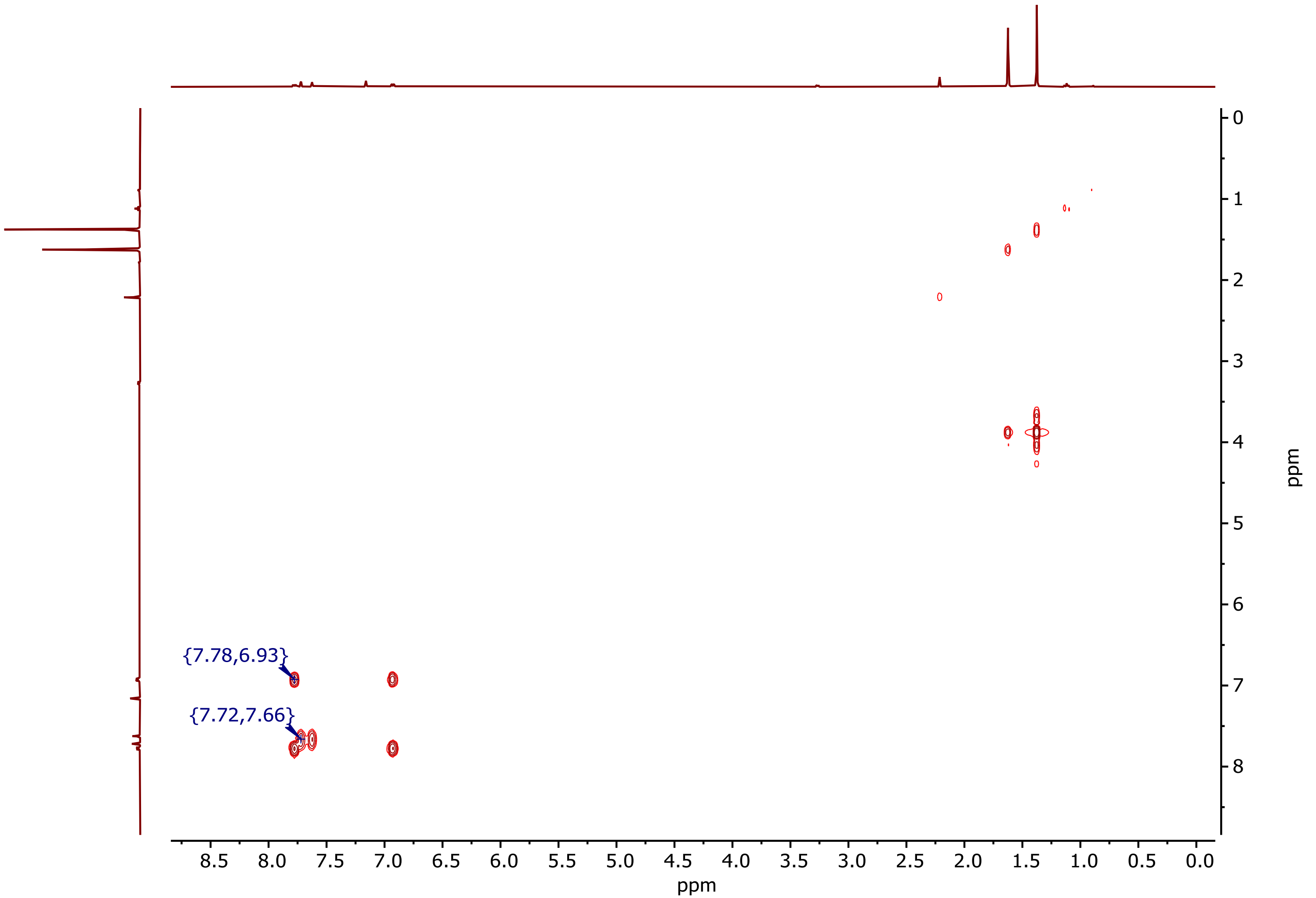

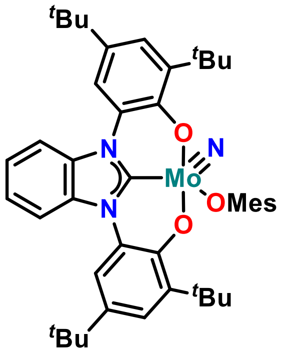

*Figure S 73: ^{1}H – ^{1}H COSY of **2-OMes** in C_6D_6 at 298 K.*

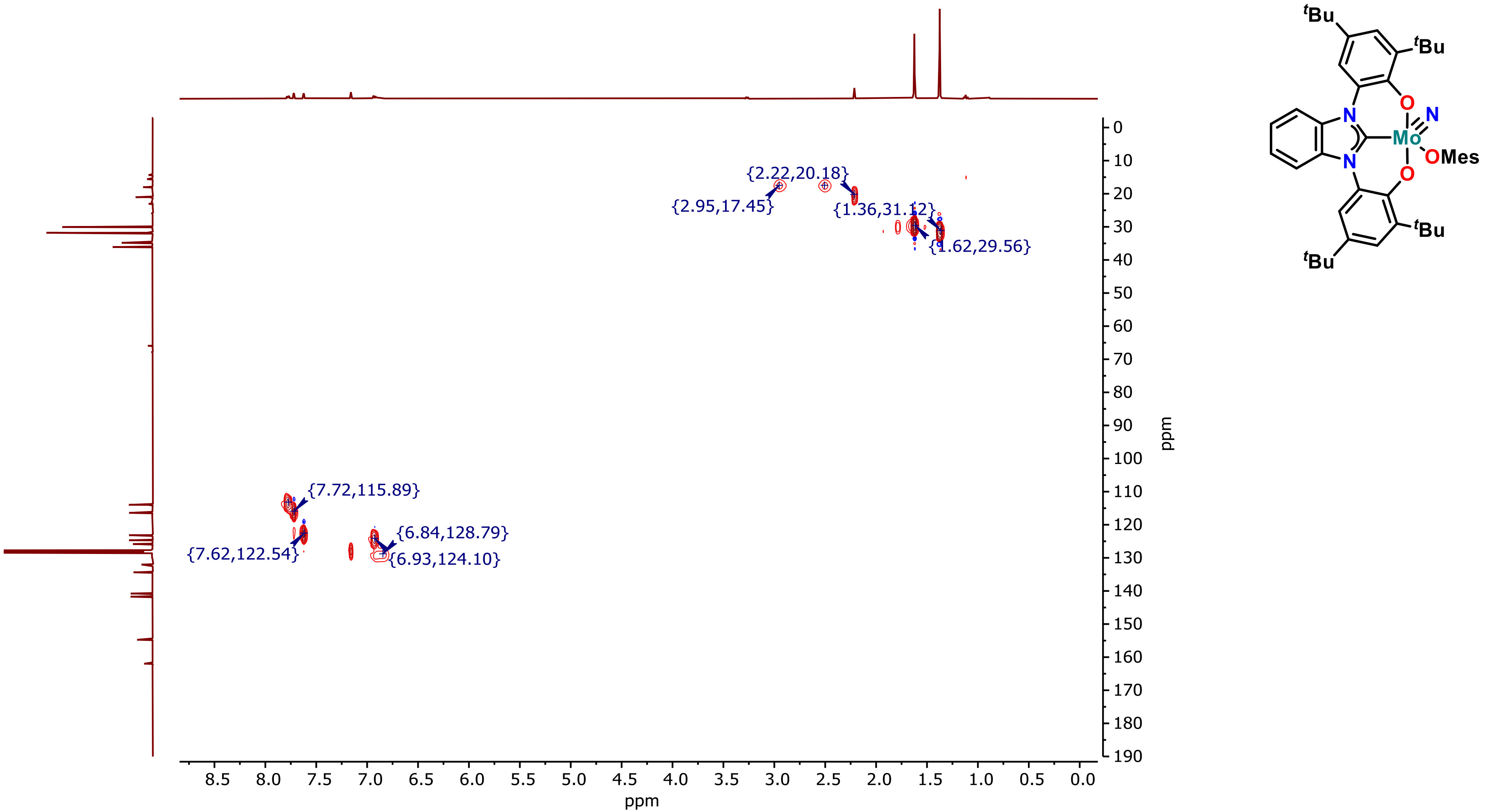

*Figure S 74: $^{1}H-^{13}C$ HSQC of **2-OMes** in C_6D_6 at 298 K.*

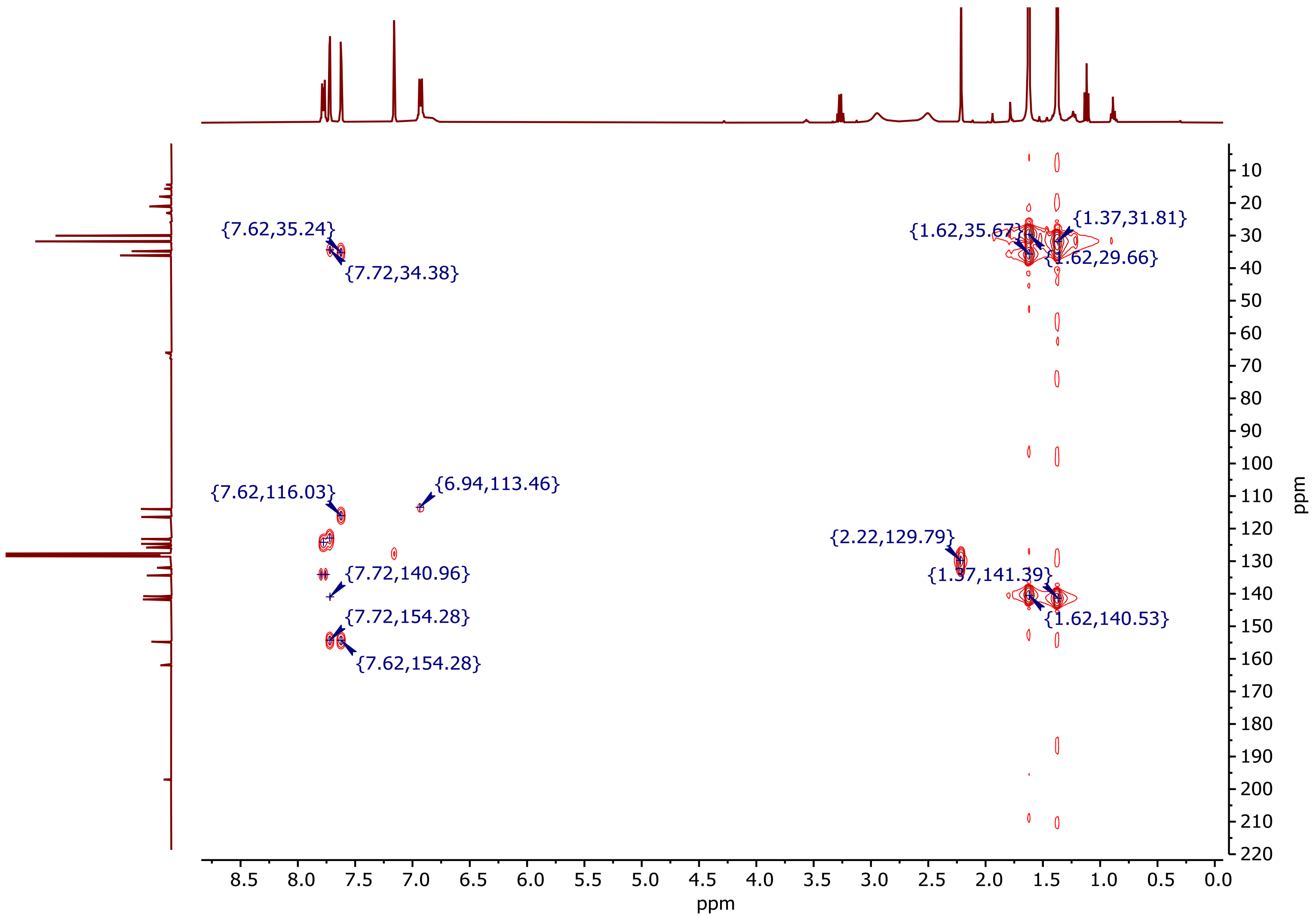

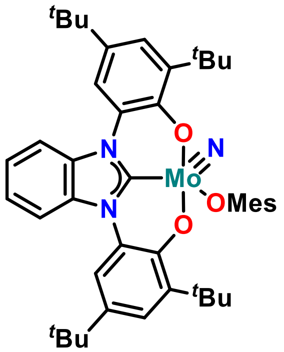

*Figure S 75: ¹H – ¹³C HMBC of **2-OMes** in C_6D_6 at 298 K.*

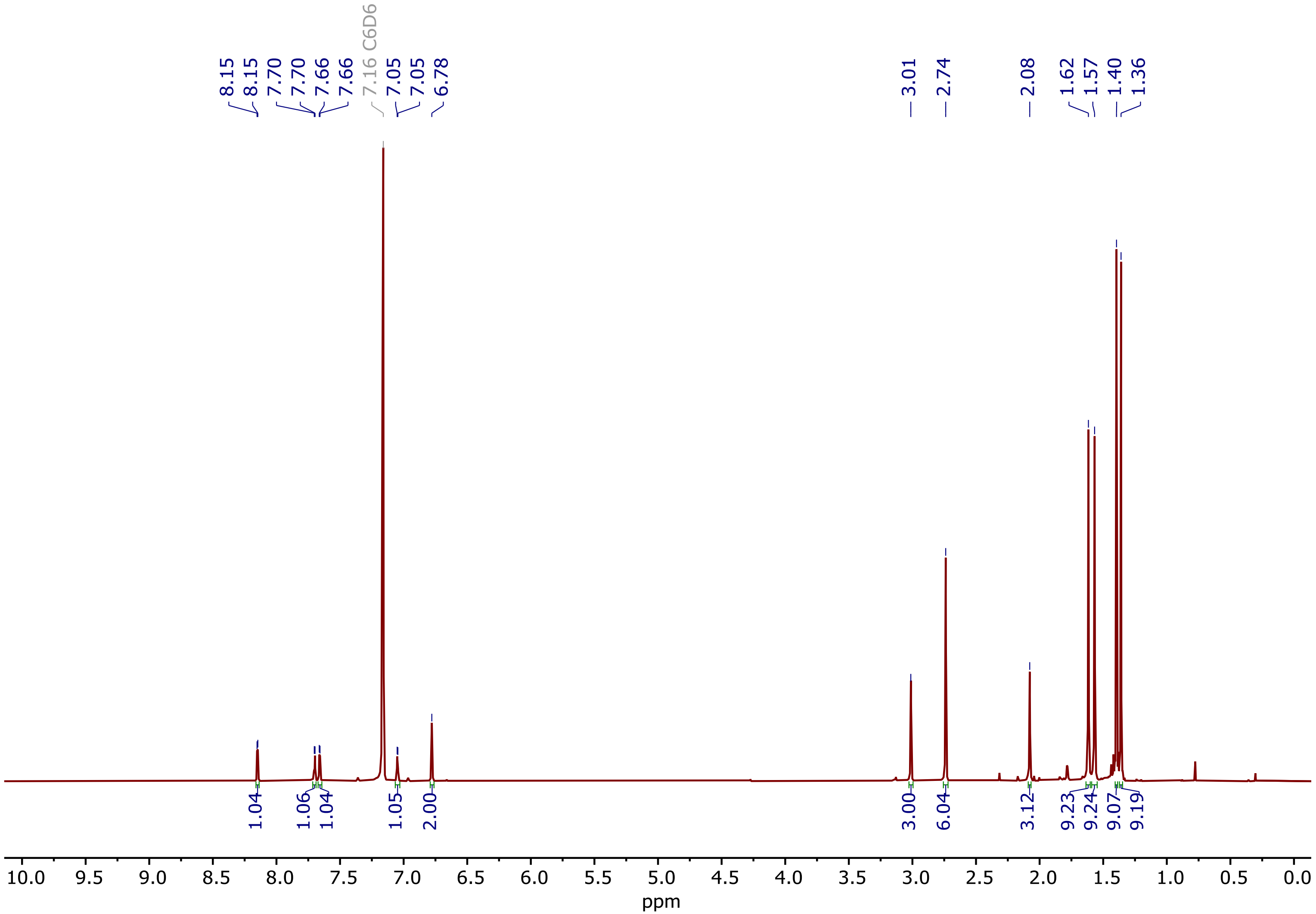

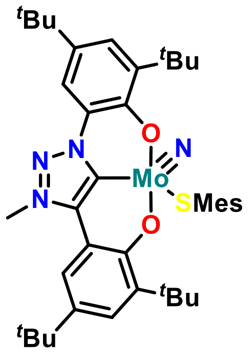

*Figure S 76: ¹H NMR of **1-SMes** in C_6D_6 at 298 K.*

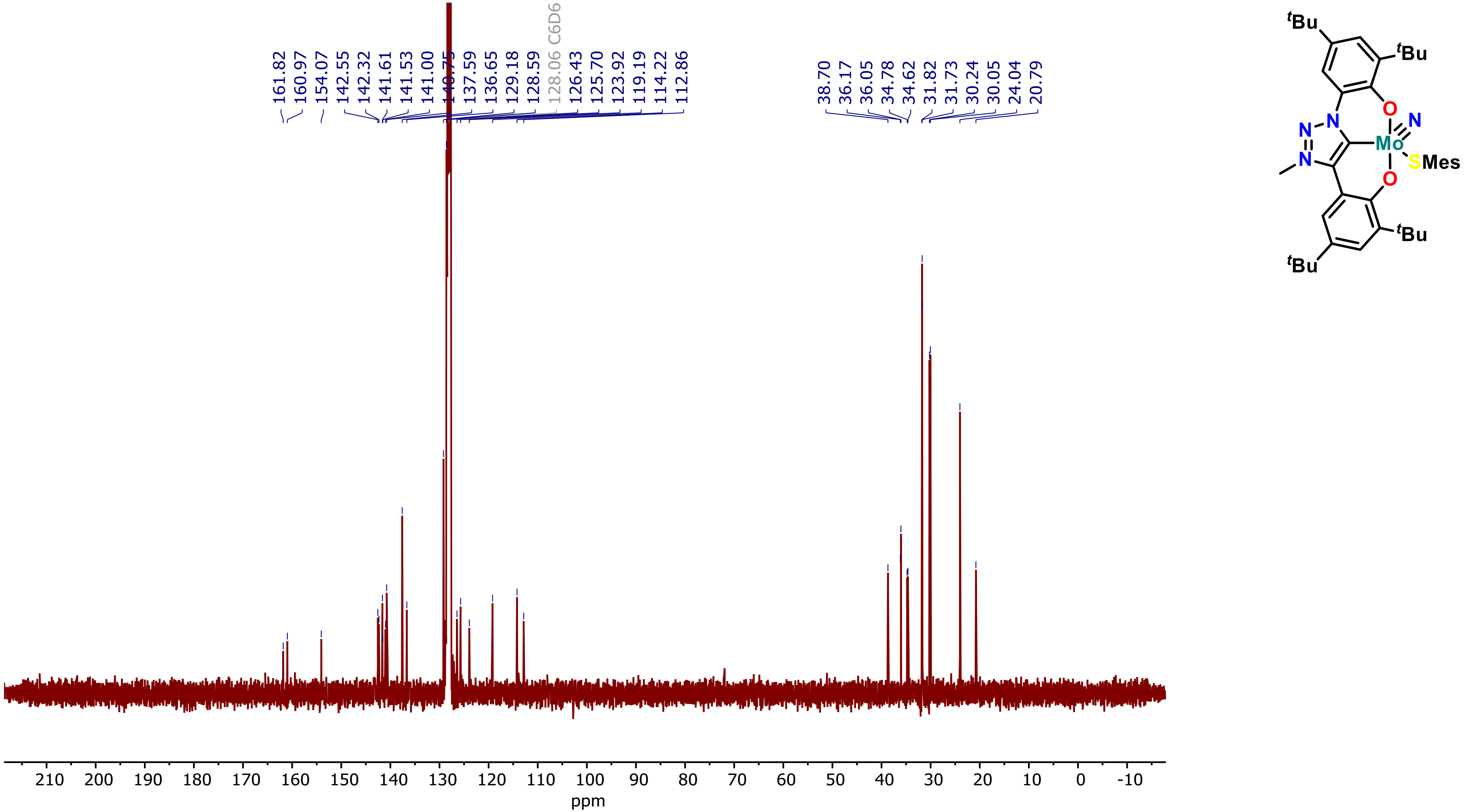

*Figure S 77: ^{13}C NMR of **1-SMes** in C_6D_6 at 298 K.*

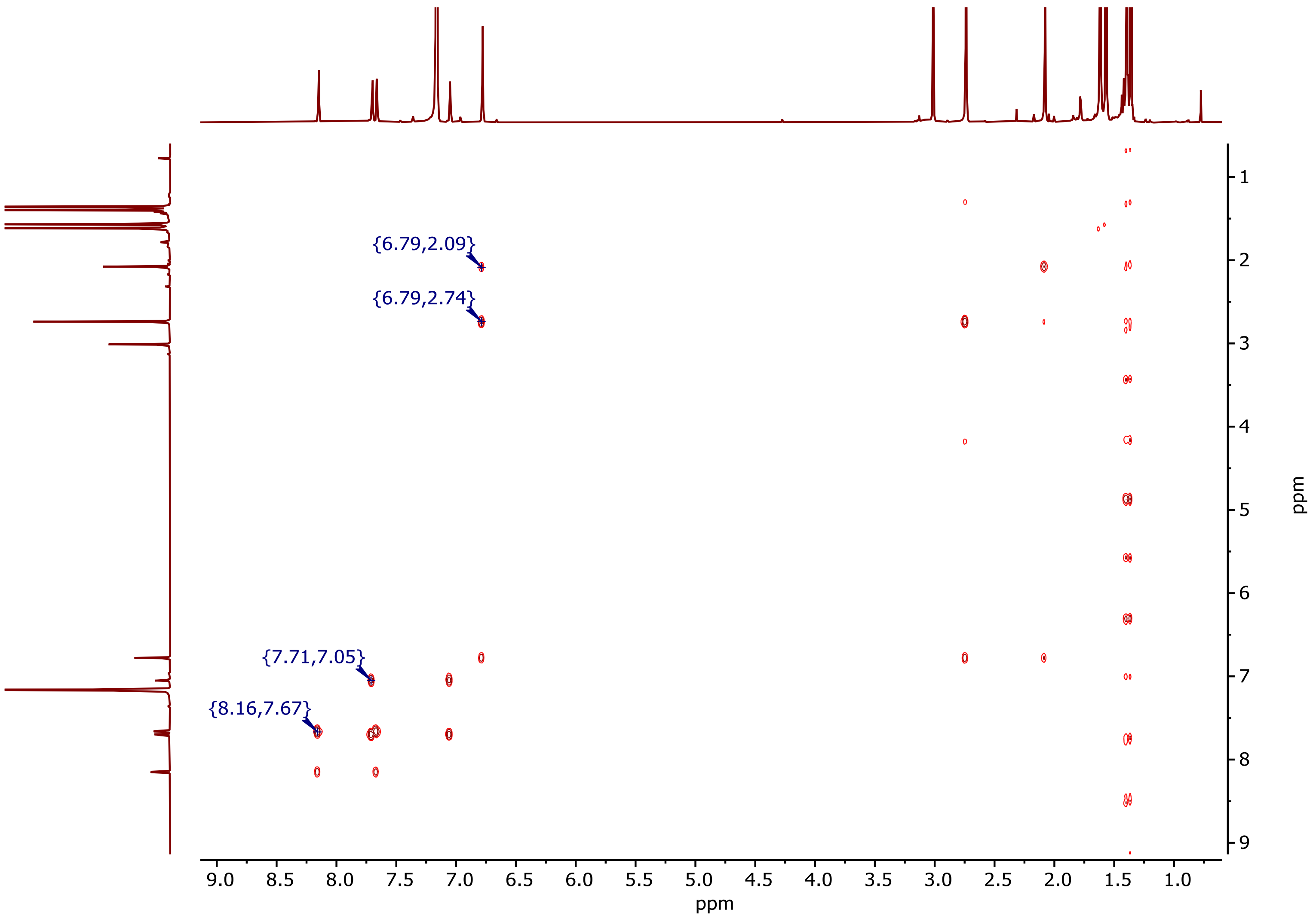

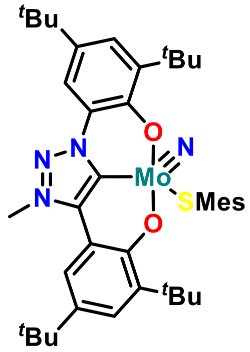

*Figure S 78: ^{1}H – ^{1}H COSY of **1-SMes** in C_6D_6 at 298 K.*

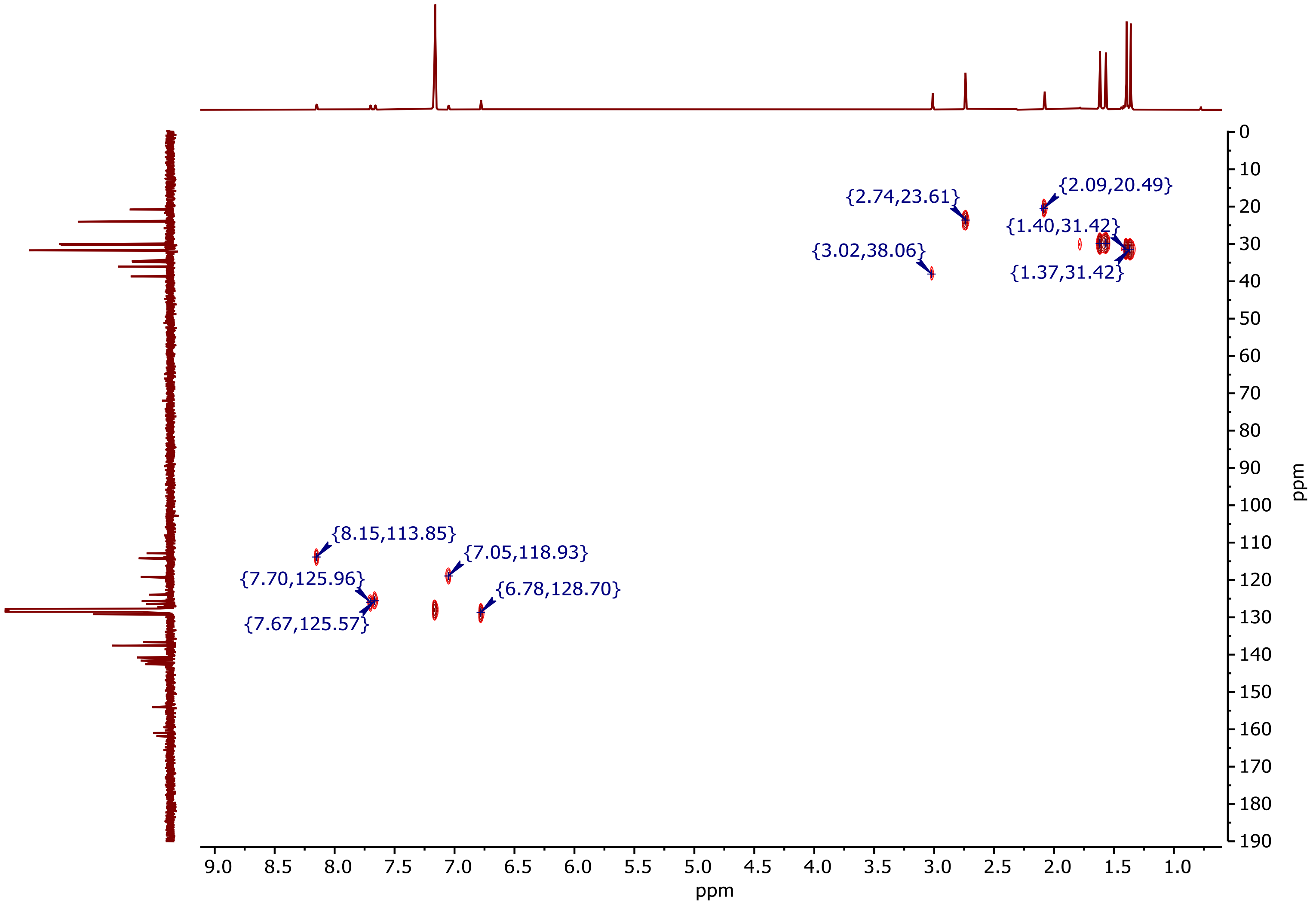

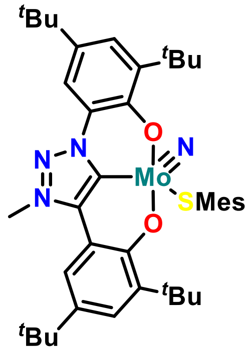

*Figure S 79: ^{1}H – ^{13}C HSQC of **1-SMes** in C_6D_6 at 298 K.*

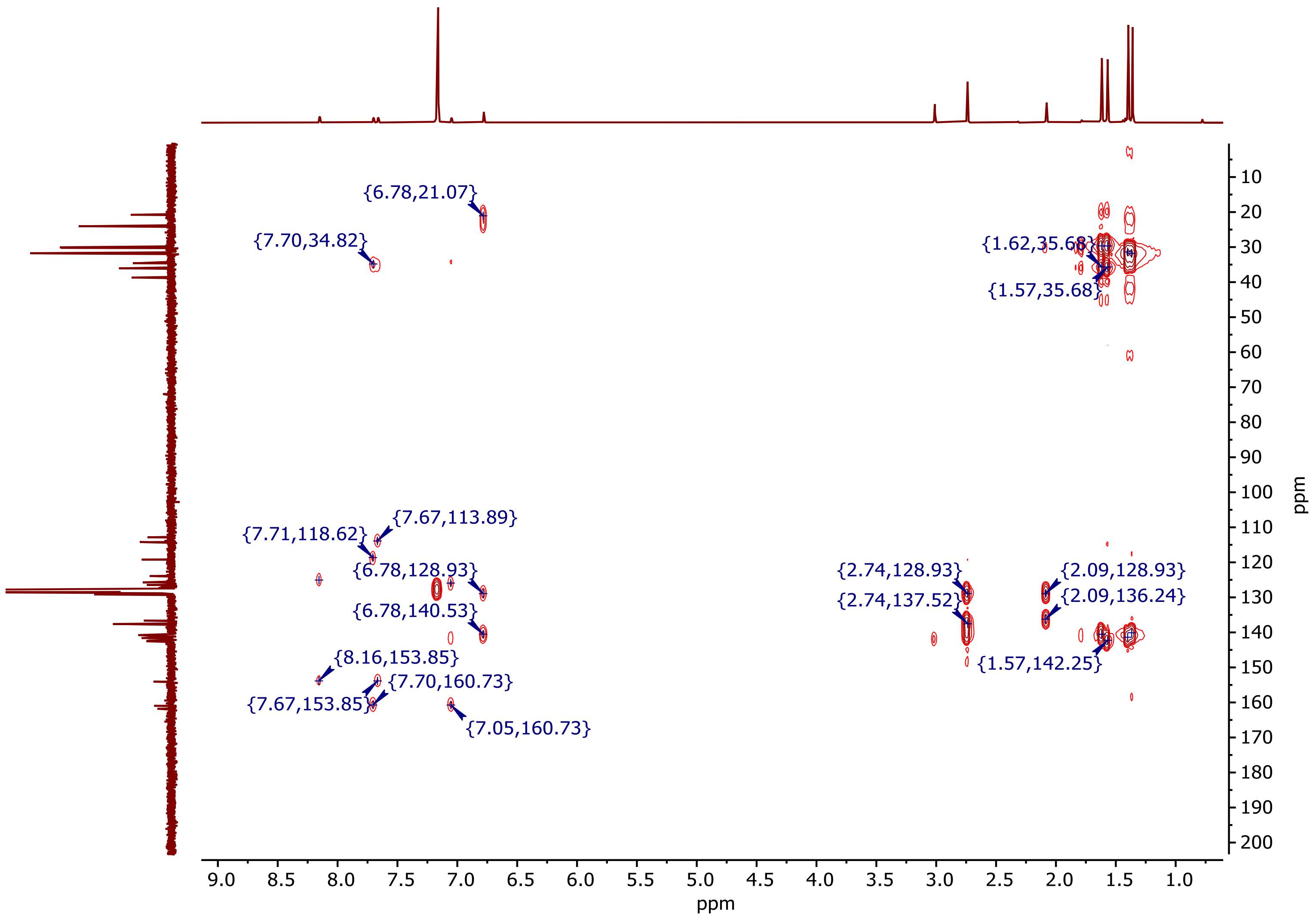

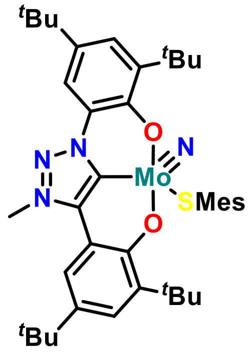

*Figure S 80: $^{1}H - ^{13}C$ HMBC of **1-SMes** in C_6D_6 at 298 K.*

2. IR spectroscopy

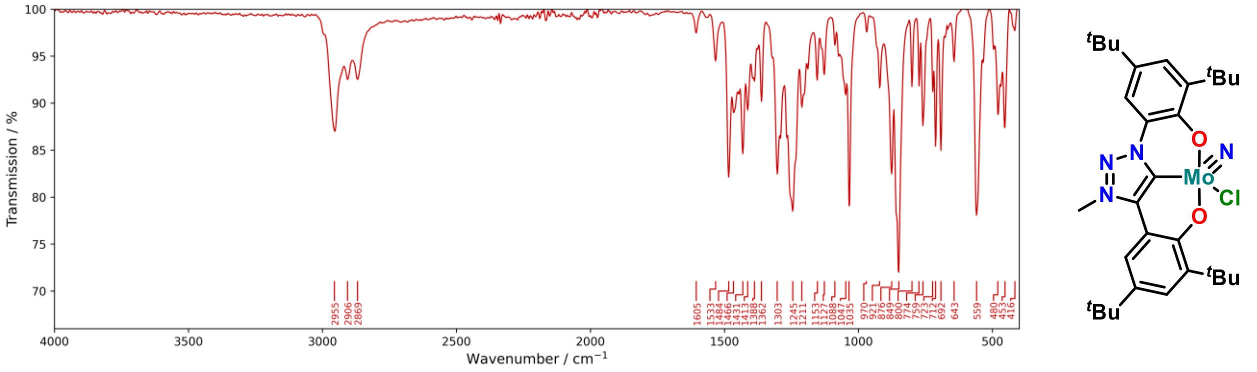

*Figure S 81: ATR-IR spectrum of **1-Cl** at 298 K.*

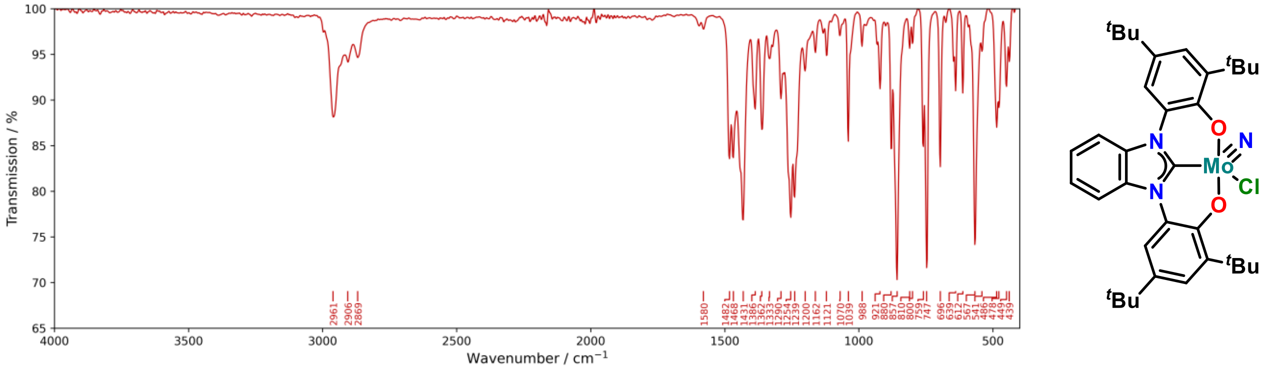

*Figure S 82: ATR-IR spectrum of **2-Cl** at 298 K.*

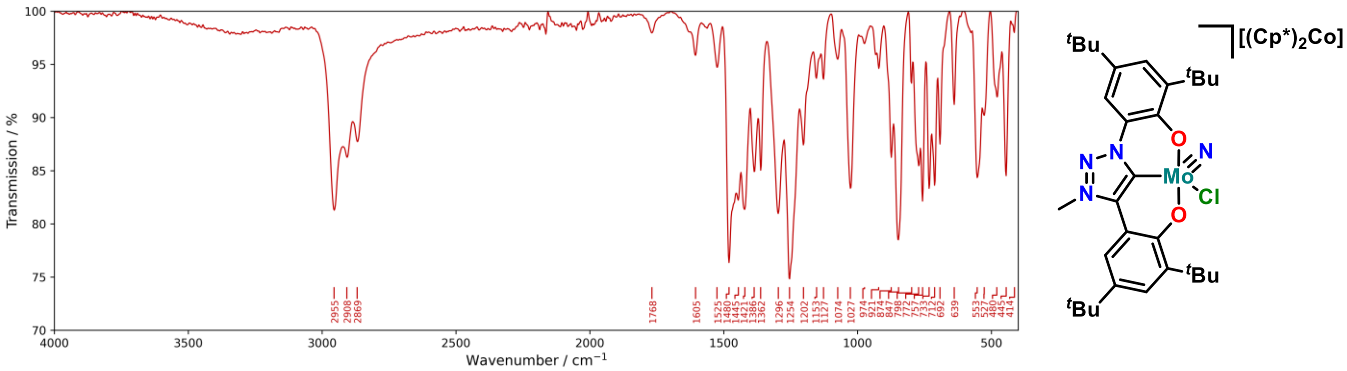

*Figure S 83: ATR-IR spectrum of **[1-Cl]**⁻ at 298 K.*

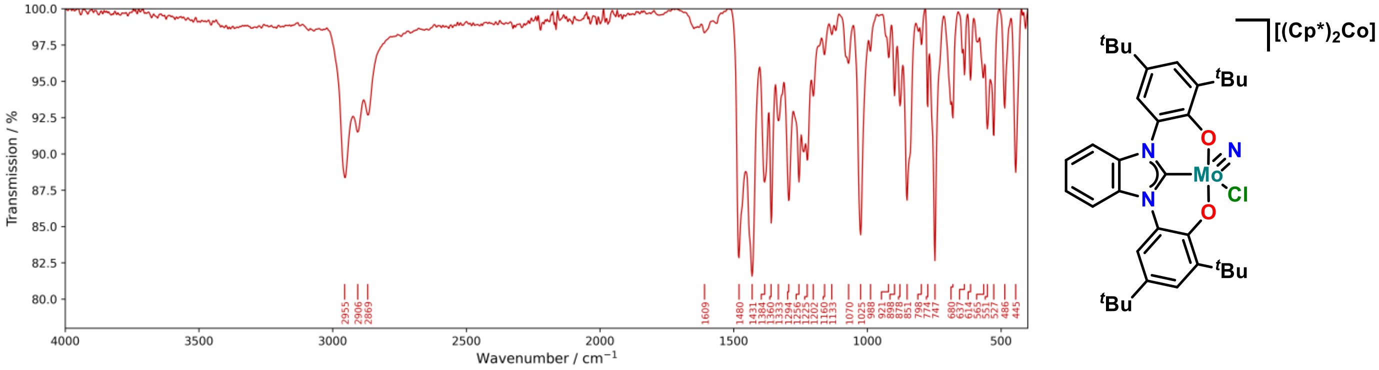

Figure S 84: ATR-IR spectrum of ***[2-Cl]****$^-$ at 298 K.*

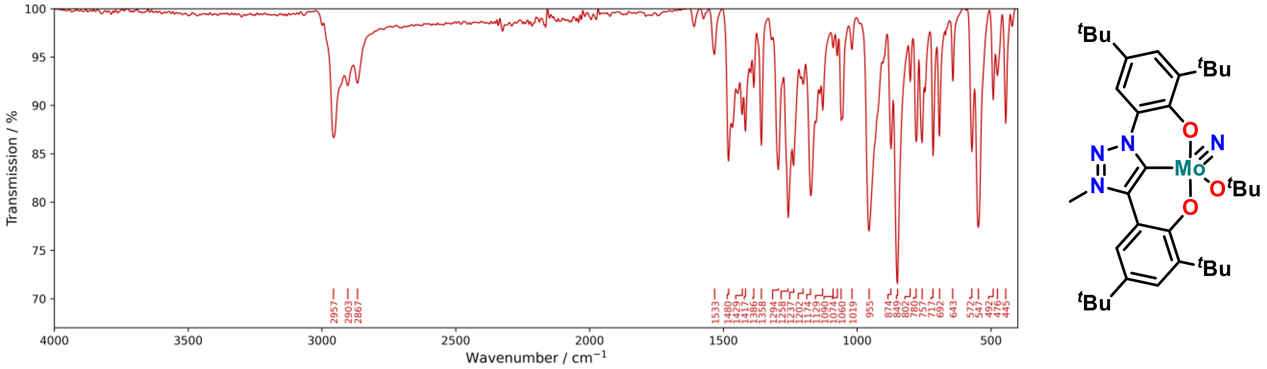

Figure S 85: ATR-IR spectrum of ***1-O^{t}Bu*** *at 298 K.*

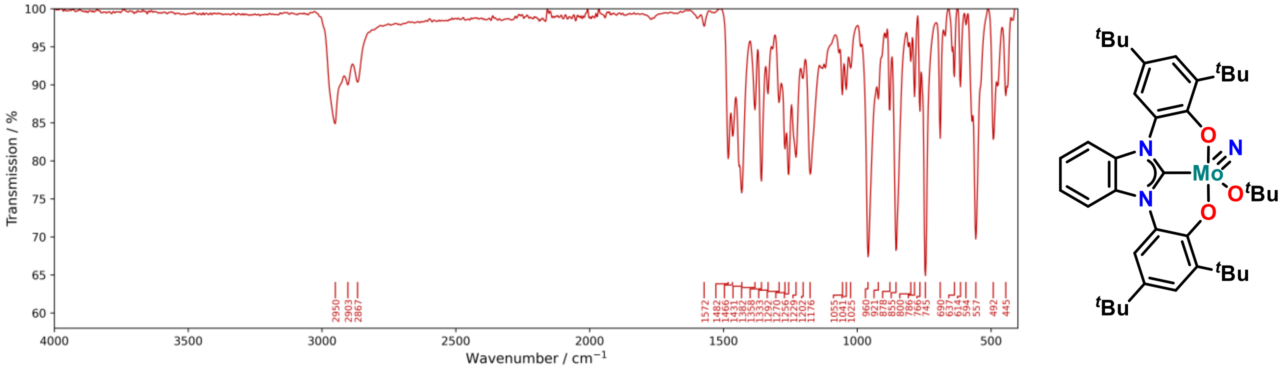

Figure S 86: ATR-IR spectrum of ***2-O^{t}Bu*** *at 298 K.*

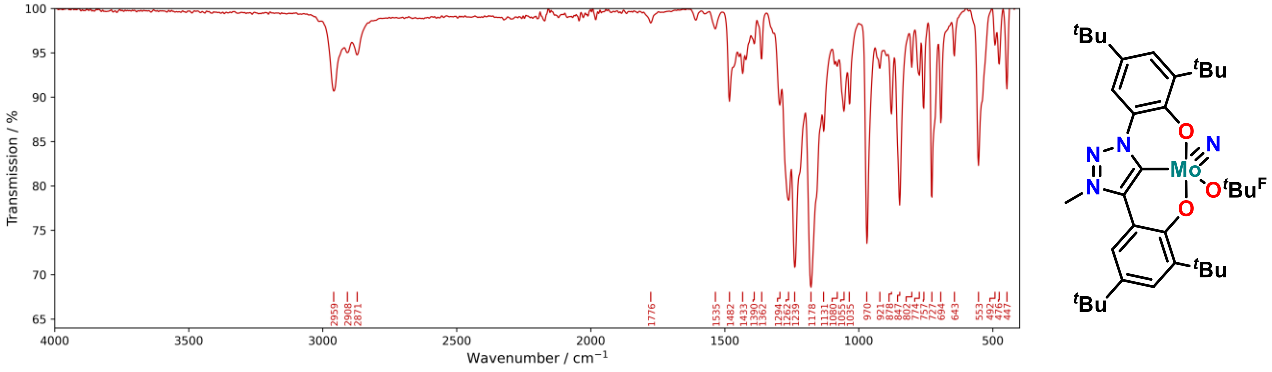

Figure S 87: ATR-IR spectrum of ***1-O^{t}Bu^{F9}*** *at 298 K.*

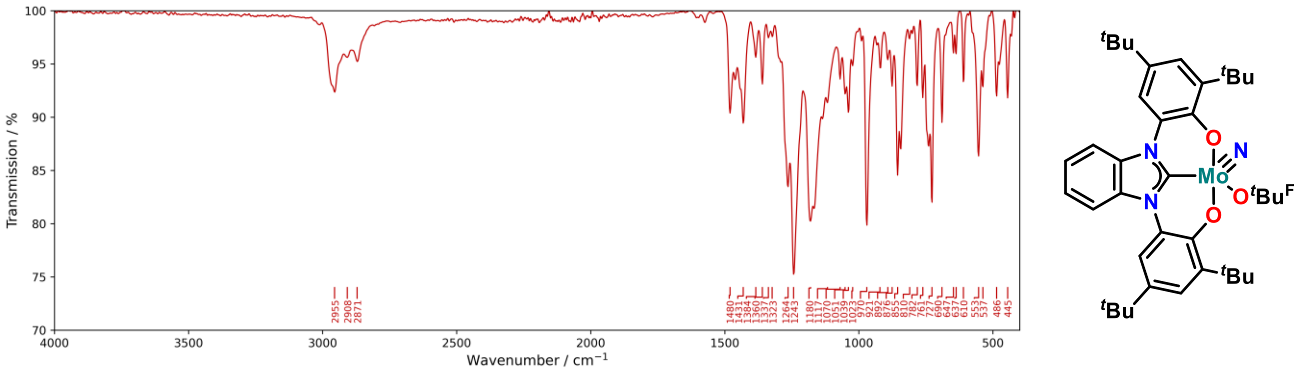

Figure S 88: ATR-IR spectrum of ***2-O^{t}Bu^{F9}*** *at 298 K.*

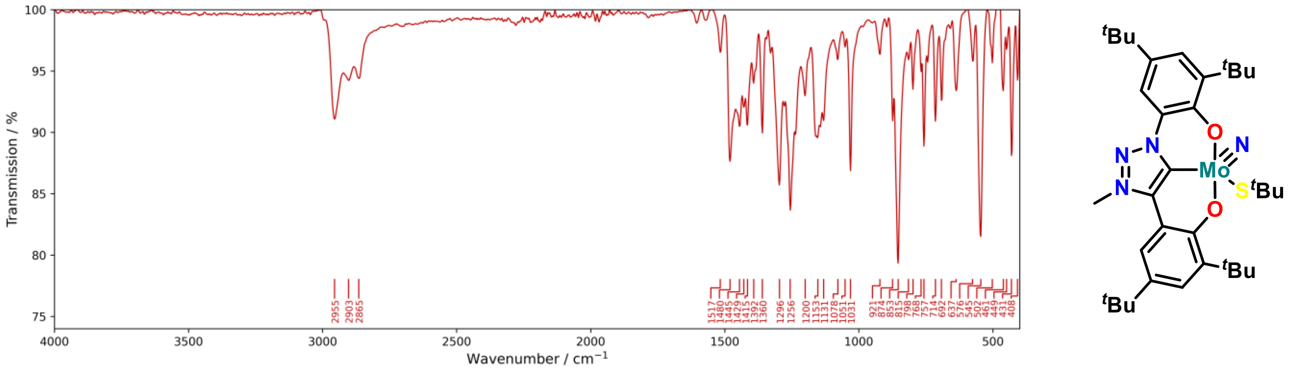

Figure S 89: ATR-IR spectrum of ***1-S^{t}Bu*** *at 298 K.*

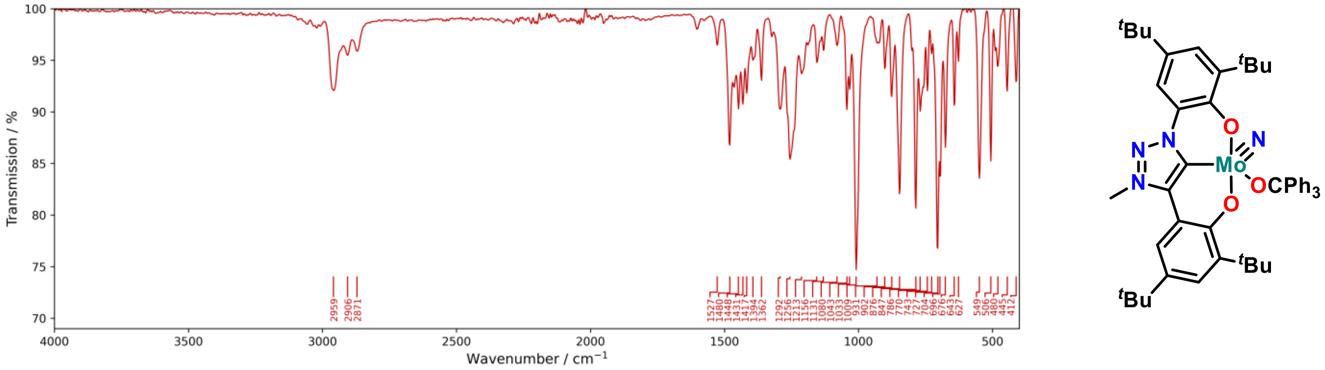

Figure S 90: ATR-IR spectrum of ***1-OCPh$_3$*** *at 298 K.*

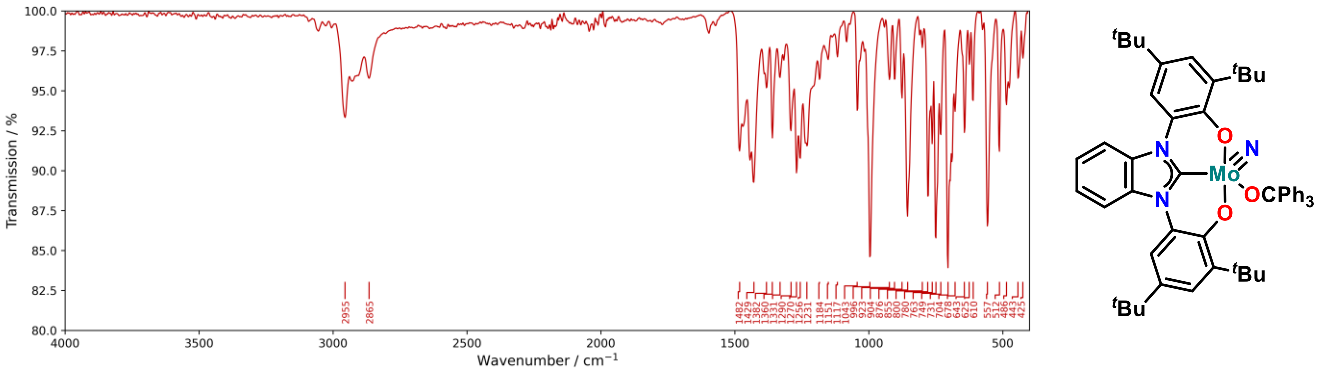

Figure S 91: ATR-IR spectrum of ***2-OCPh$_3$*** *at 298 K.*

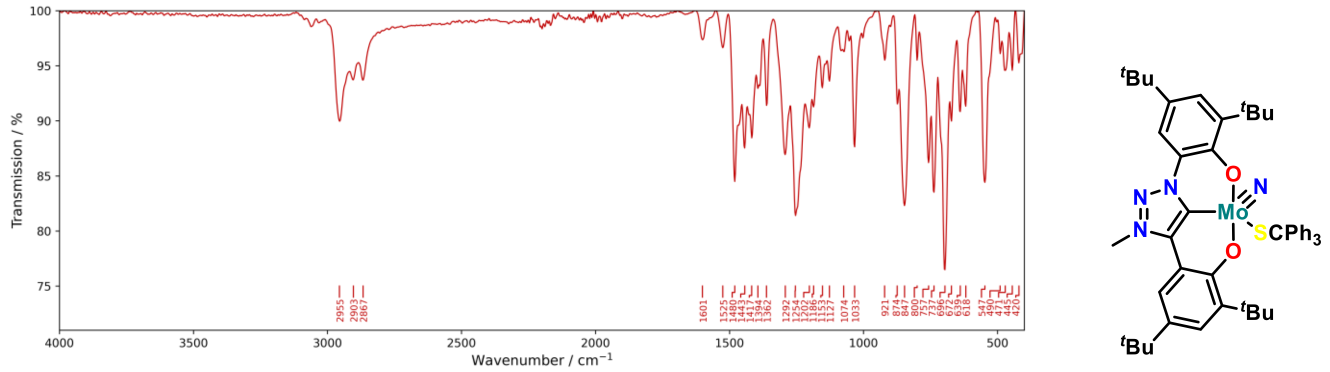

*Figure S 92: ATR-IR spectrum of **1-SCPh$_3$** at 298 K.*

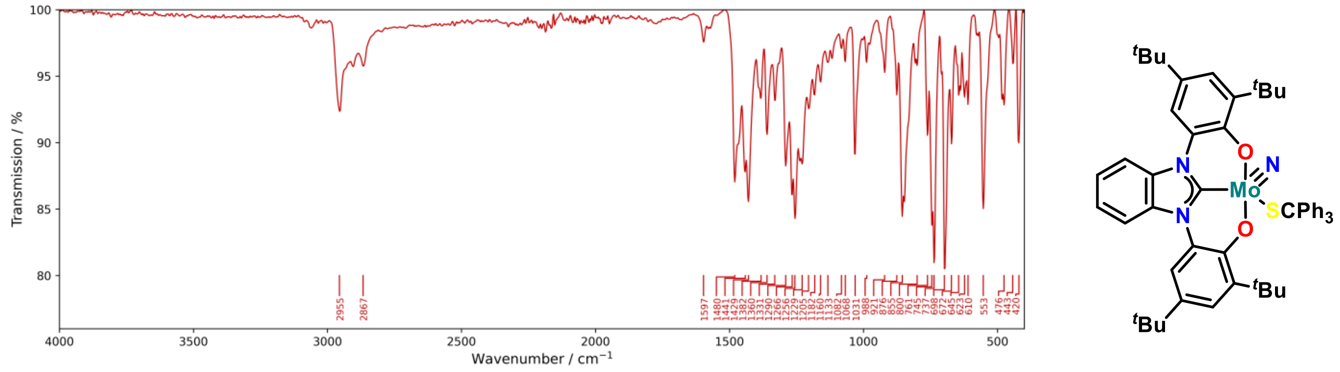

*Figure S 93: ATR-IR spectrum of **2-SCPh$_3$** at 298 K.*

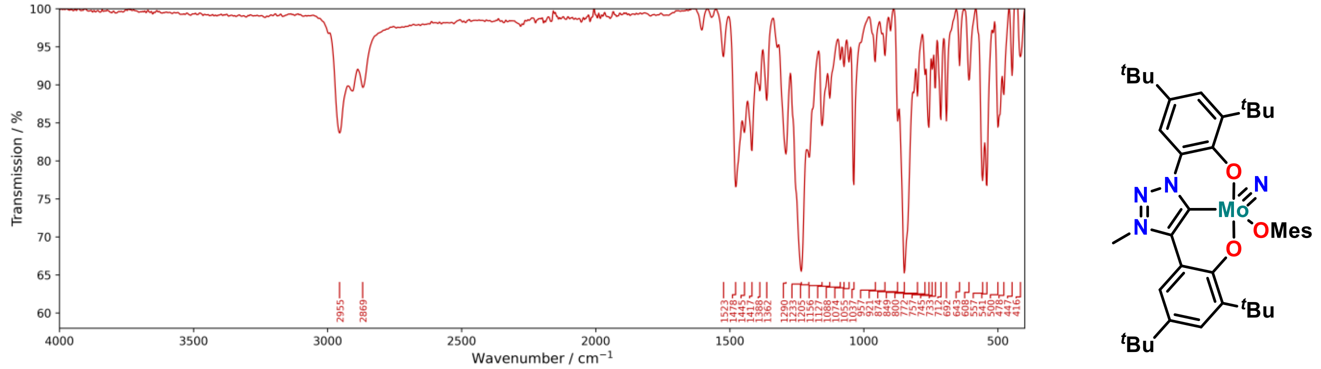

*Figure S 94: ATR-IR spectrum of **1-OMes** at 298 K.*

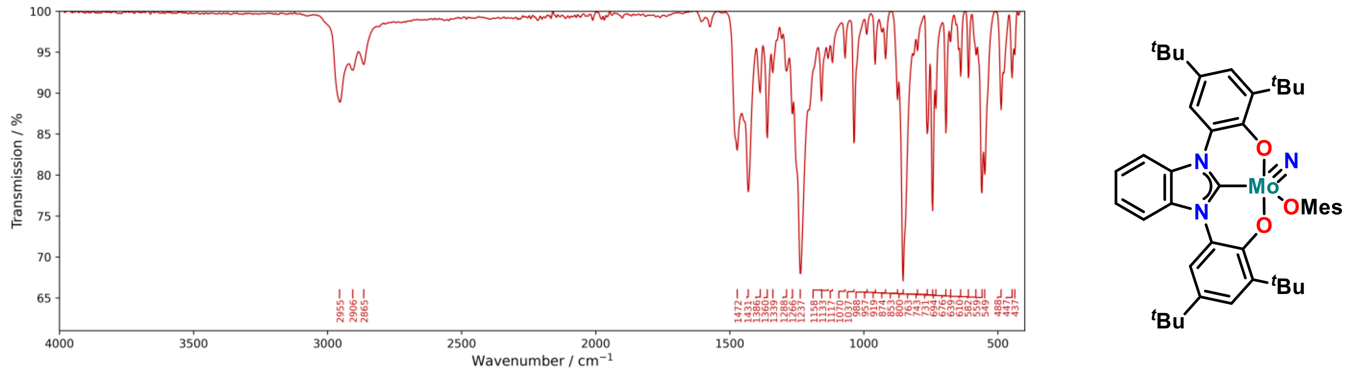

*Figure S 95: ATR-IR spectrum of **2-OMes** at 298 K.*

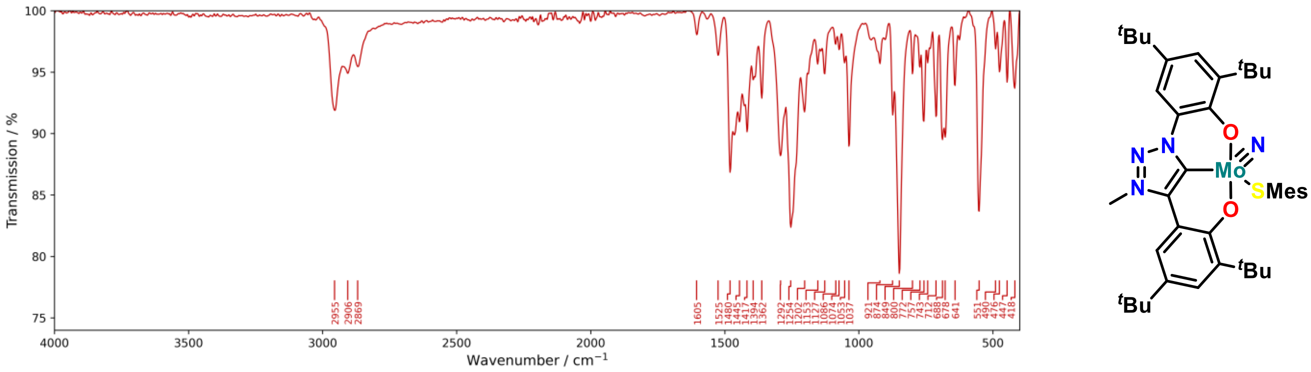

*Figure S 96: ATR-IR spectrum of **1-SMes** at 298 K.*

3. UV-Vis Spectroscopy

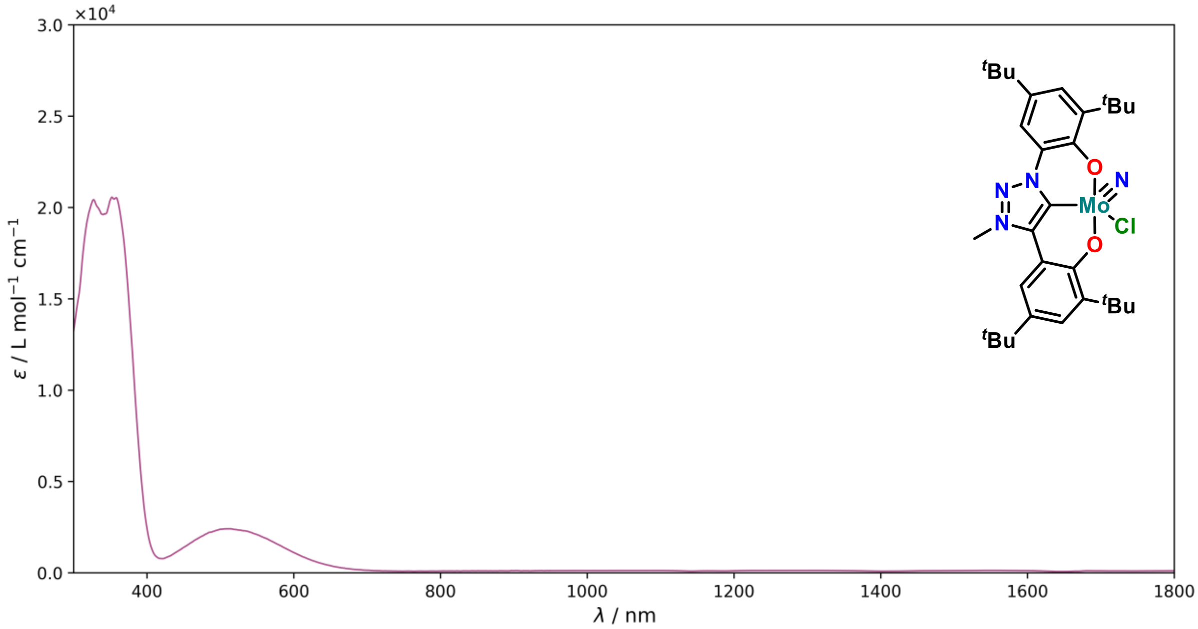

*Figure S 97: UV-VIS-NIR spectrum of **1-Cl** in CH_2Cl_2 at 298 K.*

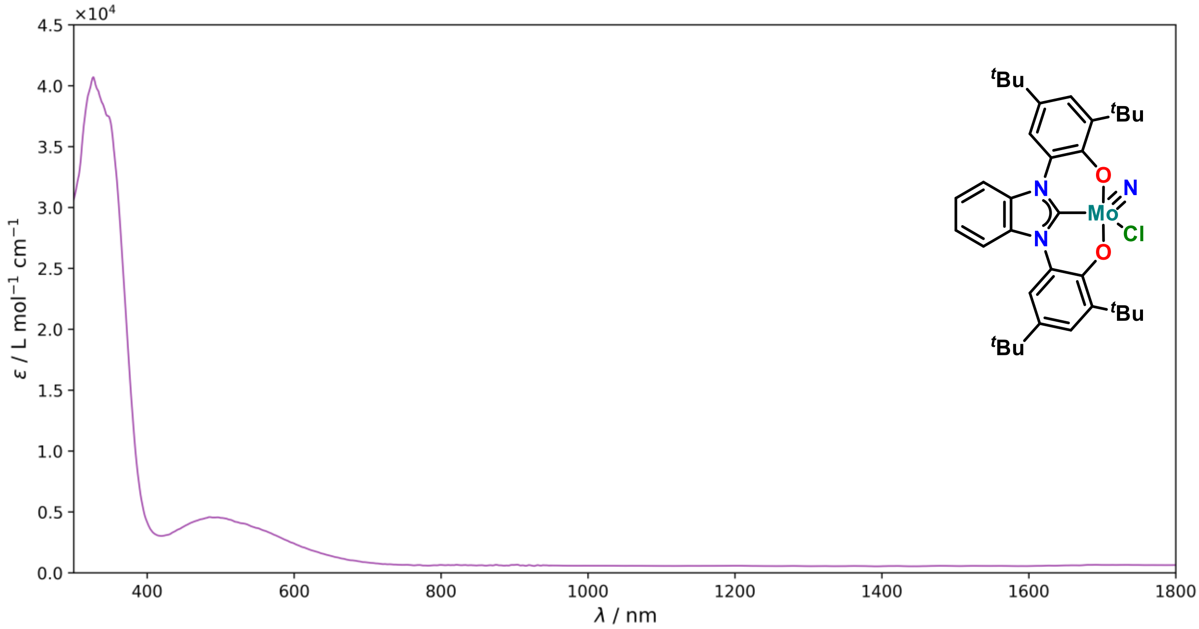

*Figure S 98: UV-VIS-NIR spectrum of **2-Cl** in CH_2Cl_2 at 298 K.*

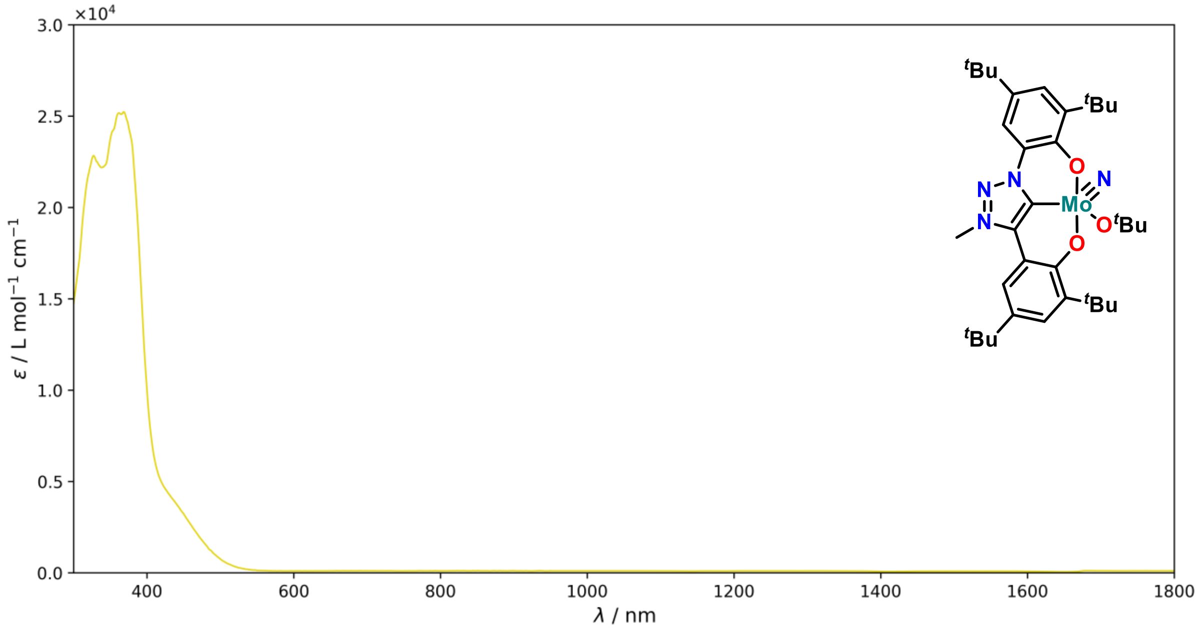

*Figure S 99: UV-VIS-NIR spectrum of **1-O^{t}Bu** in toluene at 298 K.*

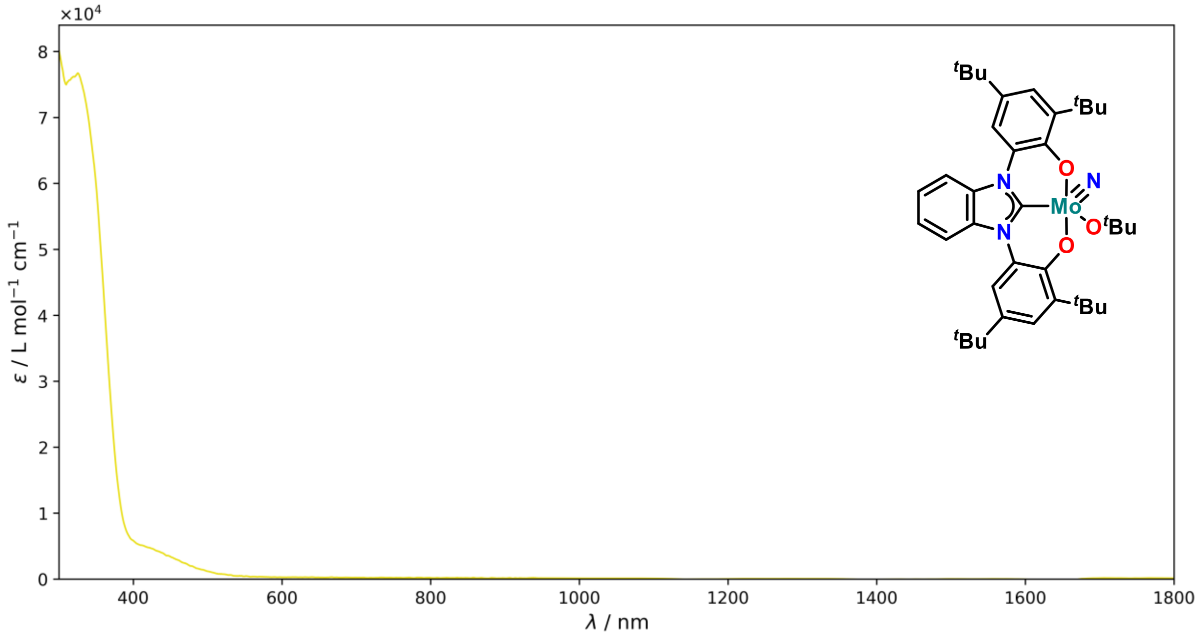

*Figure S 100: UV-VIS-NIR spectrum of **2-O^{t}Bu** in toluene at 298 K.*

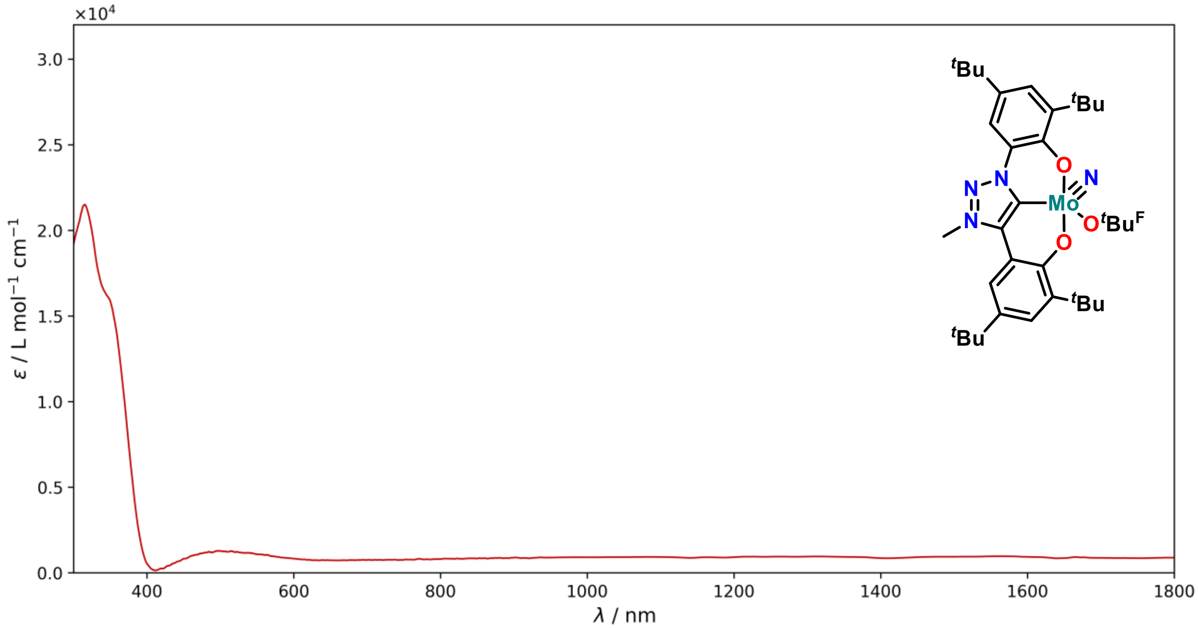

*Figure S 101: UV-VIS-NIR spectrum of **1-O^{t}Bu^{F9}** in toluene at 298 K.*

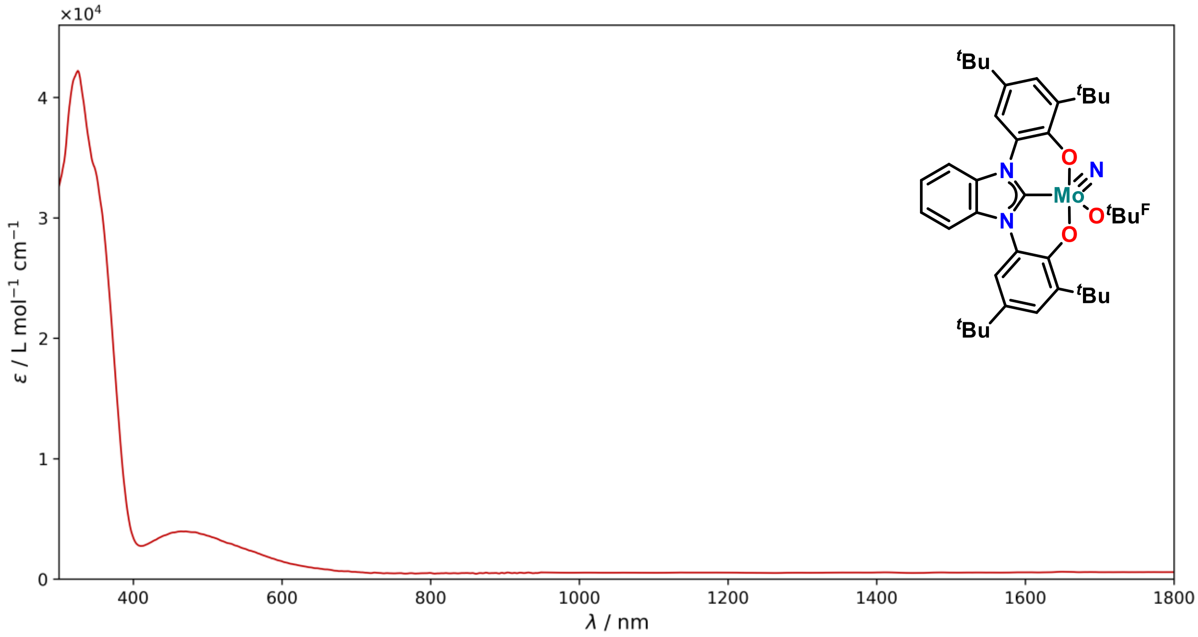

*Figure S 102: UV-VIS-NIR spectrum of **2-O^{t}Bu^{F9}** in toluene at 298 K.*

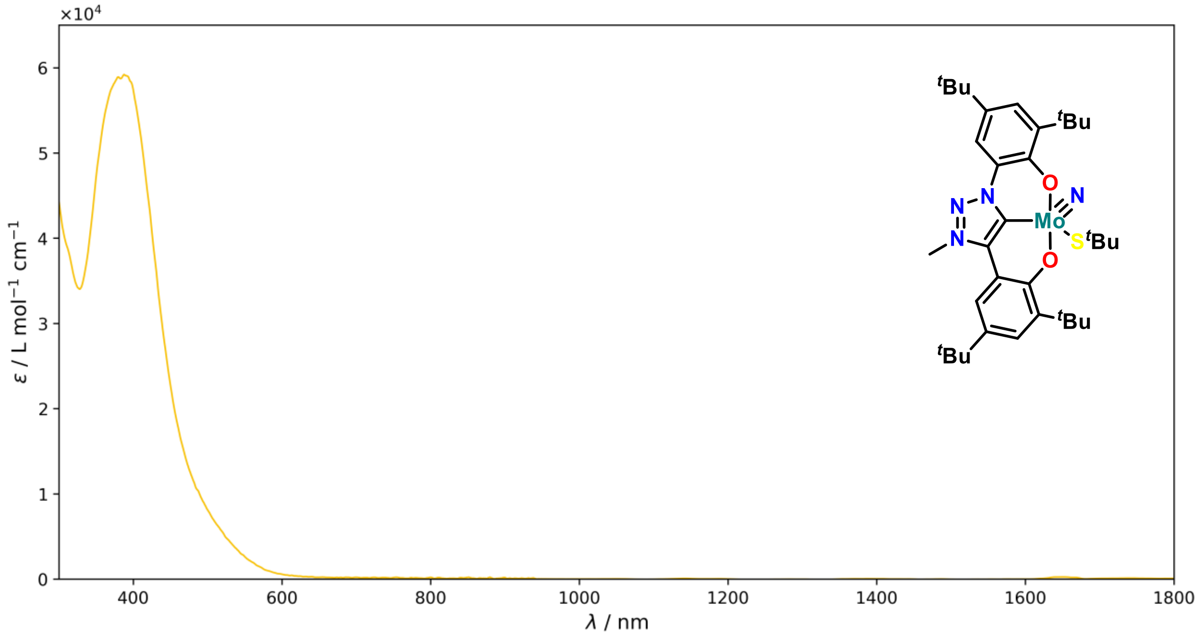

Figure S 103: UV-VIS-NIR spectrum of ***1-S^{t}Bu*** *in toluene at 298 K.*

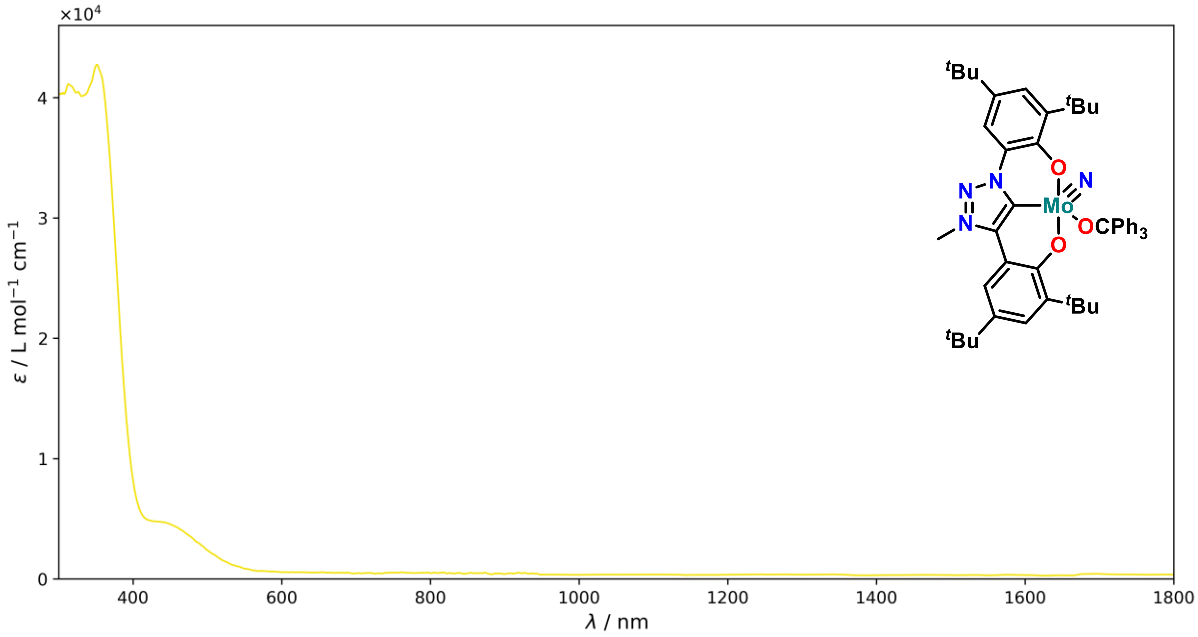

Figure S 104: UV-VIS-NIR spectrum of ***1-OCPh$_3$*** *in toluene at 298 K.*

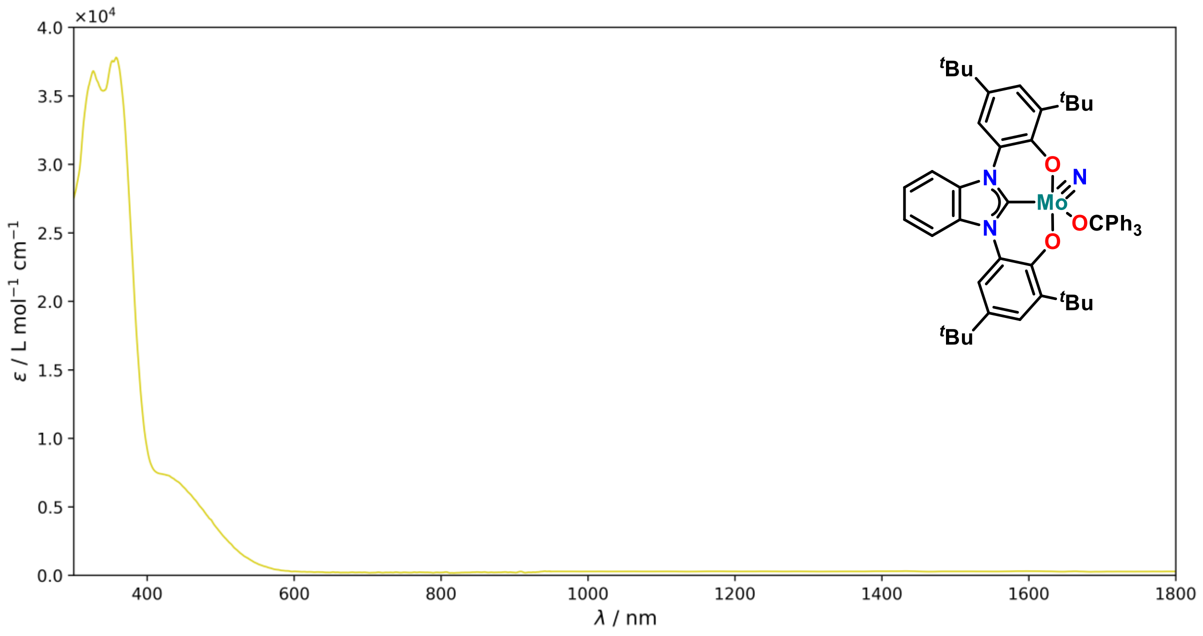

*Figure S 105: UV-VIS-NIR spectrum of **2-OCPh₃** in toluene at 298 K.*

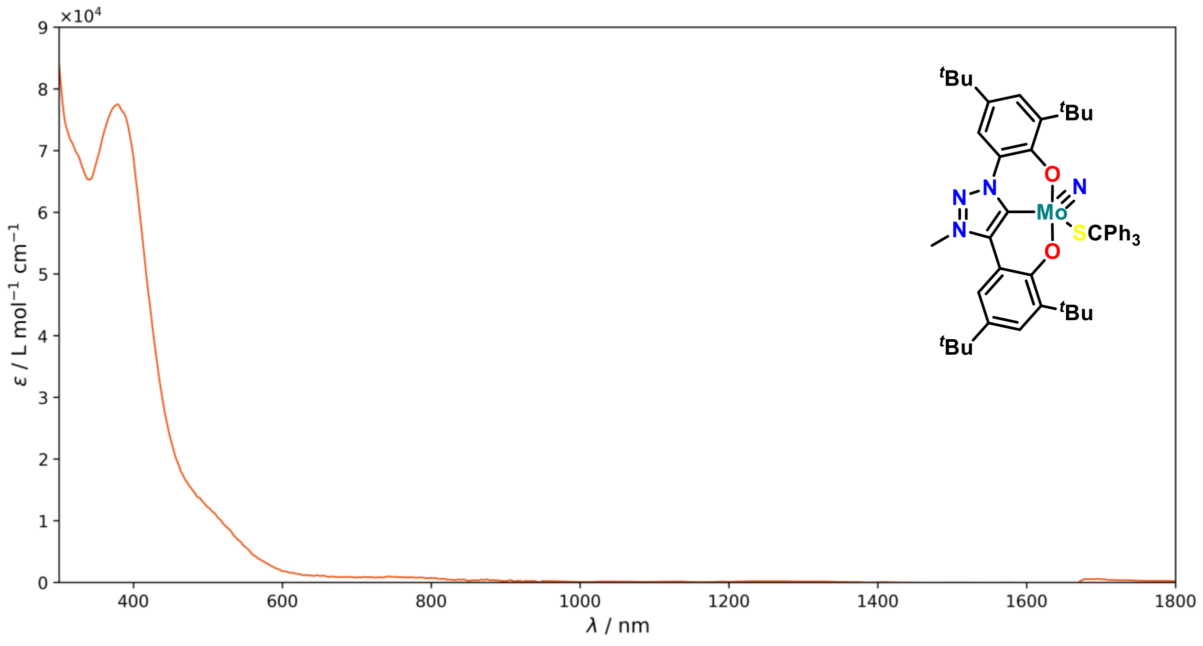

*Figure S 106: UV-VIS-NIR spectrum of **1-SCPh₃** in toluene at 298 K.*

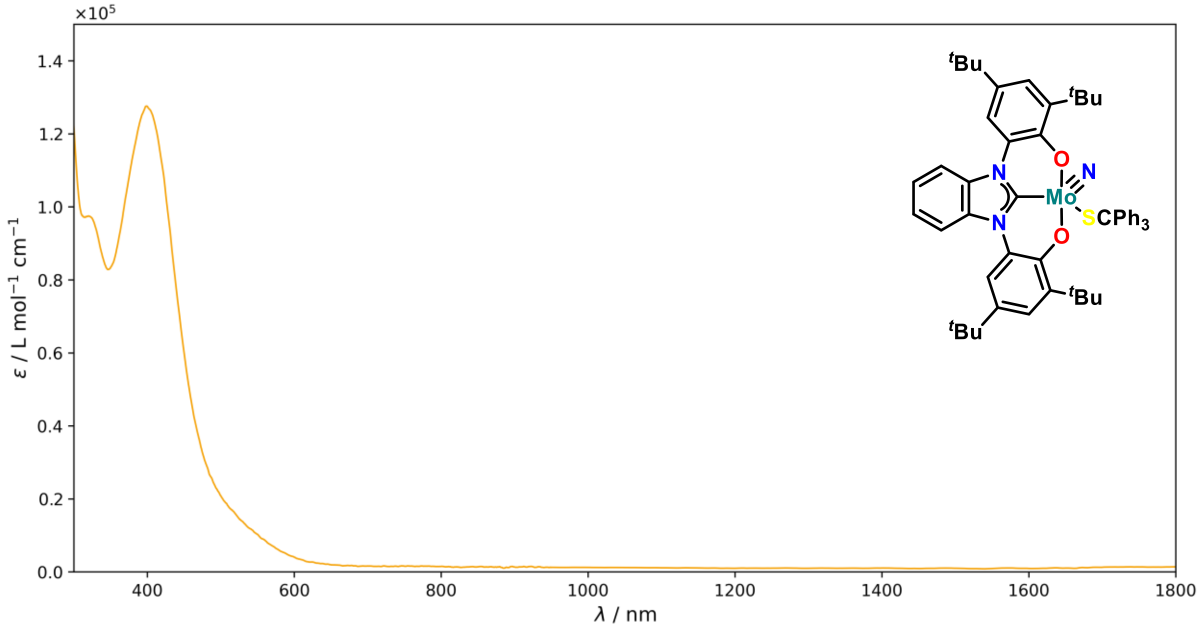

Figure S 107: UV-VIS-NIR spectrum of ***2-SCPh$_3$*** *in toluene at 298 K.*

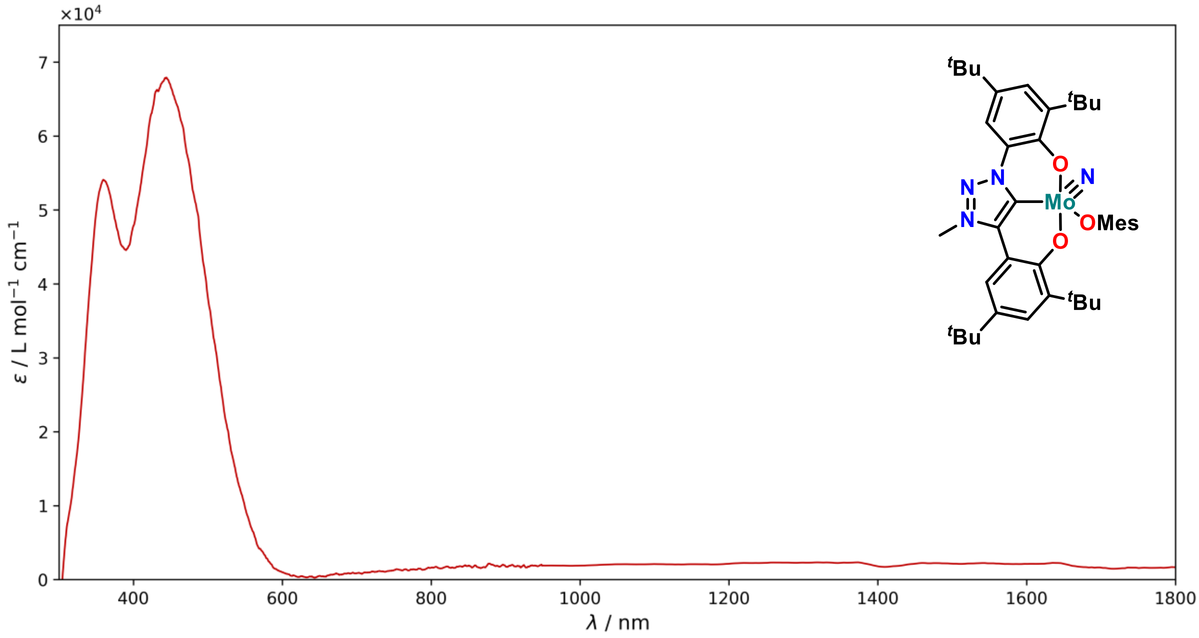

Figure S 108: UV-VIS-NIR spectrum of ***1-OMes*** *in toluene at 298 K.*

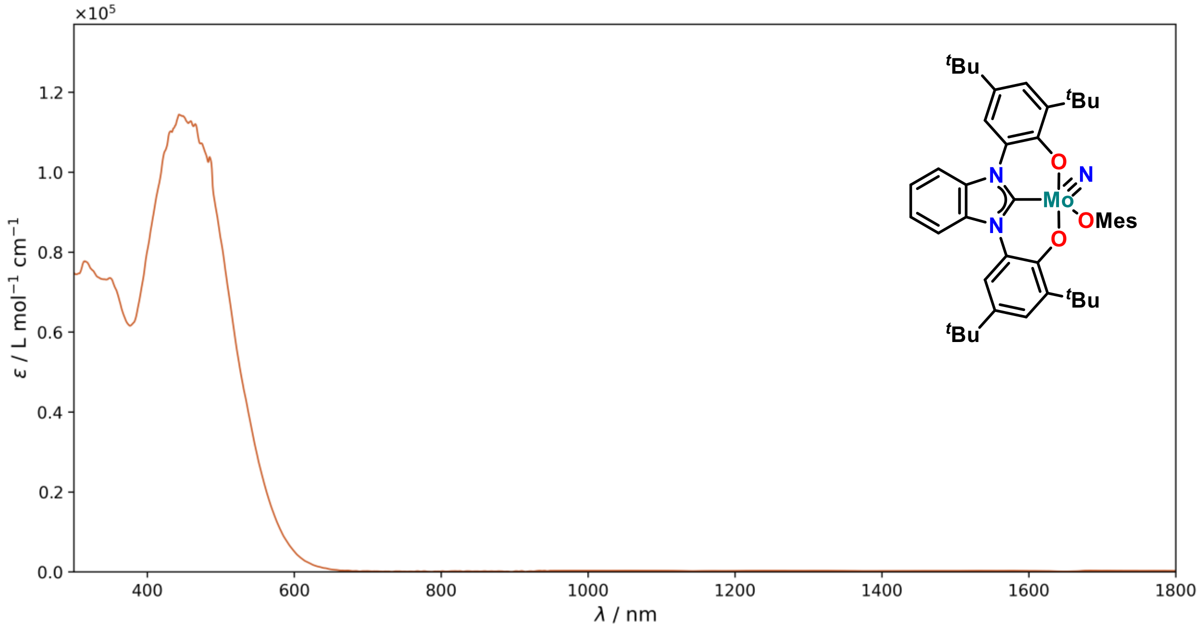

*Figure S 109: UV-VIS-NIR spectrum of **2-OMes** in toluene at 298 K.*

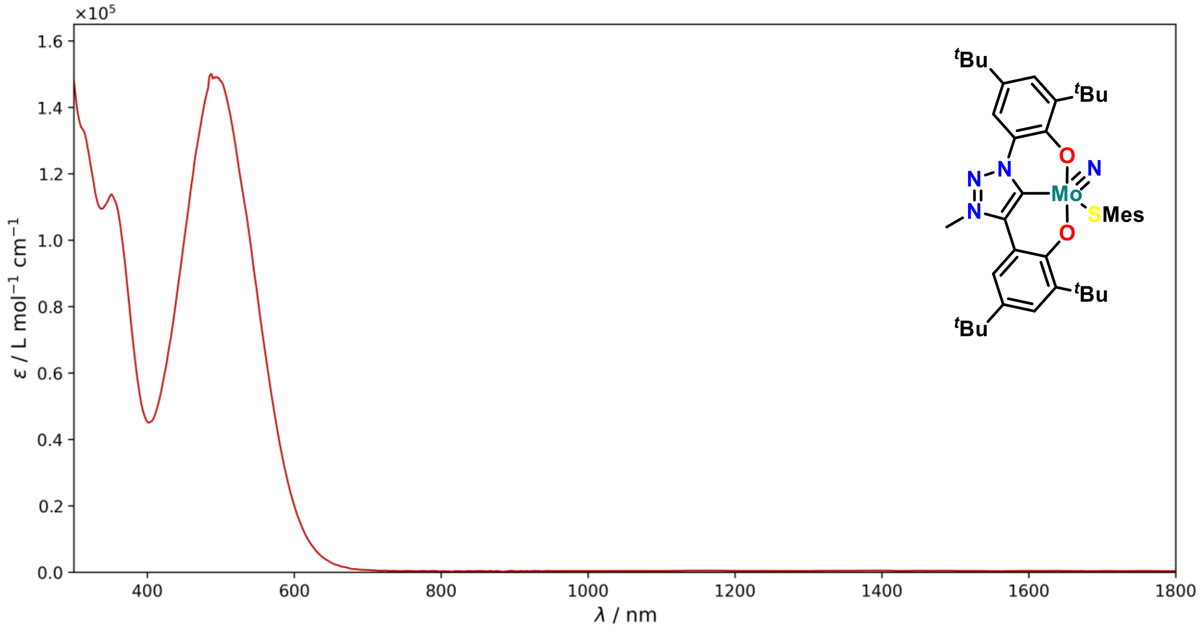

*Figure S 110: UV-VIS-NIR spectrum of **1-SMes** in toluene at 298 K.*

4. Cyclic Voltammetry

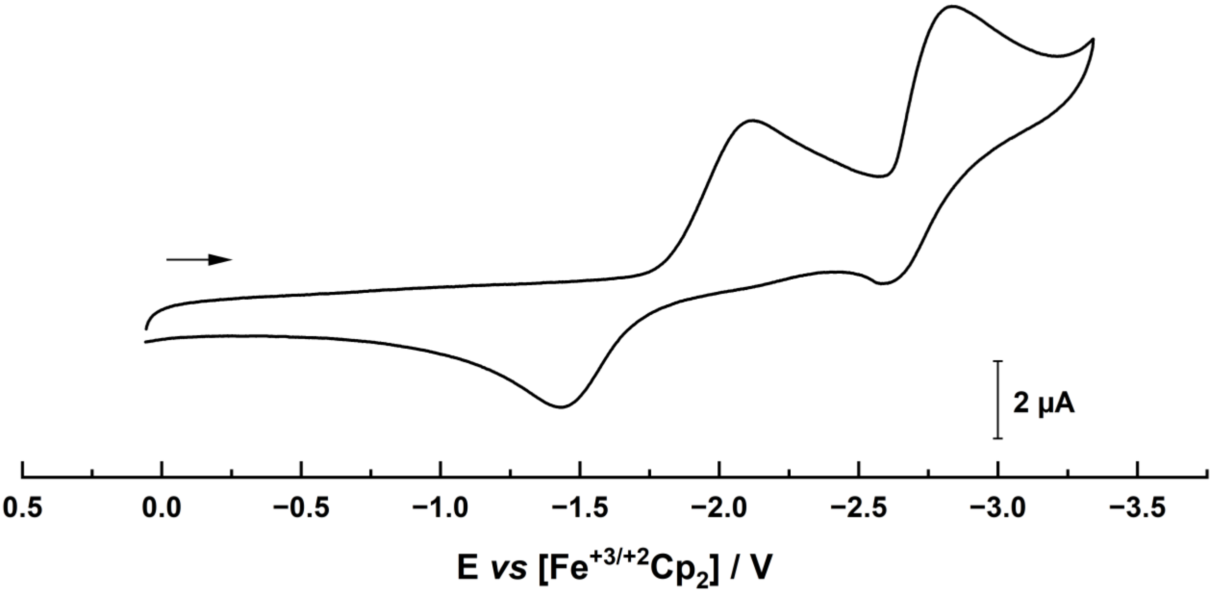

*Figure S 111: Cyclic voltammogram of **1-O^{t}Bu** in 0.15 M NBu_4PF_6 THF solution at a scan rate of 100 mV s^{-1}.*

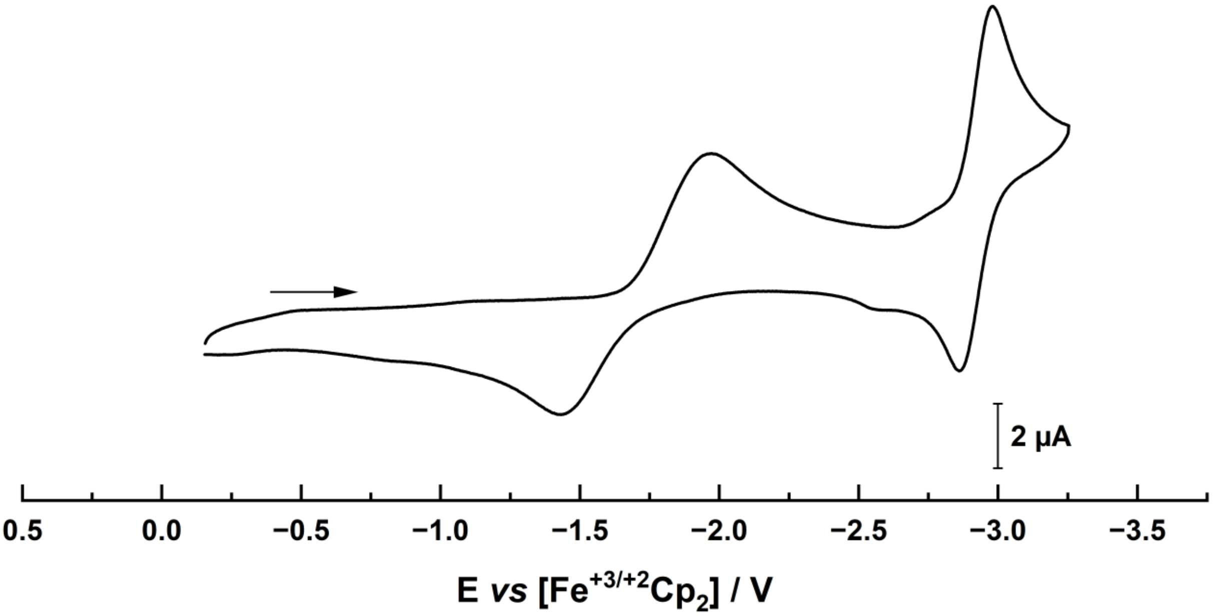

*Figure S 112: Cyclic voltammogram of **2-O^{t}Bu** in 0.15 M NBu_4PF_6 THF solution at a scan rate of 100 mV s^{-1}.*

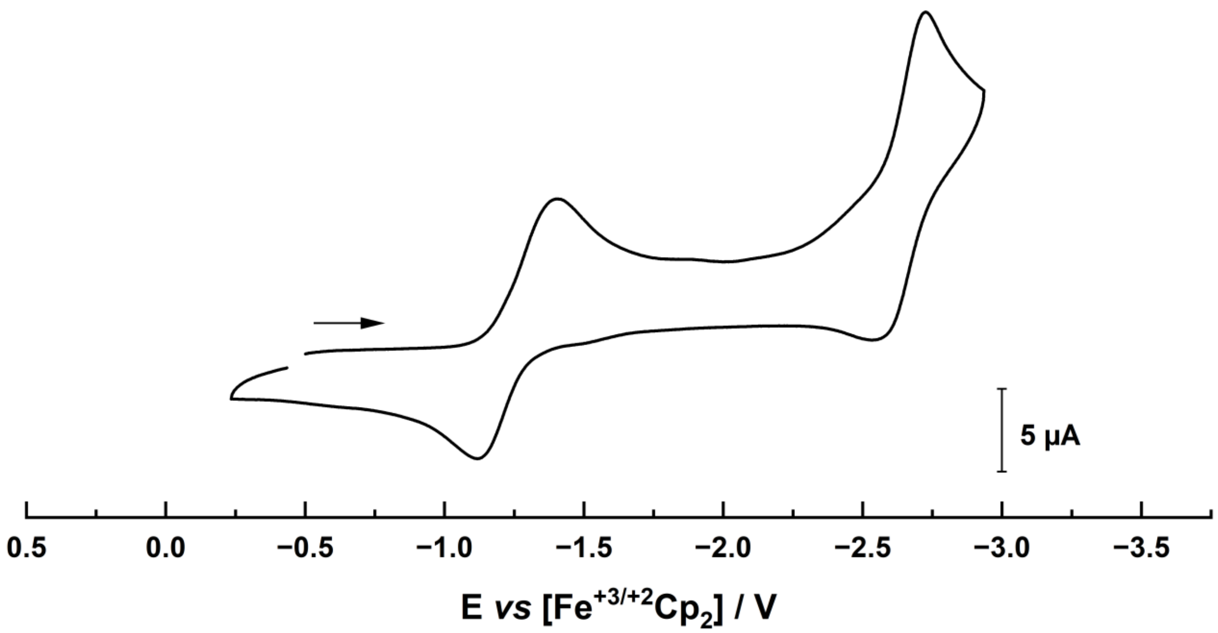

*Figure S 113: Cyclic voltammogram of **1-O^{t}Bu^{F9}** in 0.15 M NBu$_4$PF$_6$ THF solution at a scan rate of 100 mV s^{-1}.*

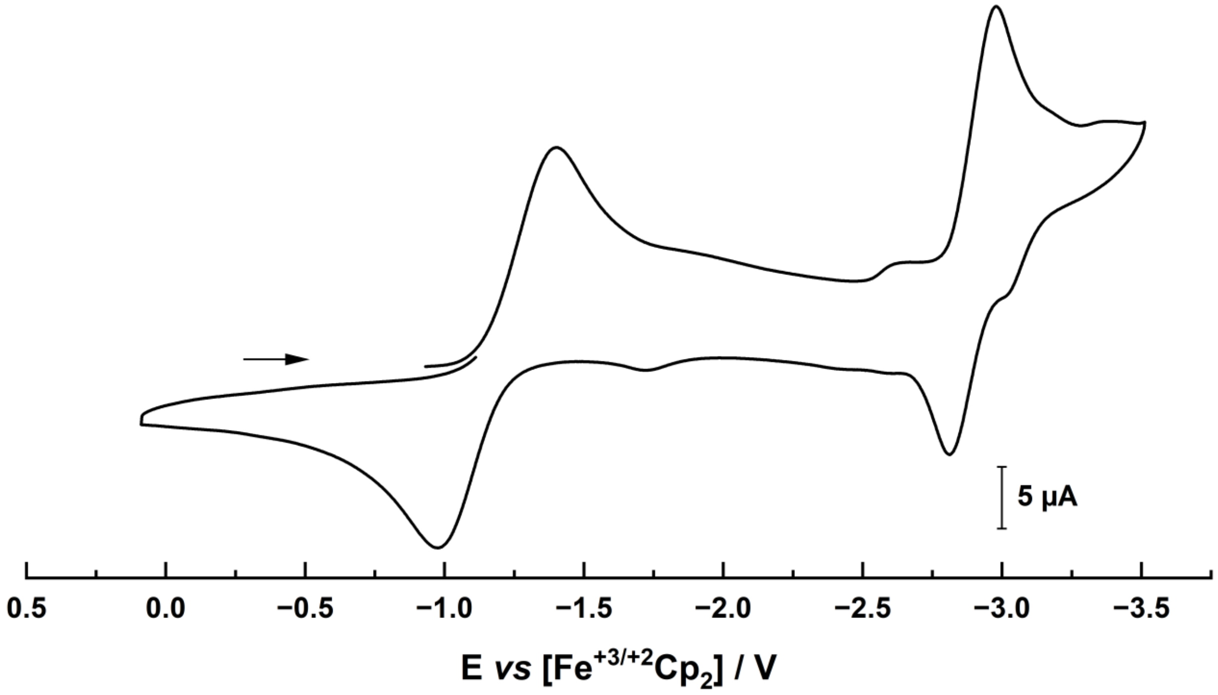

*Figure S 114: Cyclic voltammogram of **2-O^{t}Bu^{F9}** in 0.15 M NBu$_4$PF$_6$ THF solution at a scan rate of 100 mV s^{-1}.*

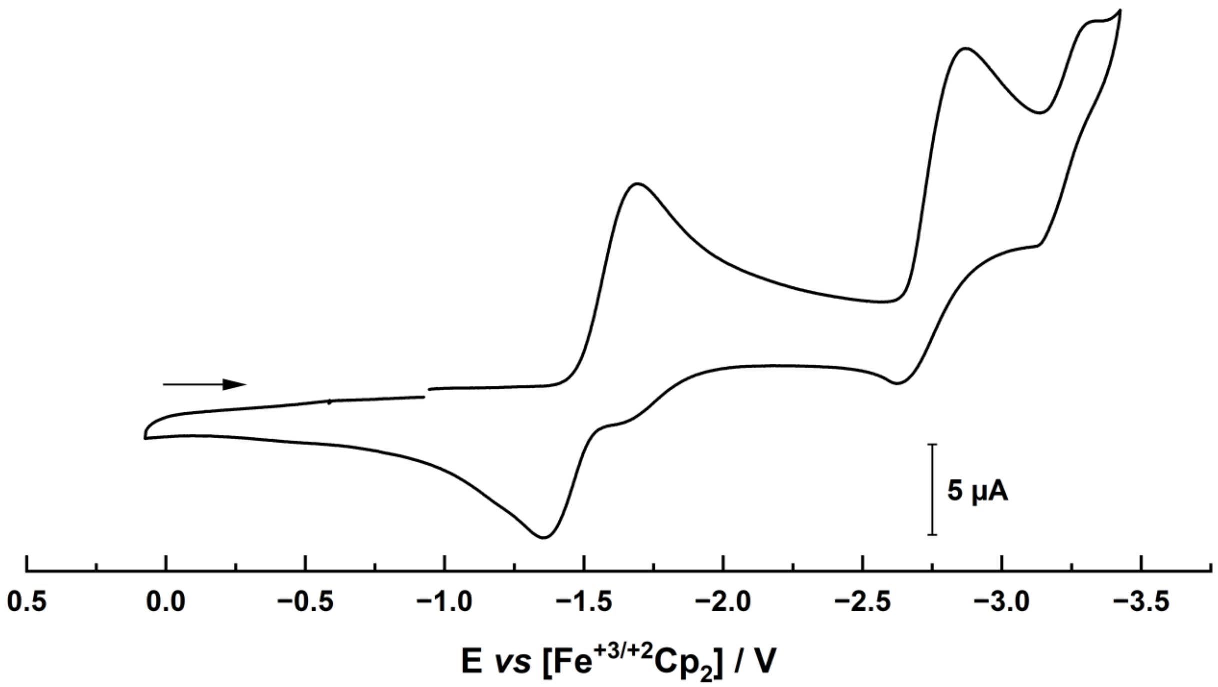

*Figure S 115: Cyclic voltammogram of **1-S^{t}Bu** in 0.15 M NBu_4PF_6 THF solution at a scan rate of 100 mV s^{-1}.*

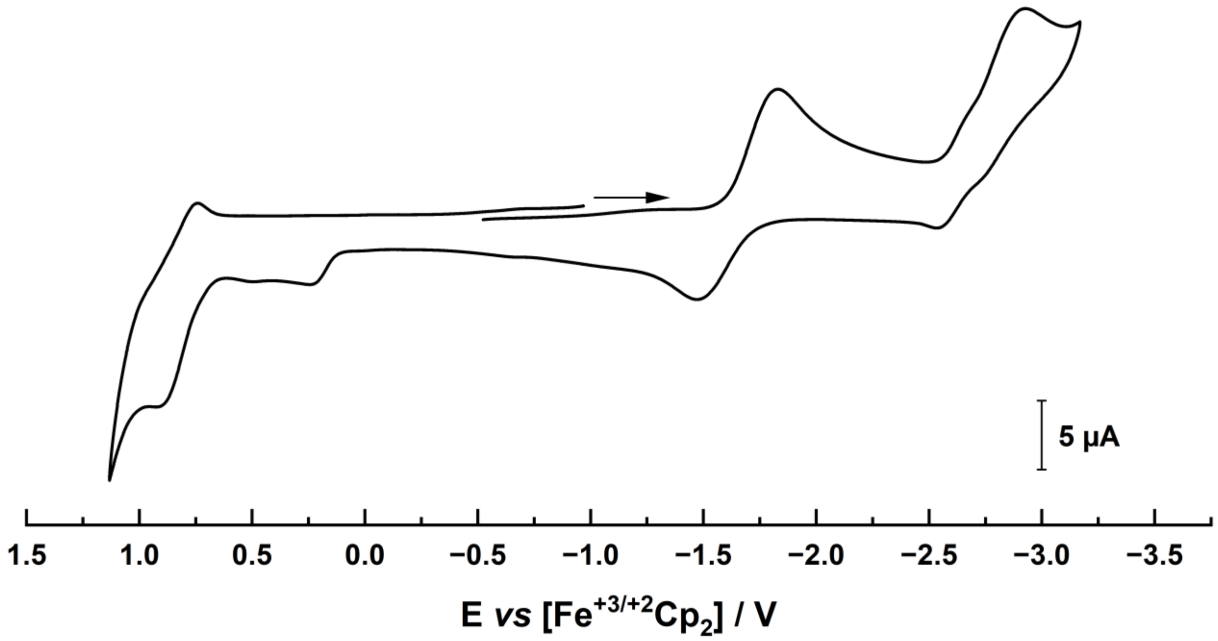

*Figure S 116: Cyclic voltammogram of **1-OCPh$_3$** in 0.15 M NBu_4PF_6 THF solution at a scan rate of 100 mV s^{-1}.*

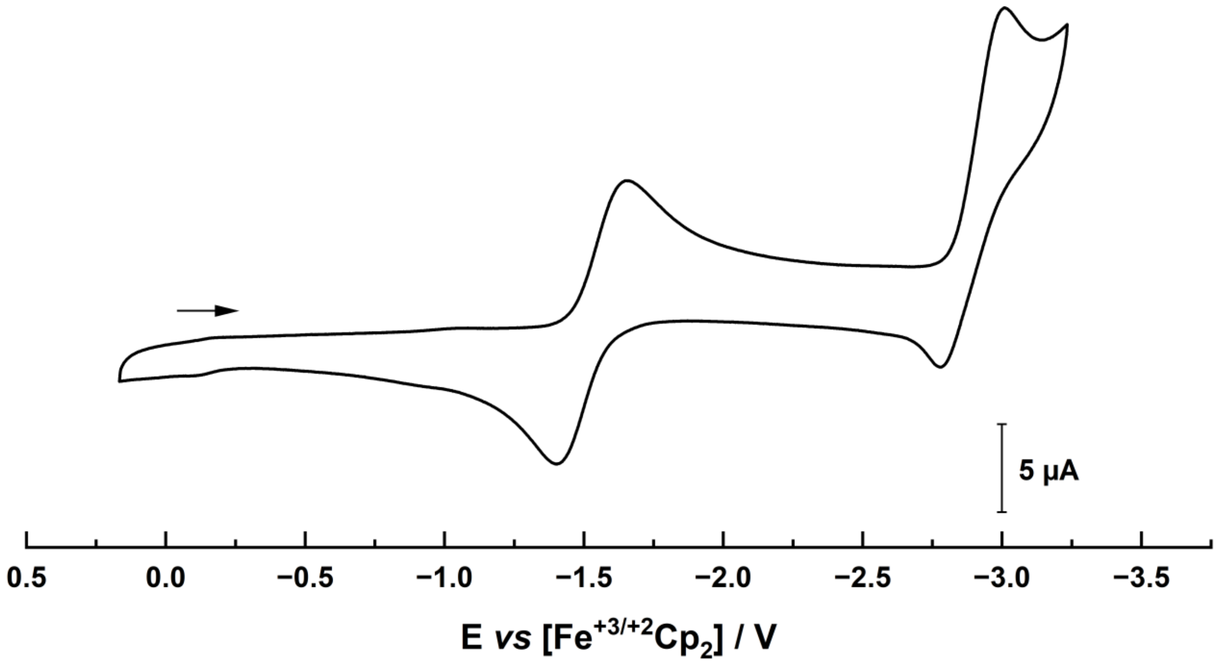

*Figure S 117: Cyclic voltammogram of **2-OCPh₃** in 0.15 M NBu_4PF_6 THF solution at a scan rate of 100 mV s^{-1}.*

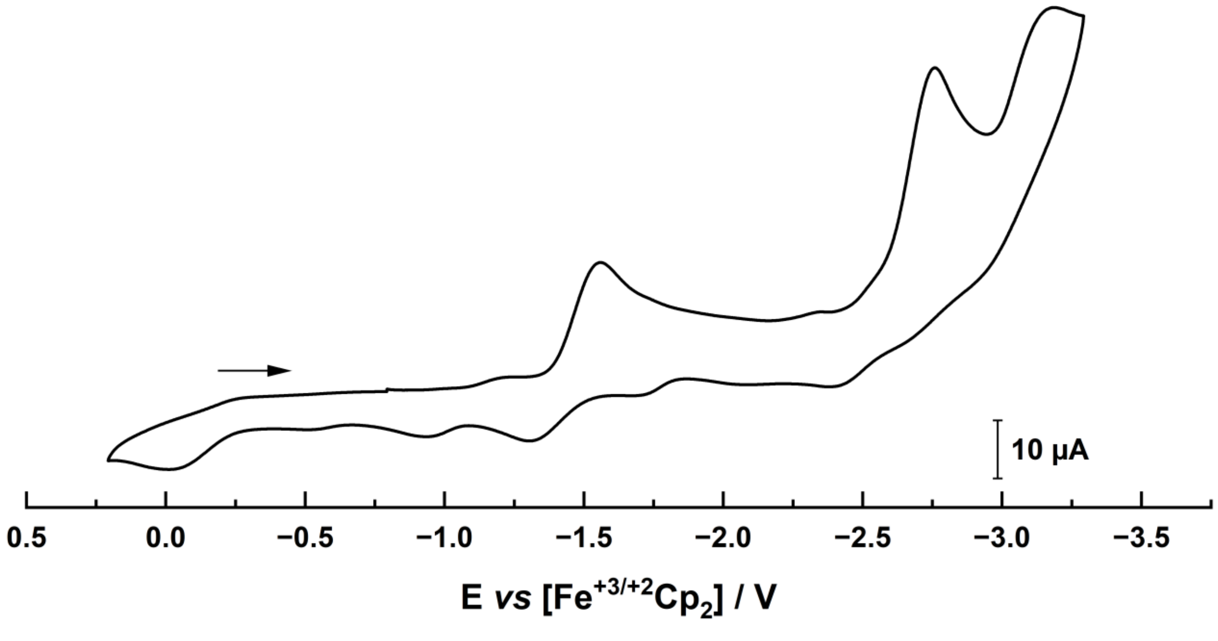

*Figure S 118: Cyclic voltammogram of **1-SCPh$_3$** in 0.15 M NBu_4PF_6 THF solution at a scan rate of 100 mV s^{-1}.*

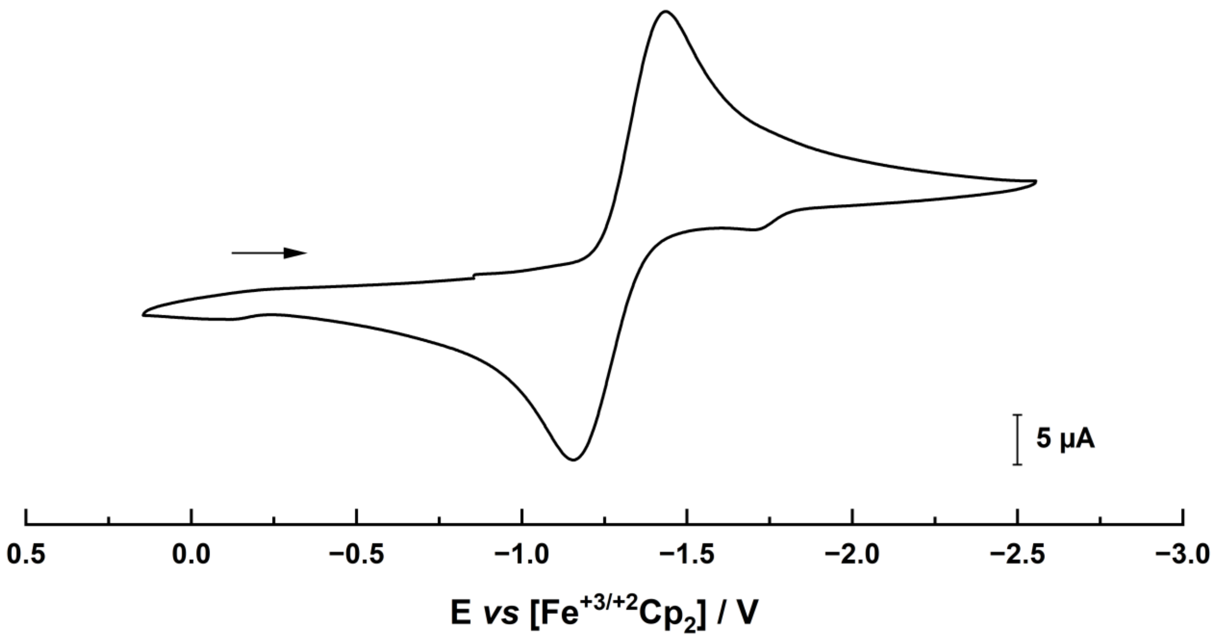

*Figure S 119: Cyclic voltammogram of **2-SCPh₃** in 0.15 M NBu_4PF_6 THF solution at a scan rate of 100 mV s^{-1}.*

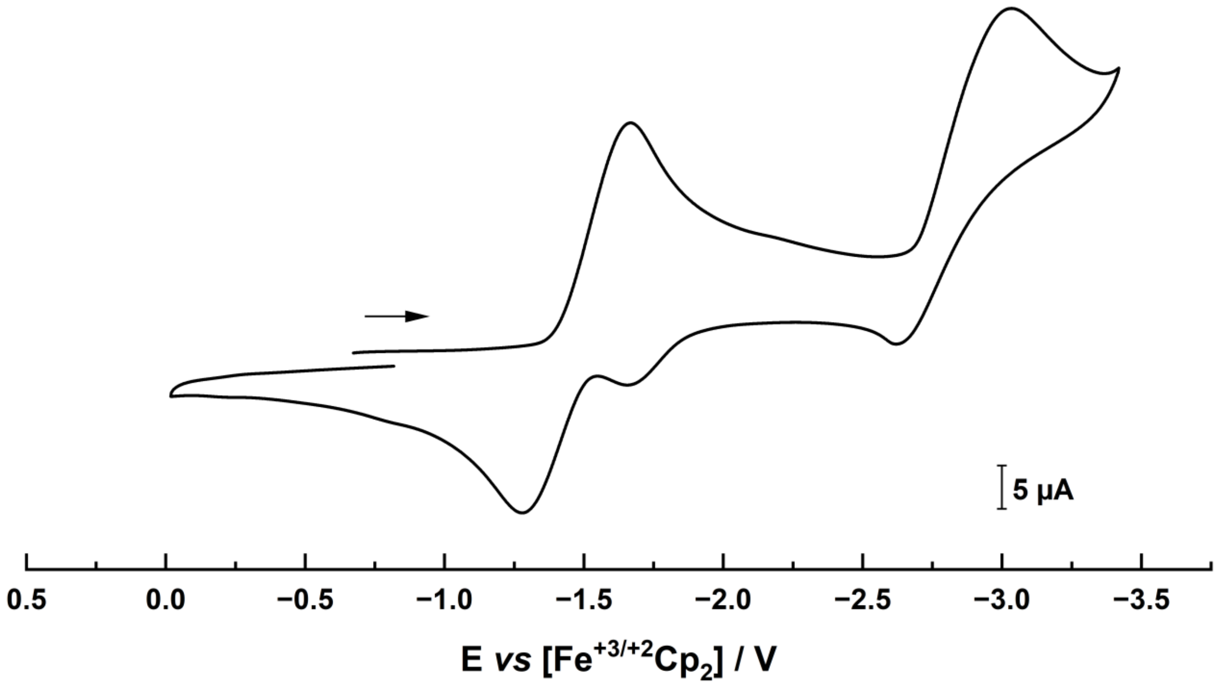

*Figure S 120: Cyclic voltammogram of **1-OMes** in 0.15 M NBu_4PF_6 THF solution at a scan rate of 100 mV s^{-1}.*

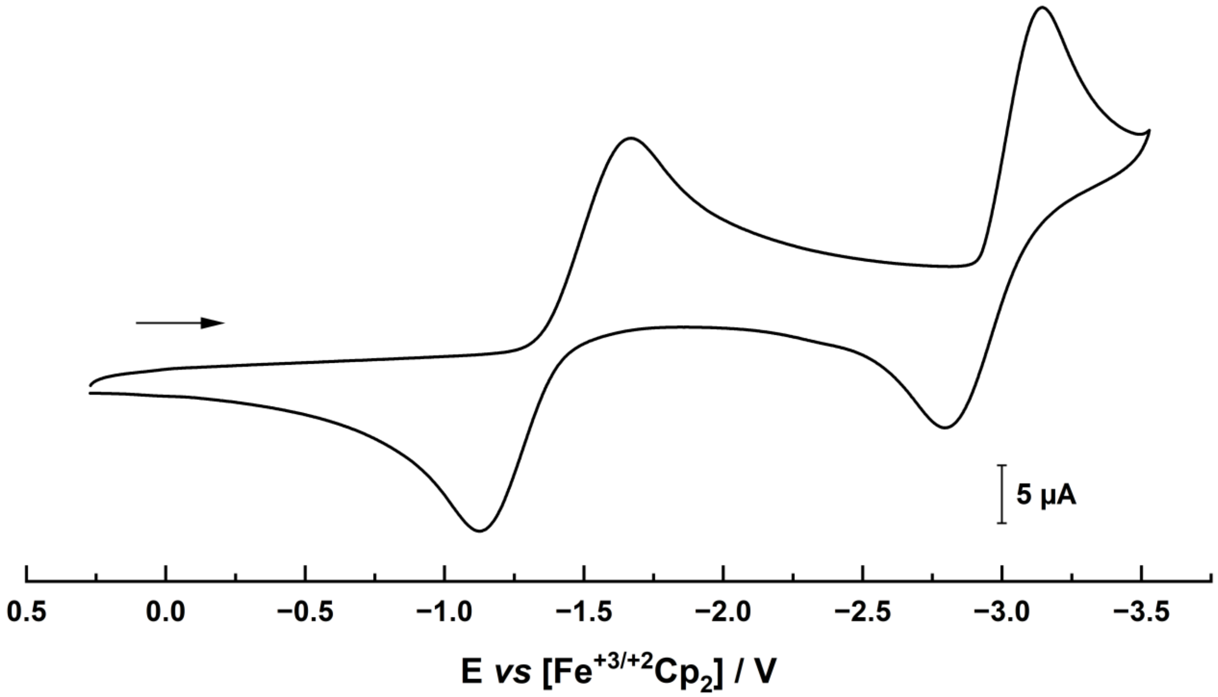

*Figure S 121: Cyclic voltammogram of **2-OMes** in 0.15 M NBu_4PF_6 THF solution at a scan rate of 100 mV s^{-1}.*

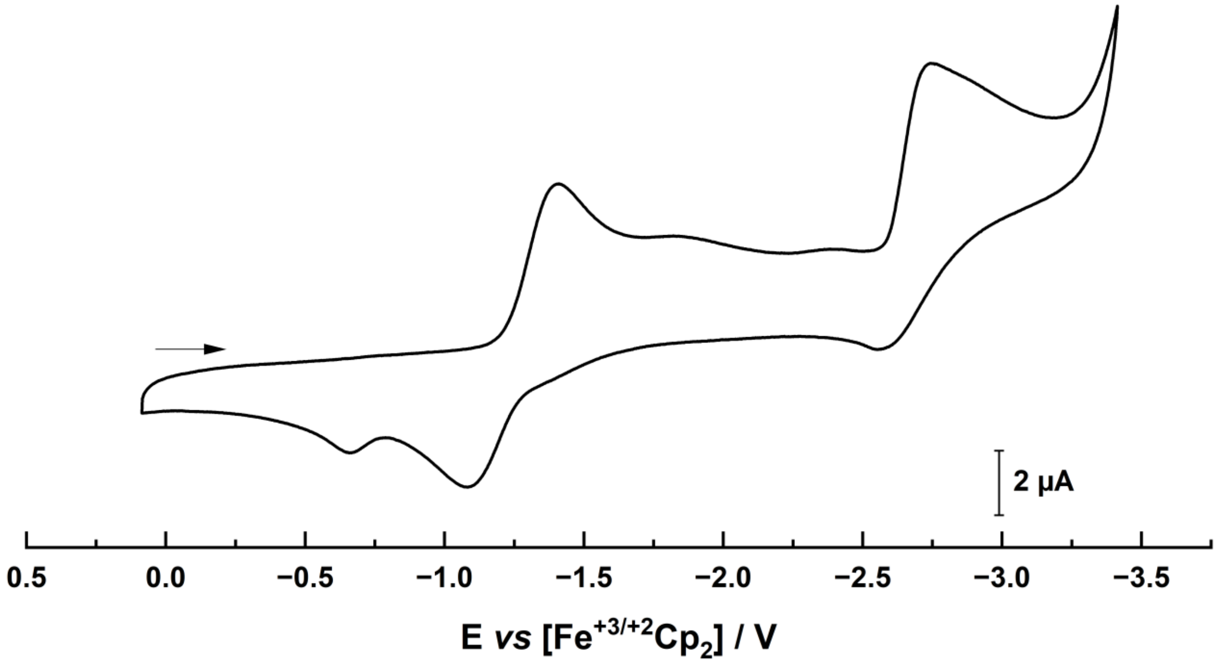

*Figure S 122: Cyclic voltammogram of **1-SMes** in 0.15 M NBu_4PF_6 THF solution at a scan rate of 100 mV s^{-1}.*

5. Computational Details

Calculations were performed using the ORCA v5.0.4 software package.[1] Geometric parameters were optimized from the x-ray diffraction obtained structures on the ZORA-PBE-D3BJ level using the ZORA-def2-SVP basis set[2] for all atoms except molybdenum for which the all electron SARC-ZORA-TZVP set was used.[3] The final single point energy was calculated using the ZORA-def2-TZVP and SARC-ZORA-TZVPP basis sets for all other elements and molybdenum respectively. Scalar relativistic effects were modeled with the "Zeroth Order Regular Approximation" (ZORA).[4] The RI approximation was used with the SARC/J auxiliary basis set.[5] The optimized structures were verified as true minima by the absence of negative eigenvalues in the harmonic vibrational frequency analysis.

1^-

Mo	7.027779	7.171852	19.018112
Cl	8.870647	8.477580	18.125386
O	7.931570	7.124924	20.877023
O	6.668131	6.332661	17.170780
N	6.322977	4.780053	20.938485
N	5.811930	3.545200	21.050014
N	5.645576	3.153317	19.775046
N	5.677424	8.087360	19.324751
C	6.464082	5.214058	19.638767
C	6.065395	4.110897	18.859418
C	5.871814	2.768972	16.735425
H	5.772553	1.858923	17.327858
C	5.840687	2.667245	15.343814
C	6.074209	4.001732	17.407682
C	6.660628	5.497555	22.119962
C	6.264550	5.107647	15.190765
C	6.345696	5.192718	16.631383
C	6.228916	4.989149	23.360101
H	5.620236	4.081673	23.345718
C	6.016505	3.863848	14.605215
H	5.975137	3.804514	13.511895
C	7.481274	6.667435	22.017575
C	6.482634	6.374779	14.342203
C	7.379275	6.789423	24.458163
H	7.653368	7.300796	25.387248
C	6.580125	5.623919	24.553926
C	5.630303	1.335423	14.603229
C	7.830384	7.331935	23.249029
C	5.447169	7.456219	14.738543
H	4.418475	7.096002	14.551474
H	5.606778	8.369731	14.134928
H	5.539326	7.721451	15.803064
C	8.665304	8.626083	23.199342
C	6.137635	5.111067	25.934962
C	7.882201	9.710096	22.417845

H	7.670421	9.387834	21.386502
H	8.472447	10.645244	22.374823
H	6.921341	9.930120	22.917650
C	10.025951	8.366633	22.507107
H	10.585385	7.569779	23.031983
H	10.639740	9.287512	22.527470
H	9.880442	8.067119	21.457392
C	7.918112	6.918065	14.555460
H	8.093302	7.224434	15.599711
H	8.084525	7.798595	13.905616
H	8.665006	6.148865	14.283779
C	5.318550	6.206352	26.657408
H	5.907340	7.130938	26.789424
H	4.998152	5.861671	27.659582
H	4.417154	6.467115	26.074342
C	8.952729	9.175341	24.610473
H	8.023689	9.425593	25.155459
H	9.547599	10.102902	24.525112
H	9.533054	8.460869	25.223956
C	5.264533	3.847627	25.829398
H	4.346775	4.037506	25.244558
H	4.960056	3.512681	26.838305
H	5.809997	3.016361	25.347588
C	4.953220	1.892834	19.590482
H	5.647259	1.080855	19.307194
H	4.181740	1.995530	18.810087
H	4.478866	1.641819	20.551440
C	6.307054	6.091601	12.837066
H	7.051285	5.365372	12.460271
H	6.446346	7.031451	12.272431
H	5.297071	5.706437	12.602321
C	7.387823	4.767829	26.779298
H	7.984936	3.980872	26.284682
H	7.098845	4.406520	27.785226
H	8.039896	5.649329	26.908443
H	4.407746	2.243339	13.009258
C	6.841936	1.053760	13.684040
H	6.703794	0.105844	13.128839
H	7.770772	0.977424	14.277309
C	5.480170	0.147525	15.571606
H	5.331935	-0.788834	15.002752
H	4.609296	0.274284	16.240466
H	6.985755	1.861402	12.945125
C	4.347049	1.421125	13.743592
H	3.464134	1.609352	14.380585
H	4.180591	0.478977	13.186267
H	6.380684	0.019761	16.199494

2^-

Mo	3.135411	4.239936	7.508071
Cl	3.597551	2.950281	5.501103
O	2.624647	2.455054	8.436086
O	4.845016	5.348405	7.092467
N	3.396110	4.045609	10.657877
N	4.572153	5.743136	9.881405
C	3.640382	4.794943	9.529628
C	2.070542	2.242165	9.603360
N	1.799758	5.203388	7.276896
C	4.995569	5.543062	11.201056
C	5.767202	7.533374	6.796141
C	0.783929	0.264402	8.584746
C	5.040435	6.775745	9.010793
C	5.506524	4.516157	13.743444
H	5.729279	4.115104	14.739546
C	4.241630	4.448351	11.700344
C	0.521741	1.002437	11.037203
H	-0.198549	0.188430	11.150703
C	2.386907	3.037304	10.753594
C	5.330178	8.040739	9.552198
H	5.097158	8.217507	10.606106
C	1.712028	2.864894	11.970951
H	1.914207	3.571997	12.783480
C	5.191614	6.498364	7.616149
C	6.202909	10.458770	9.299610
C	6.254209	5.606985	13.247400
H	7.049957	6.042798	13.863500
C	1.128706	1.171559	9.781672
C	0.111893	1.108630	7.473032
H	-0.793631	1.610950	7.859284
H	-0.188750	0.454579	6.632246
H	0.798859	1.875119	7.081024
C	6.088566	8.760025	7.391270
H	6.516148	9.544636	6.757354
C	6.017565	6.129000	11.968048
H	6.626650	6.942251	11.562810
C	5.983227	7.274263	5.293098
C	0.048191	1.689408	13.496966
C	0.768378	1.840249	12.144974
C	-0.774632	2.966358	13.787803
H	-0.130014	3.862062	13.825610
H	-1.300382	2.884845	14.758774
H	-1.527842	3.133125	12.997211
C	2.067521	-0.395216	8.023341
H	2.765625	0.370215	7.648572
H	1.806154	-1.069550	7.185831
H	2.572676	-0.995489	8.803072

C	4.498619	3.915719	12.975629
H	3.953890	3.039460	13.338736
C	5.865497	9.060347	8.755452
C	5.879855	10.591352	10.799021
H	4.805403	10.431252	11.000439
H	6.140235	11.605754	11.153345
H	6.452942	9.867254	11.406494
C	1.092292	1.490425	14.621286
H	1.696520	0.584162	14.435663
H	0.597826	1.383301	15.605915
H	1.784438	2.348713	14.685461
C	-0.909728	0.484208	13.512279
H	-1.703801	0.586143	12.751172
H	-1.399254	0.403991	14.500449
H	-0.374944	-0.463800	13.322250
C	6.658241	8.472789	4.598238
H	6.039224	9.387657	4.649951
H	6.808969	8.234816	3.529582
H	7.649506	8.699576	5.033826
C	7.712545	10.735484	9.105118
H	8.318612	9.991406	9.652905
H	7.979533	11.743390	9.477310
H	8.000057	10.679358	8.040612
C	5.385238	11.524693	8.533140
H	5.596722	11.493268	7.450118
H	5.624201	12.542356	8.897711
H	4.302078	11.353006	8.665191
C	6.890486	6.035519	5.092649
H	7.868570	6.181334	5.588115
H	7.072421	5.874251	4.013053
H	6.408621	5.133887	5.503211
C	-0.191597	-0.860866	8.981671
H	0.224738	-1.512223	9.772902
H	-0.392980	-1.494252	8.098621
H	-1.162349	-0.465562	9.334018
C	4.617403	7.025893	4.605612
H	4.132780	6.115961	4.992940
H	4.763959	6.899109	3.515904
H	3.937228	7.882110	4.765164

6. Crystallographic Information

Table S 1: Crystallographic Details of Complexes 1-Cl, 2-Cl, [1-Cl]⁻, [2-Cl]⁻, 3, 4, 1-OtBu, 2-OtBu and 1-OtBu^{F9}.

	**1-Cl**	**2-Cl**	**[1-Cl]$^-$**	**[2-Cl]$^-$**	**3**	**4**	**1-O^{t}Bu**	**2-O^{t}Bu**	**1-O^{t}Bu^{F9}**
Chemical Formula	$C_{31}H_{43}ClMoN_4O_2$<br>$0.5(C_6H_6)$	$C_{35}H_{44}ClMoN_3O_2$	$C_{31}H_{43}ClMoN_4O_2$<br>$C_{20}H_{30}Co$<br>CH_2Cl_2	$C_{35}H_{44}ClMoN_3O_2$<br>$C_{20}H_{30}Co$<br>CH_2Cl_2	$C_{62}H_{86}Cl_2Mo_2N_6O_4$<br>$0.75(C_7H_8)$	$C_{70}H_{88}Cl_2Mo_2N_4O_4$<br>$0.6[C_4H_{10}O]$	$C_{35}H_{52}MoN_4O_3$	$C_{39}H_{53}MoN_3O_3$	$C_{35}H_{43}N_4O_3F_9Mo$<br>C_4H8O<br>$0.9\ C_4H_{10}O$
M_r (g mol^{-1})	674.14	670.12	1049.38	1084.41	1311.24	1356.69	672.74	707.78	973.48
Crystal System	orthorhombic	orthorhombic	orthorhombic	monoclinic	triclinic	triclinic	orthorhombic	orthorhombic	Monoclinic
Space Group	C m c e	P b c a	P b c a	C 1 c 1	P -1	P -1	P b c m	P 21 21 21	$P2_1/n$
a (Å)	32.4454(15)	13.7921(6)	11.2229(8)	19.5656(5)	10.0665(10)	13.0612(17)	5.9259(3)	5.8275(12)	16.7763(10)
b (Å)	9.7068(4)	16.5408(8)	18.4476(14)	15.4251(4)	10.9440(11)	16.0524(18)	19.4438(7)	19.533(4)	10.5017(6)
c (Å)	21.5529(9)	29.0621(13)	51.454(4)	19.5452(5)	30.514(3)	18.038(2)	31.0776(13)	31.857(7)	28.0439(18)
α (°)	90	90	90	90	90.214(4)	103.777(6)	90	90	90
β (°)	90	90	90	112.7890(10)	97.829(4)	100.008(6)	90	90	96.409(2)
γ (°)	90	90	90	90	94.020(4)	104.777(6)	90	90	90
V (Å^3)	6787.9(5)	6630.0(5)	10652.8(13)	5438.3(2)	3321.8(6)	3439.1(7)	3580.8(3)	3626.2(13)	4909.9(5)
Z	8	8	8	4	2	2	4	4	4
Density (g cm^{-3})	1.319	1.343	1.309	1.324	1.311	1.310	1.248	1.296	1.317
F(000)	2824	2800	4408	2276	1375	1422	1424	1496	2023
Radiation Type	Mo$K\alpha$	Mo$K\alpha$	Mo$K\alpha$	Mo$K\alpha$	Mo$K\alpha$	Mo$K\alpha$	Mo$K\alpha$	Mo$K\alpha$	Mo$K\alpha$
μ (mm^{-1})	0.500	0.511	0.738	0.725	0.508	0.493	0.403	0.401	0.344
Crystal Size (mm)	0.48x0.11x0.09	0.35x0.32x0.02	0.4x0.38x0.25	0.05x0.04x0.02	0.15x0.11x0.04	0.35x0.01x0.005	0.25x0.1x0.08	0.13x0.02x0.01	0.10x0.08x0.01
Meas. Refl.	69352	95100	66020	78827	13717	45614	39210	35165	67721
Indep. Refl.	3069	6071	10874	10329	13717	12246	3746	6425	8700
Obsvd. $[I > 2\sigma(I)]$	2605	5020	8776	10139	12646	6189	3286	3816	6362
R_{int}	0.0789	0.0620	0.0560	0.0480	?	0.2026	0.0540	0.2076	0.1059
R [$F^2 > 2\sigma(F^2)$]	0.0377	0.0311	0.0437	0.0212	0.0776	0.0718	0.0275	0.0673	0.0492
wR(F^2)	0.1021	0.0774	0.0980	0.0516	0.2007	0.1879	0.0671	0.1459	0.1407
S	1.134	1.100	1.082	1.048	1.144	0.922	1.067	0.985	1.044
$\Delta\rho_{max}$	1.427	0.782	0.738	0.399	1.578	0.709	0.263	0.653	0.475
$\Delta\rho_{min}$	-0.598	-0.519	-0.534	-0.296	-1.559	-0.866	-0.358	-0.378	-0.894
CCDC	2364769	2364763	1364770	2364765	2364775	2364773	2364774	2364760	2364772

Table S 2: Crystallographic Details of Complexes 1-R (R = S^{t}Bu, SCPh$_3$, OMes, SMes) and 2-R (R = OtBu^{F9}, OCPh$_3$, OMes, SMes).

	**2-O^{t}Bu^{F9}**	**1-S^{t}Bu**	**2-OCPh$_3$**	**1-SCPh$_3$**	**1-OMes**	**2-OMes**	**1-SMes**	**2-SMes**
Chemical Formula	$C_{39}H_{44}N_3O_3F_9Mo$	$C_{35}H_{52}N_4O_2SMo$	$C_{54}H_{59}N_3O_3Mo$	$C_{50}H_{58}N_4O_2SMo$	$C_{40}H_{54}N_4O_3Mo$	$C_{44}H_{55}N_3O_3Mo$	$C_{40}H_{54}N_4O_2SMo$	$C_{44}H_{55}N_3O_2SMo$
	C_4H_8O							
	0.6 ($C_4H_{10}O$)	0.167 (C_6H_{14})	1.2 C_5H_{12}		1.5 C_6H_6		C_6H_{14}	C_5H_{12}
M_r (g mol^{-1})	986.28	703.16	980.55	875.00	851.97	769.85	837.04	858.05
Crystal System	Orthorhombic	Triclinic	Monoclinic	Triclinic	Triclinic	Monoclinic	Triclinic	Trigonal
Space Group	*Pnnm*	*P*-1	$P2_1/c$	*P*1	*P*-1	C_2/c	*P*-1	*R*-3
a (Å)	23.4925(19)	19.0492(14)	13.5078(7)	9.3240(16)	12.3406(6)	33.202(4)	16.754(2)	37.041(2)
b (Å)	9.8772(9)	19.4669(16)	28.4083(15)	11.1134(18)	13.6168(7)	13.0643(10)	17.595(2)	37.041(2)
c (Å)	20.6824(15)	20.4769(17)	13.9736(7)	23.386(4)	15.6475(9)	18.5211(17)	18.362(2)	21.5515(16)
α (°)	90	102.384(4)	90	81.921(5)	95.467(2)	90	65.177(59	90
β (°)	90	115.020(3)	95.292(2)	85.646(5)	107.910(2)	93.051(3)	68.890(5)	90
γ (°)	90	101.929(4)	90	86.098(5)	109.900(2)	90	81.949(5)	120
V (Å^3)	4799.1(7)	6330.0(9)	5339.3(5)	2388.2(7)	2293.1(2)	8022.4(13)	4582.7(10)	25608(3)
Z	4	6	4	2	2	8	4	18
Density (g cm^{-3})	1.365	1.107	1.220	1.217	1.234	1.275	1.213	1.002
F(000)	2045	2234	2082	920	902	3248	1784	8208
Radiation Type	Mo$K\alpha$	Mo$K\alpha$	Mo$K\alpha$	Mo$K\alpha$	Mo$K\alpha$	Mo$K\alpha$	Mo$K\alpha$	Mo$K\alpha$
μ (mm^{-1})	0.353	0.391	0.292	0.359	0.330	0.369	0.371	0.300
Crystal Size (mm)	0.24x0.19x0.02	0.02x0.01x0.005	0.10x0.09x0.05	0.20x0.19x0.01	0.25x0.23x0.08	0.25x0.20x0.04	0.08x0.04x0.02	0.34x0.21x0.18
Meas. Refl.	100217	135451	107140	16723	78746	44014	75311	242159
Indep. Refl.	4844	22463	9449	16723	9426	8212	16173	12577
Obsvd. $[I > 2\sigma(I)]$	3665	10622	7267	15095	7786	5487	8411	8512
R_{int}	0.1261	0.2740	0.0806	-	0.0968	0.1445	0.1975	0.1711
R [$F^2 > 2\sigma(F^2)$]	0.0543	0.0716	0.0544	0.0625	0.0432	0.0540	0.0680	0.0582
wR(F^2)	0.1706	0.1946	0.1457	0.1622	0.1114	0.1360	0.1828	0.1722
S	1.064	0.939	1.053	1.042	1.040	1.027	0.958	1.000
$\Delta\rho_{max}$	0.828	1.549	0.598	1.590	0.656	0.680	0.377	0.647
$\Delta\rho_{min}$	-0.886	-0.576	-0.657	-0.873	-0.586	-0.663	-0.823	-0.807
CCDC	2364764	2364766	2364768	2364759	2364771	2364761	2364762	2364767

*Table S 3: Selected bond lengths and angles for the complexes 1-R (R = Cl, O^{t}Bu, OtBu^{F9}, S^{t}Bu, SCPh$_3$, OMes, SMes) and 2-R (R = Cl, O^{t}Bu, OtBu^{F9}, OCPh$_3$, OMes, SMes) as well as reduced complexes **[1]$^-$** and **[2]$^-$** and dimers **3** and **4.***

**Atoms**	**1-Cl**	**2-Cl**	**[1]$^-$**	**[2]$^-$**	**3**	**4**	**1-O^{t}Bu**	**2-O^{t}Bu**	**1-O^{t}Bu^{F9}**
Mo1 – Mo1A	-	-	-	-	2.2707(8)	2.2726(10)	-	-	-
Mo1 – C1	2.145(4)	2.208(2)	2.115(3)	2.173(3)	2.142(6)	2.177(7)	2.188(2)	2.256(11)	2.163(3)
Mo1 – O1	1.9268(19)	1.9217(18)	2.0422(19)	2.0585(18)	1.929(5)	1.921(5)	1.9650(12)	1.945(7)	1.942(2)
Mo1 – O2	1.9269(19)	1.9203(18)	2.0655(19)	2.0477(19)	1.929(5)	1.918(5)	1.9651(12)	1.959(8)	1.950(2)
Mo1 – N10	1.642(4)	1.647(2)	1.654(3)	1.657(2)	-	-	1.648(2)	1.641(8)	1.645(3)
Mo1 – X*	2.3714(11)	2.3502(7)	2.4559(8)	2.4520(7)	2.3643(18)	2.3399(19)	1.8987(17)	1.892(6)	1.997(2)
C1 – Mo1 – X	161.74(10)	157.88(7)	147.83(8)	146.80(7)	157.31(18)	159.1(2)	153.20((9)	156.2(3)	158.5911)
O1 – Mo1 – O2	141.21(13)	140.72(8)	145.93(8)	142.13(8)	147.7(2)	140.4(2)	145.94(8)	143.7(3)	149.61(10)
C1 – Mo1 – N10/Mo1A	92.99(16)	94.49(10)	102.61(11)	104.15(11)	96.71(17)	96.3(2)	98.68(10)	97.3(4)	94.75(14)
N10/Mo1A – Mo1 – X*	105.30(13)	107.62(8)	109.40(9)	109.04(9)	105.76(6)	104.55(6)	108.13(10)	106.5(4)	106.66(13)
Mo1 – X – C40	-	-	-	-	-	-	141.87(17)	142.4(6)	152.0(2)
τ_5	0.34	0.29	0.03	0.08	0.16	0.31	0.12	0.21	0.15

	**2-O^{t}Bu^{F9}**	**1-S^{t}Bu**	**2-OCPh$_3$**	**1-SCPh$_3$**	**1-OMes**	**2-OMes**	**1-SMes**	**2-SMes**	
Mo1 – Mo1A	-	-	-	-	-	-	-	-	
Mo1 – C1	2.221(5)	2.179(7)	2.227(4)	2.178(10)	2.154(3)	2.201(4)	2.183(6)	2.225(3)	
Mo1 – O1	1.957(2)	1.958(4)	1.954(3)	1.947(7)	1.9271(19)	1.970(3)	1.941(4)	1.954(2)	
Mo1 – O2	1.957(2)	1.970(5)	1.945(3)	1.947(7)	1.9734(19)	1.941(3)	1.977(4)	1.946(2)	
Mo1 – N10	1.638(5)	1.654(6)	1.639(3)	1.639(8)	1.644(2)	1.650(4)	1.657(5)	1.639(2)	
Mo1 – X*	1.979(4)	2.360(2)	1.929(2)	2.369(3)	1.9378(17)	1.922(3)	2.3767(17)	2.3529(8)	
C1 – Mo1 – X	157.06(18)	166.6(2)	151.56(13)	156.1(3)	159.09(9)	157.85(15)	165.92(17)	163.45(8)	
O1 – Mo1 – O2	149.49(16)	141.53(19)	148.22(11)	150.5(3)	140.65(8)	138.39(13)	142.88(18)	140.73(8)	
C1 – Mo1 – N10	96.5(2)	96.7(3)	101.22(15)	103.3(4)	94.71(11)	92.50(16)	96.6(3)	97.39(12)	
N10 – Mo1 – X*	106.4(2)	98.43(14)	107.22(14)	100.6(3)	105.44(10)	109.27(15)	97.09(19)	98.74(9)	
Mo1 – X – C40	155.6(4)	112.2(3)	126.1(2)	107.5(3)	138.89(17)	158.3(3)	118.1(2)	116.17(10)	
τ_5	0.13	0.42	0.06	0.09	0.14	0.32	0.38	0.38	

*X = Cl1, O40, S40

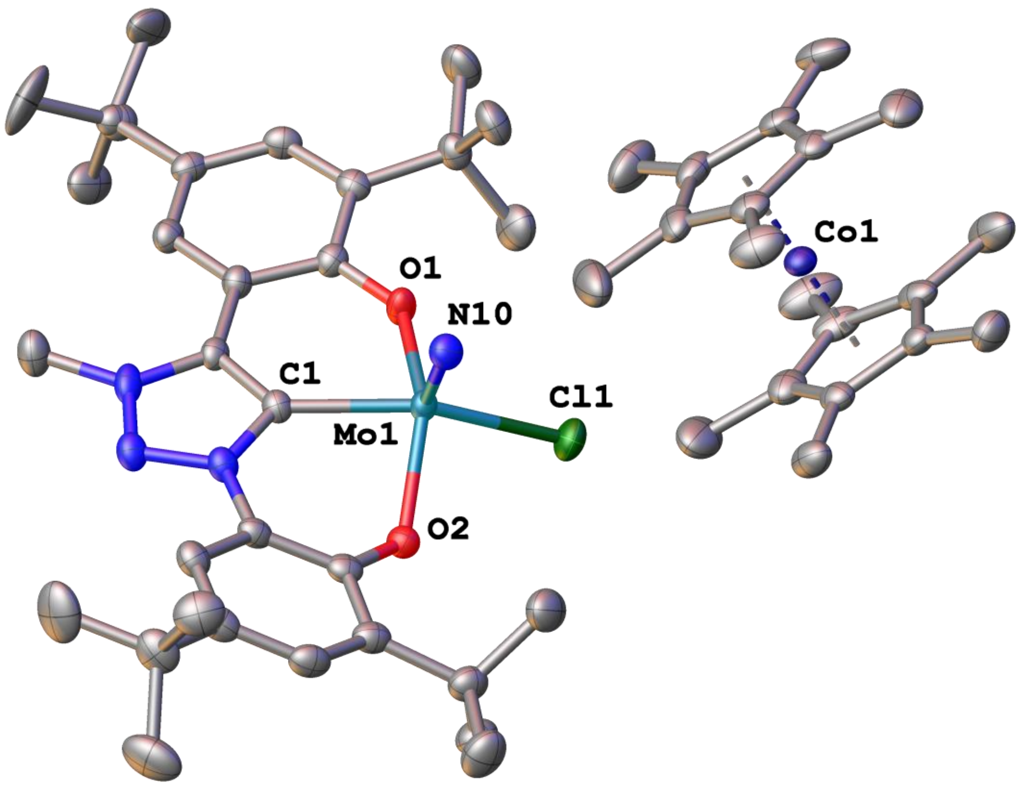

Figure S 123: Full molecular structure of the anionic complex ***[1-Cl]***$^{-}$ *including the decamethyl cobaltocenium counterion. Hydrogen atoms and lattice solvent molecules have been omitted for clarity. Ellipsoids are shown at a probability level of 50%.*

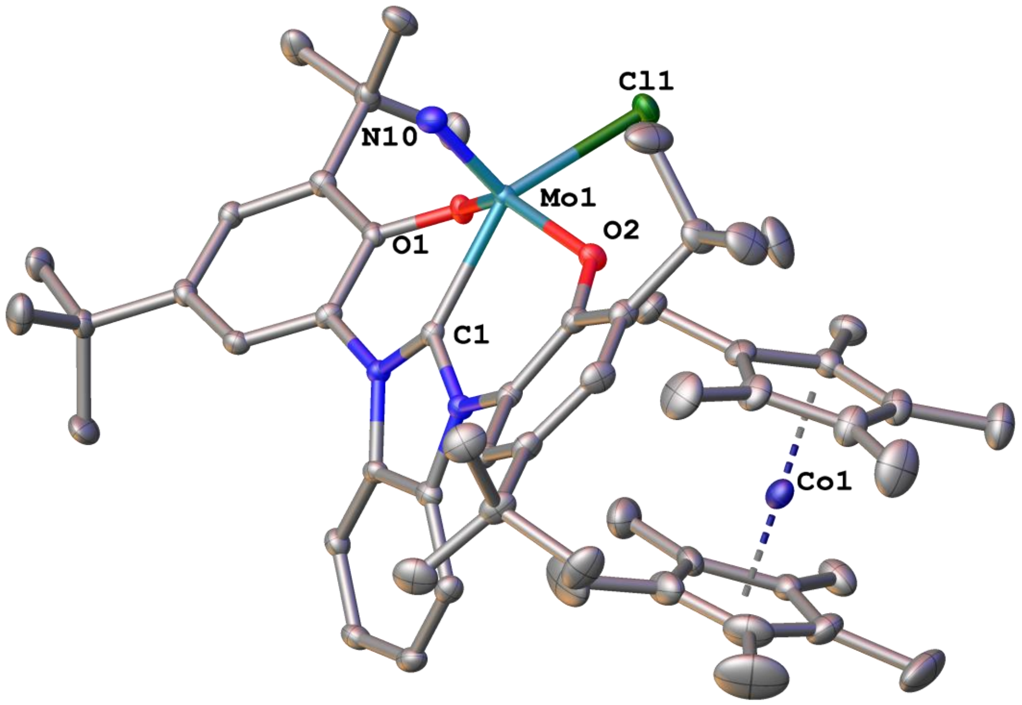

Figure S 124: Full molecular structure of the anionic complex ***[2-Cl]***$^{-}$ *including the decamethyl cobaltocenium counterion. Hydrogen atoms and lattice solvent molecules have been omitted for clarity. Ellipsoids are shown at a probability level of 50%.*

7. References

[1] F. Neese, *WIREs*, **2022**, *12*, e1606.

[2] a) S. Grimme, S. Ehrlich, L. Goerigk, *J. Comput. Chem.* **2011**, *32*, 1456-1465; b) S. Grimme, J. Antony, S. Ehrlich, H. Krieg, *J. Chem. Phys.* **2010**, *132*, 154104; d) J. P. Perdew, *Phys. Rev. B* **1986**, *33*, 8822-8824; e) F. Weigend, R. Ahlrichs, *Phys. Chem. Chem. Phys.* **2005**, *7*, 3297- 3305; g) J. P. Perdew, K. Burke, M. Ernzerhof, *Phys. Rev. Lett.* **1996**, *77*, 3865-3868.

[3] D. A. Pantazis, X.-Y. Chen, C. R. Landis, F. Neese, *J. Chem. Theor. Comput.* **2008**, *4*, 908-919

[4] a) E. v. Lenthe, E. J. Baerends, J. G. Snijders, *J. Chem. Phys.* **1993**, *99*, 4597-4610; b) C. van Wüllen, *J. Chem. Phys.* **1998**, *109*, 392-399.

[5] F. Weigend, *Phys. Chem. Chem. Phys.* **2006**, *8*, 1057-1065.
